# Radiomics and machine learning of multisequence multiparametric prostate MRI: Towards improved non-invasive prostate cancer characterization

**DOI:** 10.1371/journal.pone.0217702

**Published:** 2019-07-08

**Authors:** Jussi Toivonen, Ileana Montoya Perez, Parisa Movahedi, Harri Merisaari, Marko Pesola, Pekka Taimen, Peter J. Boström, Jonne Pohjankukka, Aida Kiviniemi, Tapio Pahikkala, Hannu J. Aronen, Ivan Jambor

**Affiliations:** 1 Dept. of Diagnostic Radiology, University of Turku, Turku, Finland; 2 Dept. of Future Technologies, University of Turku, Turku, Finland; 3 Turku PET Centre, University of Turku, Turku, Finland; 4 Institute of Biomedicine, University of Turku and Dept. of Pathology, Turku University Hospital, Turku, Finland; 5 Dept. of Urology, Turku University Hospital, Turku, Finland; 6 Medical Imaging Centre of Southwest Finland, Turku University Hospital, Turku, Finland; 7 Department of Radiology, Icahn School of Medicine at Mount Sinai, New York, NY, United States of America; Brigham and Women’s and Harvard Medical School, UNITED STATES

## Abstract

**Purpose:**

To develop and validate a classifier system for prediction of prostate cancer (PCa) Gleason score (GS) using radiomics and texture features of T_2_-weighted imaging (T_2_w), diffusion weighted imaging (DWI) acquired using high b values, and T_2_-mapping (T_2_).

**Methods:**

T_2_w, DWI (12 b values, 0–2000 s/mm^2^), and T_2_ data sets of 62 patients with histologically confirmed PCa were acquired at 3T using surface array coils. The DWI data sets were post-processed using monoexponential and kurtosis models, while T_2_w was standardized to a common scale. Local statistics and 8 different radiomics/texture descriptors were utilized at different configurations to extract a total of 7105 unique per-tumor features. Regularized logistic regression with implicit feature selection and leave pair out cross validation was used to discriminate tumors with 3+3 vs >3+3 GS.

**Results:**

In total, 100 PCa lesions were analysed, of those 20 and 80 had GS of 3+3 and >3+3, respectively. The best model performance was obtained by selecting the top 1% features of T_2_w, ADC_m_ and K with ROC AUC of 0.88 (95% CI of 0.82–0.95). Features from T_2_ mapping provided little added value. The most useful texture features were based on the gray-level co-occurrence matrix, Gabor transform, and Zernike moments.

**Conclusion:**

Texture feature analysis of DWI, post-processed using monoexponential and kurtosis models, and T_2_w demonstrated good classification performance for GS of PCa. In multisequence setting, the optimal radiomics based texture extraction methods and parameters differed between different image types.

## 1 Introduction

Prostate cancer (PCa) is the most common cancer in men, and the second most common among the causes of death related to cancer. For example in USA, 161 360 new cases of PCa are estimated to be diagnosed in 2017, while the estimated cancer related deaths are 26 730 [1]. However, in approximately half of the cases of newly diagnosed PCa, the patients have a low risk of death from the disease [2, 3]. Due to this wide range of possible outcomes, it is important to accurately predict the risk caused by PCa and to stratify patients accordingly aiming to limit over-treatment and PCa mortality simultaneously.

The Gleason score is a commonly used marker for estimating the possible threat posed by a PCa lesion, based on histopathological analysis of biopsy and prostatectomy specimens under the microscope [4]. It is a two or three-component numerical grading that is based on observed structural patterns, and can be expected to provide indication of tissue abnormality and tumor’s estimated likeliness to spread (metastatic potential). Gleason score can be estimated using speciments acquired by using transrectal ultrasound (TRUS) guided prostate biopsy. Unfortunately, in 30–50% of patients the findings based on systematic TRUS do not represent true Gleason score [5–7].

Magnetic resonance imaging (MRI) is increasingly being used for the detection of PCa lesions. Diffusion weighted MR imaging (DWI) has been shown to have potential for the detection and characterization of PCa. DWI data sets are still usually being analyzed by measuring only the first-order statistical properties found in parametric maps such as apparent diffusion coefficient of the monoexponential function (ADC_m_) [8–10].

Various different fitting methods have been applied for modeling PCa DWI signal decay. The biexponential function [11] provides the best fitting quality for DWI data sets acquired using “high” b values (∼2000 s/mm^2^) [12]. However, it is not robust to noise and has low repeatability. In contrast, the kurtosis function [13] provides similar fitting quality while being substantially more robust to noise [12]. Texture features of parametric maps derived from the kurtosis function have not been evaluated and hold promise by better signal characterization compared with the most commonly used monoexponential function [9]. Other MRI methods, such as T_2_-mapping (T_2_) and anatomical T_2_-weighted imaging (T_2_w), could provide complimentary information to DWI for prediction of PCa characteristics [10].

Computer-aided diagnostics (CAD) based on MRI has been demonstrated to have complementary role to a reporting radiologist in PCa detection [14–16]. However, only a limited number of studies focused on characterizing the detected PCa lesions, and they typically utilize only a small number of texture features. Tiwari et al. [17] used MR spectroscopy data and various texture features from T_2_w to first detect PCa and then predict its Gleason score. Peng et al. [18] evaluated histogram-based features from multiparametric MRI regarding correlation with Gleason score. Texture features from DWI and T_2_w have been assessed for differentiating Gleason scores [19–21]. Rozenberg et al. [22] evaluated whole-lesion histogram and texture features from DWI in order to predict Gleason score upgrade after radical prostatectomy.

The aim of this study was to use carefully optimized high quality MRI data sets to develop and validate machine learning methods for non-invasive Gleason score prediction, meaning prediction of PCa aggressiveness. In this study, we built and evaluated a classifier system based on multiple texture features of high quality T_2_w, DWI (the monoexponential and kurtosis functions), and T_2_ relaxation maps for prediction of PCa Gleason score dichotomized as 3+3 (low risk) vs >3+3 (high risk). Moreover, we explored which combinations of imaging modalities and texture extraction methods are most useful for this task.

The current study is first of its kind in multiple aspects: a) direct comparison of textures extracted from T_2_w and T_2_ relaxation maps, b) elevation of texture features from non-monoexponetial DWI signal decay using high quality data sets, and c) evaluation of a large number of texture extraction methods and parameters to calculate them in a multi-dimensional high quality MRI data sets of patients with PCa.

All data sets, post-processing program code as well as all MR sequences are freely available for review upon request following publication of the manuscript. Supporting Material is available at http://mrc.utu.fi/data. Program code used for calculations is available at https://github.com/jupito/dwilib.

## 2 Methods

The Ethics Committee of the Hospital District of Southwest Finland has approved this study. All patients have given written informed consent. The MR examinations were performed between March 2013 and May 2014. The study enrolled 72 consecutive patients with histologically confirmed PCa who were scheduled for robotic assisted laparoscopic prostatectomy.

Two of the patients had the Gonadotropin releasing hormone antagonist (Degarelix, Ferring Pharmaceuticals) started just before the MR examination. The rest of the patients had no prostate-related hormonal, surgical, or radiotherapy treatment before or during imaging.

A subset of the data has already been used in previous studies. DWI data sets (12 b values, 0–2000 s/mm^2^) of 48 patients were used for evaluating mathematical models of DWI [9, 10, 12], while T_2_ of 37 patients were used in feasibility evaluation of relaxation along fictitious field and continuous wave T_1*ρ*_ imaging of PCa [10, 23].

### 2.1 MRI examination

The MR examinations, as previously described [9, 10, 12], were performed using a 3T MR scanner (Ingenuity PET/MR, Philips, Cleveland, USA), a two channel volume whole body RF coil for excitation, and a 32 channel manufacture’s cardiac coils for measuring the signal.

Transversal single shot turbo spin echo (TSE) T_2_-weighted images (T_2_w) were acquired with repetition time/echo time (TR/TE) 4668/130 ms, field of view (FOV) 250×250 mm^2^, matrix size 250×320, slice thickness 2.5 mm, no intersection gap, and SENSE [24] factor 2. The acquisition time was 1 min 10 s.

For acquiring the DWI data sets, a single shot spin-echo based sequence was used with monopolar diffusion gradient scheme and echo-planar read out. Other parameters were TR/TE 3141/51 ms, FOV 250×250 mm^2^, acquisition matrix 100×99, reconstruction matrix 224×224, slice thickness 5.0 mm, number of slices 20, intersection gap 0.5 mm, diffusion gradients applied in three directions, diffusion gradient timing (Δ) 24.5 ms, diffusion gradient duration (*δ*) 12.6 ms, diffusion time (Δ − *δ*/3) 20.3 ms, SENSE [24] factor 2, partial-Fourier acquisition 0.69, SPAIR fat suppression, and b values (number of signal averages) 0 (2), 100 (2), 300 (2), 500 (2), 700 (2), 900 (2), 1100 (2), 1300 (2), 1500 (2), 1700 (3), 1900 (4), 2000 (4) s/mm^2^. The acquisition time was 8 min 48 s.

T_2_ relaxation values (T_2_ mapping) were obtained using a gradient and spin echo (GraSE) sequence with TR/TEs of 686/20, 40, 60, 80, 100 ms, FOV 230×183 mm^2^, acquisition matrix 256×163, reconstruction matrix 512×400, slice thickness 5.0 mm, and no intersection gap. The acquisition time was 1 min 35 s.

### 2.2 Histopathological analysis and cancer delineation on MRI

The whole mount prostatectomy sections were processed as previously described [12, 23]. The hematoxylin-eosin stained histological slides were first reviewed by one staff board certified pathologist and later re-reviewed by one experienced genitourinary pathologist (PT). In the cases there were differences between the two reviews, the opinion of the third genitourinary pathologist was searched and consensus was reached between the involved genitourinary pathologists.

The histology slice thickness of whole mount prostatectomy sections was approximately 4 mm (range 4–6 mm). Gleason scores were assigned to tumors as combinations of primary, secondary, and tertiary Gleason grade, as defined by the 2005 International Society of Urological Pathology Modified Gleason Grading System [4]. If a Gleason grade pattern higher than the primary and secondary grade was present and visually accounted for less than 5% of the tumor volume, it was assigned as tertiary Gleason grade [25].

Prostate cancer extent on each MRI acquisition (T_2_w, DWI, T_2_) was manually delineated by one research fellow (IJ) working in consensus with the genitourinary pathologist (PT), using whole mount prostatectomy sections as “ground truth.” Anatomical landmarks were used to align each MRI acquisition (T_2_w, DWI, T_2_) with mount prostatectomy sections.

### 2.3 Final data set

Ten of the patients were excluded from further analysis due to presence of motion (n = 2), severe susceptibility artifacts (n = 5), or incomplete data (n = 3). The characteristics of the remaining 62 patients are shown in Table A in S1 File. Their median age was 65 years (range 45–73 years), while the median serum PSA value was 9.3 ng/ml (range 1.3–30.0 ng/ml). The number of patients having one, two, and three lesions was 29, 28, and 5, respectively.

The final data set was composed of 100 PCa lesions derived from the MRI data sets of these 62 patients. In total, 67 and 33 lesions were located in peripheral zone (PZ) and central gland (CG), respectively. For the purpose of classifier performance evaluation, the Gleason scores of prostate cancer lesions were divided into two groups of low (3+3) and high (>3+3), containing 20 and 80 lesions, respectively.

### 2.4 MRI data post-processing

**Fig 1:**
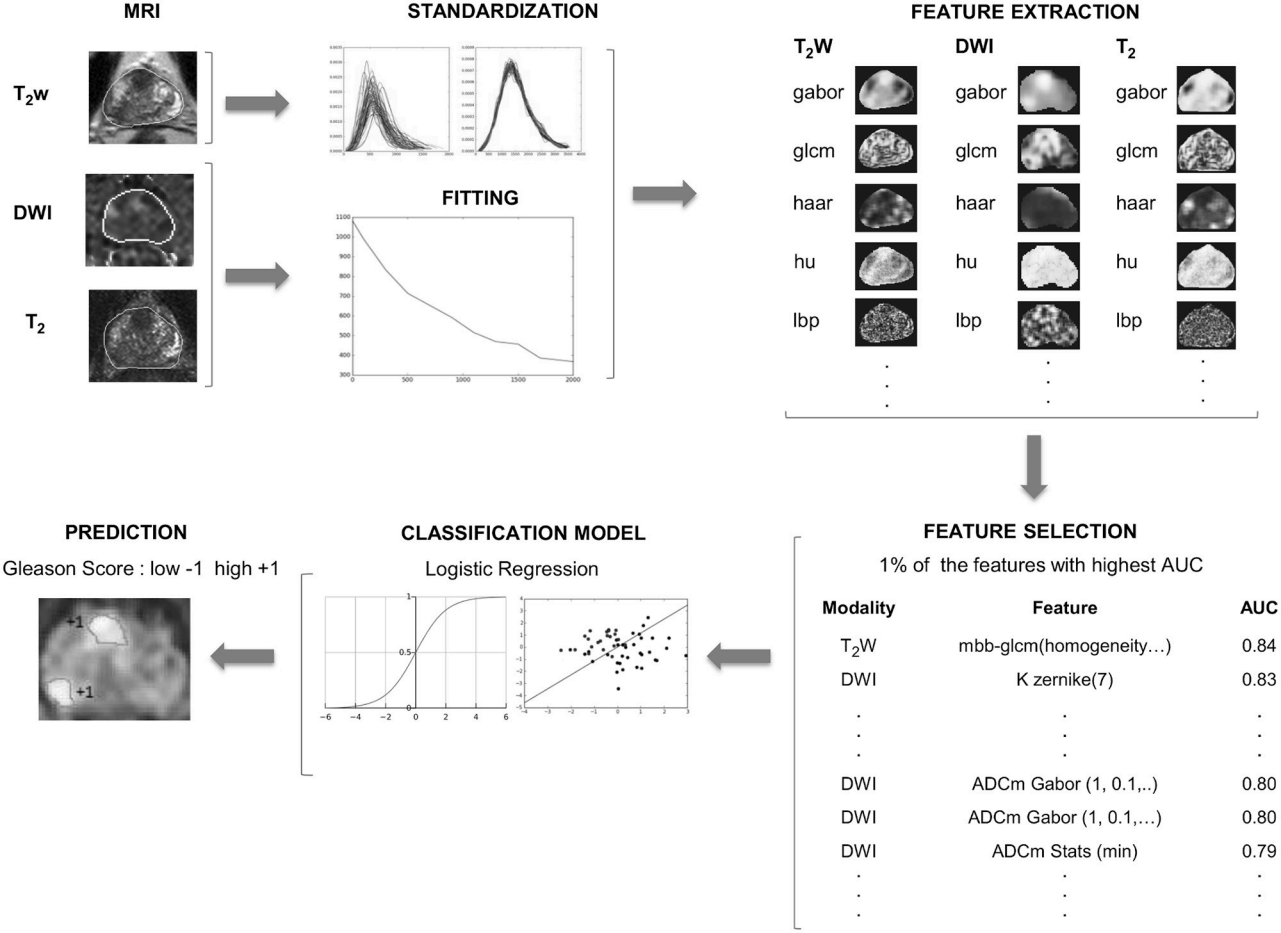
The pipeline. The T_2_-weighted images (T_2_w) are standardized, the monoexponential and kurtosis models are fitted to the diffusion weighted images (DWI), and the T_2_ relaxation values are obtained using a two parameter monoexponential function. Texture features are extracted subsequently. Top 1% of the features are selected by AUC. A logistic regression model is fitted to the selected features, and is used to predict the lesion’s Gleason score class.

**Fig 2:**
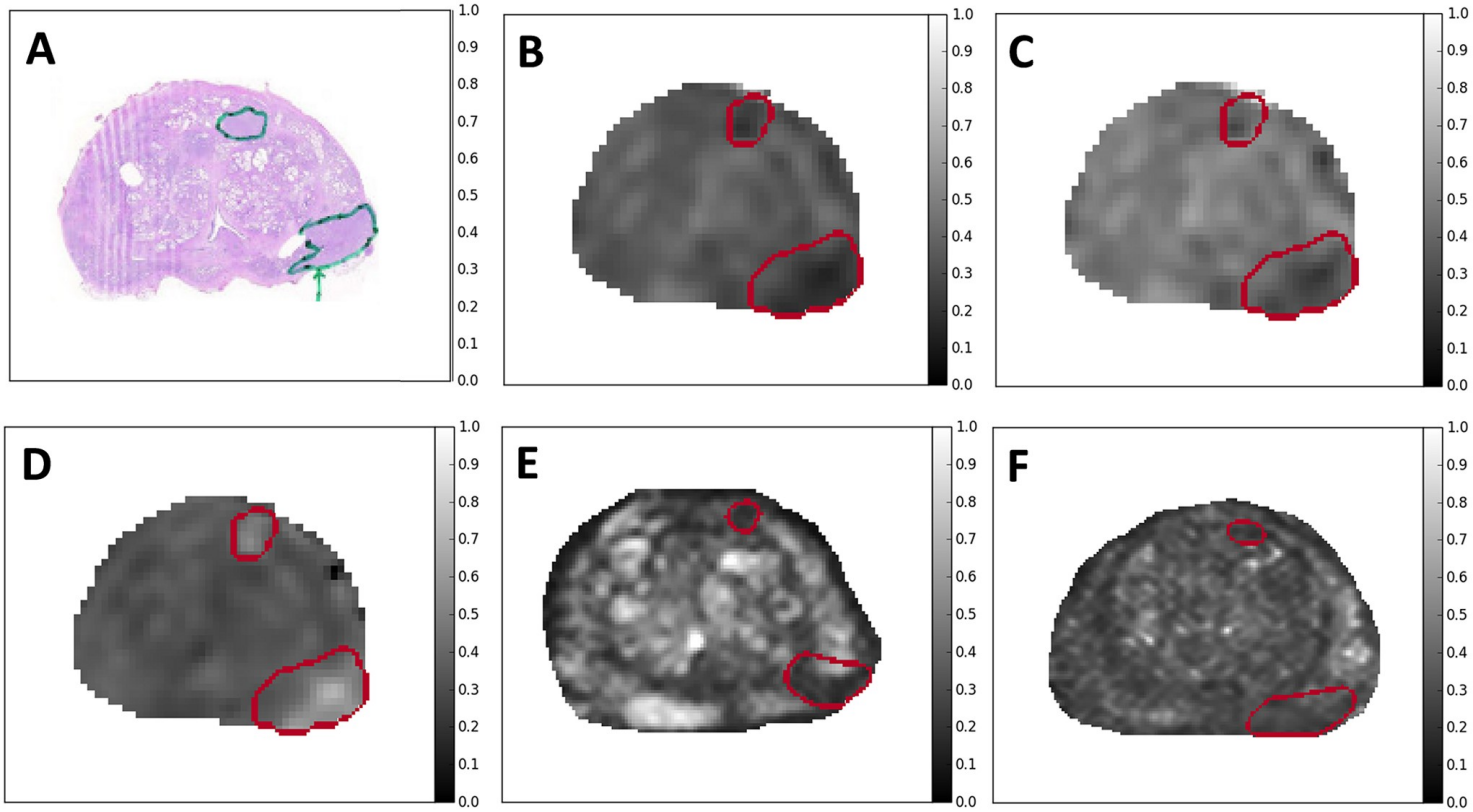
An example case with parametric maps. A: Whole mount prostate histological section. B: ADC_m_ (apparent diffusion coefficient, monoexponential model). C: ADC_m_ (apparent diffusion coefficient, kurtosis model). D: K (kurtosis parameter, kurtosis model). E: T_2_w (T_2_-weighted imaging). F: T_2_ (T_2_-mapping). This is from patient #43 (see Table A in S1 File). The two lesions are outlined; their Gleason scores are 4+3 (lower, posterolateral region) and 3+4 (upper, anterior region).

The post-processing pipeline is outlined in Fig 1. An example case with resulting standardized image and fitted parametric maps is shown in Fig 2. The in-house software used in fitting was quality controlled for correctness with cross-comparison to independent implementation and with visual inspections of parametric maps and distributions.

#### 2.4.1 T_2_w standardization

The signal intensity of a T_2_w image is not a specific tissue property and this non-standardness of T_2_w images (“intensity drift”) requires standardization to a common scale. To correct this bias, a histogram alignment method was used, as described by Nyúl et al. [26, 27]. This simple method transforms the images to make their histograms match at certain landmark locations, and interpolates the values between.

In this study, the deciles (i.e. every tenth percentile) were used as landmarks, as suggested by Nyúl et al. [26]. Only delineated prostate volume was considered for histogram averages, as other parts of the image might distort the learning. The result was validated by visual inspection.

#### 2.4.2 DWI fitting

Diffusion weighted imaging data sets were fitted on voxel level using the monoexponential model:

$$S(b)=S_{0}\;\text{exp}(-b{\text{ADC}}_{\text{m}})$$ (1)

and the kurtosis model [13]:

$$S(b)=S_{0}\;\text{exp}\left(-b{\text{ADC}}_{\text{k}}+\frac{1}{6}b^{2}{\text{ADC}}_{\text{k}}{}^{2}\text{K}\right)$$ (2)

where *S*(*b*) is the signal intensity as a function of *b* value, *S*_0_ is the signal intensity at *b* = 0 s/mm^2^, ADC_m_ is the apparent diffusion coefficient of the monoexponential model, ADC_k_ is the apparent diffusion coefficient of the kurtosis model, and K is the kurtosis.

The fitting procedure was performed using the Broyden-Fletcher-Goldfarb-Shanno (BFGS) algorithm [28] implemented by the dlib [29] library. In order to find a reliable fit and prevent local minima, the algorithm was executed with multiple evenly spaced initialization values. Their intervals (step sizes) were for ADC_m_ 0.1–3.0 μm^2^/ms (0.01 μm^2^/ms), for ADC_k_ 0.01–3.0 μm^2^/ms (0.1 μm^2^/ms), and for K 0.0001–4.0 (0.2).

#### 2.4.3 T_2_ fitting

T_2_ relaxation values were calculated on a single voxel level using a two parameter monoexponential function:

$$S(\text{TE})=S_{0}\;\text{exp}(-\text{TE}/{\text{T}}_{\text{2}})$$ (3)

where *S*(TE) is the signal intensity at given time TE, *S*_0_ is the signal intensity at TE = 0 ms, and T_2_ is the spin-spin relaxation time.

The Levenberg-Marquardt algorithm was used for fitting, as implemented by the SciPy library, with the multiple initialization values of 0.0 ms to 300 ms with step size of 50 ms. T_2_ relaxation values were constrained to 1–300 ms interval.

### 2.5 Feature extraction

Texture extraction methods can be roughly categorized into four main groups [30], although this taxonomy is somewhat ambiguous. The statistical approach is based on local spatial distributions and relationships of intensity occurrences in the image. The structural methods use well-defined geometrical primitives to measure texture. The model-based methods attempt to represent the image properties as parameters of various mathematical models. The transform methods use signal processing transformations such as Fourier and wavelets to analyze the image in a different space.

In this study, the gray-level co-occurrence matrix, the local binary patterns, and the histogram of oriented gradients can be assigned into the statistical category, and the Sobel operator into the structural one. The Hu and Zernike moments belong to the model-based group, while the Gabor filter and the Haar wavelet are transform methods.

The selection of texture extraction methods used in this study was based mainly on prior experiences in existing MRI literature [30, 31] and the availability of applicable open-source software components. All of these texture descriptor methods, except the Hu and Zernike moments, have been previously used for CAD of PCa. However, detailed information on their implementation and parameter selection are usually very scarce. This issue was addressed in this study by providing complete information on the parameters, and by using a wide array of them.

Three-dimensional texture extraction of MRI has been utilized in various studies, including some of PCa diagnosis [32]. In this study, however, texture analysis was performed only in 2D and per-slice, due to voxel anisotropy.

#### 2.5.1 General implementation details

Several texture descriptor methods with various parameter combinations were used for extracting 2D texture features from the manually delineated PCa lesions. Most of the methods in this study inherently incorporate the so-called “sliding window” algorithm. This means that the local voxel neighborhood for calculations is represented as a fixed-shape subwindow centered at each voxel in turn. The output image, a texture feature map, consists of the feature values positioned on corresponding neighborhood center locations. A more detailed explanation is provided by Clausi et al. [33], for example.

Seven window configurations were used for DWI and nine for T_2_w and T_2_ data. These square-shaped windows had evenly spaced voxel side lengths of 3, 5, …, 15 for DWI and 3, 7, …, 35 for the higher-resolution T_2_w and T_2_. These lengths correspond to 3.3–17 mm for DWI and 1.4–16 mm for T_2_w and T_2_, maintaining similar physical cover over different resolutions.

For each lesion, the window was placed on all possible locations along the transverse planes so that it still stayed completely within the lesion area. In cases of the window not fitting completely inside lesion area, the window locations with maximum lesion area were used. Extracted texture feature maps were then averaged over all slices to be used as lesion-wise median features. The use of different window sizes and parameter combinations resulted in 1281 features per DWI image type (ADC_m_, ADC_k_, K) and 1631 features per other image type (T_2_w, T_2_), totaling 7105 features all five image types combined. The free software libraries Scikit-image [34] and Mahotas [35] were utilized in the implementation of the process.

#### 2.5.2 Method-specific implementation details

The gray-level co-occurrence matrix (GLCM) [36] is a very popular method of texture characterization. It observes all the pixel gray level pairings that occur in the image at a certain distance and direction. In this study, the GLCM was calculated for each window using four different voxel distances of 1 to 4 (unless prevented by window dimensions). Because the 22 GLCM-derived features introduced by Haralick et al. [36] have correlation [37], using only three to five features have been recommended [37–39] in order to minimize redundancy and dimensionality.

In this study, the six GLCM features implemented by the Scikit-image software library [34] were extracted. These features, namely contrast, dissimilarity, homogeneity, energy, correlation, and angular second moment (i.e. uniformity), are among the ones most commonly used in previous studies [33]. They are generally considered effective texture discriminators, and maintain invariance regarding scale and shift [38]. In order to gain orientation invariance, the results were averaged over four bidirectional axes, and mean range over the orientations was added [36].

Since GLCM requires a discrete source image, the images needed prior normalization depending on source type. All images were quantized by uniform scaling to 32 gray levels, based on previous studies [33, 37, 38]. The image type specific source intensity ranges for scaling were manually defined by observing prostate volume histograms. In total 324–420 GLCM related features with sliding window approach were extracted per image type (T_2_w, ADC_m_, ADC_k_, K, T_2_).

In addition to the sliding window approach, the same GLCM-based features were also extracted using the minimum bounding box (MBB) around the whole lesion area of each image slice as the window, while ignoring any non-lesion voxels. This procedure is similar to the method used in a study by Vignati et al. [20]. The MBB-GLCM features were averaged over slices, and 48 features in total were extracted per image type.

Local binary patterns (LBP) [40] is a method that compares every voxel intensity value to a certain number of neighboring values on a circle around it. Each comparison result is stored as a single bit that tells whether the neighbor is larger or smaller than the center. The bit patterns are collected from the neighborhood and encoded as numbers, and the resulting histogram can then be used as a feature vector invariant to gray scale. In this study, the LBP were calculated within each window, observing eight interpolated neighboring points at the maximum radius allowed by window size. The different orientations of the uniform patterns were combined into rotation-invariant groups, and all non-uniform patterns were treated as a single pattern. Pattern frequency histograms were then used as features resulting in 70–90 different features per image type.

The Gabor function [41] is a Gaussian modulated by a sinusoidal wave. Gabor filter banks can be used for texture characterization, as each filter, shaped by its parameters, responds to specific local spatial frequency properties of the image [42]. Gabor filters are sensitive to edges in the image, so given that different lesions contain different regions, the detected edges between the regions could yield different responses. In this study, the texture extraction scheme described by Tüceryan et al. [43] was applied, which uses the sliding window on the Gabor-filtered complex images.

Based on experimentation and data visualization, the filter bank included the combinations of five different frequencies for the sinusoidal (*f* = 0.1, 0.2, *x*…, 0.5 per voxel), three sizes for a circular Gaussian envelope (*σ* = 1, 2, 3), and four bidirectional orientations. A number of derived features have been suggested [44–46]. Here, the extracted features included mean of the real part, variance of the real part, mean of the absolute of the real part, and mean of the magnitude. Various ways to achieve orientation invariance have been proposed [47–50], although not all are equally suitable for texture classification. In this study, the simple method of summing filtered images over orientations was used [48], yielding 420–540 features per image type.

The Haar transform is a simple wavelet decomposition of the image. Providing local spatial frequency information, it is useful as a tool of texture analysis [51]. In this study, a four-level Haar transform was first done for each image slice, and the three higher frequency coefficient planes were used, upscaled to the original size. For each sliding window, the mean absolute value and the standard deviation were then extracted as features [51], resulting in 168–216 features per image type.

Image moments are weighted averages that describe the distribution of intensity within image. A few variations are widely used as object shape descriptors, but they have also been applied to texture analysis [52]. In this study, logarithms of the absolute values of the seven Hu moments [53], and the magnitude values of the complex Zernike moments [54] up to the 8th degree were calculated for each window. The Hu moments are invariant regarding translation, scaling, rotation, and reflection [55]. The Zernike moment magnitudes are rotation invariant, robust to noise, and, due to their orthogonality, have minimal redundancy [56, 57]. In total, 49–63 Hu moments and 175–225 Zernike moments were extracted from each image type.

The histogram of oriented gradients [58] is an algorithm developed primarily for object recognition. It describes an object as a set of local gradient direction distributions. In this study, it was applied for texture analysis, using a single cell with eight directions for each window. The average over windows was then used as a feature, resulting in one feature per window size and 7–9 features in total per image type.

The Sobel operator [59] is a simple convolution filter that emphasizes edges in image. The shape of the kernel window is 3×3 by definition, so no other window sizes were used. Instead, the median of the whole lesion-wide texture map was used, both with the lesion edge voxels included and excluded, resulting in 2 features.

In addition, first-order statistical features were calculated over the whole lesion. First-order statistics observe only the probabilistic distribution of intensity values, ignoring their spatial relations. Existing literature typically utilizes averages and some of the percentiles [60]. Here, 18 features were included, namely mean, standard deviation, range, minimum, maximum, quartiles, deciles, kurtosis, and skewness.

### 2.6 Classification

For image types ADC_m_, ADC_k_, and K, a corresponding data set of 100 data points and 1281 features were used to build models for predicting prostate lesion aggressiveness based on Gleason score, while for T_2_w and T_2_ the number of features was 1631. The features were normalized to zero mean and unit variance. The data points were divided into two groups by Gleason score, low and high (3+3 and >3+3, respectively).

Logistic regression with either L1 or L2 regularization [61] implemented by Python Scikit-learn library [62] were used to train the low vs high Gleason score classifiers. Both regularization mechanisms compensate the high dimensionality of the data by penalizing large coefficient values of the inferred linear models, which in turn makes them less likely to overfit to the training data and more able to generalize to data unseen in the training phase.

L1 regularization has the additional property of shrinking the coefficients of the least useful features down to zero, and hence it also performs feature selection [63]. The number of coefficients ending down to zero depends on the amount of regularization. However, regularizing too strongly might lose valuable features and lead to underfitting. Therefore, it was also tested whether the simultaneous use of the classical filtering based feature selection approach would improve the prediction performance.

The predictive performance of the models built by the regularized logistic regression algorithms was estimated by a nested cross validation strategy [64], which consisted of an outer leave-pair-out cross-validation (LPOCV) [65] and an inner 10-fold cross validation (10FCV) for hyperparameter selection. In LPOCV every possible pair of data points were held out at a time as test set, while the remaining data formed the training set used to build the model for predicting on the held out pair. Both the filter based feature selection and the hyperparameter selection were performed for each round on LPOCV using the training set.

For selecting the best features, their performance was estimated using the receiver operating characteristic (ROC) curve, summarized as the area under the ROC curve (AUC). Ranked by AUC, the highest-performing 1% of all features (including statistical ones) were used to train the classifier.

After selecting the features the training set was transformed accordingly and the optimum regularization hyperparameter value was selected from *ω* = {0.001, 0.01, 0.1, 1, 10}, as measured by the AUC in stratified 10FCV. A classifier was then trained with the selected features and the regularization hyperparameter, and used for performing predictions on the two data points held out during the LPOCV round.

Afterwards, each data point was assigned an LPOCV score according to the ordering-by-the-number-of-wins method, in which the score of a data point is the number of times it obtains a larger predicted value than the other point during the LPOCV rounds when it is one of the two held out points. These LPOCV scores were then used to perform the ROC curve analysis, whose validity was previously demonstrated by Balcan et al. [66]. More precisely, using the LPOCV scores of the data points and their corresponding true label, the AUC and 95% confidence interval (CI) were calculated using the R package by LeDell et al. [67].

The feature selection and hyperparameter selection process as part of the LPOCV is illustrated in Pseudo code 1. The algorithm starts with a loop referred as LPOCV. During this loop the indices associated with the test pair in turn, (*i*, *j*) with *i* ≠ *j*, are not included in the index set *C* of training data. Every feature AUC is calculated using data from matrix **X** and label vector **y**. The notation **X**[*C*, *k*] refers to the submatrix of **X** containing the rows indexed by *C* and the columns by *k*, that is, the vector with the values for *k*th feature in the training data. The top 1% independent features are indexed by *B*. The optimum regularization hyperparameter value *α* ∈ *ω* is calculated using 10FCV on data (**y**[*C*], **X**[*C*, *B*]), which is then used to train a model *f* to make predictions on the test pair (**X**[*i*, *B*], **X**[*j*, *B*]). The last line of the pseudo code describes the calculation of LPOCV score using ordering-by-the-number-of-wins method.

**Pseudo code 1 LPOCV with inner feature selection by AUC filtering and hyperparameter selection**.

**Input**: **X**, matrix of *n* lesions × *F* features

**Input**: **y**, vector of labels (1 high, -1 low)

**Input**: *ω* = {0.001, 0.01, 0.1, 1, 10}, set of hyperparameters

**Output**: LPOCV scores

 **for** *i*, *j* ∈ {1, 2, …, *n*} **do**  *{All possible lesion pairs}*

  *C* ← {1, 2, …, *n*}\{*i*, *j*}    *{All lesions except i, j}*

  **auc** ← vector of length *F*

  **for** *k* ∈ {1, 2, …, *F*} **do**

   **auc**[*k*] ← *AUC*(**X**[*C*, *k*], **y**[*C*])  *{Calculate AUC for each feature}*

   **auc**[*k*] ← max(**auc**[*k*], 1 − **auc**[*k*])   *{Handle inverse correlation}*

  **end for**

  *B* ← arg sort(**auc**)[1…(0.01 × *F*)]  *{Get indices of the best features}*

  *α* ← gridSearch(**y**[*C*], **X**[*C*, *B*], *ω*)   *{Grid search with 10FCV, returns the best hyperparameter}*

  *f* ← *A*(**y**[*C*], **X**[*C*, *B*], *α*)  *{Train model f with algorithm A and hyperparameter α}*

  **W**_*ij*_ ← *H*(*f*(**X**[*i*, *B*]) − *f*(**X**[*j*, *B*]))   *{Matrix W stores results from Heaviside function (H) of i and j prediction difference}*

 **end for**

 $\tilde{y}\leftarrow W1$  *{Score of each element obtained by summing along axis j}*

 **return** $\tilde{y}$   *{Returns LPOCV scores}*

### 2.7 Addressing bias and imbalance

It is important to note that the nested cross-validation scheme allows feature selection and hyperparameter tuning while avoiding bias in the performance estimate [64]. In each round of LPOCV, the pair of data points left for testing does not affect the feature selection nor the hyperparameter tuning of the predictive model in turn.

The ratio between low and high Gleason score is 1:4 in the data set, so there is some degree of imbalance between classes. However, the model performance was estimated using LPOCV together with AUC, and that degree of imbalance in the classes has low effect on these methods [65, 68]. LPOCV is an unbiased estimate of the prediction performance of a model [65], and the ROC AUC is not affected by imbalance as it measures how accurately a model ranks a random positive unit from a negative one [69].

## 3 Results

The highest ranking features for differentiating Gleason scores are summarized by texture extraction method in Table 1. Features based on Gabor filters were included in all image types. GLCM features were selected for T_2_w and T_2_, and Zernike moments for K and T_2_. Features from the Hu moments and LBP were also selected for T_2_, in which the top 1% had more variability than other image types, regarding both the texture extraction method and window size.

**Table 1:** Texture methods ranked in the best one percent.

Image type	Window sizes	Texture extraction methods	AUC range
T_2_w	27	MBB-GLCM, GLCM, Gabor	0.71–0.84
ADC_m_	11	Gabor	0.79–0.80
ADC_k_	11	Gabor	0.79–0.80
K	7, 9	Zernike, Gabor	0.78–0.83
T_2_	15, 19, 27, 31, 35	Zernike, Hu, MBB-GLCM, LBP, GLCM, Gabor	0.71–0.75

### 3.1 Univariate analysis

ROC analysis was performed for each texture feature. The resulting best features are shown in Table 2. The best one was MBB-GLCM homogeneity in T_2_w with AUC = 0.84.

**Table 2:** Best texture feature per image type.

Image type	Window sizes	Type of Texture feature	AUC
T_2_w	NA	MBB-GLCM: homogeneity, *d* = 3, range	0.84
ADC_m_	11	Gabor: *σ* = 1, *f* = 0.3, mean	0.80
ADC_k_	11	Gabor: *σ* = 2, *f* = 0.1, mean	0.80
K	7	Zernike: index = 7	0.83
T_2_	35	Zernike: index = 3	0.75

A similar analysis was performed for the first-order statistical features; results are shown in Table 3. Although the best statistical features had good performance for most of the modalities, they did not out-perform the best texture features.

**Table 3:** Best statistical feature per image type.

Image type	Statistical feature	AUC
T_2_w	Minimum	0.72
ADC_m_	Minimum	0.79
ADC_k_	Minimum	0.79
K	Range	0.78
T_2_	20th percentile	0.55

**Fig 3:**
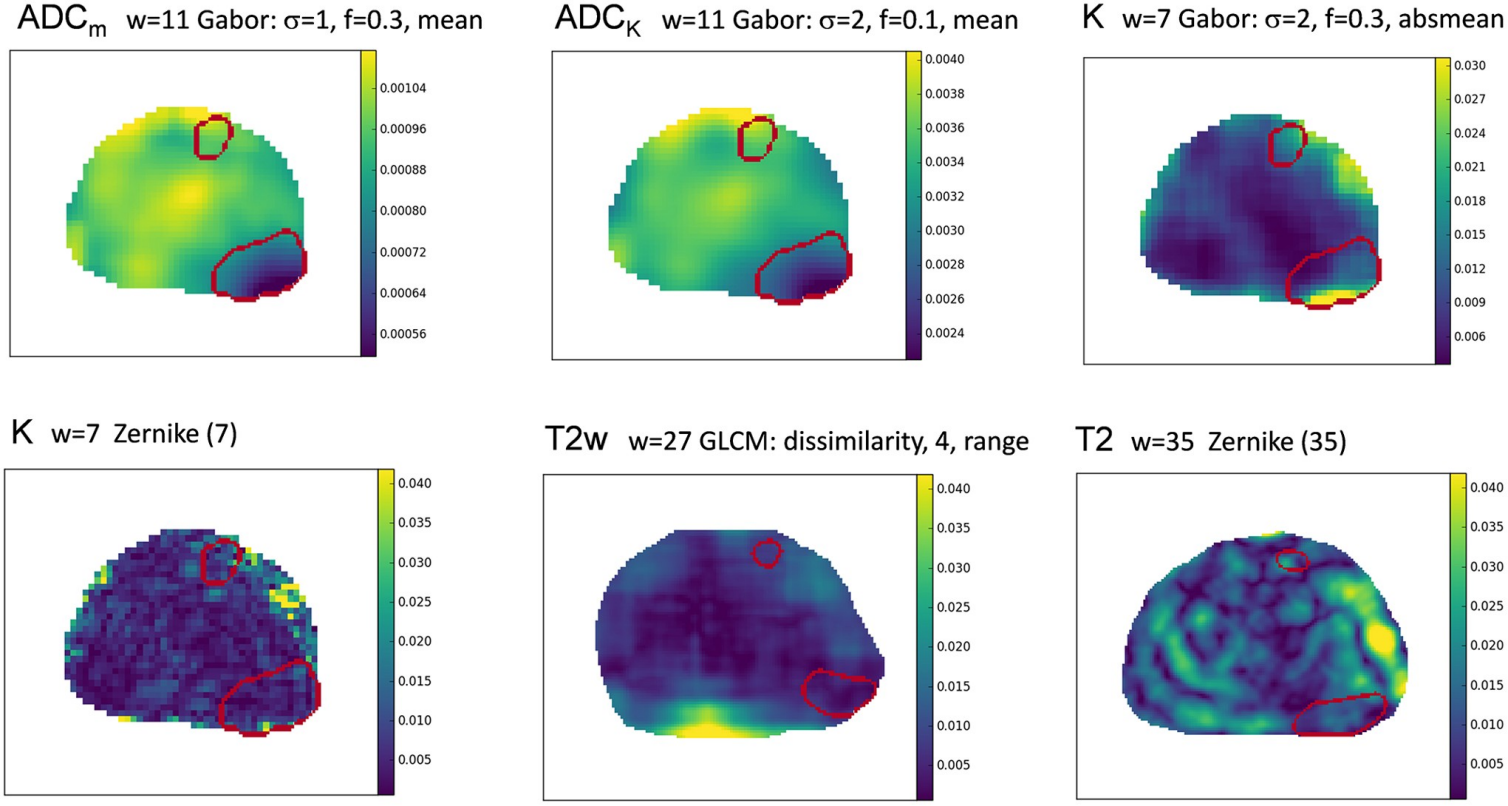
An example of texture feature maps. These are extracted from DWI parametric maps (ADC_m_, ADC_k_, K), T_2_-weighted imaging (T_2_w), and parametric map of T_2_ relaxation values (T_2_). Source image type, window size, and texture descriptor parameters are shown above the images. The two lesions are outlined; their Gleason scores are 4+3 (lower) and 3+4 (upper).

**Fig 4:**
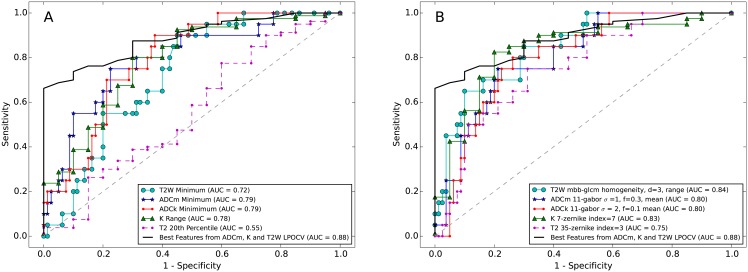
ROC curves within each image type (T_2_w, ADC_m_, ADC_k_, K, T_2_). A: The best statistical feature. B: The best texture feature. The final model of the best selected features from ADC_m_, K, and T_2_w obtained using L1 regularized logistic regression and validated with leave-pair-out cross-validation (LPOCV) is also included in both A and B.

Some of the high-ranking features are visualized in Fig 3. ROC curves for best statistical and texture features are presented in Fig 4.

Some of the top features were highly correlated between different image types. See Table M in S1 File for Spearman rank correlation coefficients for the features listed in Tables 2 and 3, calculated across image types. In addition, Table N in S1 File contains similar correlation metrics for all feature pairs, where top five features were taken from each image type.

### 3.2 Multivariate analysis

The prediction performance of the models trained using regularized logistic regression was estimated by LPOCV. Both L1 and L2 regularization methods were utilized separately. Table 4 contains the results for models within each image type using all of the features and top 1% of them. The results are presented as ROC AUC values along with 95% confidence intervals.

**Table 4:** Performance figures for each image type alone. They are ROC AUC (receiver operating characteristic, area under curve) values estimated using outer leave-pair-out cross-validation (LPOCV) and different feature subsets.

Image type	ML algorithm	All features		Top 1% features		18 statistical
		N	AUC (95% CI)	N	AUC (95% CI)	AUC (95% CI)
T_2_w	Log. Reg. L1	1631	0.82 (0.72–0.92)	16	0.80 (0.69–0.90)	0.67 (0.56–0.77)
	Log. Reg. L2		0.68 (0.55–0.82)		0.75 (0.64–0.87)	0.71 (0.60–0.81)
ADC_m_	Log. Reg. L1	1281	0.67 (0.55–0.79)	12	0.71 (0.60–0.82)	0.79 (0.68–0.90)
	Log. Reg. L2		0.69 (0.57–0.81)		0.75 (0.65–0.86)	0.75 (0.63–0.86)
ADC_k_	Log. Reg. L1	1281	0.71 (0.58–0.83)	12	0.74 (0.63–0.84)	0.78 (0.69–0.88)
	Log. Reg. L2		0.73 (0.63–0.83)		0.76 (0.65–0.86)	0.73 (0.61–0.84)
K	Log. Reg. L1	1281	0.64 (0.52–0.77)	12	0.78 (0.67–0.89)	0.75 (0.61–0.88)
	Log. Reg. L2		0.73 (0.60–0.85)		0.76 (0.64–0.87)	0.73 (0.60–0.86)
T_2_	Log. Reg. L1	1631	0.58 (0.45–0.71)	16	0.51 (0.37–0.65)	0.67 (0.55–0.79)
	Log. Reg. L2		0.70 (0.59–0.82)		0.56 (0.43–0.69)	0.56 (0.43–0.68)

Similar performance estimates of the models combining features from different image types are presented in Table 5.

**Table 5:** Performance figures for image type combinations. They are ROC AUC (receiver operating characteristic, area under curve) values estimated using outer leave-pair-out cross-validation (LPOCV) and different feature subsets.

Image types	ML algorithm	All features		Top 1% features	
		N	AUC (95% CI)	N	AUC (95% CI)
ADC_k_, K	Log. Reg. L1	2562	0.61 (0.49–0.74)	25	0.82 (0.72–0.92)
	Log. Reg. L2		0.72 (0.60–0.83)		0.81 (0.70–0.91)
ADC_m_, K	Log. Reg. L1	2562	0.57 (0.45–0.70)	25	0.81 (0.71–0.91)
	Log. Reg. L2		0.72 (0.60–0.84)		0.79 (0.68–0.89)
ADC_m_, ADC_k_, K	Log. Reg. L1	3843	0.58 (0.44–0.72)	38	0.83 (0.74–0.92)
	Log. Reg. L2		0.69 (0.56–0.81)		0.79 (0.70–0.88)
ADC_m_, ADC_k_, K, T_2_	Log. Reg. L1	5474	0.61 (0.47–0.74)	54	0.84 (0.75–0.92)
	Log. Reg. L2		0.77 (0.66–0.87)		0.79 (0.70–0.88)
ADC_m_, ADC_k_, K, T_2_w	Log. Reg. L1	5474	0.78 (0.68–0.89)	54	0.88 (0.81–0.95)
	Log. Reg. L2		0.70 (0.58–0.82)		0.86 (0.78–0.93)
ADC_m_, ADC_k_, K, T_2_, T_2_w	Log. Reg. L1	7105	0.74 (0.62–0.86)	71	0.88 (0.81–0.95)
	Log. Reg. L2		0.79 (0.69–0.90)		0.86 (0.78–0.93)
ADC_m_, K, T_2_	Log. Reg. L1	4193	0.69 (0.56–0.82)	41	0.83 (0.74–0.91)
	Log. Reg. L2		0.78 (0.68–0.88)		0.82 (0.73–0.90)
ADC_m_, K, T_2_w	Log. Reg. L1	4193	0.81 (0.71–0.91)	41	0.88 (0.82–0.95)
	Log. Reg. L2		0.70 (0.59–0.82)		0.86 (0.79–0.93)
ADC_m_, K, T_2_, T_2_w	Log. Reg. L1	5824	0.76 (0.65–0.87)	58	0.87 (0.81–0.94)
	Log. Reg. L2		0.79 (0.68–0.89)		0.85 (0.77–0.92)
ADC_k_, K, T_2_	Log. Reg. L1	4193	0.53 (0.40–0.66)	41	0.81 (0.72–0.91)
	Log. Reg. L2		0.80 (0.71–0.89)		0.81 (0.71–0.91)
ADC_k_, K, T_2_w	Log. Reg. L1	4193	0.81 (0.72–0.91)	41	0.85 (0.77–0.93)
	Log. Reg. L2		0.72 (0.61–0.84)		0.84 (0.76–0.92)
T_2_, T_2_w	Log. Reg. L1	3262	0.82 (0.73–0.91)	32	0.66 (0.52–0.79)
	Log. Reg. L2		0.78 (0.68–0.88)		0.61 (0.48–0.74)

#### 3.2.1 All features within individual image types

When using all features and L1 regularization (Table 4), T_2_w had AUC = 0.82, DWI derived parametric maps (ADC_m_, ADC_k_, K) had AUC range 0.64–0.71, and T_2_ derived features had AUC = 0.58.

In contrast to L1, L2 regularization yielded better performance for all image types except T_2_w where AUC dropped to 0.68. DWI-derived parametric maps (ADC_m_, ADC_k_, K) had AUC range 0.69–0.73, and T_2_ derived features had AUC = 0.70.

These results indicate that, when it comes to logistic regression models, all features weighted by L2 regularization may perform better than fewer features selected by L1, with the exception of T_2_w images. With T_2_w, L1 regularization performed better than L2, suggesting that a subset of features would perform better than all of them.

#### 3.2.2 Selected features within individual image types

The feature selection was based on filtering features by AUC. Only the best 1% of features (12 or 16 features, depending on image modality) with highest ranking AUC were selected in each image type. When using L1 regularization, the best T_2_w features showed better performance (AUC = 0.80) than the features of DWI-derived parametric maps (ADC_m_, ADC_k_, K), which had AUC range 0.71–0.78. The best T_2_ features had AUC = 0.51 which is the lowest performance among the modalities.

The estimated AUC values using L2 regularization and the best 1% features, compared with L1 regularization, were lower for T_2_w and K.

The texture features did not substantially out-perform the 18 statistical features the corresponding image type (Table 4), except in T_2_w where the texture feature model obtained with L1 regularization had the best performance among all other models. The highest AUC value based on statistical features was 0.79, achieved using L1 regularization and ADC_m_. The corresponding value for the best 1% texture features was 0.71.

The AUC values for the top-1% texture features based on T_2_w, ADC_m_, ADC_k_, K, and T_2_ are shown in Tables B, D, F, H, and J in S1 File, respectively. Similarly, AUCs for the statistical features for each image type are shown in Tables C, E, G, I, and K in S1 File.

#### 3.2.3 All features of combined image types

No substantial improvements of AUC values were present when combining all features of all image types (Table 5). The AUCs were within range 0.53–0.82 with L1 regularization and within 0.69–0.80 with L2.

#### 3.2.4 Selected features of combined image types

In contrast to the use all features, a better model performance was present when combining the best 1% features of all image types (T_2_w, ADC_m_, ADC_k_, K, T_2_). The best performing model with AUC = 0.88 was obtained when selecting the best 1% features based on ADC_k_, K and T_2_w. The combinations of features extracted from DWI parametric maps (ADC_m_, ADC_k_, K) and those extracted from T_2_w and T_2_ together with the feature selection method lead to improved prediction performance regardless of the regularization method (L1, L2).

The final model proposed to differentiate low from high Gleason score PCa includes the features from ADC_m_, K, and T_2_w listed in Table L in S1 File. The expected performance ROC is presented in Fig 4.

## 4 Discussion

There is an increasing number of research groups studying and developing CAD of prostate cancer. The topic was recently reviewed by Lemaître et al. [31]. Most of the publications have focused on the task of cancer detection rather than characterization. However, both are required for proper treatment decision planning. In this study, we built a classifier for prostate cancer characterization utilizing texture features extracted from T_2_w, the monoexponential and kurtosis models of high-b-value DWI, and T_2_ maps.

Several studies have demonstrated correlation between ADC_m_ values and Gleason score based on biopsy [8, 70] or prostatectomy specimens [9, 18, 71–73]. Most of the studies evaluating the performance of DWI for Gleason score prediction have used first-order statistical features, which do not consider the spatial relationships between voxels. Analyzing the texture may add useful information regarding tumor heterogeneity and other structural properties. In our previous studies we observed rectangular, fixed-shape regions-of-interest, and each tumor was characterized by only one variable per image type [9, 10, 23]. However, in this study we measured the texture properties of each of the image types (T_2_w, ADC_m_, ADC_k_, K, T_2_). We have shown that the characterization performance of prostate cancer can be improved by combining texture features from the monoexponential and kurtosis models, and the T_2_w.

Most studies on texture analysis of PCa include only a small number of texture descriptors and configurations [14–16]. In this study, we utilized a large number of both, from multiparametric source. This allows evaluating a huge number of feature combinations, as the regularization prevents overfitting caused by high dimensionality.

Texture analysis of multiparametric MRI has previously seen limited use in PCa characterization. Fehr et al. [21] evaluated PCa characterization with the whole-lesion first order statistics and GLCM texture features from a similar-size dataset of ADC and T_2_w. They used oversampling to ward off effect of class imbalance. Similarly to our study, they integrated dynamic feature selection as part of the training (using the recursive feature selection support vector machine, RFE-SVM). In our study, we included a much more diverse and numerous set of features, as one of our goals was to evaluate various texture extraction methods. Moreover, we have for the first time demonstrated that using texture features from K (kurtosis function) provided improvements to ADC_m_ (monoexponential function). This is important since first order statistics of parameters derived from kurtosis function do not lead to improved performance of ADC_m_ (monoexponential function). The effect of noise remains to be explored in future studies.

Tiwari et al. [17] classified PCa using GLCM and simple gradient features from T_2_w and MR spectroscopy (MRS). A multi-kernel classifier with graph embedding was used to reduce dimensionality. Compared to the current study, they had fewer patients, and the classification was done on equally-sized, rectangular metavoxels.

Furthermore, Wibmer et al. [19] studied the associations of Gleason scores and individual GLCM features from ADC and T_2_w of PZ lesions, using generalized linear regression and generalized estimating equations; and Vignati et al. [20] tested Gleason score differentiation using two of the GLCM features (contrast and homogeneity) from T_2_w and ADC individually.

Contrarily to previous approaches to performing non-rigid deformation and co-registration of datasets with subsequent resampling to common space and resolution [15, 16], in the current study the prostate and tumor masks were done for each MR imaging method (T_2_w, DWI, T_2_) individually, allowing us to perform texture analyses at their original native resolutions. Estimating the effect of co-registration and resampling on texture extraction is not trivial, and the process could cause loss of information. However, the accuracy of the delineations in this study could be potentially improved by an added step of co-registering MRI to histology images [74].

We highlight the limitation of performing re-slicing and non-rigid deformation of MR data sets to common space and then co-registering with whole mount prostatectomy sections. As noted by Bourne et al. [74], co-registration of whole mount prostatectomy sections to MRI data sets is important. However, the effect of re-slicing and non-rigid deformation of MR data sets to common space remains to be explored.

The gray-level co-occurrence matrix may well be the most widely used tool for texture analysis of prostate MRI data sets. The Sobel operator, Gabor filters, Haar transform, and local binary patterns have already been extensively applied for texture analysis of prostate MRI, as have a few others [31] not included in this study.

The image moments, on the other hand, have been used more often for global morphological analysis like shape recognition rather than local texture analysis, although they have been used for texture as well [52, 75, 76]. To the best of our knowledge, there are no published studies using moment-based texture analysis for detection or characterization of prostate cancer using MRI data sets. Tahmasbi et al. [56] used Zernike moments to characterize breast cancer, but as a global mass descriptor and not for texture. Our results suggest that moment-based texture features might be valuable for PCa characterization. More specifically, the best 1% features of the image types K and T_2_, and the final model ADC_m_, K, and T_2_w combined included some of the texture features based on Hu or Zernike moments.

The GLCM summarizes pixel intensity occurrences, the Gabor descriptors detect gradients of certain frequencies, and the LBP responds to point-like intensity transformation patterns. The image moments describe the mass distribution of the image content which is seen as a function that is integrated over space. Given the supposed difference in tissue heterogeneity, it makes sense that a metric based on mass distribution would discriminate lesions of varying Gleason scores.

Most of the texture extraction methods in this study use the sliding window algorithm with seven or nine different window sizes depending on image resolution. Usually, the window should be large enough to provide reliable statistical information about its contents to characterize the texture, yet small enough so that patches of different classes do not overlap too much [36, 38]. The nature of each texture extraction algorithm also affects the specific role and usefulness of each window configuration. The optimal window size depends on method and data, and typically cannot be estimated in practice without experimentation [77]. Most of the previous studies have utilized a very small number of different window sizes, often without presenting validation for the choice. In this study, we explored several window sizes simultaneously. This approach greatly increases the number of features, which is usually something to be avoided in order to produce an effective classifier. However, the machine learning method we used scales well to a large number of features.

In addition to texture features, shape descriptors might provide information useful for Gleason score characterization [78]. However, we decided to leave them out of this study and focus on texture features only. Including shape features would have required to treat lesions of different prostate regions differently, since lesions in peripheral zone might spread differently than lesions in central/transitional zone.

We have evaluated an extensive number of MRI texture features in multivariate setting for their ability to predict the Gleason score of prostate cancer. Moreover, we have presented a machine learning system that, from a very large number of candidate features, searches for a relevant subset for the task and alternatively weights the features accordingly.

The single feature with highest prediction performance estimate (AUC = 0.84) was a gray-level co-occurrence matrix homogeneity of T_2_w. The Gabor transform features performed well with the ADC and K parameters. The lowest percentile statistics were useful with ADC and T_2_w. The features based on Hu and Zernike moments performed well for K and T_2_. Our results imply that a specific set of features and feature extraction methods is needed to obtain maximal information from DWI, T_2_w, and T_2_.

The highest overall performance estimate (AUC = 0.88) was obtained for the model utilizing a small subset of texture features from the ADC_m_, K, and T_2_w parameters. These features included texture descriptors based on gray-level co-occurrence matrix, Gabor transform, and the Zernike and Hu moments.

Our study has several limitations. First of all, only 62 patients were included and further validation of our results in large patient cohort is needed. All of the patients had gone through prostatectomy, and therefore it is biased on the high Gleason score group with 80% of the lesions. As is the case with many previous studies, only one MRI data set per imaging method per patient was evaluated. Therefore, the repeatability of the texture features cannot be evaluated. Ideally, quantitative imaging methods would have high reliability and repeatability, allowing the use of derived features for disease characterization [79].

Many of the texture extraction methods used in this study could be further refined. Variations of the methods and the derived features have been proposed, for example for Gabor filters [44], and local binary patterns [80, 81]. For Gabor filters, schemes for unsupervised tuning of optimal parameters have been proposed [82]. The Zernike moments can be provided scale and transformation invariance [83].

In the cross-validation process the set of selected features was slightly different in every round, implying that some of the features may convey similar information. This is natural since we tested such a large number of feature candidates.

In this study, we focused on the characterization of histologically confirmed and manually delineated cancer lesions. In a more practical setting, this process should be preceded by automatic segmentation of the prostate and detection of cancerous tissue. This limitation should be addressed in future studies.

Studies show increased risk of PCa specific mortality for Gleason score 4+3 in comparison to 3+4 [84]. Differentiating these scores in the characterization process would be useful in addition to the 3+3 vs >3+3 threshold that was considered in this study.

Our results suggest that the use of texture features extracted from T_2_w, ADC_m_, and K parametric maps leads to improved PCa characterization accuracy compared to the more commonly used statistical features of DWI. In contrast, adding features from T_2_ did not improve the classification accuracy. The results point out certain features and feature combinations that were succesful, out of a very numerous set that includes various source image types, texture extraction methods, window sizes, and method-specific configurations. Most of the useful methods have already performed well in other studies (GLCM, Gabor, LBP). However, the image moment based texture features (Hu, Zernike) appear to be novel in the context of PCa characterization.

## Supporting information

S1 File Supporting tables. Patient characteristics; best features of each image type; features in final proposed model; best features’ correlation coefficients among image types.
(PDF)

S2 File Supporting figures. Files DWI-Mono-ADCm-xxx.png, T2-fitted-xxx.png, and T2w-std-xxx.png correspond to ADC_m_ and T_2_ parametric maps, and T_2_-weighted images of each patient, respectively. On the first row of slices they show positions of regions of interest placed on the prostate cancer lesions (red, yellow) and around whole prostate (white). The prostate mask is on the second row, while the remaining rows are lesion masks. Files histology-xx.jpg contain the whole mount prostatectomy sections of each patient, with tumor outlines in green. Please note that identical MRI acquisition protocol has been used on all patients, including slice thickness. Here all prostate cancer masks are show with corresponding whole mount prostatectomy sections.
(ZIP)

S3 File Lesion radiomics. This file contains the 7105 radiomics calculated for each of the 100 lesions from all 62 patients in CSV format (comma-separated values). The first column contains the patient ID number. The second column contains the Gleason score group used for classifying: –1 for low score group, 1 for high score group. The rest of the columns are the calculated feature averages over each lesion. The first row with column descriptions contains the feature names.
(ZIP)
