# Supplementary material for: Radiomics and machine learning of multisequence multiparametric prostate MRI: Towards improved non-invasive prostate cancer characterization
Source: PLoS One. 2019 Jul 8;14(7):e0217702. doi: 10.1371/journal.pone.0217702 (PMC6613688; doi:10.1371/journal.pone.0217702)
Supplement: S1 File — Patient characteristics; best features of each image type; features in final proposed model; best features’ correlation coefficients among image types. (PDF) [file pone.0217702.s001.pdf]

Radiomics and machine learning of multisequence multiparametric prostate MRI: towards improved non-invasive prostate cancer characterization

S1 File: Supporting Tables

Table A: Patient characteristics. PSA = prostate specific antigen; PZ = peripheral zone; CG = central gland.

Patient no.	Age (years)	PSA (ng/ml)	Gleason score	Location	Time (days)
1	60	9.2	3+3	PZ	5
2	67	11.0	3+4	PZ	11
3	66	9.3	4+3	PZ	16
4	66	9.3	3+3	CG/CG	20
5	63	6.0	3+3	PZ	27
6	67	13.0	3+4+5	CG	16
7	51	1.3	3+4	CG	21
8	68	6.2	3+4	PZ	32
9	73	13.0	3+3	PZ	32
10	68	30.0	5+4	PZ	29
11	66	15.0	4+5	PZ	35
12	67	12.0	3+4/3+4	PZ/PZ	5
13	68	3.9	3+4/3+4	PZ/PZ	3
14	60	28.0	4+3+5/3+4	PZ/PZ	18
15	69	27.0	4+5/4+3	PZ/CG	1
16	57	19.0	5+3/3+4	PZ/PZ	60
17	71	9.5	4+5/3+4	PZ/CG	45
18	62	7.7	3+4	PZ	11
19	58	19.0	4+5/3+4	PZ/CG	61
20	62	7.7	5+4/3+4	PZ/CG	10
21	70	10.0	4+4/3+4	PZ/CG	17
22	67	5.1	4+4	PZ	15
23	58	9.8	3+4, 3+3	CG/CG	11
24	60	3.9	3+3/3+3/3+3	PZ/CG/CG	10
25	66	18.0	4+5	CG	14
26	63	4.8	5+4/5+5	PZ/CG	4
27	68	9.5	3+4	CG	9
28	57	3.4	3+4	PZ	27
29	65	5.7	3+4, 3+4	PZ	4
30	65	9.9	3+4	PZ	5
31	59	30.0	5+4/3+3	PZ/CG	11
32	49	20.0	3+4	CG	18
33	62	14.0	3+3	CG	18
34	69	13.0	3+4/3+4	CG/CG	6
35	63	9.1	3+4	CG	2

36	64	5.2	3+3+4/3+3	CG/CG	7
37	70	12.0	3+4+5/3+4	PZ/PZ	13
38	62	4.1	4+5	PZ	23
39	67	4.6	4+5	PZ	11
40	67	8.3	4+3+5	PZ	18
41	66	6.6	4+3	PZ	18
42	64	8.8	3+4/3+4	PZ/PZ	9
43	71	18.0	4+3/3+4	PZ/CG	18
44	45	12.0	3+4	PZ	20
45	60	8.6	4+5	PZ	2
46	64	4.7	3+4	PZ	12
47	53	22.0	3+4+5	PZ	1
48	59	10.0	3+4/3+3/3+3	CG/PZ/PZ	0
49	65	3.5	3+4/3+3	PZ/PZ	3
50	61	11.0	3+3	PZ	1
51	72	5.4	3+4	CG	1
52	64	5.6	3+3	PZ	6
53	65	4.5	3+4/3+3	PZ/PZ	16
54	68	3.2	3+4/3+4	PZ/PZ	6
55	68	12.0	4+53+4	PZ/CG	14
56	63	8.2	4+3/3+4	PZ/CG	1
57	68	6.2	3+4/4+5/3+4	CG/PZ/PZ	7
58	59	24.0	4+3	PZ	13
59	64	16.0	4+3/3+4	PZ/CG	27
60	62	7.7	3+4/3+4	PZ/CG	2
61	73	13.0	3+4/3+4/3+3	PZ/CG/PZ	7
62	68	5.3	3+4/3+4/3+4	PZ/CG/PZ	21

Table B: T_2-weighted imaging: best 1% features.

Window size	Texture	AUC
3	zernike(20)	0.763
NA	stats(min)	0.726
27	gabor(3,0.1,var)	0.721
27	gabor(1,0.1,var)	0.715
27	gabor(2,0.1,var)	0.715
11	zernike(14)	0.712
27	gabor(1,0.2,var)	0.711
27	gabor(3,0.1,mag)	0.709
27	gabor(1,0.3,var)	0.708
27	gabor(2,0.2,absmean)	0.704
27	gabor(1,0.4,mag)	0.698
11	zernike(22)	0.692
27	gabor(1,0.3,mag)	0.689
27	gabor(1,0.4,absmean)	0.689

19	gabor(2,0.4,var)	0.688
27	gabor(2,0.1,mag)	0.688

Table C: T_2-weighted imaging: statistical features.

Window size	Texture	AUC
NA	stats(min)	0.726
NA	stats(decile2)	0.637
NA	stats(decile1)	0.636
NA	stats(q1)	0.635
NA	stats(decile3)	0.632
NA	stats(decile4)	0.624
NA	stats(mean)	0.621
NA	stats(decile5)	0.62
NA	stats(decile6)	0.614
NA	stats(decile7)	0.614
NA	stats(q3)	0.601
NA	stats(decile8)	0.596
NA	stats(decile9)	0.563
NA	stats(range)	0.554
NA	stats(stddev)	0.549
NA	stats(kurtosis)	0.537
NA	stats(skewness)	0.519
NA	stats(max)	0.516

Table D: Apparent diffusion coefficient of the monoexponential model (ADC_m): best 1% features.

Window size	Texture	AUC
11	gabor(1,0.1,mean)	0.802
11	gabor(1,0.1,absmean)	0.802
11	gabor(1,0.2,mean)	0.802
11	gabor(1,0.2,absmean)	0.802
11	gabor(1,0.3,mean)	0.802
11	gabor(1,0.4,mean)	0.802
11	gabor(2,0.1,mean)	0.802
11	gabor(2,0.1,absmean)	0.802
11	gabor(2,0.2,mean)	0.802
11	gabor(2,0.3,mean)	0.802
11	gabor(2,0.4,mean)	0.802
11	gabor(3,0.1,mean)	0.802

Table E: Apparent diffusion coefficient of the monoexponential model (ADC_m): statistical features.

Window size	Texture	AUC
NA	stats(min)	0.794
NA	stats(decile1)	0.76
NA	stats(decile2)	0.744
NA	stats(q1)	0.738
NA	stats(decile3)	0.728
NA	stats(mean)	0.71
NA	stats(decile4)	0.709
NA	stats(decile5)	0.689
NA	stats(decile6)	0.679
NA	stats(decile7)	0.666
NA	stats(q3)	0.66
NA	stats(decile8)	0.659
NA	stats(decile9)	0.638
NA	stats(range)	0.614
NA	stats(stddev)	0.587
NA	stats(skewness)	0.514
NA	stats(kurtosis)	0.511
NA	stats(max)	0.501

Table F: Apparent diffusion coefficient of the kurtosis model (ADC_k): best 1% features.

Window size	Texture	AUC
11	gabor(2,0.1,mag)	0.799
11	gabor(1,0.1,mag)	0.798
11	gabor(3,0.1,absmean)	0.798
11	gabor(1,0.1,mean)	0.797
11	gabor(1,0.1,absmean)	0.797
11	gabor(1,0.2,mean)	0.797
11	gabor(1,0.2,absmean)	0.797
11	gabor(1,0.2,mag)	0.797
11	gabor(1,0.3,mean)	0.797
11	gabor(1,0.3,mag)	0.797
11	gabor(1,0.4,mean)	0.797
11	gabor(2,0.1,mean)	0.797

Table G: Apparent diffusion coefficient of the kurtosis model (ADC_k): statistical features.

Window size	Texture	AUC

NA	stats(min)	0.793
NA	stats(decile1)	0.768
NA	stats(decile2)	0.746
NA	stats(q1)	0.738
NA	stats(decile3)	0.733
NA	stats(decile4)	0.715
NA	stats(mean)	0.71
NA	stats(decile5)	0.691
NA	stats(decile6)	0.672
NA	stats(stddev)	0.66
NA	stats(decile7)	0.651
NA	stats(q3)	0.642
NA	stats(range)	0.64
NA	stats(decile8)	0.627
NA	stats(decile9)	0.579
NA	stats(max)	0.519
NA	stats(skewness)	0.511
NA	stats(kurtosis)	0.501

Table H: Kurtosis parameter of the kurtosis model (K): best 1% features.

Window size	Texture	AUC
7	gabor(3,0.3,var)	0.841
7	gabor(2,0.4,mag)	0.838
7	gabor(2,0.4,absmean)	0.837
7	gabor(3,0.3,absmean)	0.837
7	gabor(3,0.3,mag)	0.837
7	zernike(7)	0.836
7	gabor(2,0.4,var)	0.834
7	gabor(3,0.4,var)	0.829
7	gabor(2,0.3,var)	0.824
7	gabor(3,0.4,absmean)	0.824
7	gabor(3,0.4,mag)	0.821
7	gabor(2,0.3,absmean)	0.815

Table I: Kurtosis parameter of the kurtosis model (K): statistical features.

Window size	Texture	AUC
NA	stats(range)	0.786
NA	stats(stddev)	0.776
NA	stats(max)	0.776
NA	stats(decile9)	0.74

NA	stats(decile8)	0.714
NA	stats(kurtosis)	0.712
NA	stats(q3)	0.709
NA	stats(decile7)	0.697
NA	stats(min)	0.693
NA	stats(decile6)	0.668
NA	stats(mean)	0.658
NA	stats(decile5)	0.641
NA	stats(decile4)	0.617
NA	stats(decile3)	0.608
NA	stats(q1)	0.602
NA	stats(decile2)	0.577
NA	stats(decile1)	0.524
NA	stats(skewness)	0.514

Table J: T_2 relaxation values (T_2): best 1% features.

Window size	Texture	AUC
15	hu(2)	0.749
27	hu(1)	0.741
19	hu(2)	0.722
19	hu(4)	0.719
27	hu(5)	0.716
19	hu(6)	0.714
3	zernike(20)	0.713
27	zernike(2)	0.71
27	hu(4)	0.708
27	zernike(3)	0.708
23	hu(2)	0.704
15	zernike(8)	0.701
27	glcm(correlation,3)	0.699
27	glcm(correlation,avg)	0.698
19	hu(5)	0.698
27	glcm(correlation,1)	0.696

Table K: T_2 relaxation values (T_2): statistical features.

Window size	Texture	AUC
NA	stats(decile2)	0.555
NA	stats(decile1)	0.553
NA	stats(decile9)	0.552
NA	stats(max)	0.551
NA	stats(q1)	0.547
NA	stats(range)	0.546

NA	stats(decile3)	0.541
NA	stats(q3)	0.528
NA	stats(decile8)	0.528
NA	stats(decile4)	0.527
NA	stats(stddev)	0.526
NA	stats(mean)	0.525
NA	stats(skewness)	0.521
NA	stats(decile6)	0.519
NA	stats(decile7)	0.518
NA	stats(decile5)	0.514
NA	stats(kurtosis)	0.511
NA	stats(min)	0.51

Table L: Final model features.

Image type	Window size	Texture	AUC
T2W	mbb	glcm(homogeneity,3,range)	0.847
K	7	zernike(7)	0.836
K	7	gabor(2,0.3,absmean)	0.818
K	7	gabor(2,0.3,var)	0.814
K	7	gabor(2,0.3,mag)	0.813
K	9	gabor(2,0.3,absmean)	0.811
K	9	gabor(2,0.3,mag)	0.809
ADCm	11	gabor(1,0.3,mean)	0.804
ADCm	11	gabor(1,0.3,absmean)	0.804
ADCm	11	gabor(3,0.1,absmean)	0.804
K	7	gabor(3,0.2,var)	0.803
K	7	gabor(3,0.2,mag)	0.803
K	7	gabor(1,0.4,var)	0.802
ADCm	11	gabor(2,0.1,mag)	0.801
ADCm	11	gabor(2,0.1,mean)	0.801
ADCm	11	gabor(2,0.1,absmean)	0.801
ADCm	11	gabor(1,0.2,mag)	0.799
ADCm	11	gabor(1,0.3,mag)	0.799
K	9	gabor(2,0.3,var)	0.799
ADCm	11	gabor(1,0.1,mean)	0.799
ADCm	11	gabor(1,0.1,absmean)	0.799
ADCm	9	gabor(2,0.1,mag)	0.798
ADCm	11	gabor(1,0.2,mean)	0.798
ADCm	11	gabor(1,0.2,absmean)	0.798
ADCm	9	gabor(1,0.1,mean)	0.798
ADCm	9	gabor(1,0.1,absmean)	0.798
ADCm	9	gabor(1,0.1,mag)	0.798
ADCm	11	gabor(1,0.1,mag)	0.798
K	7	gabor(3,0.2,absmean)	0.798

ADCm	9	gabor(2,0.1,mean)	0.797
ADCm	9	gabor(2,0.1,absmean)	0.797
T2W	mbb	glcm(homogeneity,4,range)	0.797
ADCm	11	gabor(1,0.4,mean)	0.796
ADCm	11	gabor(1,0.4,absmean)	0.794
ADCm	11	gabor(3,0.1,mag)	0.794
ADCm	NA	stats(min)	0.794
ADCm	9	gabor(1,0.2,mean)	0.792
ADCm	9	gabor(1,0.2,absmean)	0.793
ADCm	9	gabor(1,0.2,mag)	0.791
ADCm	11	hu(0)	0.791
ADCm	9	gabor(3,0.1,mag)	0.790

Table M: Best features' correlations among image types, sorted by absolute Spearman rank correlation coefficients (ρ), with corresponding p values and the features' single performance estimates as ROC AUCs.

Image types	Window size	Texture	ρ	p	AUCs
ADCk, ADCm	11	gabor(1,0.3,mean)	0.96	0.00	0.80, 0.80
ADCk, ADCm	11	gabor(2,0.1,mean)	0.95	0.00	0.80, 0.80
ADCk, ADCm	NA	stats(min)	0.89	0.00	0.79, 0.79
ADCk, ADCm	NA	stats(min)	0.89	0.00	0.79, 0.79
ADCm, K	11	gabor(1,0.3,mean)	-0.79	0.00	0.80, 0.74
ADCk, K	11	gabor(2,0.1,mean)	-0.71	0.00	0.80, 0.74
ADCk, K	NA	stats(range)	0.67	0.00	0.64, 0.79
ADCm, K	NA	stats(range)	0.63	0.00	0.61, 0.79
T2, T2W	NA	stats(min)	0.53	0.00	0.51, 0.73
ADCk, T2W	NA	stats(min)	0.49	0.00	0.79, 0.73
ADCk, T2W	NA	stats(min)	0.49	0.00	0.79, 0.73
ADCk, K	NA	stats(min)	0.47	0.00	0.79, 0.69
ADCm, T2W	NA	stats(min)	0.44	0.00	0.79, 0.73
ADCm, T2W	NA	stats(min)	0.44	0.00	0.79, 0.73
K, T2W	NA	stats(range)	0.43	0.00	0.79, 0.55
ADCm, K	7	zernike(7)	0.42	0.00	0.67, 0.84
ADCk, T2	NA	stats(decile2)	0.40	0.00	0.75, 0.56
T2, T2W	35	zernike(3)	0.40	0.00	0.76, 0.61
ADCm, K	NA	stats(min)	0.39	0.00	0.79, 0.69
K, T2W	mbb	glcm(homogeneity,3,range)	0.39	0.00	0.73, 0.85
T2, T2W	mbb	glcm(homogeneity,3,range)	0.39	0.00	0.61, 0.85
K, T2W	NA	stats(min)	0.38	0.00	0.69, 0.73
ADCk, K	7	zernike(7)	0.38	0.00	0.64, 0.84
ADCm, T2W	mbb	glcm(homogeneity,3,range)	0.37	0.00	0.73, 0.85
ADCk, T2	NA	stats(min)	0.35	0.00	0.79, 0.51
ADCm, T2	NA	stats(decile2)	0.34	0.00	0.74, 0.56

T2, T2W	NA	stats(decile2)	0.33	0.00	0.56, 0.64
ADCk, T2W	mbb	glcm(homogeneity,3,range)	0.32	0.00	0.67, 0.85
ADCm, T2	NA	stats(min)	0.29	0.00	0.79, 0.51
ADCk, T2	11	gabor(2,0.1,mean)	0.20	0.05	0.80, 0.55
K, T2	NA	stats(range)	0.18	0.07	0.79, 0.55
K, T2	NA	stats(decile2)	-0.16	0.11	0.58, 0.56
K, T2	7	zernike(7)	-0.16	0.12	0.84, 0.54
ADCm, T2	11	gabor(1,0.3,mean)	0.15	0.13	0.80, 0.55
K, T2W	7	zernike(7)	0.14	0.17	0.84, 0.54
ADCm, T2W	11	gabor(1,0.3,mean)	0.12	0.25	0.80, 0.59
ADCk, T2W	11	gabor(2,0.1,mean)	0.10	0.30	0.80, 0.59

Table N: All possible pairs of top five features of each image type, sorted by absolute Spearman rank correlation coefficients (ρ), with corresponding p values and the features' single performance estimates as ROC AUCs. Here image type and window size are included in the front of feature names.

Textures	ρ	p	AUCs
ADCm-11-gabor(1,0.1,absmean), ADCm-11-gabor(1,0.1,mean)	1.00	0.00	0.80, 0.80
ADCk-11-gabor(1,0.1,absmean), ADCk-11-gabor(1,0.1,mean)	1.00	0.00	0.80, 0.80
ADCm-11-gabor(1,0.2,absmean), ADCm-11-gabor(1,0.2,mean)	1.00	0.00	0.80, 0.80
ADCk-11-gabor(1,0.1,mag), ADCk-11-gabor(1,0.1,mean)	1.00	0.00	0.80, 0.80
ADCk-11-gabor(1,0.1,absmean), ADCk-11-gabor(1,0.1,mag)	1.00	0.00	0.80, 0.80
T2W-27-gabor(1,0.1,var), T2W-27-gabor(2,0.1,var)	1.00	0.00	0.71, 0.71
ADCm-11-gabor(1,0.1,mean), ADCm-11-gabor(1,0.2,mean)	1.00	0.00	0.80, 0.80
ADCm-11-gabor(1,0.1,mean), ADCm-11-gabor(1,0.2,absmean)	1.00	0.00	0.80, 0.80
ADCm-11-gabor(1,0.1,absmean), ADCm-11-gabor(1,0.2,mean)	1.00	0.00	0.80, 0.80
ADCm-11-gabor(1,0.1,absmean), ADCm-11-gabor(1,0.2,absmean)	1.00	0.00	0.80, 0.80
ADCk-11-gabor(1,0.1,mag), ADCk-11-gabor(2,0.1,mag)	1.00	0.00	0.80, 0.80
ADCk-11-gabor(1,0.1,absmean), ADCk-11-gabor(2,0.1,mag)	1.00	0.00	0.80, 0.80
ADCk-11-gabor(1,0.1,mean), ADCk-11-gabor(2,0.1,mag)	1.00	0.00	0.80, 0.80
K-7-gabor(3,0.3,absmean), K-7-gabor(3,0.3,mag)	1.00	0.00	0.76, 0.76
K-7-gabor(2,0.4,absmean), K-7-gabor(2,0.4,mag)	1.00	0.00	0.77, 0.75
ADCm-11-gabor(1,0.2,mean), ADCm-11-gabor(1,0.3,mean)	0.99	0.00	0.80, 0.80
ADCm-11-gabor(1,0.2,absmean), ADCm-11-gabor(1,0.3,mean)	0.99	0.00	0.80, 0.80
T2W-27-gabor(1,0.1,var), T2W-27-gabor(3,0.1,var)	0.99	0.00	0.71, 0.73
T2W-27-gabor(2,0.1,var), T2W-27-gabor(3,0.1,var)	0.99	0.00	0.71, 0.73
ADCm-11-gabor(1,0.1,absmean), ADCm-11-gabor(1,0.3,mean)	0.99	0.00	0.80, 0.80
ADCm-11-gabor(1,0.1,mean), ADCm-11-gabor(1,0.3,mean)	0.99	0.00	0.80, 0.80
K-7-gabor(3,0.3,mag), K-7-gabor(3,0.3,var)	0.98	0.00	0.76, 0.75
ADCk-11-gabor(1,0.1,mag), ADCk-11-gabor(3,0.1,absmean)	0.98	0.00	0.80, 0.79
ADCk-11-gabor(1,0.1,mean), ADCk-11-gabor(3,0.1,absmean)	0.98	0.00	0.80, 0.79
ADCk-11-gabor(1,0.1,absmean), ADCk-11-gabor(3,0.1,absmean)	0.98	0.00	0.80, 0.79
K-7-gabor(3,0.3,absmean), K-7-gabor(3,0.3,var)	0.98	0.00	0.76, 0.75

ADCk-11-gabor(2,0.1,mag), ADCk-11-gabor(3,0.1,absmean)	0.97	0.00	0.80, 0.79
ADCk-11-gabor(1,0.1,mean), ADCm-11-gabor(1,0.3,mean)	0.96	0.00	0.80, 0.80
ADCk-11-gabor(1,0.1,absmean), ADCm-11-gabor(1,0.3,mean)	0.96	0.00	0.80, 0.80
ADCk-11-gabor(1,0.1,mag), ADCm-11-gabor(1,0.3,mean)	0.96	0.00	0.80, 0.80
ADCk-11-gabor(2,0.1,mag), ADCm-11-gabor(1,0.2,absmean)	0.96	0.00	0.80, 0.80
ADCk-11-gabor(2,0.1,mag), ADCm-11-gabor(1,0.2,mean)	0.96	0.00	0.80, 0.80
ADCk-11-gabor(1,0.1,absmean), ADCm-11-gabor(1,0.2,mean)	0.96	0.00	0.80, 0.80
ADCk-11-gabor(1,0.1,mean), ADCm-11-gabor(1,0.2,absmean)	0.96	0.00	0.80, 0.80
ADCk-11-gabor(1,0.1,mean), ADCm-11-gabor(1,0.2,mean)	0.96	0.00	0.80, 0.80
ADCk-11-gabor(1,0.1,absmean), ADCm-11-gabor(1,0.2,absmean)	0.96	0.00	0.80, 0.80
ADCk-11-gabor(1,0.1,mag), ADCm-11-gabor(1,0.2,mean)	0.96	0.00	0.80, 0.80
ADCk-11-gabor(1,0.1,mag), ADCm-11-gabor(1,0.2,absmean)	0.96	0.00	0.80, 0.80
ADCk-11-gabor(2,0.1,mag), ADCm-11-gabor(1,0.1,absmean)	0.96	0.00	0.80, 0.80
ADCk-11-gabor(2,0.1,mag), ADCm-11-gabor(1,0.1,mean)	0.96	0.00	0.80, 0.80
ADCk-11-gabor(2,0.1,mag), ADCm-11-gabor(1,0.3,mean)	0.96	0.00	0.80, 0.80
ADCk-11-gabor(1,0.1,absmean), ADCm-11-gabor(1,0.1,mean)	0.95	0.00	0.80, 0.80
ADCk-11-gabor(1,0.1,absmean), ADCm-11-gabor(1,0.1,absmean)	0.95	0.00	0.80, 0.80
ADCk-11-gabor(1,0.1,mean), ADCm-11-gabor(1,0.1,mean)	0.95	0.00	0.80, 0.80
ADCk-11-gabor(1,0.1,mean), ADCm-11-gabor(1,0.1,absmean)	0.95	0.00	0.80, 0.80
ADCk-11-gabor(1,0.1,mag), ADCm-11-gabor(1,0.1,absmean)	0.95	0.00	0.80, 0.80
ADCk-11-gabor(1,0.1,mag), ADCm-11-gabor(1,0.1,mean)	0.95	0.00	0.80, 0.80
ADCk-11-gabor(3,0.1,absmean), ADCm-11-gabor(1,0.3,mean)	0.94	0.00	0.79, 0.80
ADCk-11-gabor(3,0.1,absmean), ADCm-11-gabor(1,0.2,absmean)	0.92	0.00	0.79, 0.80
ADCk-11-gabor(3,0.1,absmean), ADCm-11-gabor(1,0.2,mean)	0.92	0.00	0.79, 0.80
ADCk-11-gabor(3,0.1,absmean), ADCm-11-gabor(1,0.1,absmean)	0.91	0.00	0.79, 0.80
ADCk-11-gabor(3,0.1,absmean), ADCm-11-gabor(1,0.1,mean)	0.91	0.00	0.79, 0.80
K-7-gabor(2,0.4,absmean), K-7-gabor(3,0.3,absmean)	0.91	0.00	0.77, 0.76
K-7-gabor(2,0.4,mag), K-7-gabor(3,0.3,absmean)	0.91	0.00	0.75, 0.76
K-7-gabor(2,0.4,absmean), K-7-gabor(3,0.3,mag)	0.91	0.00	0.77, 0.76
K-7-gabor(2,0.4,mag), K-7-gabor(3,0.3,mag)	0.91	0.00	0.75, 0.76
K-7-gabor(2,0.4,absmean), K-7-gabor(3,0.3,var)	0.89	0.00	0.77, 0.75
K-7-gabor(2,0.4,mag), K-7-gabor(3,0.3,var)	0.88	0.00	0.75, 0.75
T2-27-hu(1), T2-27-hu(5)	0.68	0.00	0.74, 0.72
T2-15-hu(2), T2-19-hu(2)	0.67	0.00	0.75, 0.72
T2-19-hu(2), T2-19-hu(4)	0.60	0.00	0.72, 0.72
T2-15-hu(2), T2-19-hu(4)	0.48	0.00	0.75, 0.72
ADCk-11-gabor(1,0.1,mag), T2W-NA-stats(min)	0.46	0.00	0.80, 0.73
ADCk-11-gabor(1,0.1,absmean), T2W-NA-stats(min)	0.46	0.00	0.80, 0.73
ADCk-11-gabor(1,0.1,mean), T2W-NA-stats(min)	0.46	0.00	0.80, 0.73
ADCk-11-gabor(2,0.1,mag), T2W-NA-stats(min)	0.46	0.00	0.80, 0.73
ADCm-11-gabor(1,0.3,mean), T2W-NA-stats(min)	0.46	0.00	0.80, 0.73
ADCk-11-gabor(3,0.1,absmean), T2W-NA-stats(min)	0.46	0.00	0.79, 0.73
ADCm-11-gabor(1,0.2,mean), T2W-27-gabor(3,0.1,var)	0.46	0.00	0.80, 0.73
ADCm-11-gabor(1,0.2,absmean), T2W-27-gabor(3,0.1,var)	0.46	0.00	0.80, 0.73
ADCm-11-gabor(1,0.2,absmean), T2W-NA-stats(min)	0.46	0.00	0.80, 0.73
ADCm-11-gabor(1,0.2,mean), T2W-NA-stats(min)	0.46	0.00	0.80, 0.73

ADCm-11-gabor(1,0.1,absmean), T2W-27-gabor(3,0.1,var)	0.46	0.00	0.80, 0.73
ADCm-11-gabor(1,0.1,mean), T2W-27-gabor(3,0.1,var)	0.46	0.00	0.80, 0.73
ADCm-11-gabor(1,0.2,absmean), T2W-27-gabor(1,0.1,var)	0.45	0.00	0.80, 0.71
ADCm-11-gabor(1,0.2,mean), T2W-27-gabor(1,0.1,var)	0.45	0.00	0.80, 0.71
ADCm-11-gabor(1,0.1,mean), T2W-27-gabor(1,0.1,var)	0.45	0.00	0.80, 0.71
ADCm-11-gabor(1,0.1,absmean), T2W-27-gabor(1,0.1,var)	0.45	0.00	0.80, 0.71
ADCm-11-gabor(1,0.3,mean), T2W-27-gabor(3,0.1,var)	0.45	0.00	0.80, 0.73
ADCm-11-gabor(1,0.1,mean), T2W-27-gabor(2,0.1,var)	0.45	0.00	0.80, 0.71
ADCm-11-gabor(1,0.1,absmean), T2W-27-gabor(2,0.1,var)	0.45	0.00	0.80, 0.71
ADCm-11-gabor(1,0.2,absmean), T2W-27-gabor(2,0.1,var)	0.45	0.00	0.80, 0.71
ADCm-11-gabor(1,0.2,mean), T2W-27-gabor(2,0.1,var)	0.45	0.00	0.80, 0.71
ADCk-11-gabor(2,0.1,mag), T2W-27-gabor(3,0.1,var)	0.45	0.00	0.80, 0.73
ADCm-11-gabor(1,0.1,mean), T2W-NA-stats(min)	0.45	0.00	0.80, 0.73
ADCm-11-gabor(1,0.1,absmean), T2W-NA-stats(min)	0.45	0.00	0.80, 0.73
ADCk-11-gabor(2,0.1,mag), T2W-27-gabor(1,0.1,var)	0.44	0.00	0.80, 0.71
ADCk-11-gabor(1,0.1,absmean), T2W-27-gabor(3,0.1,var)	0.44	0.00	0.80, 0.73
ADCk-11-gabor(1,0.1,mean), T2W-27-gabor(3,0.1,var)	0.44	0.00	0.80, 0.73
ADCm-11-gabor(1,0.3,mean), T2W-27-gabor(1,0.1,var)	0.44	0.00	0.80, 0.71
ADCk-11-gabor(1,0.1,mag), T2W-27-gabor(3,0.1,var)	0.44	0.00	0.80, 0.73
ADCm-11-gabor(1,0.3,mean), T2W-27-gabor(2,0.1,var)	0.44	0.00	0.80, 0.71
ADCk-11-gabor(2,0.1,mag), T2W-27-gabor(2,0.1,var)	0.44	0.00	0.80, 0.71
ADCk-11-gabor(1,0.1,absmean), T2W-27-gabor(1,0.1,var)	0.44	0.00	0.80, 0.71
ADCk-11-gabor(1,0.1,mean), T2W-27-gabor(1,0.1,var)	0.44	0.00	0.80, 0.71
ADCk-11-gabor(1,0.1,mag), T2W-27-gabor(1,0.1,var)	0.44	0.00	0.80, 0.71
ADCk-11-gabor(1,0.1,absmean), T2W-27-gabor(2,0.1,var)	0.44	0.00	0.80, 0.71
ADCk-11-gabor(1,0.1,mean), T2W-27-gabor(2,0.1,var)	0.44	0.00	0.80, 0.71
ADCk-11-gabor(1,0.1,mag), T2W-27-gabor(2,0.1,var)	0.44	0.00	0.80, 0.71
ADCk-11-gabor(3,0.1,absmean), T2W-27-gabor(3,0.1,var)	0.41	0.00	0.79, 0.73
ADCk-11-gabor(3,0.1,absmean), T2W-27-gabor(1,0.1,var)	0.41	0.00	0.79, 0.71
ADCk-11-gabor(3,0.1,absmean), T2W-27-gabor(2,0.1,var)	0.40	0.00	0.79, 0.71
T2-19-hu(4), T2-27-hu(5)	0.40	0.00	0.72, 0.72
T2-19-hu(2), T2-27-hu(1)	0.37	0.00	0.72, 0.74
ADCm-11-gabor(1,0.1,absmean), T2-15-hu(2)	0.36	0.00	0.80, 0.75
ADCm-11-gabor(1,0.1,mean), T2-15-hu(2)	0.36	0.00	0.80, 0.75
ADCm-11-gabor(1,0.2,mean), T2-15-hu(2)	0.36	0.00	0.80, 0.75
ADCm-11-gabor(1,0.2,absmean), T2-15-hu(2)	0.36	0.00	0.80, 0.75
ADCm-11-gabor(1,0.3,mean), T2-15-hu(2)	0.35	0.00	0.80, 0.75
ADCk-11-gabor(1,0.1,mag), T2-15-hu(2)	0.35	0.00	0.80, 0.75
ADCk-11-gabor(1,0.1,mean), T2-15-hu(2)	0.35	0.00	0.80, 0.75
ADCk-11-gabor(1,0.1,absmean), T2-15-hu(2)	0.35	0.00	0.80, 0.75
ADCk-11-gabor(2,0.1,mag), T2-15-hu(2)	0.35	0.00	0.80, 0.75
T2-15-hu(2), T2W-27-gabor(1,0.1,var)	0.34	0.00	0.75, 0.71
T2-15-hu(2), T2W-27-gabor(3,0.1,var)	0.34	0.00	0.75, 0.73
T2-15-hu(2), T2W-27-gabor(2,0.1,var)	0.34	0.00	0.75, 0.71
ADCm-11-gabor(1,0.3,mean), K-7-gabor(3,0.3,absmean)	-0.33	0.00	0.80, 0.76
ADCm-11-gabor(1,0.1,absmean), T2-27-hu(1)	0.33	0.00	0.80, 0.74

ADCm-11-gabor(1,0.1,mean), T2-27-hu(1)	0.33	0.00	0.80, 0.74
ADCk-11-gabor(3,0.1,absmean), T2-15-hu(2)	0.32	0.00	0.79, 0.75
ADCm-11-gabor(1,0.3,mean), K-7-gabor(3,0.3,mag)	-0.32	0.00	0.80, 0.76
ADCk-11-gabor(2,0.1,mag), T2-27-hu(1)	0.32	0.00	0.80, 0.74
ADCm-11-gabor(1,0.2,mean), T2-27-hu(1)	0.32	0.00	0.80, 0.74
ADCm-11-gabor(1,0.2,absmean), T2-27-hu(1)	0.32	0.00	0.80, 0.74
T2-15-hu(2), T2-27-hu(1)	0.32	0.00	0.75, 0.74
ADCk-11-gabor(1,0.1,mag), T2-27-hu(1)	0.31	0.00	0.80, 0.74
ADCk-11-gabor(1,0.1,mean), T2-27-hu(1)	0.31	0.00	0.80, 0.74
ADCk-11-gabor(1,0.1,absmean), T2-27-hu(1)	0.31	0.00	0.80, 0.74
ADCk-11-gabor(3,0.1,absmean), K-7-gabor(2,0.4,absmean)	-0.31	0.00	0.79, 0.77
ADCm-11-gabor(1,0.2,mean), K-7-gabor(3,0.3,absmean)	-0.31	0.00	0.80, 0.76
ADCm-11-gabor(1,0.2,absmean), K-7-gabor(3,0.3,absmean)	-0.31	0.00	0.80, 0.76
ADCm-11-gabor(1,0.1,absmean), T2-19-hu(2)	0.31	0.00	0.80, 0.72
ADCm-11-gabor(1,0.1,mean), T2-19-hu(2)	0.31	0.00	0.80, 0.72
T2-19-hu(2), T2W-27-gabor(3,0.1,var)	0.31	0.00	0.72, 0.73
ADCm-11-gabor(1,0.3,mean), T2-27-hu(1)	0.31	0.00	0.80, 0.74
T2-27-hu(1), T2W-27-gabor(1,0.1,var)	0.31	0.00	0.74, 0.71
T2-27-hu(1), T2W-27-gabor(3,0.1,var)	0.31	0.00	0.74, 0.73
ADCm-11-gabor(1,0.1,mean), K-7-gabor(3,0.3,absmean)	-0.31	0.00	0.80, 0.76
ADCm-11-gabor(1,0.1,absmean), K-7-gabor(3,0.3,absmean)	-0.31	0.00	0.80, 0.76
ADCm-11-gabor(1,0.3,mean), K-7-gabor(3,0.3,var)	-0.31	0.00	0.80, 0.75
T2-27-hu(1), T2W-27-gabor(2,0.1,var)	0.31	0.00	0.74, 0.71
ADCm-11-gabor(1,0.2,absmean), K-7-gabor(3,0.3,mag)	-0.31	0.00	0.80, 0.76
ADCm-11-gabor(1,0.2,mean), K-7-gabor(3,0.3,mag)	-0.31	0.00	0.80, 0.76
T2-19-hu(2), T2W-27-gabor(2,0.1,var)	0.30	0.00	0.72, 0.71
T2W-27-gabor(3,0.1,var), T2W-NA-stats(min)	0.30	0.00	0.73, 0.73
ADCk-11-gabor(1,0.1,absmean), K-7-gabor(3,0.3,absmean)	-0.30	0.00	0.80, 0.76
ADCk-11-gabor(1,0.1,mean), K-7-gabor(3,0.3,absmean)	-0.30	0.00	0.80, 0.76
ADCm-11-gabor(1,0.2,mean), T2-19-hu(2)	0.30	0.00	0.80, 0.72
ADCm-11-gabor(1,0.2,absmean), T2-19-hu(2)	0.30	0.00	0.80, 0.72
ADCk-11-gabor(3,0.1,absmean), K-7-gabor(2,0.4,mag)	-0.30	0.00	0.79, 0.75
ADCk-11-gabor(1,0.1,mag), K-7-gabor(3,0.3,absmean)	-0.30	0.00	0.80, 0.76
ADCk-11-gabor(3,0.1,absmean), K-7-gabor(3,0.3,absmean)	-0.30	0.00	0.79, 0.76
T2-19-hu(2), T2W-27-gabor(1,0.1,var)	0.30	0.00	0.72, 0.71
ADCm-11-gabor(1,0.3,mean), K-7-gabor(2,0.4,absmean)	-0.30	0.00	0.80, 0.77
ADCk-11-gabor(3,0.1,absmean), T2-27-hu(1)	0.30	0.00	0.79, 0.74
ADCm-11-gabor(1,0.1,mean), K-7-gabor(3,0.3,mag)	-0.30	0.00	0.80, 0.76
ADCm-11-gabor(1,0.1,absmean), K-7-gabor(3,0.3,mag)	-0.30	0.00	0.80, 0.76
ADCk-11-gabor(2,0.1,mag), K-7-gabor(3,0.3,absmean)	-0.30	0.00	0.80, 0.76
ADCm-11-gabor(1,0.3,mean), T2-19-hu(2)	0.30	0.00	0.80, 0.72
ADCk-11-gabor(1,0.1,mean), K-7-gabor(3,0.3,mag)	-0.30	0.00	0.80, 0.76
ADCk-11-gabor(1,0.1,absmean), K-7-gabor(3,0.3,mag)	-0.30	0.00	0.80, 0.76
ADCk-11-gabor(3,0.1,absmean), K-7-gabor(3,0.3,mag)	-0.30	0.00	0.79, 0.76
ADCk-11-gabor(1,0.1,mag), K-7-gabor(3,0.3,mag)	-0.30	0.00	0.80, 0.76
ADCm-11-gabor(1,0.2,mean), K-7-gabor(3,0.3,var)	-0.29	0.00	0.80, 0.75

ADCm-11-gabor(1,0.2,absmean), K-7-gabor(3,0.3,var)	-0.29	0.00	0.80, 0.75
ADCk-11-gabor(2,0.1,mag), K-7-gabor(3,0.3,mag)	-0.29	0.00	0.80, 0.76
ADCk-11-gabor(1,0.1,mag), T2-19-hu(2)	0.29	0.00	0.80, 0.72
ADCm-11-gabor(1,0.1,absmean), K-7-gabor(3,0.3,var)	-0.29	0.00	0.80, 0.75
ADCm-11-gabor(1,0.1,mean), K-7-gabor(3,0.3,var)	-0.29	0.00	0.80, 0.75
ADCm-11-gabor(1,0.3,mean), K-7-gabor(2,0.4,mag)	-0.29	0.00	0.80, 0.75
ADCk-11-gabor(1,0.1,mean), T2-19-hu(2)	0.29	0.00	0.80, 0.72
ADCk-11-gabor(1,0.1,absmean), T2-19-hu(2)	0.29	0.00	0.80, 0.72
ADCk-11-gabor(1,0.1,absmean), K-7-gabor(2,0.4,absmean)	-0.29	0.00	0.80, 0.77
ADCk-11-gabor(1,0.1,mean), K-7-gabor(2,0.4,absmean)	-0.29	0.00	0.80, 0.77
ADCk-11-gabor(1,0.1,mag), K-7-gabor(2,0.4,absmean)	-0.29	0.00	0.80, 0.77
ADCk-11-gabor(2,0.1,mag), T2-19-hu(2)	0.29	0.00	0.80, 0.72
T2-15-hu(2), T2-27-hu(5)	0.28	0.00	0.75, 0.72
ADCk-11-gabor(1,0.1,absmean), K-7-gabor(3,0.3,var)	-0.28	0.01	0.80, 0.75
ADCk-11-gabor(1,0.1,mean), K-7-gabor(3,0.3,var)	-0.28	0.01	0.80, 0.75
ADCk-11-gabor(1,0.1,mag), K-7-gabor(3,0.3,var)	-0.28	0.01	0.80, 0.75
ADCk-11-gabor(1,0.1,absmean), K-7-gabor(2,0.4,mag)	-0.28	0.01	0.80, 0.75
ADCk-11-gabor(1,0.1,mean), K-7-gabor(2,0.4,mag)	-0.28	0.01	0.80, 0.75
ADCk-11-gabor(2,0.1,mag), K-7-gabor(2,0.4,absmean)	-0.28	0.01	0.80, 0.77
ADCk-11-gabor(1,0.1,mag), K-7-gabor(2,0.4,mag)	-0.27	0.01	0.80, 0.75
ADCk-11-gabor(3,0.1,absmean), K-7-gabor(3,0.3,var)	-0.27	0.01	0.79, 0.75
ADCk-11-gabor(2,0.1,mag), K-7-gabor(3,0.3,var)	-0.27	0.01	0.80, 0.75
T2W-27-gabor(1,0.1,var), T2W-NA-stats(min)	0.27	0.01	0.71, 0.73
ADCm-11-gabor(1,0.2,absmean), K-7-gabor(2,0.4,absmean)	-0.27	0.01	0.80, 0.77
ADCm-11-gabor(1,0.2,mean), K-7-gabor(2,0.4,absmean)	-0.27	0.01	0.80, 0.77
ADCk-11-gabor(3,0.1,absmean), T2-19-hu(2)	0.27	0.01	0.79, 0.72
ADCk-11-gabor(2,0.1,mag), K-7-gabor(2,0.4,mag)	-0.26	0.01	0.80, 0.75
ADCm-11-gabor(1,0.1,mean), K-7-gabor(2,0.4,absmean)	-0.26	0.01	0.80, 0.77
ADCm-11-gabor(1,0.1,absmean), K-7-gabor(2,0.4,absmean)	-0.26	0.01	0.80, 0.77
ADCm-11-gabor(1,0.2,absmean), K-7-gabor(2,0.4,mag)	-0.25	0.01	0.80, 0.75
ADCm-11-gabor(1,0.2,mean), K-7-gabor(2,0.4,mag)	-0.25	0.01	0.80, 0.75
T2W-27-gabor(2,0.1,var), T2W-NA-stats(min)	0.25	0.01	0.71, 0.73
T2-19-hu(2), T2-27-hu(5)	0.25	0.01	0.72, 0.72
ADCm-11-gabor(1,0.1,absmean), K-7-gabor(2,0.4,mag)	-0.24	0.01	0.80, 0.75
ADCm-11-gabor(1,0.1,mean), K-7-gabor(2,0.4,mag)	-0.24	0.01	0.80, 0.75
T2-19-hu(4), T2-27-hu(1)	0.24	0.02	0.72, 0.74
K-7-gabor(2,0.4,absmean), T2W-NA-stats(min)	-0.22	0.03	0.77, 0.73
K-7-gabor(3,0.3,absmean), T2W-NA-stats(min)	-0.22	0.03	0.76, 0.73
K-7-gabor(3,0.3,var), T2W-NA-stats(min)	-0.21	0.04	0.75, 0.73
K-7-gabor(2,0.4,mag), T2W-NA-stats(min)	-0.21	0.04	0.75, 0.73
K-7-gabor(3,0.3,mag), T2W-NA-stats(min)	-0.20	0.04	0.76, 0.73
ADCk-11-gabor(2,0.1,mag), T2W-3-zernike(20)	-0.20	0.05	0.80, 0.52
T2-27-hu(1), T2W-NA-stats(min)	0.19	0.06	0.74, 0.73
ADCk-11-gabor(1,0.1,mag), T2W-3-zernike(20)	-0.19	0.06	0.80, 0.52
ADCk-11-gabor(1,0.1,absmean), T2W-3-zernike(20)	-0.19	0.06	0.80, 0.52
ADCk-11-gabor(1,0.1,mean), T2W-3-zernike(20)	-0.19	0.06	0.80, 0.52

T2-19-hu(4), T2W-27-gabor(3,0.1,var)	0.18	0.07	0.72, 0.73
ADCk-11-gabor(3,0.1,absmean), T2W-3-zernike(20)	-0.18	0.07	0.79, 0.52
T2-19-hu(4), T2W-27-gabor(2,0.1,var)	0.18	0.07	0.72, 0.71
T2-27-hu(5), T2W-27-gabor(2,0.1,var)	0.18	0.08	0.72, 0.71
T2-19-hu(4), T2W-27-gabor(1,0.1,var)	0.18	0.08	0.72, 0.71
T2-27-hu(5), T2W-27-gabor(1,0.1,var)	0.17	0.09	0.72, 0.71
ADCm-11-gabor(1,0.1,mean), T2W-3-zernike(20)	-0.17	0.10	0.80, 0.52
ADCm-11-gabor(1,0.1,absmean), T2W-3-zernike(20)	-0.17	0.10	0.80, 0.52
T2-27-hu(5), T2W-27-gabor(3,0.1,var)	0.16	0.11	0.72, 0.73
ADCm-11-gabor(1,0.2,absmean), T2W-3-zernike(20)	-0.16	0.11	0.80, 0.52
ADCm-11-gabor(1,0.2,mean), T2W-3-zernike(20)	-0.16	0.11	0.80, 0.52
K-7-gabor(2,0.4,absmean), T2-15-hu(2)	-0.15	0.13	0.77, 0.75
T2W-27-gabor(3,0.1,var), T2W-3-zernike(20)	-0.15	0.13	0.73, 0.52
T2W-27-gabor(1,0.1,var), T2W-3-zernike(20)	-0.15	0.13	0.71, 0.52
ADCm-11-gabor(1,0.3,mean), T2W-3-zernike(20)	-0.15	0.14	0.80, 0.52
T2W-27-gabor(2,0.1,var), T2W-3-zernike(20)	-0.15	0.14	0.71, 0.52
K-7-gabor(2,0.4,mag), T2-15-hu(2)	-0.14	0.17	0.75, 0.75
T2-27-hu(5), T2W-3-zernike(20)	-0.13	0.19	0.72, 0.52
T2W-3-zernike(20), T2W-NA-stats(min)	-0.13	0.21	0.52, 0.73
T2-27-hu(1), T2W-3-zernike(20)	-0.12	0.23	0.74, 0.52
T2-15-hu(2), T2W-NA-stats(min)	0.11	0.26	0.75, 0.73
K-7-gabor(3,0.3,mag), T2-19-hu(2)	-0.11	0.27	0.76, 0.72
K-7-gabor(3,0.3,absmean), T2-19-hu(2)	-0.11	0.28	0.76, 0.72
T2-19-hu(4), T2W-3-zernike(20)	-0.11	0.28	0.72, 0.52
K-7-gabor(3,0.3,absmean), T2-15-hu(2)	-0.11	0.29	0.76, 0.75
K-7-gabor(3,0.3,mag), T2-15-hu(2)	-0.11	0.30	0.76, 0.75
ADCk-11-gabor(3,0.1,absmean), T2-19-hu(4)	0.10	0.30	0.79, 0.72
K-7-gabor(3,0.3,var), T2-19-hu(4)	-0.10	0.31	0.75, 0.72
ADCm-11-gabor(1,0.3,mean), T2-19-hu(4)	0.10	0.31	0.80, 0.72
K-7-gabor(3,0.3,absmean), T2-19-hu(4)	-0.10	0.32	0.76, 0.72
K-7-gabor(3,0.3,var), T2-15-hu(2)	-0.10	0.33	0.75, 0.75
K-7-gabor(2,0.4,absmean), T2-19-hu(2)	-0.10	0.33	0.77, 0.72
K-7-gabor(3,0.3,mag), T2-19-hu(4)	-0.09	0.35	0.76, 0.72
T2-19-hu(4), T2W-NA-stats(min)	-0.09	0.36	0.72, 0.73
K-7-gabor(2,0.4,mag), T2-19-hu(2)	-0.09	0.38	0.75, 0.72
ADCm-11-gabor(1,0.2,absmean), T2-19-hu(4)	0.09	0.38	0.80, 0.72
ADCm-11-gabor(1,0.2,mean), T2-19-hu(4)	0.09	0.38	0.80, 0.72
ADCk-11-gabor(1,0.1,mag), T2-19-hu(4)	0.09	0.39	0.80, 0.72
ADCm-11-gabor(1,0.1,absmean), T2-19-hu(4)	0.09	0.39	0.80, 0.72
ADCm-11-gabor(1,0.1,mean), T2-19-hu(4)	0.09	0.39	0.80, 0.72
ADCk-11-gabor(1,0.1,absmean), T2-19-hu(4)	0.09	0.39	0.80, 0.72
ADCk-11-gabor(1,0.1,mean), T2-19-hu(4)	0.09	0.39	0.80, 0.72
K-7-gabor(3,0.3,var), T2-19-hu(2)	-0.09	0.40	0.75, 0.72
ADCk-11-gabor(2,0.1,mag), T2-19-hu(4)	0.09	0.40	0.80, 0.72
T2-19-hu(2), T2W-3-zernike(20)	-0.08	0.43	0.72, 0.52
ADCk-11-gabor(3,0.1,absmean), T2-27-hu(5)	0.07	0.51	0.79, 0.72

ADCm-11-gabor(1,0.3,mean), T2-27-hu(5)	0.06	0.56	0.80, 0.72
ADCk-11-gabor(2,0.1,mag), T2-27-hu(5)	0.05	0.60	0.80, 0.72
T2-27-hu(5), T2W-NA-stats(min)	-0.05	0.60	0.72, 0.73
ADCm-11-gabor(1,0.1,mean), T2-27-hu(5)	0.05	0.61	0.80, 0.72
ADCm-11-gabor(1,0.1,absmean), T2-27-hu(5)	0.05	0.61	0.80, 0.72
ADCk-11-gabor(1,0.1,mag), T2-27-hu(5)	0.05	0.62	0.80, 0.72
ADCk-11-gabor(1,0.1,mean), T2-27-hu(5)	0.05	0.62	0.80, 0.72
ADCk-11-gabor(1,0.1,absmean), T2-27-hu(5)	0.05	0.62	0.80, 0.72
ADCm-11-gabor(1,0.2,mean), T2-27-hu(5)	0.05	0.62	0.80, 0.72
ADCm-11-gabor(1,0.2,absmean), T2-27-hu(5)	0.05	0.62	0.80, 0.72
K-7-gabor(2,0.4,absmean), T2-19-hu(4)	-0.05	0.63	0.77, 0.72
K-7-gabor(2,0.4,mag), T2-19-hu(4)	-0.05	0.63	0.75, 0.72
K-7-gabor(2,0.4,absmean), T2-27-hu(5)	-0.04	0.66	0.77, 0.72
K-7-gabor(2,0.4,mag), T2-27-hu(5)	-0.04	0.70	0.75, 0.72
K-7-gabor(3,0.3,var), T2W-3-zernike(20)	0.03	0.78	0.75, 0.52
K-7-gabor(2,0.4,absmean), T2-27-hu(1)	-0.03	0.79	0.77, 0.74
K-7-gabor(2,0.4,absmean), T2W-27-gabor(3,0.1,var)	-0.02	0.81	0.77, 0.73
K-7-gabor(2,0.4,mag), T2-27-hu(1)	-0.02	0.81	0.75, 0.74
K-7-gabor(3,0.3,absmean), T2W-27-gabor(1,0.1,var)	0.02	0.82	0.76, 0.71
K-7-gabor(2,0.4,mag), T2W-3-zernike(20)	-0.02	0.83	0.75, 0.52
K-7-gabor(3,0.3,absmean), T2-27-hu(5)	-0.02	0.83	0.76, 0.72
K-7-gabor(3,0.3,mag), T2W-3-zernike(20)	0.02	0.84	0.76, 0.52
K-7-gabor(3,0.3,absmean), T2W-27-gabor(2,0.1,var)	0.02	0.84	0.76, 0.71
K-7-gabor(3,0.3,var), T2W-27-gabor(1,0.1,var)	0.02	0.86	0.75, 0.71
K-7-gabor(3,0.3,var), T2W-27-gabor(2,0.1,var)	0.02	0.87	0.75, 0.71
K-7-gabor(3,0.3,mag), T2-27-hu(5)	-0.02	0.87	0.76, 0.72
K-7-gabor(2,0.4,absmean), T2W-3-zernike(20)	-0.02	0.87	0.77, 0.52
K-7-gabor(3,0.3,mag), T2W-27-gabor(1,0.1,var)	0.02	0.87	0.76, 0.71
K-7-gabor(3,0.3,var), T2-27-hu(1)	-0.02	0.88	0.75, 0.74
K-7-gabor(3,0.3,mag), T2W-27-gabor(2,0.1,var)	0.02	0.88	0.76, 0.71
K-7-gabor(3,0.3,absmean), T2W-3-zernike(20)	0.01	0.90	0.76, 0.52
K-7-gabor(3,0.3,absmean), T2-27-hu(1)	-0.01	0.91	0.76, 0.74
K-7-gabor(2,0.4,mag), T2W-27-gabor(2,0.1,var)	0.01	0.91	0.75, 0.71
K-7-gabor(2,0.4,mag), T2W-27-gabor(1,0.1,var)	0.01	0.91	0.75, 0.71
K-7-gabor(2,0.4,absmean), T2W-27-gabor(2,0.1,var)	-0.01	0.92	0.77, 0.71
K-7-gabor(2,0.4,absmean), T2W-27-gabor(1,0.1,var)	-0.01	0.93	0.77, 0.71
K-7-gabor(3,0.3,mag), T2-27-hu(1)	-0.01	0.94	0.76, 0.74
K-7-gabor(2,0.4,mag), T2W-27-gabor(3,0.1,var)	-0.01	0.95	0.75, 0.73
K-7-gabor(3,0.3,absmean), T2W-27-gabor(3,0.1,var)	0.01	0.96	0.76, 0.73
K-7-gabor(3,0.3,mag), T2W-27-gabor(3,0.1,var)	-0.00	0.98	0.76, 0.73
K-7-gabor(3,0.3,var), T2W-27-gabor(3,0.1,var)	-0.00	0.98	0.75, 0.73
K-7-gabor(3,0.3,var), T2-27-hu(5)	0.00	0.99	0.75, 0.72
T2-15-hu(2), T2W-3-zernike(20)	0.00	0.99	0.75, 0.52
T2-19-hu(2), T2W-NA-stats(min)	-0.00	0.99	0.72, 0.73
