# Supplementary figures and images for: Radiomics and machine learning of multisequence multiparametric prostate MRI: Towards improved non-invasive prostate cancer characterization (part 1 of 2)
Source: PLoS One. 2019 Jul 8;14(7):e0217702. doi: 10.1371/journal.pone.0217702 (PMC6613688; doi:10.1371/journal.pone.0217702)

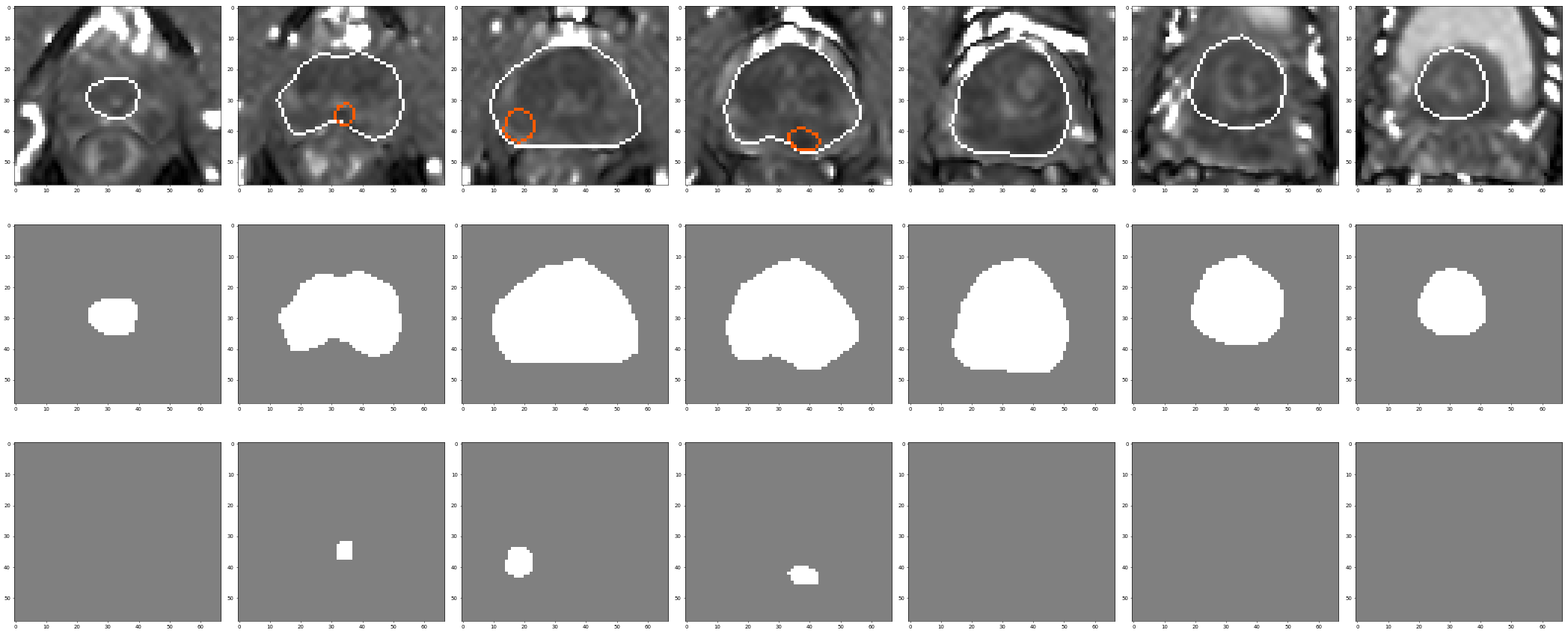

Supplement: S2 File — Files DWI-Mono-ADCm-xxx.png, T2-fitted-xxx.png, and T2w-std-xxx.png correspond to ADCm and T2 parametric maps, and T2-weighted images of each patient, respectively. On the first row of slices they show positions of regions of interest placed on the prostate cancer lesions (red, yellow) and around whole prostate (white). The prostate mask is on the second row, while the remaining rows are lesion masks. Files histology-xx.jpg contain the whole mount prostatectomy sections of each patient, with tumor outlines in green. Please note that identical MRI acquisition protocol has been used on all patients, including slice thickness. Here all prostate cancer masks are show with corresponding whole mount prostatectomy sections. (ZIP) [file pone.0217702.s002.zip › supporting_figures/DWI-Mono-ADCm-001.png]

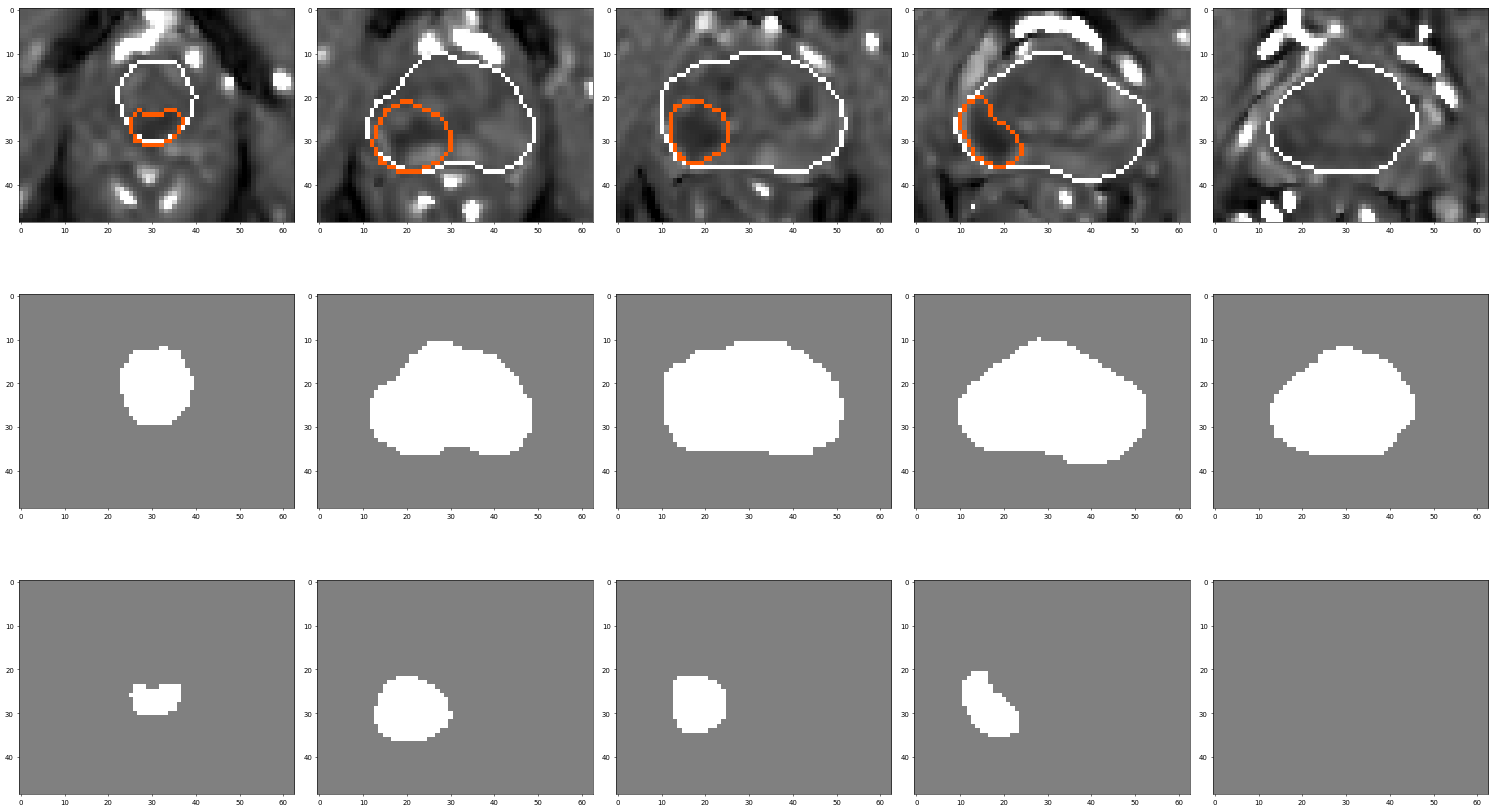

Supplement: S2 File — Files DWI-Mono-ADCm-xxx.png, T2-fitted-xxx.png, and T2w-std-xxx.png correspond to ADCm and T2 parametric maps, and T2-weighted images of each patient, respectively. On the first row of slices they show positions of regions of interest placed on the prostate cancer lesions (red, yellow) and around whole prostate (white). The prostate mask is on the second row, while the remaining rows are lesion masks. Files histology-xx.jpg contain the whole mount prostatectomy sections of each patient, with tumor outlines in green. Please note that identical MRI acquisition protocol has been used on all patients, including slice thickness. Here all prostate cancer masks are show with corresponding whole mount prostatectomy sections. (ZIP) [file pone.0217702.s002.zip › supporting_figures/DWI-Mono-ADCm-002.png]

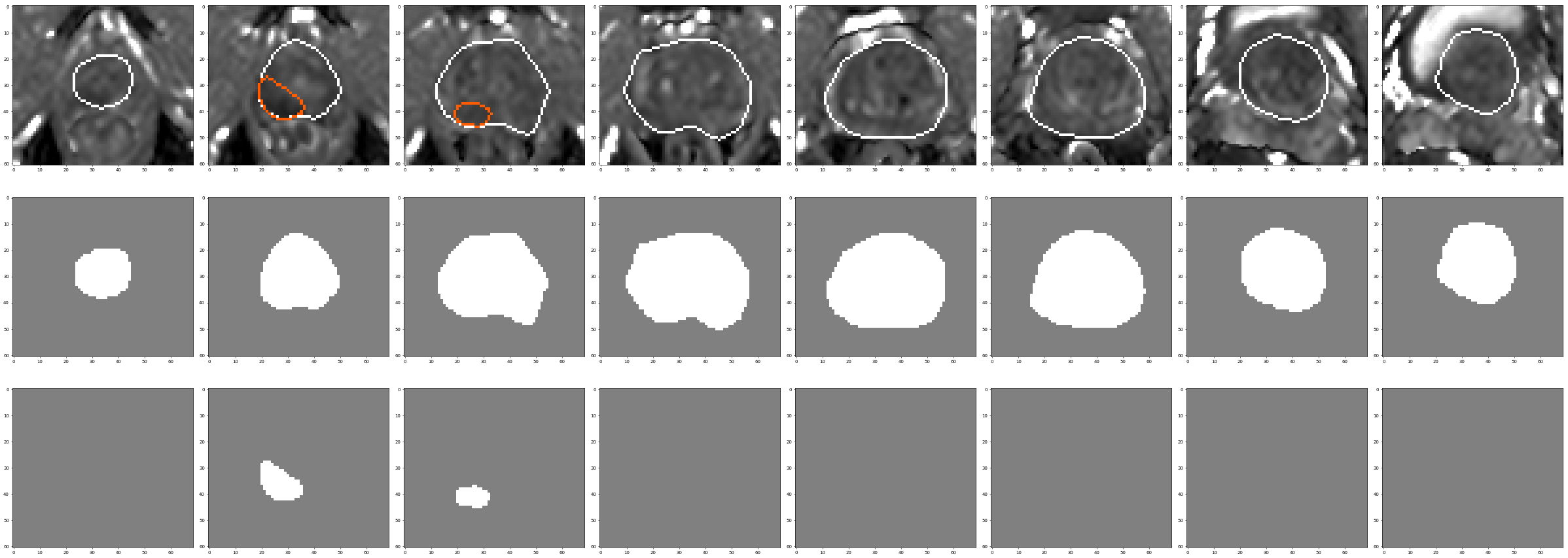

Supplement: S2 File — Files DWI-Mono-ADCm-xxx.png, T2-fitted-xxx.png, and T2w-std-xxx.png correspond to ADCm and T2 parametric maps, and T2-weighted images of each patient, respectively. On the first row of slices they show positions of regions of interest placed on the prostate cancer lesions (red, yellow) and around whole prostate (white). The prostate mask is on the second row, while the remaining rows are lesion masks. Files histology-xx.jpg contain the whole mount prostatectomy sections of each patient, with tumor outlines in green. Please note that identical MRI acquisition protocol has been used on all patients, including slice thickness. Here all prostate cancer masks are show with corresponding whole mount prostatectomy sections. (ZIP) [file pone.0217702.s002.zip › supporting_figures/DWI-Mono-ADCm-003.png]

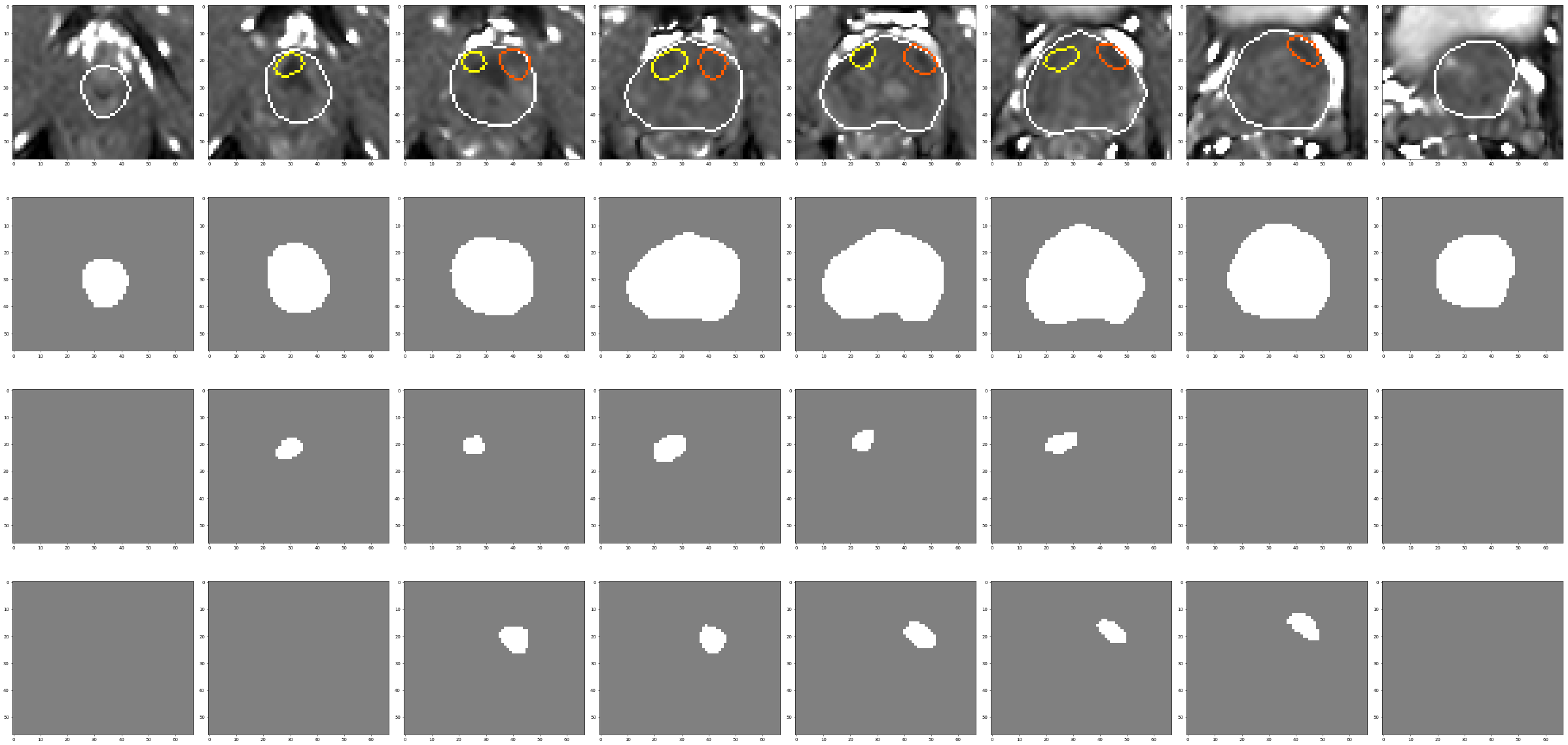

Supplement: S2 File — Files DWI-Mono-ADCm-xxx.png, T2-fitted-xxx.png, and T2w-std-xxx.png correspond to ADCm and T2 parametric maps, and T2-weighted images of each patient, respectively. On the first row of slices they show positions of regions of interest placed on the prostate cancer lesions (red, yellow) and around whole prostate (white). The prostate mask is on the second row, while the remaining rows are lesion masks. Files histology-xx.jpg contain the whole mount prostatectomy sections of each patient, with tumor outlines in green. Please note that identical MRI acquisition protocol has been used on all patients, including slice thickness. Here all prostate cancer masks are show with corresponding whole mount prostatectomy sections. (ZIP) [file pone.0217702.s002.zip › supporting_figures/DWI-Mono-ADCm-004.png]

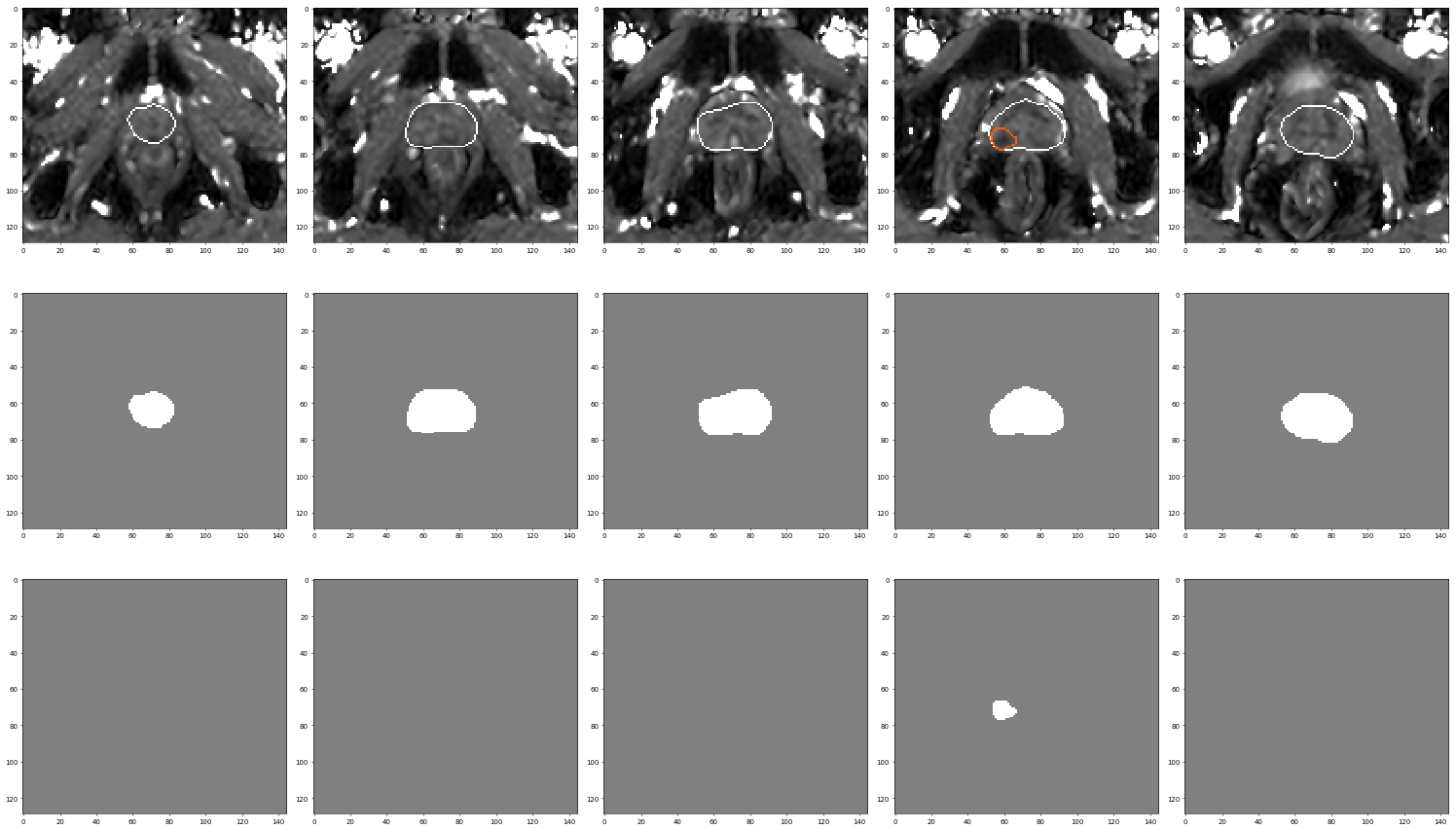

Supplement: S2 File — Files DWI-Mono-ADCm-xxx.png, T2-fitted-xxx.png, and T2w-std-xxx.png correspond to ADCm and T2 parametric maps, and T2-weighted images of each patient, respectively. On the first row of slices they show positions of regions of interest placed on the prostate cancer lesions (red, yellow) and around whole prostate (white). The prostate mask is on the second row, while the remaining rows are lesion masks. Files histology-xx.jpg contain the whole mount prostatectomy sections of each patient, with tumor outlines in green. Please note that identical MRI acquisition protocol has been used on all patients, including slice thickness. Here all prostate cancer masks are show with corresponding whole mount prostatectomy sections. (ZIP) [file pone.0217702.s002.zip › supporting_figures/DWI-Mono-ADCm-005.png]

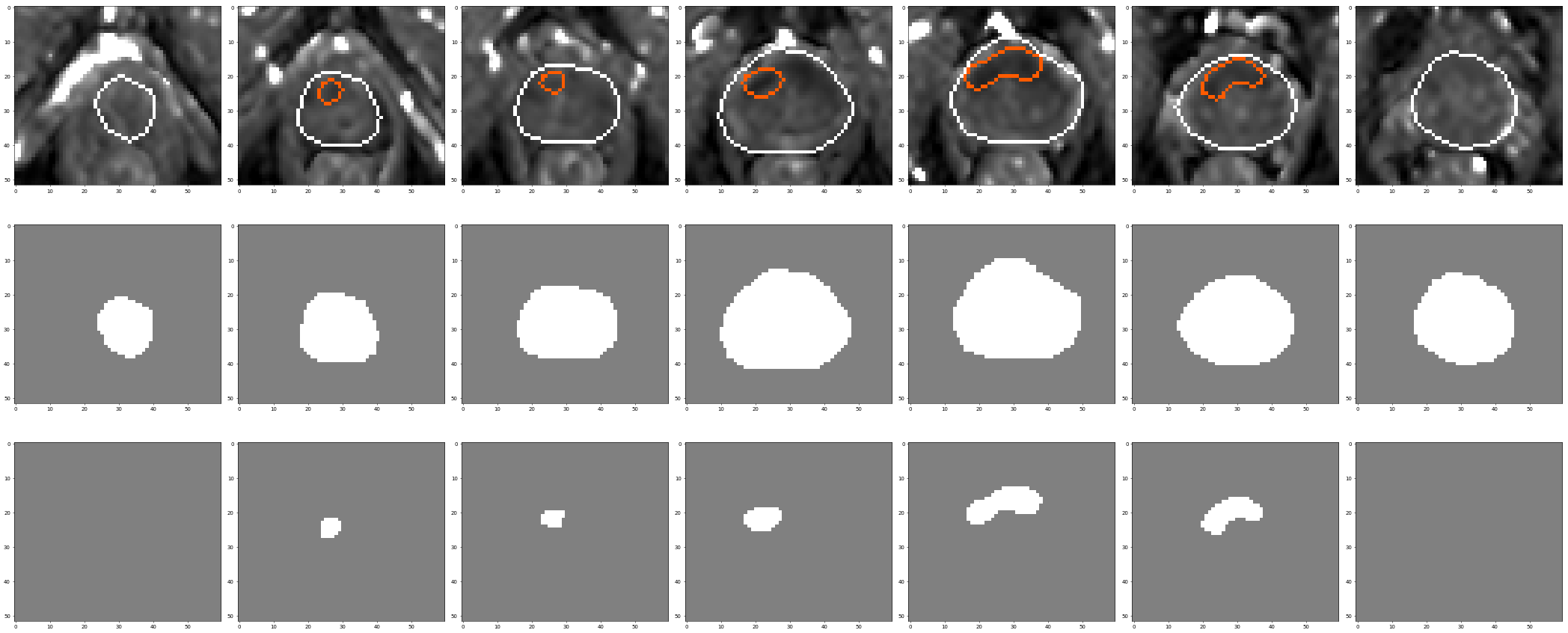

Supplement: S2 File — Files DWI-Mono-ADCm-xxx.png, T2-fitted-xxx.png, and T2w-std-xxx.png correspond to ADCm and T2 parametric maps, and T2-weighted images of each patient, respectively. On the first row of slices they show positions of regions of interest placed on the prostate cancer lesions (red, yellow) and around whole prostate (white). The prostate mask is on the second row, while the remaining rows are lesion masks. Files histology-xx.jpg contain the whole mount prostatectomy sections of each patient, with tumor outlines in green. Please note that identical MRI acquisition protocol has been used on all patients, including slice thickness. Here all prostate cancer masks are show with corresponding whole mount prostatectomy sections. (ZIP) [file pone.0217702.s002.zip › supporting_figures/DWI-Mono-ADCm-006.png]

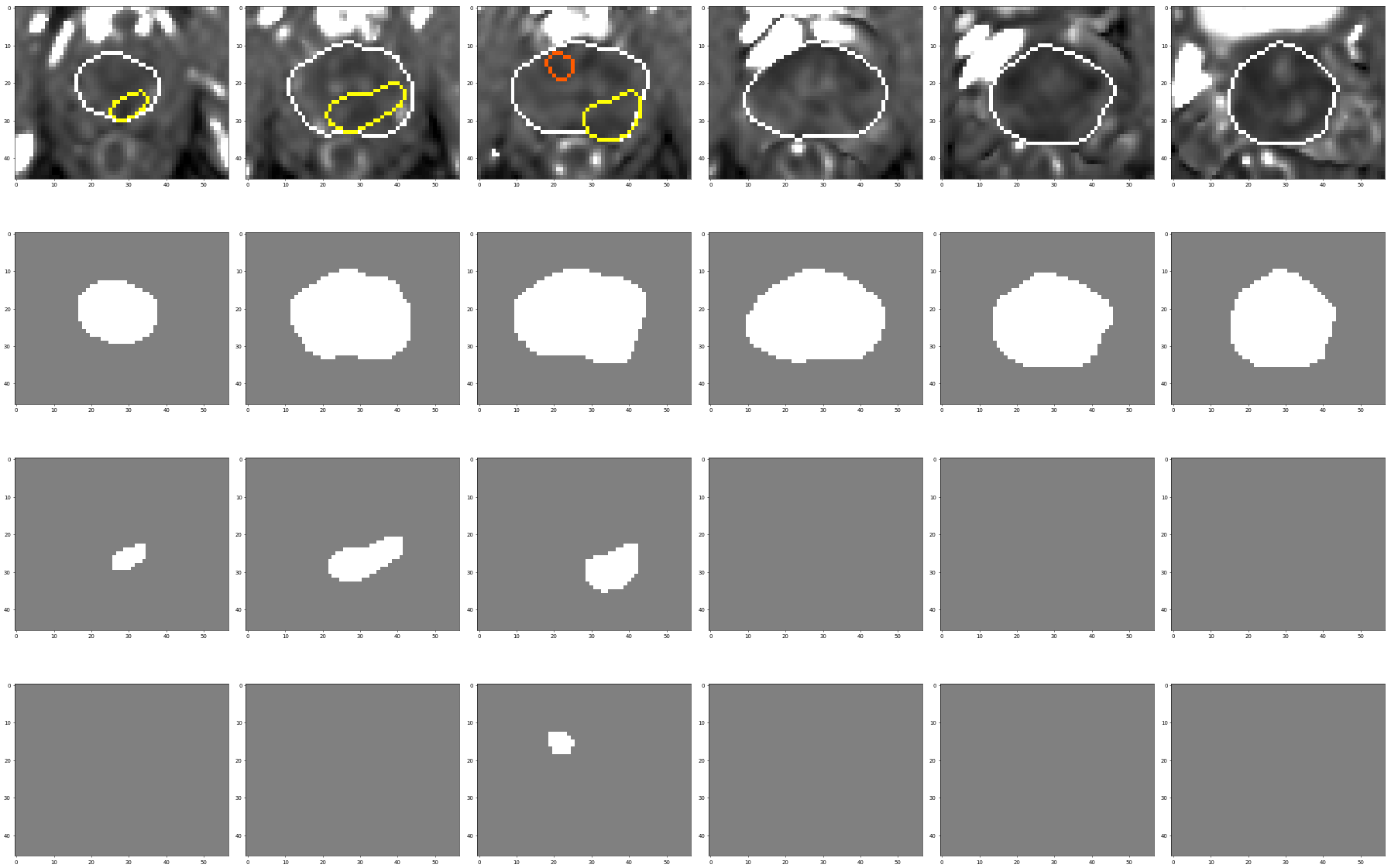

Supplement: S2 File — Files DWI-Mono-ADCm-xxx.png, T2-fitted-xxx.png, and T2w-std-xxx.png correspond to ADCm and T2 parametric maps, and T2-weighted images of each patient, respectively. On the first row of slices they show positions of regions of interest placed on the prostate cancer lesions (red, yellow) and around whole prostate (white). The prostate mask is on the second row, while the remaining rows are lesion masks. Files histology-xx.jpg contain the whole mount prostatectomy sections of each patient, with tumor outlines in green. Please note that identical MRI acquisition protocol has been used on all patients, including slice thickness. Here all prostate cancer masks are show with corresponding whole mount prostatectomy sections. (ZIP) [file pone.0217702.s002.zip › supporting_figures/DWI-Mono-ADCm-007.png]

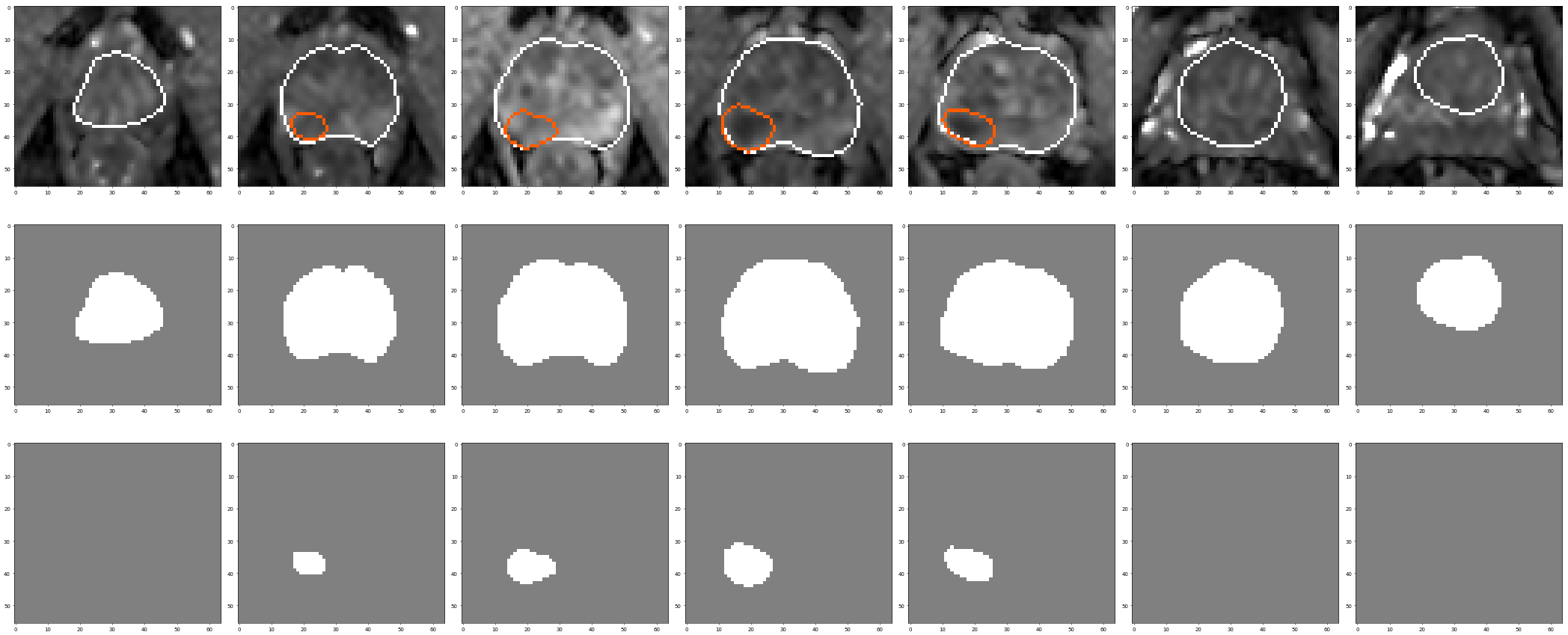

Supplement: S2 File — Files DWI-Mono-ADCm-xxx.png, T2-fitted-xxx.png, and T2w-std-xxx.png correspond to ADCm and T2 parametric maps, and T2-weighted images of each patient, respectively. On the first row of slices they show positions of regions of interest placed on the prostate cancer lesions (red, yellow) and around whole prostate (white). The prostate mask is on the second row, while the remaining rows are lesion masks. Files histology-xx.jpg contain the whole mount prostatectomy sections of each patient, with tumor outlines in green. Please note that identical MRI acquisition protocol has been used on all patients, including slice thickness. Here all prostate cancer masks are show with corresponding whole mount prostatectomy sections. (ZIP) [file pone.0217702.s002.zip › supporting_figures/DWI-Mono-ADCm-008.png]

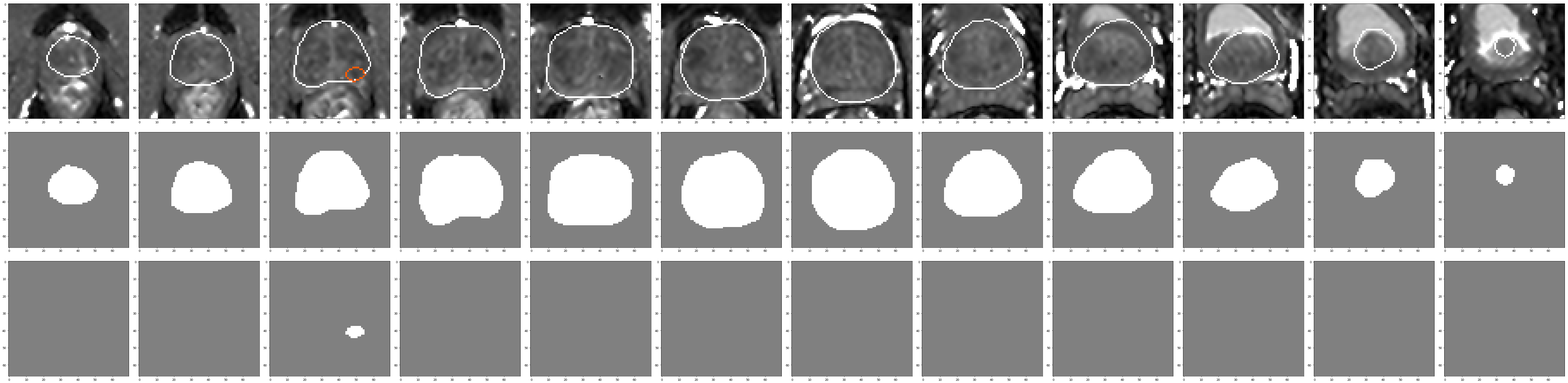

Supplement: S2 File — Files DWI-Mono-ADCm-xxx.png, T2-fitted-xxx.png, and T2w-std-xxx.png correspond to ADCm and T2 parametric maps, and T2-weighted images of each patient, respectively. On the first row of slices they show positions of regions of interest placed on the prostate cancer lesions (red, yellow) and around whole prostate (white). The prostate mask is on the second row, while the remaining rows are lesion masks. Files histology-xx.jpg contain the whole mount prostatectomy sections of each patient, with tumor outlines in green. Please note that identical MRI acquisition protocol has been used on all patients, including slice thickness. Here all prostate cancer masks are show with corresponding whole mount prostatectomy sections. (ZIP) [file pone.0217702.s002.zip › supporting_figures/DWI-Mono-ADCm-009.png]

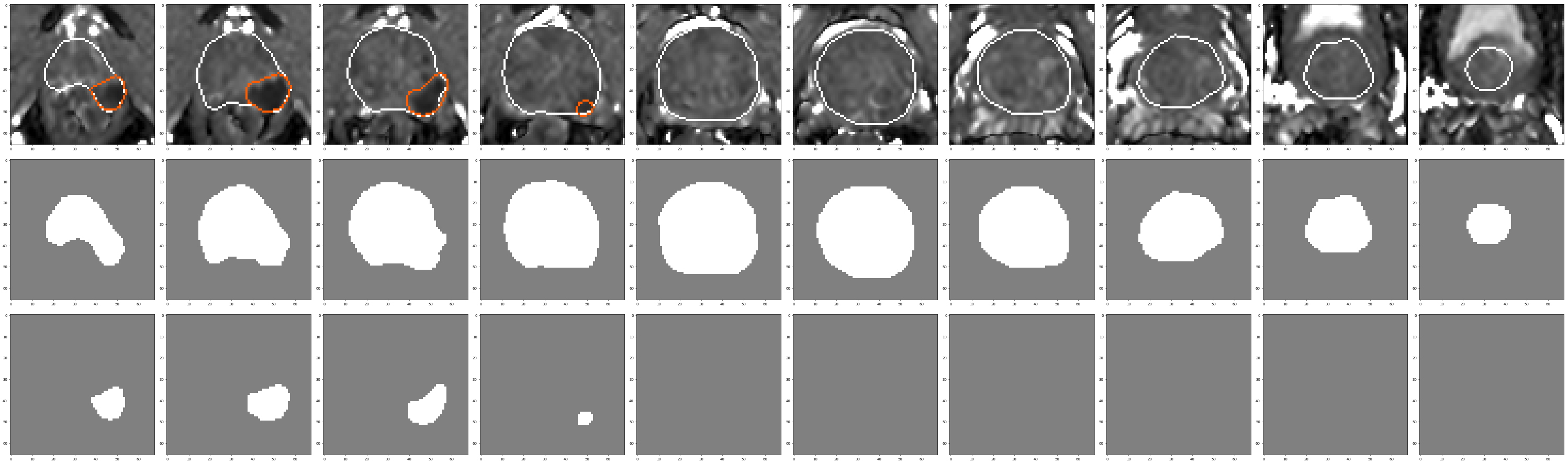

Supplement: S2 File — Files DWI-Mono-ADCm-xxx.png, T2-fitted-xxx.png, and T2w-std-xxx.png correspond to ADCm and T2 parametric maps, and T2-weighted images of each patient, respectively. On the first row of slices they show positions of regions of interest placed on the prostate cancer lesions (red, yellow) and around whole prostate (white). The prostate mask is on the second row, while the remaining rows are lesion masks. Files histology-xx.jpg contain the whole mount prostatectomy sections of each patient, with tumor outlines in green. Please note that identical MRI acquisition protocol has been used on all patients, including slice thickness. Here all prostate cancer masks are show with corresponding whole mount prostatectomy sections. (ZIP) [file pone.0217702.s002.zip › supporting_figures/DWI-Mono-ADCm-010.png]

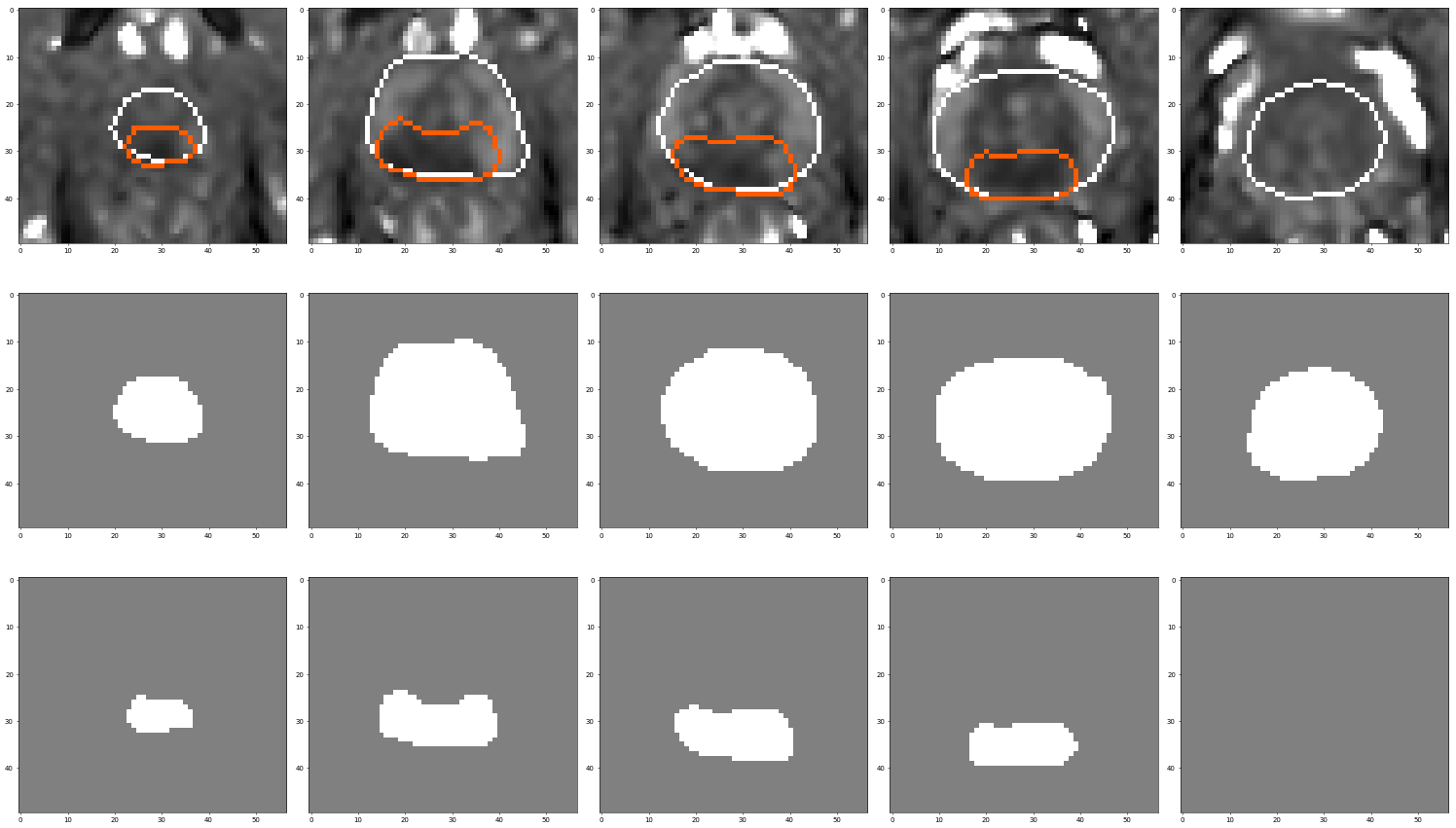

Supplement: S2 File — Files DWI-Mono-ADCm-xxx.png, T2-fitted-xxx.png, and T2w-std-xxx.png correspond to ADCm and T2 parametric maps, and T2-weighted images of each patient, respectively. On the first row of slices they show positions of regions of interest placed on the prostate cancer lesions (red, yellow) and around whole prostate (white). The prostate mask is on the second row, while the remaining rows are lesion masks. Files histology-xx.jpg contain the whole mount prostatectomy sections of each patient, with tumor outlines in green. Please note that identical MRI acquisition protocol has been used on all patients, including slice thickness. Here all prostate cancer masks are show with corresponding whole mount prostatectomy sections. (ZIP) [file pone.0217702.s002.zip › supporting_figures/DWI-Mono-ADCm-011.png]

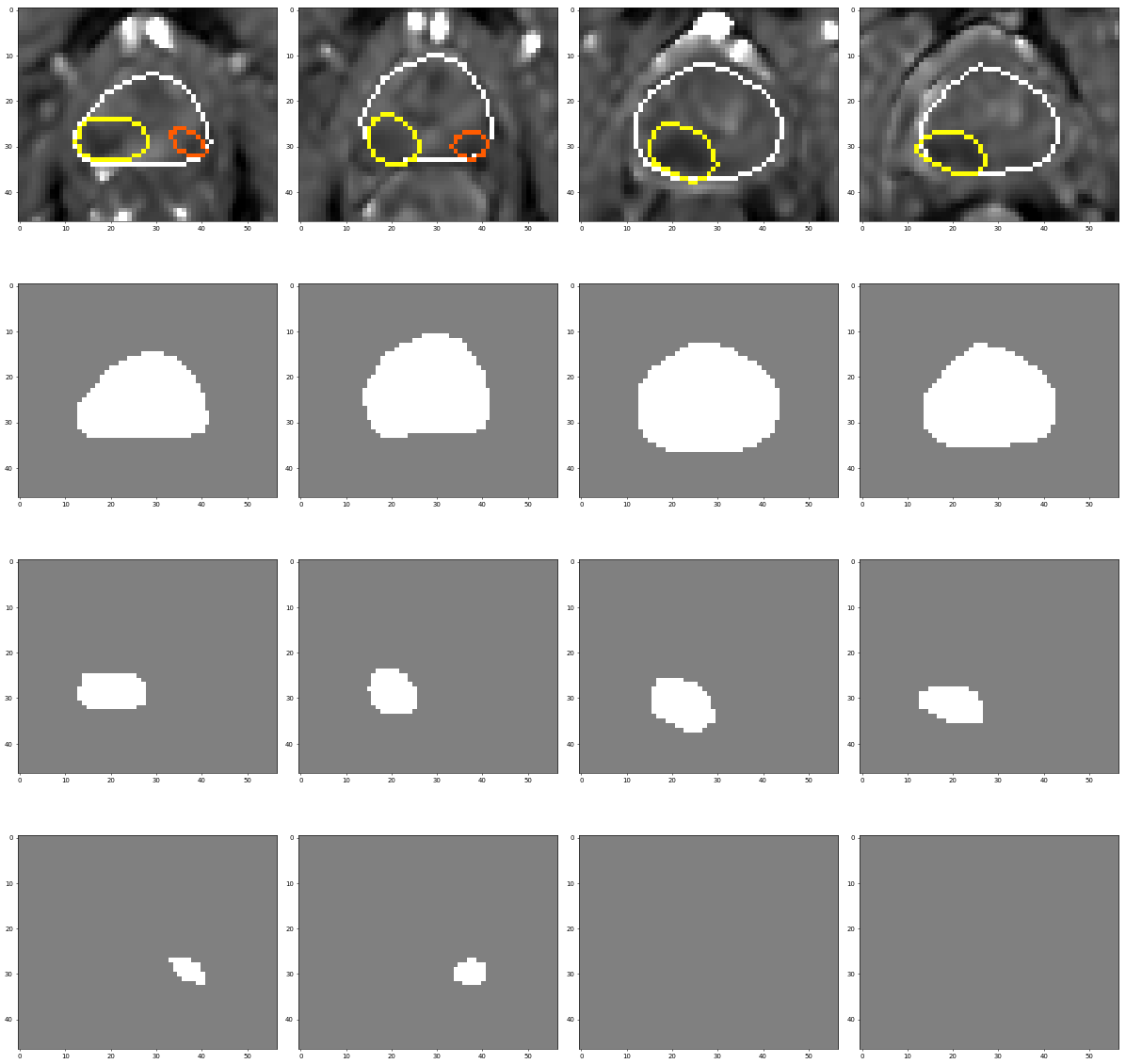

Supplement: S2 File — Files DWI-Mono-ADCm-xxx.png, T2-fitted-xxx.png, and T2w-std-xxx.png correspond to ADCm and T2 parametric maps, and T2-weighted images of each patient, respectively. On the first row of slices they show positions of regions of interest placed on the prostate cancer lesions (red, yellow) and around whole prostate (white). The prostate mask is on the second row, while the remaining rows are lesion masks. Files histology-xx.jpg contain the whole mount prostatectomy sections of each patient, with tumor outlines in green. Please note that identical MRI acquisition protocol has been used on all patients, including slice thickness. Here all prostate cancer masks are show with corresponding whole mount prostatectomy sections. (ZIP) [file pone.0217702.s002.zip › supporting_figures/DWI-Mono-ADCm-012.png]

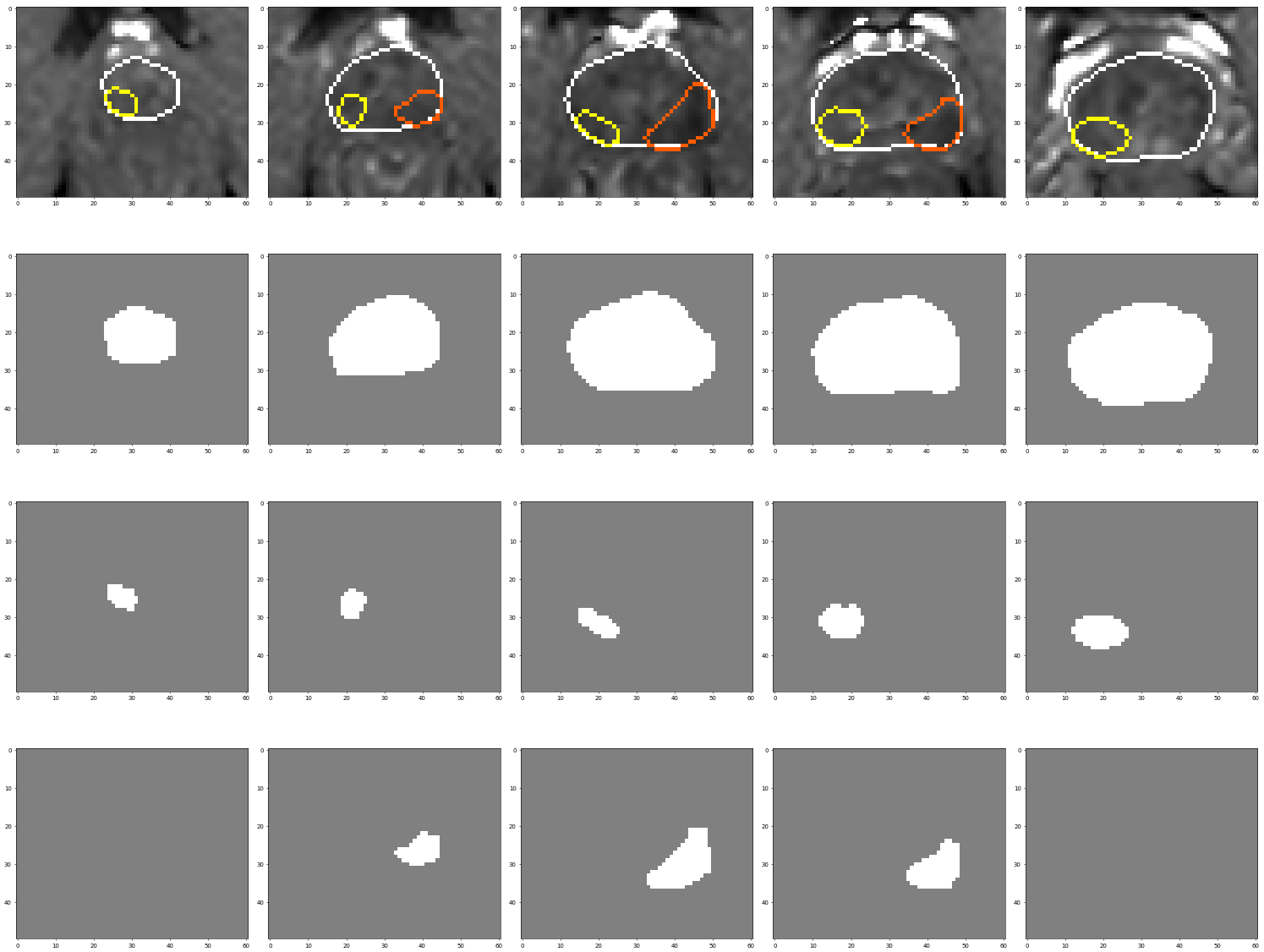

Supplement: S2 File — Files DWI-Mono-ADCm-xxx.png, T2-fitted-xxx.png, and T2w-std-xxx.png correspond to ADCm and T2 parametric maps, and T2-weighted images of each patient, respectively. On the first row of slices they show positions of regions of interest placed on the prostate cancer lesions (red, yellow) and around whole prostate (white). The prostate mask is on the second row, while the remaining rows are lesion masks. Files histology-xx.jpg contain the whole mount prostatectomy sections of each patient, with tumor outlines in green. Please note that identical MRI acquisition protocol has been used on all patients, including slice thickness. Here all prostate cancer masks are show with corresponding whole mount prostatectomy sections. (ZIP) [file pone.0217702.s002.zip › supporting_figures/DWI-Mono-ADCm-013.png]

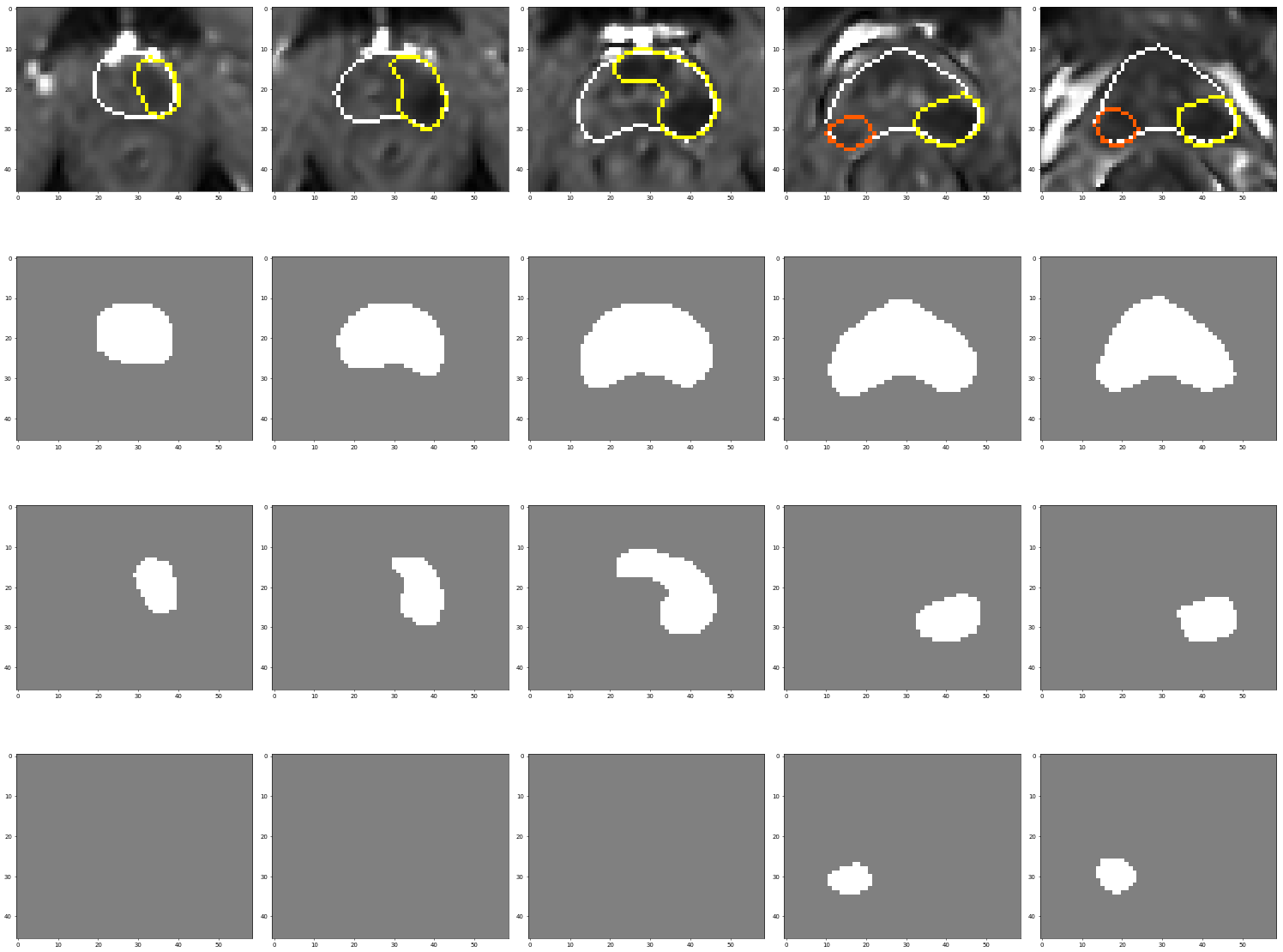

Supplement: S2 File — Files DWI-Mono-ADCm-xxx.png, T2-fitted-xxx.png, and T2w-std-xxx.png correspond to ADCm and T2 parametric maps, and T2-weighted images of each patient, respectively. On the first row of slices they show positions of regions of interest placed on the prostate cancer lesions (red, yellow) and around whole prostate (white). The prostate mask is on the second row, while the remaining rows are lesion masks. Files histology-xx.jpg contain the whole mount prostatectomy sections of each patient, with tumor outlines in green. Please note that identical MRI acquisition protocol has been used on all patients, including slice thickness. Here all prostate cancer masks are show with corresponding whole mount prostatectomy sections. (ZIP) [file pone.0217702.s002.zip › supporting_figures/DWI-Mono-ADCm-014.png]

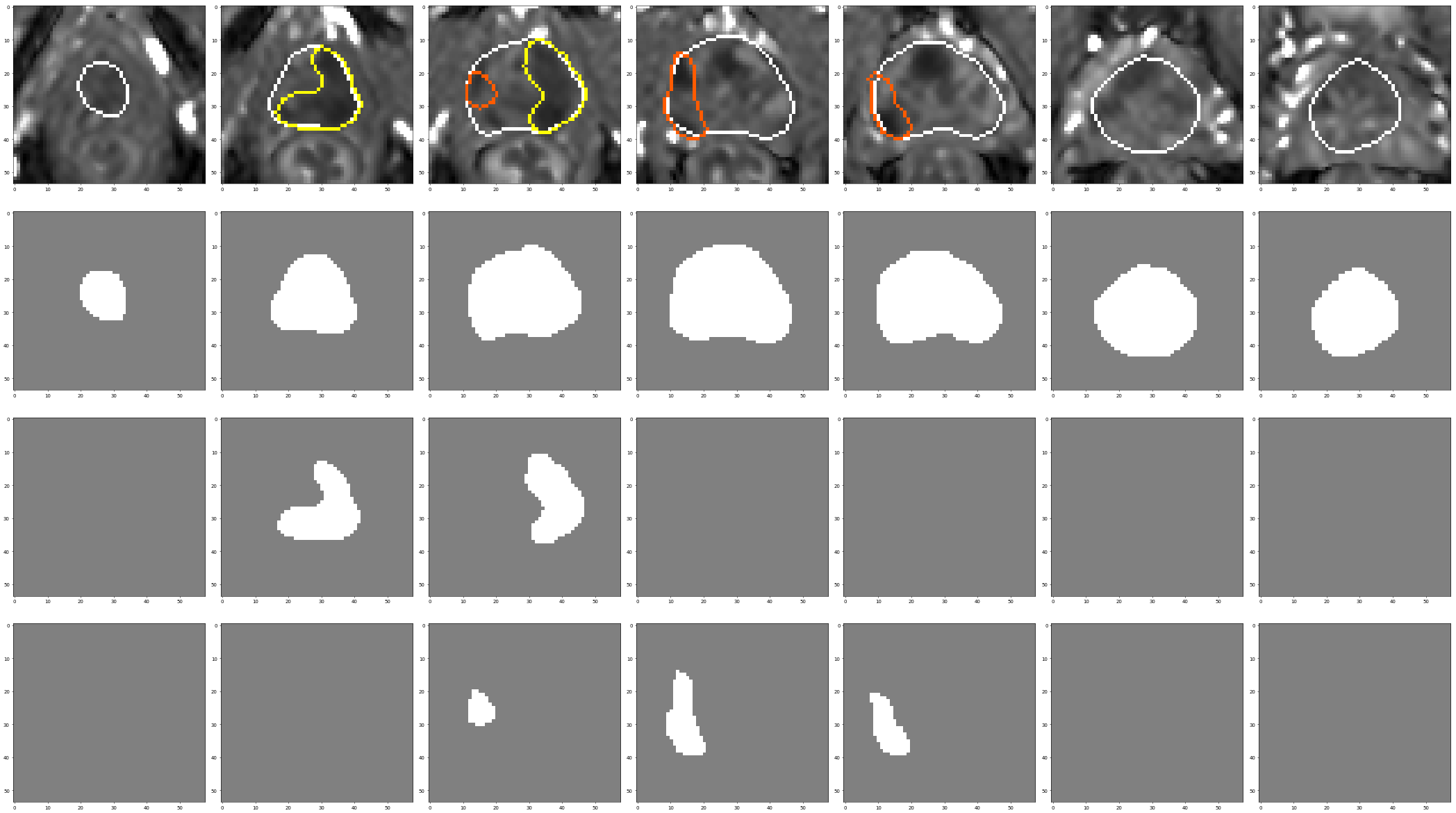

Supplement: S2 File — Files DWI-Mono-ADCm-xxx.png, T2-fitted-xxx.png, and T2w-std-xxx.png correspond to ADCm and T2 parametric maps, and T2-weighted images of each patient, respectively. On the first row of slices they show positions of regions of interest placed on the prostate cancer lesions (red, yellow) and around whole prostate (white). The prostate mask is on the second row, while the remaining rows are lesion masks. Files histology-xx.jpg contain the whole mount prostatectomy sections of each patient, with tumor outlines in green. Please note that identical MRI acquisition protocol has been used on all patients, including slice thickness. Here all prostate cancer masks are show with corresponding whole mount prostatectomy sections. (ZIP) [file pone.0217702.s002.zip › supporting_figures/DWI-Mono-ADCm-015.png]

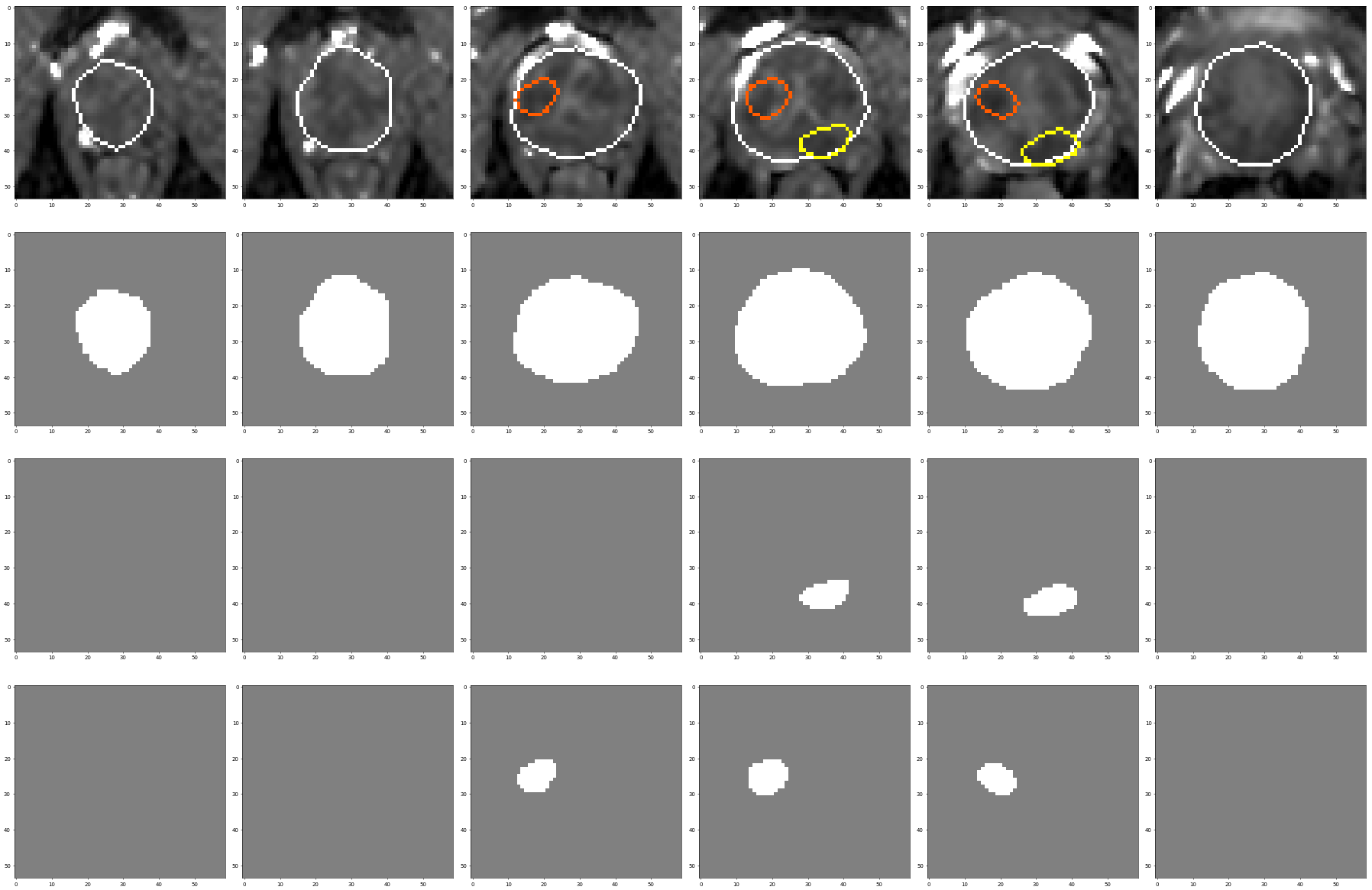

Supplement: S2 File — Files DWI-Mono-ADCm-xxx.png, T2-fitted-xxx.png, and T2w-std-xxx.png correspond to ADCm and T2 parametric maps, and T2-weighted images of each patient, respectively. On the first row of slices they show positions of regions of interest placed on the prostate cancer lesions (red, yellow) and around whole prostate (white). The prostate mask is on the second row, while the remaining rows are lesion masks. Files histology-xx.jpg contain the whole mount prostatectomy sections of each patient, with tumor outlines in green. Please note that identical MRI acquisition protocol has been used on all patients, including slice thickness. Here all prostate cancer masks are show with corresponding whole mount prostatectomy sections. (ZIP) [file pone.0217702.s002.zip › supporting_figures/DWI-Mono-ADCm-016.png]

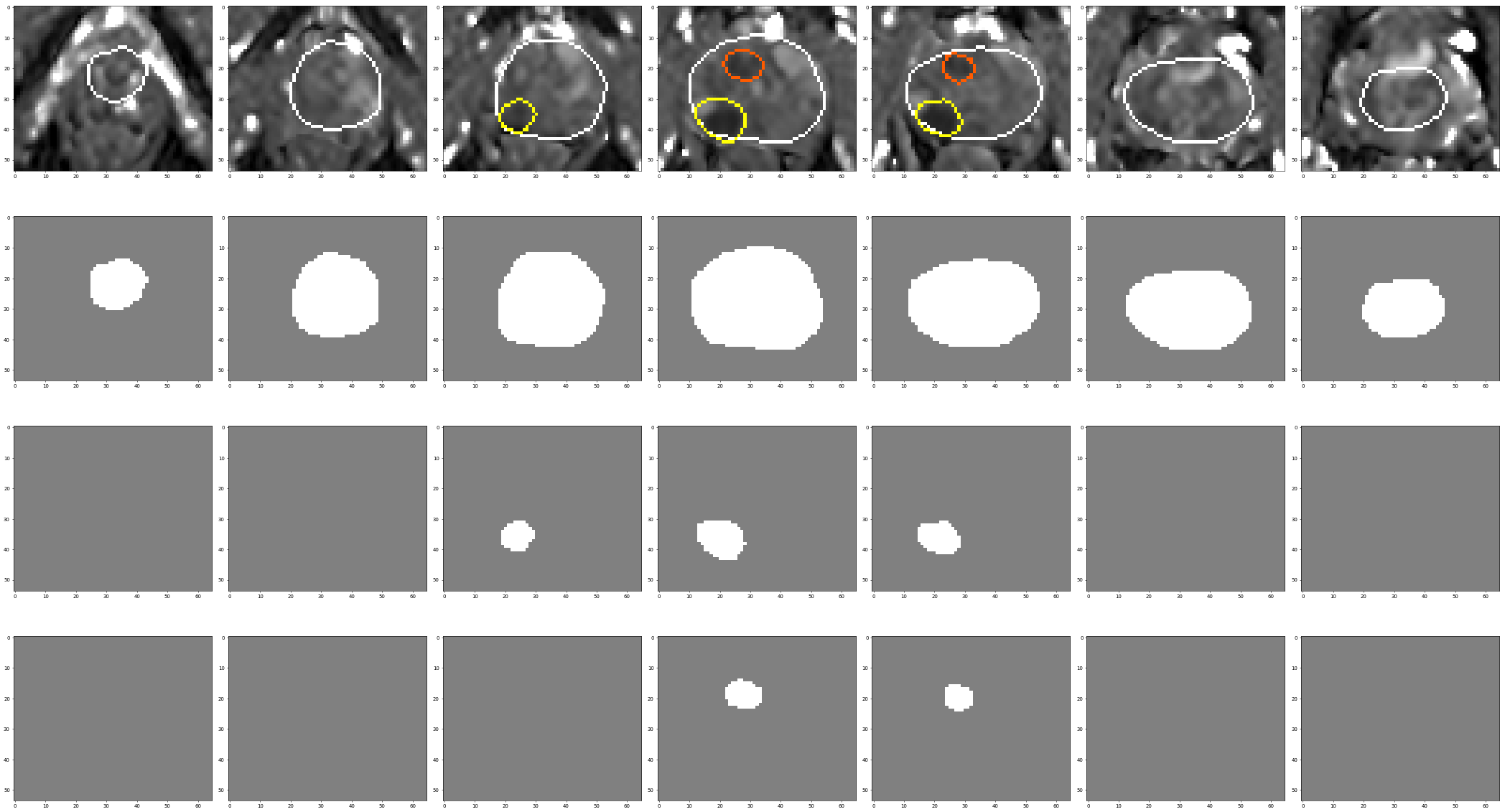

Supplement: S2 File — Files DWI-Mono-ADCm-xxx.png, T2-fitted-xxx.png, and T2w-std-xxx.png correspond to ADCm and T2 parametric maps, and T2-weighted images of each patient, respectively. On the first row of slices they show positions of regions of interest placed on the prostate cancer lesions (red, yellow) and around whole prostate (white). The prostate mask is on the second row, while the remaining rows are lesion masks. Files histology-xx.jpg contain the whole mount prostatectomy sections of each patient, with tumor outlines in green. Please note that identical MRI acquisition protocol has been used on all patients, including slice thickness. Here all prostate cancer masks are show with corresponding whole mount prostatectomy sections. (ZIP) [file pone.0217702.s002.zip › supporting_figures/DWI-Mono-ADCm-017.png]

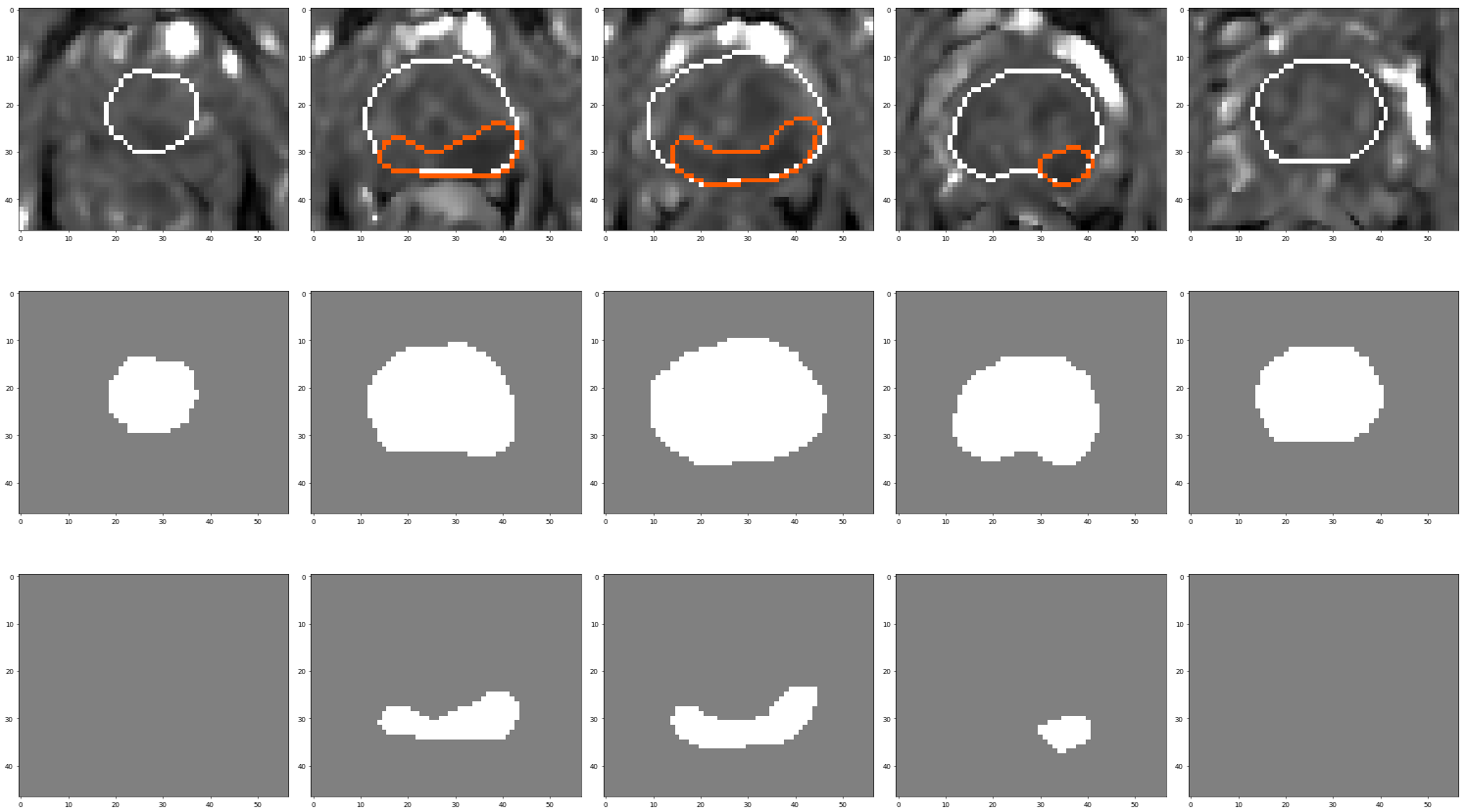

Supplement: S2 File — Files DWI-Mono-ADCm-xxx.png, T2-fitted-xxx.png, and T2w-std-xxx.png correspond to ADCm and T2 parametric maps, and T2-weighted images of each patient, respectively. On the first row of slices they show positions of regions of interest placed on the prostate cancer lesions (red, yellow) and around whole prostate (white). The prostate mask is on the second row, while the remaining rows are lesion masks. Files histology-xx.jpg contain the whole mount prostatectomy sections of each patient, with tumor outlines in green. Please note that identical MRI acquisition protocol has been used on all patients, including slice thickness. Here all prostate cancer masks are show with corresponding whole mount prostatectomy sections. (ZIP) [file pone.0217702.s002.zip › supporting_figures/DWI-Mono-ADCm-018.png]

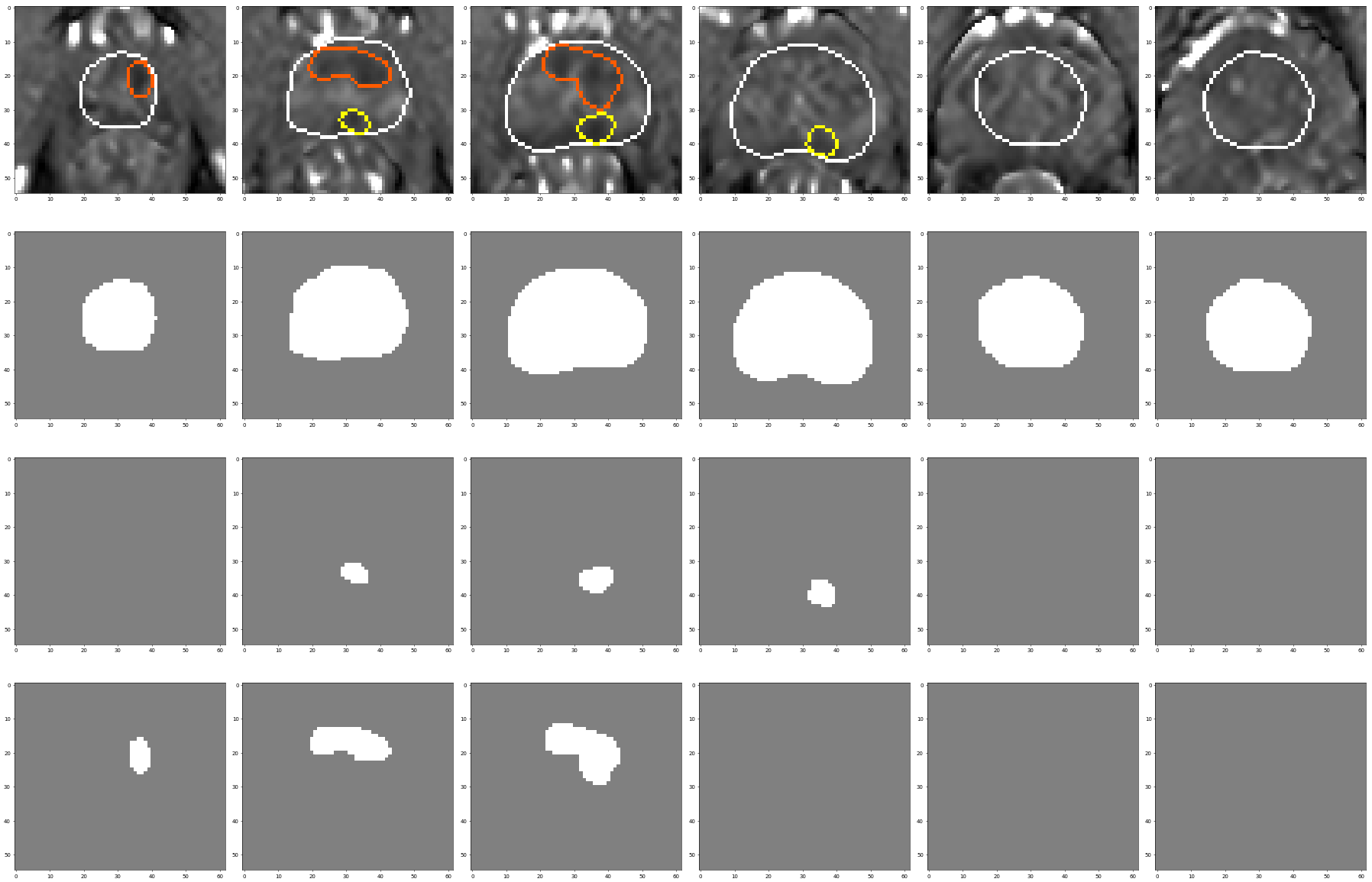

Supplement: S2 File — Files DWI-Mono-ADCm-xxx.png, T2-fitted-xxx.png, and T2w-std-xxx.png correspond to ADCm and T2 parametric maps, and T2-weighted images of each patient, respectively. On the first row of slices they show positions of regions of interest placed on the prostate cancer lesions (red, yellow) and around whole prostate (white). The prostate mask is on the second row, while the remaining rows are lesion masks. Files histology-xx.jpg contain the whole mount prostatectomy sections of each patient, with tumor outlines in green. Please note that identical MRI acquisition protocol has been used on all patients, including slice thickness. Here all prostate cancer masks are show with corresponding whole mount prostatectomy sections. (ZIP) [file pone.0217702.s002.zip › supporting_figures/DWI-Mono-ADCm-019.png]

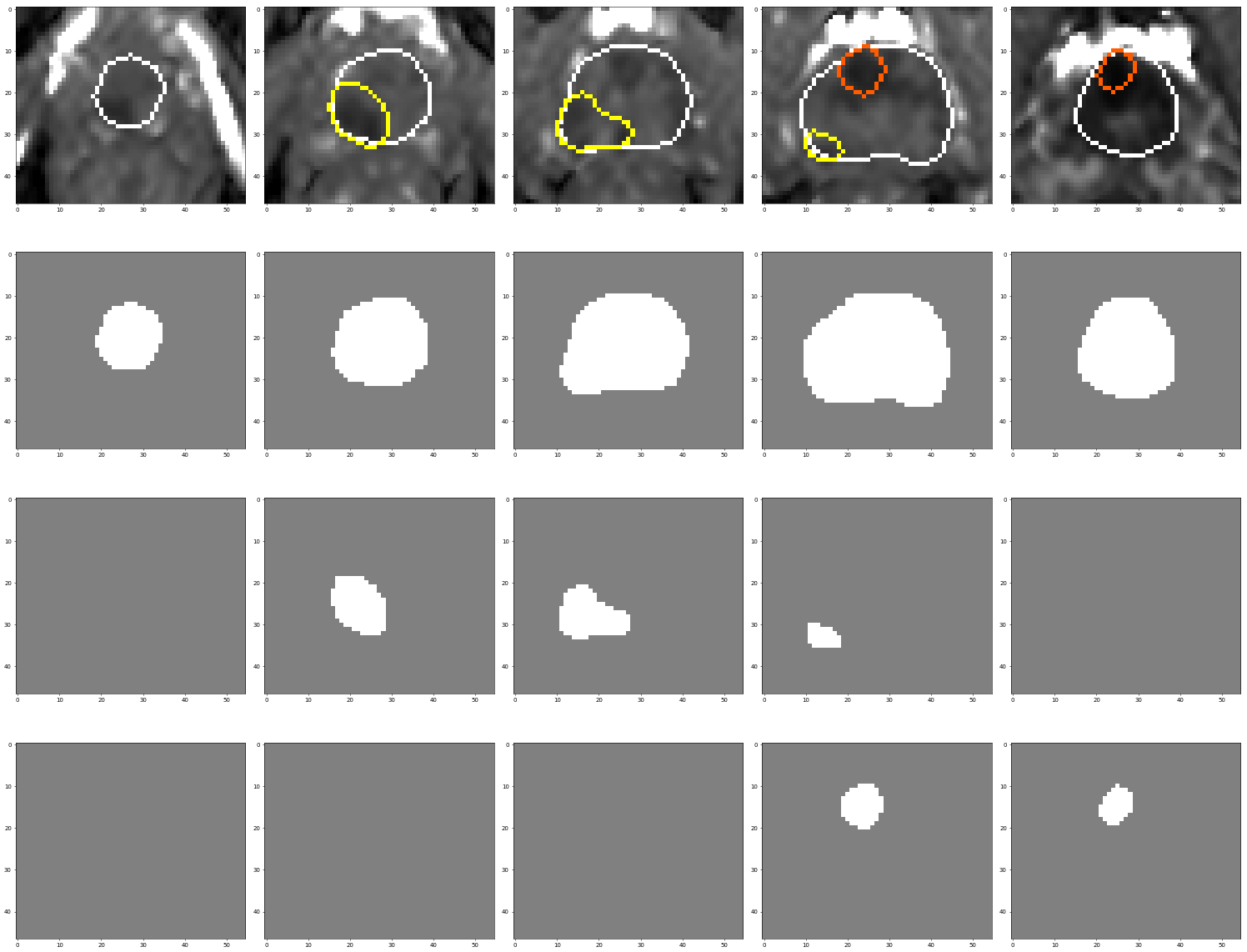

Supplement: S2 File — Files DWI-Mono-ADCm-xxx.png, T2-fitted-xxx.png, and T2w-std-xxx.png correspond to ADCm and T2 parametric maps, and T2-weighted images of each patient, respectively. On the first row of slices they show positions of regions of interest placed on the prostate cancer lesions (red, yellow) and around whole prostate (white). The prostate mask is on the second row, while the remaining rows are lesion masks. Files histology-xx.jpg contain the whole mount prostatectomy sections of each patient, with tumor outlines in green. Please note that identical MRI acquisition protocol has been used on all patients, including slice thickness. Here all prostate cancer masks are show with corresponding whole mount prostatectomy sections. (ZIP) [file pone.0217702.s002.zip › supporting_figures/DWI-Mono-ADCm-020.png]

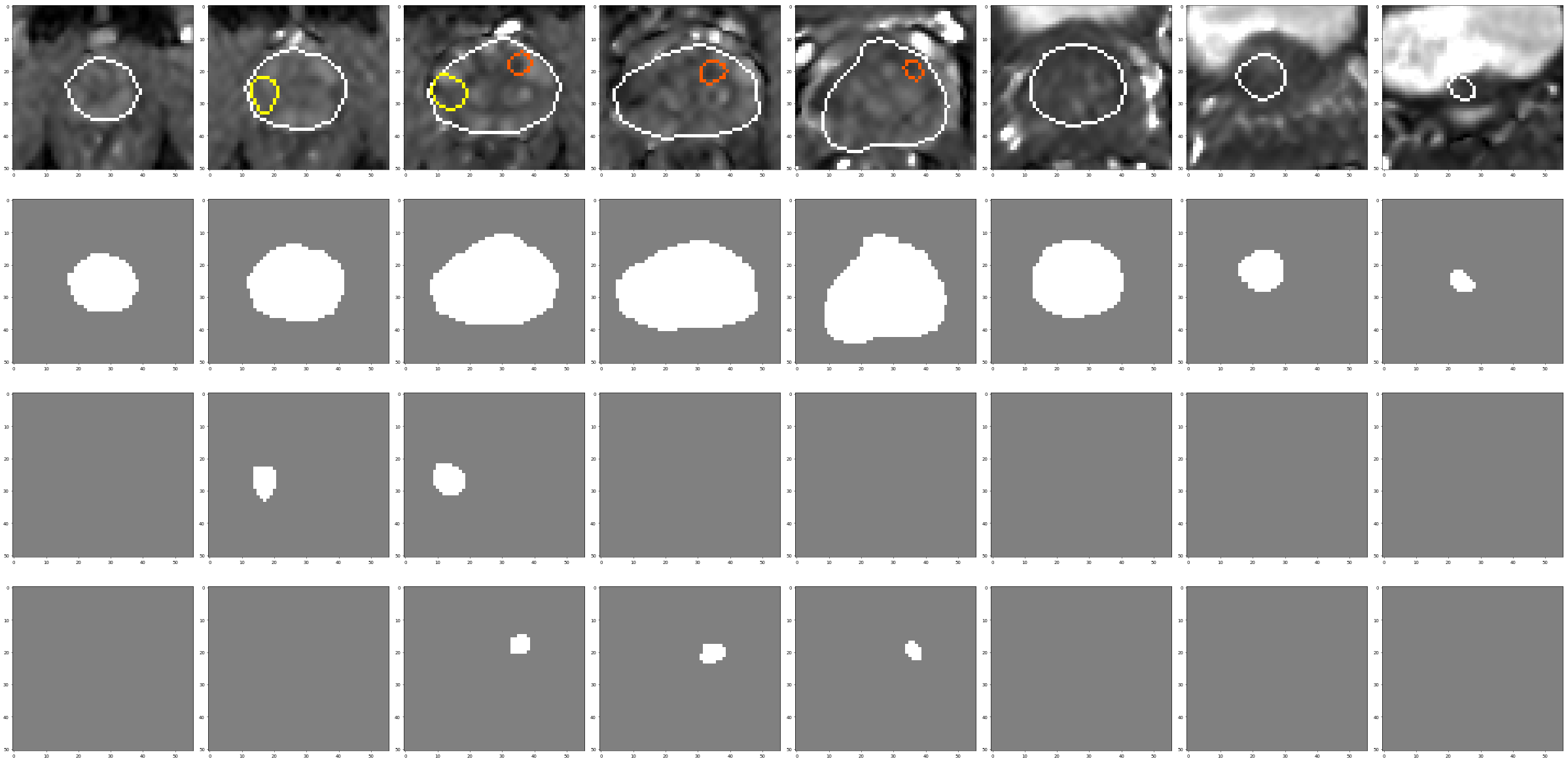

Supplement: S2 File — Files DWI-Mono-ADCm-xxx.png, T2-fitted-xxx.png, and T2w-std-xxx.png correspond to ADCm and T2 parametric maps, and T2-weighted images of each patient, respectively. On the first row of slices they show positions of regions of interest placed on the prostate cancer lesions (red, yellow) and around whole prostate (white). The prostate mask is on the second row, while the remaining rows are lesion masks. Files histology-xx.jpg contain the whole mount prostatectomy sections of each patient, with tumor outlines in green. Please note that identical MRI acquisition protocol has been used on all patients, including slice thickness. Here all prostate cancer masks are show with corresponding whole mount prostatectomy sections. (ZIP) [file pone.0217702.s002.zip › supporting_figures/DWI-Mono-ADCm-021.png]

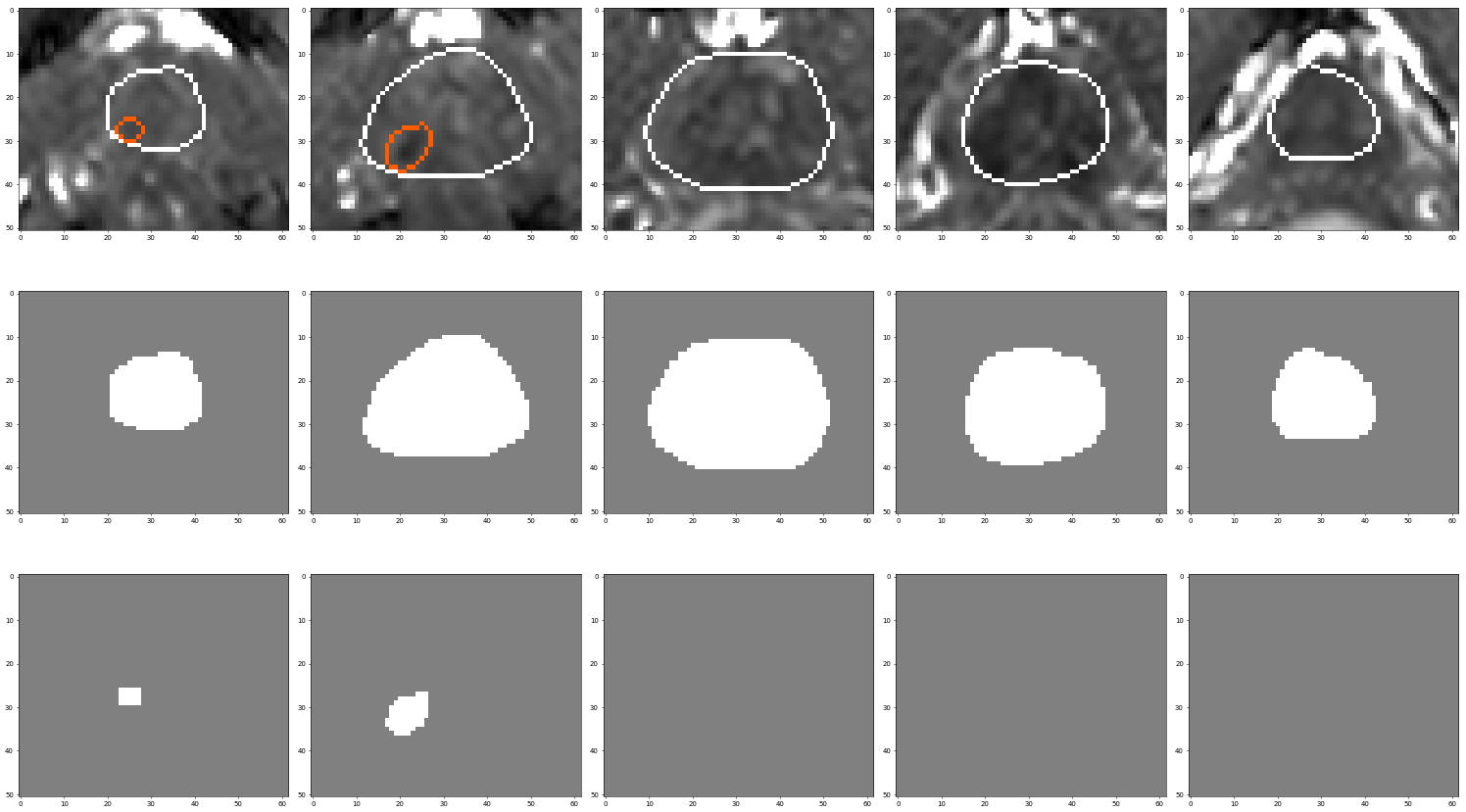

Supplement: S2 File — Files DWI-Mono-ADCm-xxx.png, T2-fitted-xxx.png, and T2w-std-xxx.png correspond to ADCm and T2 parametric maps, and T2-weighted images of each patient, respectively. On the first row of slices they show positions of regions of interest placed on the prostate cancer lesions (red, yellow) and around whole prostate (white). The prostate mask is on the second row, while the remaining rows are lesion masks. Files histology-xx.jpg contain the whole mount prostatectomy sections of each patient, with tumor outlines in green. Please note that identical MRI acquisition protocol has been used on all patients, including slice thickness. Here all prostate cancer masks are show with corresponding whole mount prostatectomy sections. (ZIP) [file pone.0217702.s002.zip › supporting_figures/DWI-Mono-ADCm-022.png]

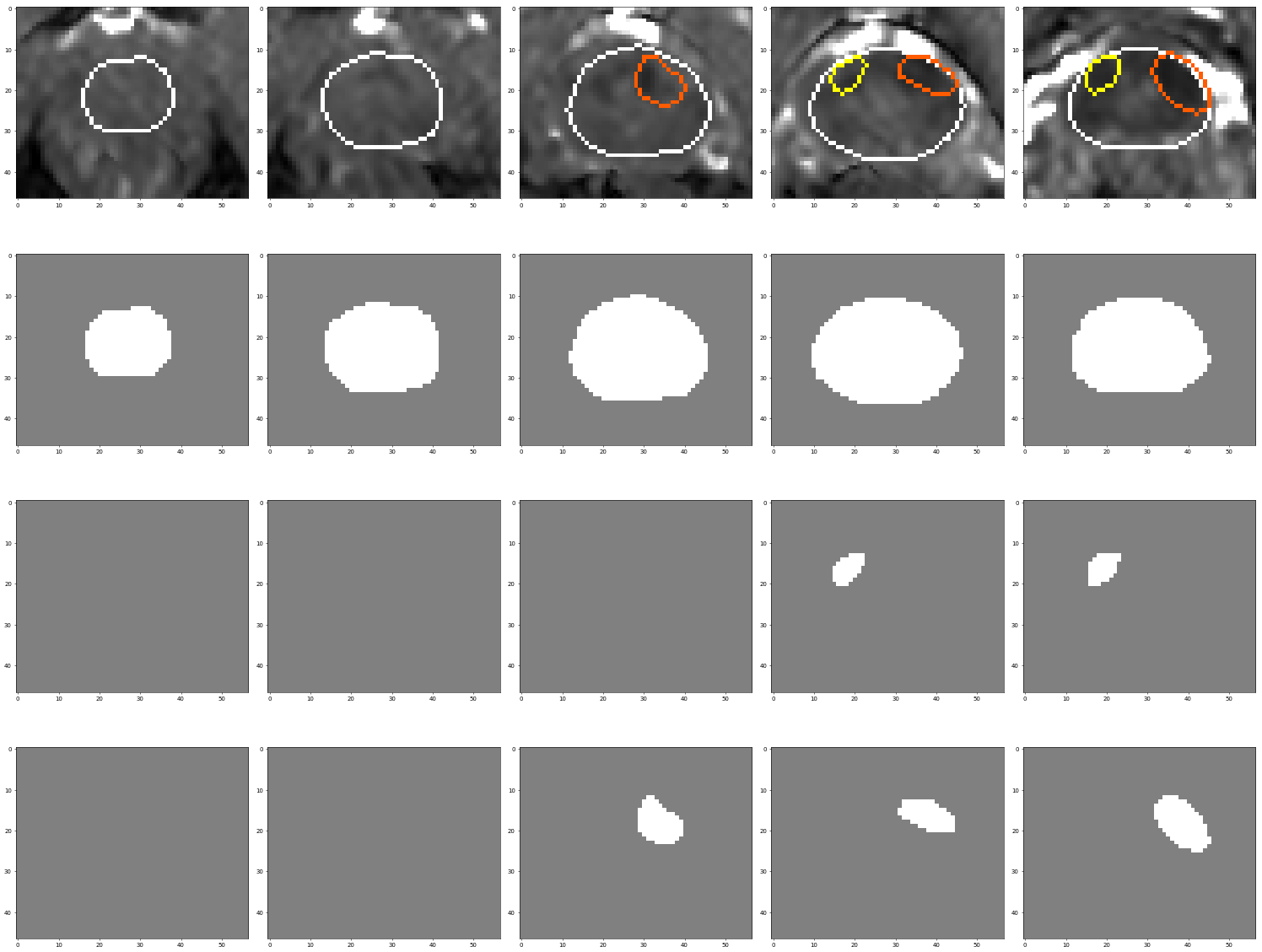

Supplement: S2 File — Files DWI-Mono-ADCm-xxx.png, T2-fitted-xxx.png, and T2w-std-xxx.png correspond to ADCm and T2 parametric maps, and T2-weighted images of each patient, respectively. On the first row of slices they show positions of regions of interest placed on the prostate cancer lesions (red, yellow) and around whole prostate (white). The prostate mask is on the second row, while the remaining rows are lesion masks. Files histology-xx.jpg contain the whole mount prostatectomy sections of each patient, with tumor outlines in green. Please note that identical MRI acquisition protocol has been used on all patients, including slice thickness. Here all prostate cancer masks are show with corresponding whole mount prostatectomy sections. (ZIP) [file pone.0217702.s002.zip › supporting_figures/DWI-Mono-ADCm-023.png]

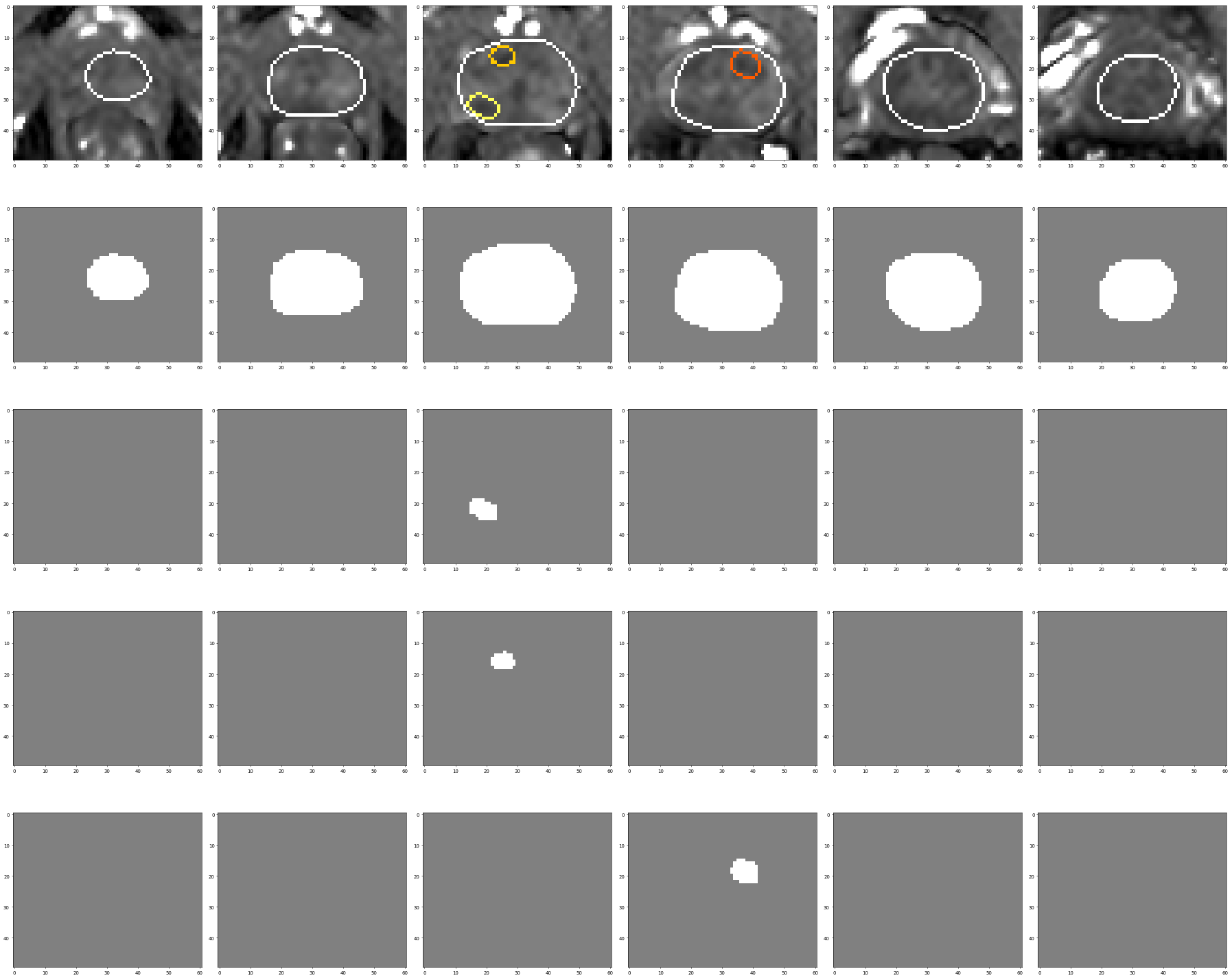

Supplement: S2 File — Files DWI-Mono-ADCm-xxx.png, T2-fitted-xxx.png, and T2w-std-xxx.png correspond to ADCm and T2 parametric maps, and T2-weighted images of each patient, respectively. On the first row of slices they show positions of regions of interest placed on the prostate cancer lesions (red, yellow) and around whole prostate (white). The prostate mask is on the second row, while the remaining rows are lesion masks. Files histology-xx.jpg contain the whole mount prostatectomy sections of each patient, with tumor outlines in green. Please note that identical MRI acquisition protocol has been used on all patients, including slice thickness. Here all prostate cancer masks are show with corresponding whole mount prostatectomy sections. (ZIP) [file pone.0217702.s002.zip › supporting_figures/DWI-Mono-ADCm-024.png]

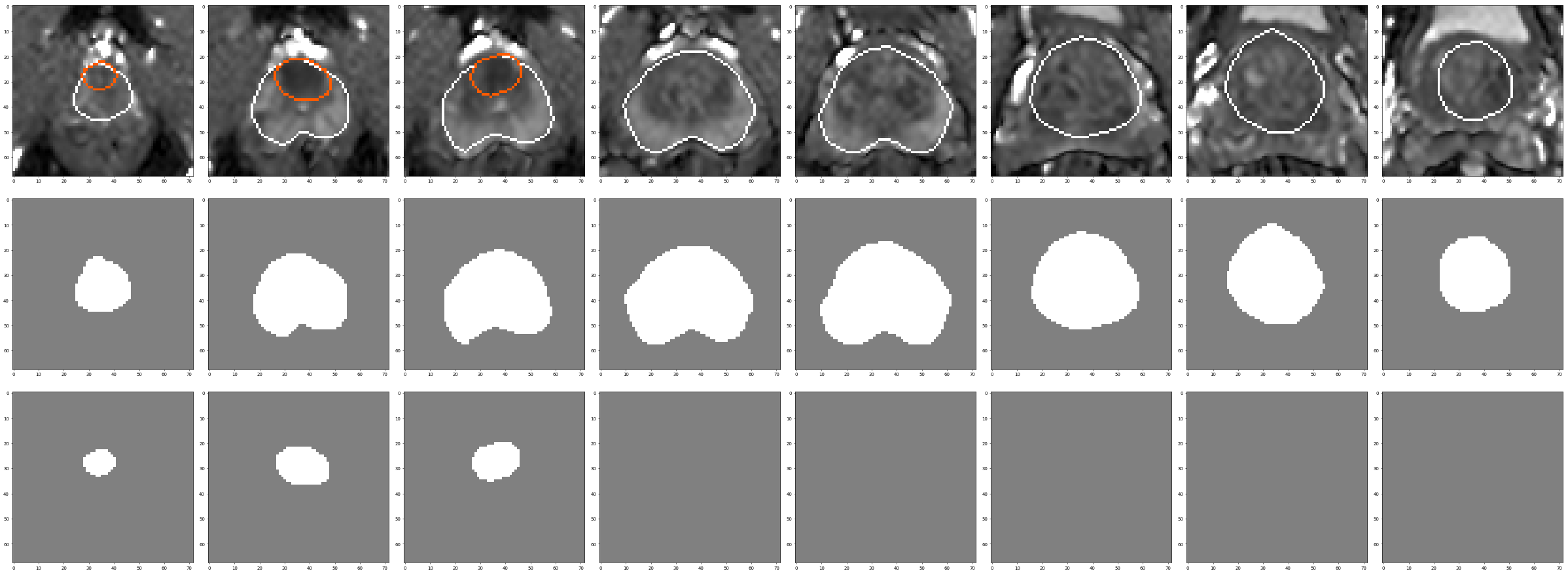

Supplement: S2 File — Files DWI-Mono-ADCm-xxx.png, T2-fitted-xxx.png, and T2w-std-xxx.png correspond to ADCm and T2 parametric maps, and T2-weighted images of each patient, respectively. On the first row of slices they show positions of regions of interest placed on the prostate cancer lesions (red, yellow) and around whole prostate (white). The prostate mask is on the second row, while the remaining rows are lesion masks. Files histology-xx.jpg contain the whole mount prostatectomy sections of each patient, with tumor outlines in green. Please note that identical MRI acquisition protocol has been used on all patients, including slice thickness. Here all prostate cancer masks are show with corresponding whole mount prostatectomy sections. (ZIP) [file pone.0217702.s002.zip › supporting_figures/DWI-Mono-ADCm-025.png]

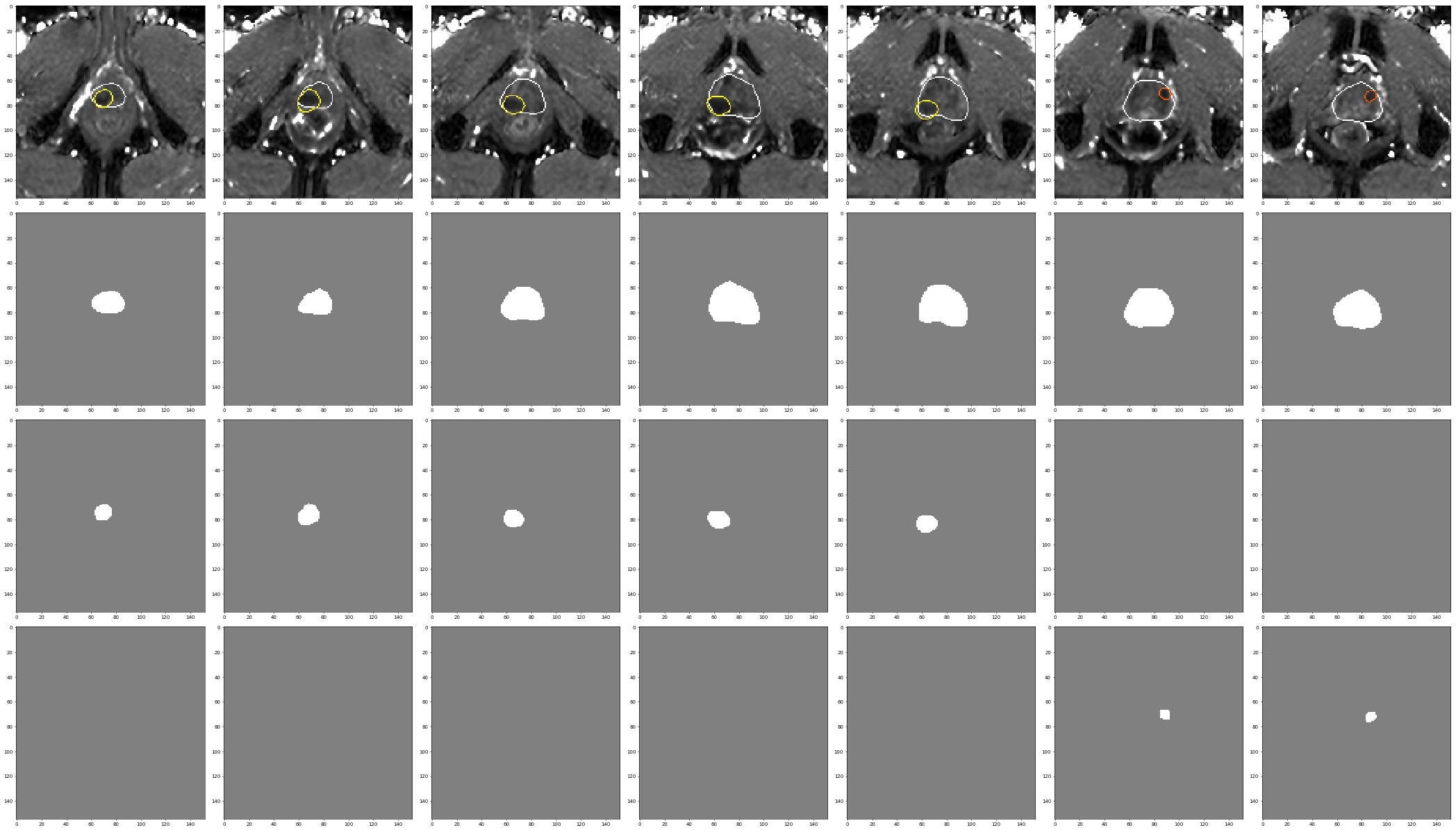

Supplement: S2 File — Files DWI-Mono-ADCm-xxx.png, T2-fitted-xxx.png, and T2w-std-xxx.png correspond to ADCm and T2 parametric maps, and T2-weighted images of each patient, respectively. On the first row of slices they show positions of regions of interest placed on the prostate cancer lesions (red, yellow) and around whole prostate (white). The prostate mask is on the second row, while the remaining rows are lesion masks. Files histology-xx.jpg contain the whole mount prostatectomy sections of each patient, with tumor outlines in green. Please note that identical MRI acquisition protocol has been used on all patients, including slice thickness. Here all prostate cancer masks are show with corresponding whole mount prostatectomy sections. (ZIP) [file pone.0217702.s002.zip › supporting_figures/DWI-Mono-ADCm-026.png]

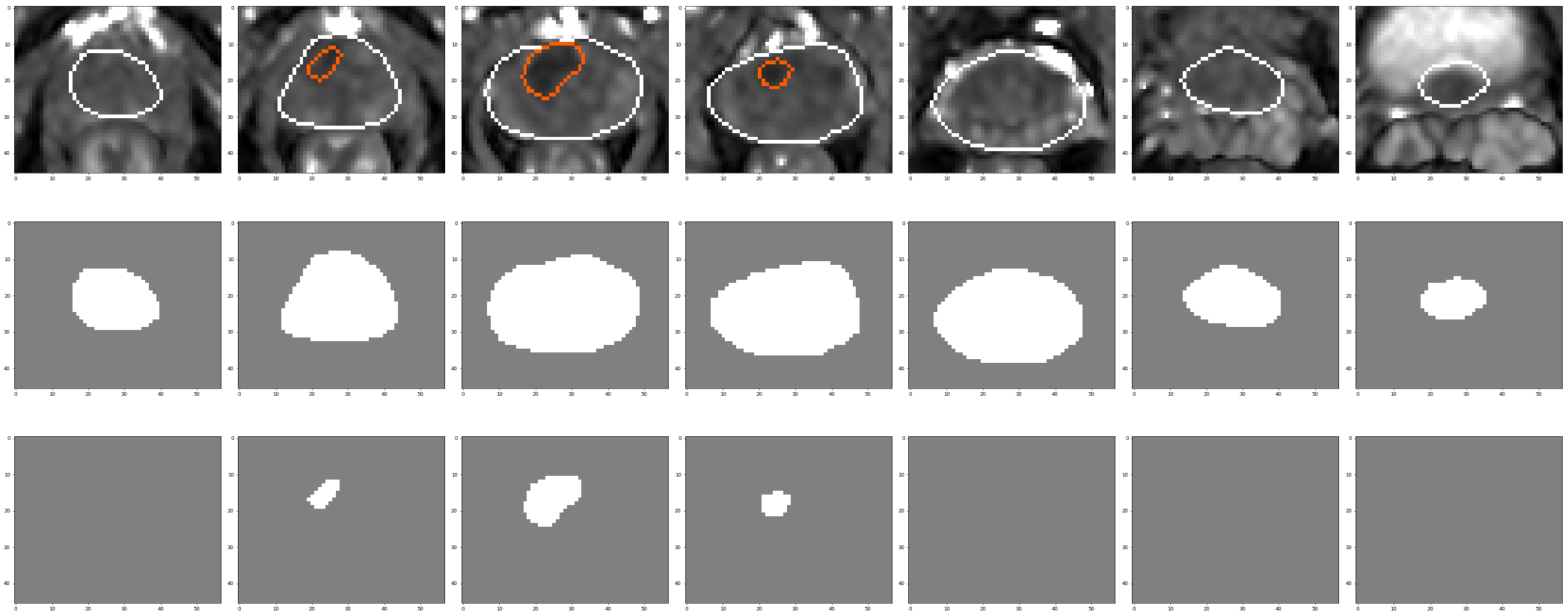

Supplement: S2 File — Files DWI-Mono-ADCm-xxx.png, T2-fitted-xxx.png, and T2w-std-xxx.png correspond to ADCm and T2 parametric maps, and T2-weighted images of each patient, respectively. On the first row of slices they show positions of regions of interest placed on the prostate cancer lesions (red, yellow) and around whole prostate (white). The prostate mask is on the second row, while the remaining rows are lesion masks. Files histology-xx.jpg contain the whole mount prostatectomy sections of each patient, with tumor outlines in green. Please note that identical MRI acquisition protocol has been used on all patients, including slice thickness. Here all prostate cancer masks are show with corresponding whole mount prostatectomy sections. (ZIP) [file pone.0217702.s002.zip › supporting_figures/DWI-Mono-ADCm-027.png]

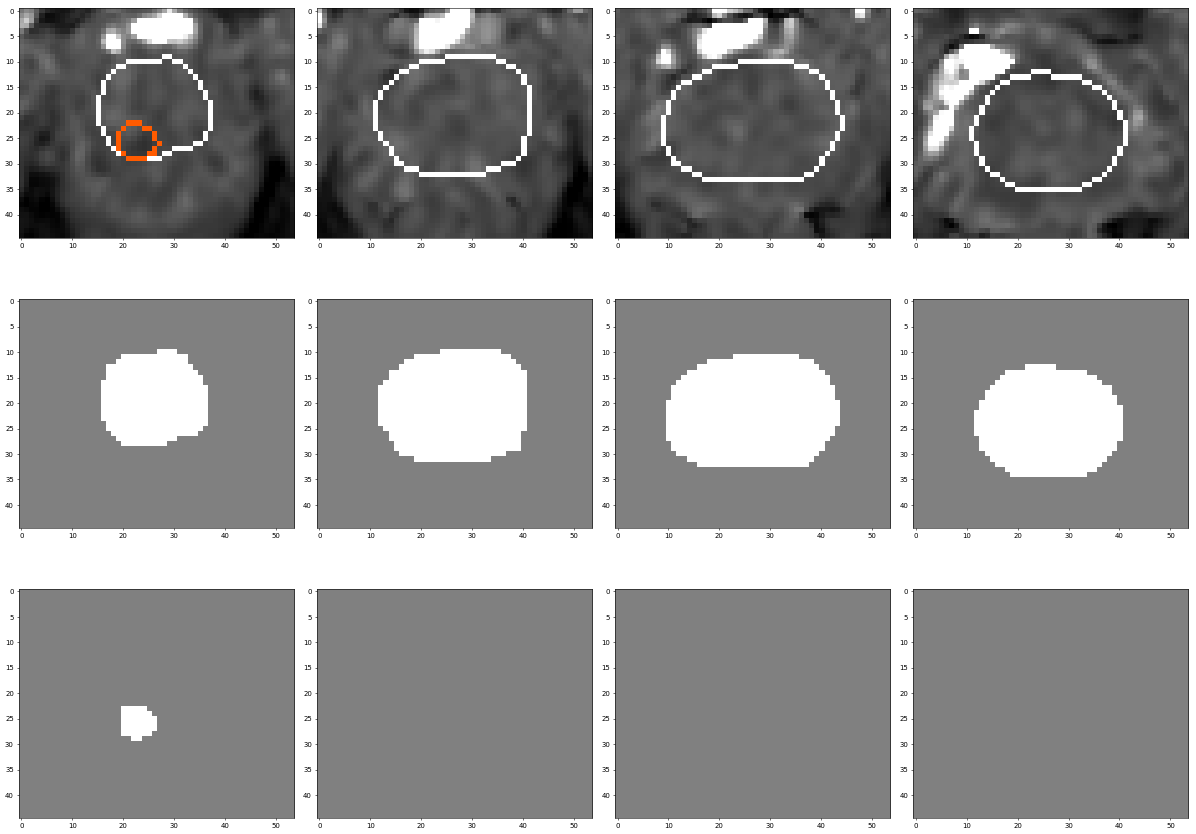

Supplement: S2 File — Files DWI-Mono-ADCm-xxx.png, T2-fitted-xxx.png, and T2w-std-xxx.png correspond to ADCm and T2 parametric maps, and T2-weighted images of each patient, respectively. On the first row of slices they show positions of regions of interest placed on the prostate cancer lesions (red, yellow) and around whole prostate (white). The prostate mask is on the second row, while the remaining rows are lesion masks. Files histology-xx.jpg contain the whole mount prostatectomy sections of each patient, with tumor outlines in green. Please note that identical MRI acquisition protocol has been used on all patients, including slice thickness. Here all prostate cancer masks are show with corresponding whole mount prostatectomy sections. (ZIP) [file pone.0217702.s002.zip › supporting_figures/DWI-Mono-ADCm-028.png]

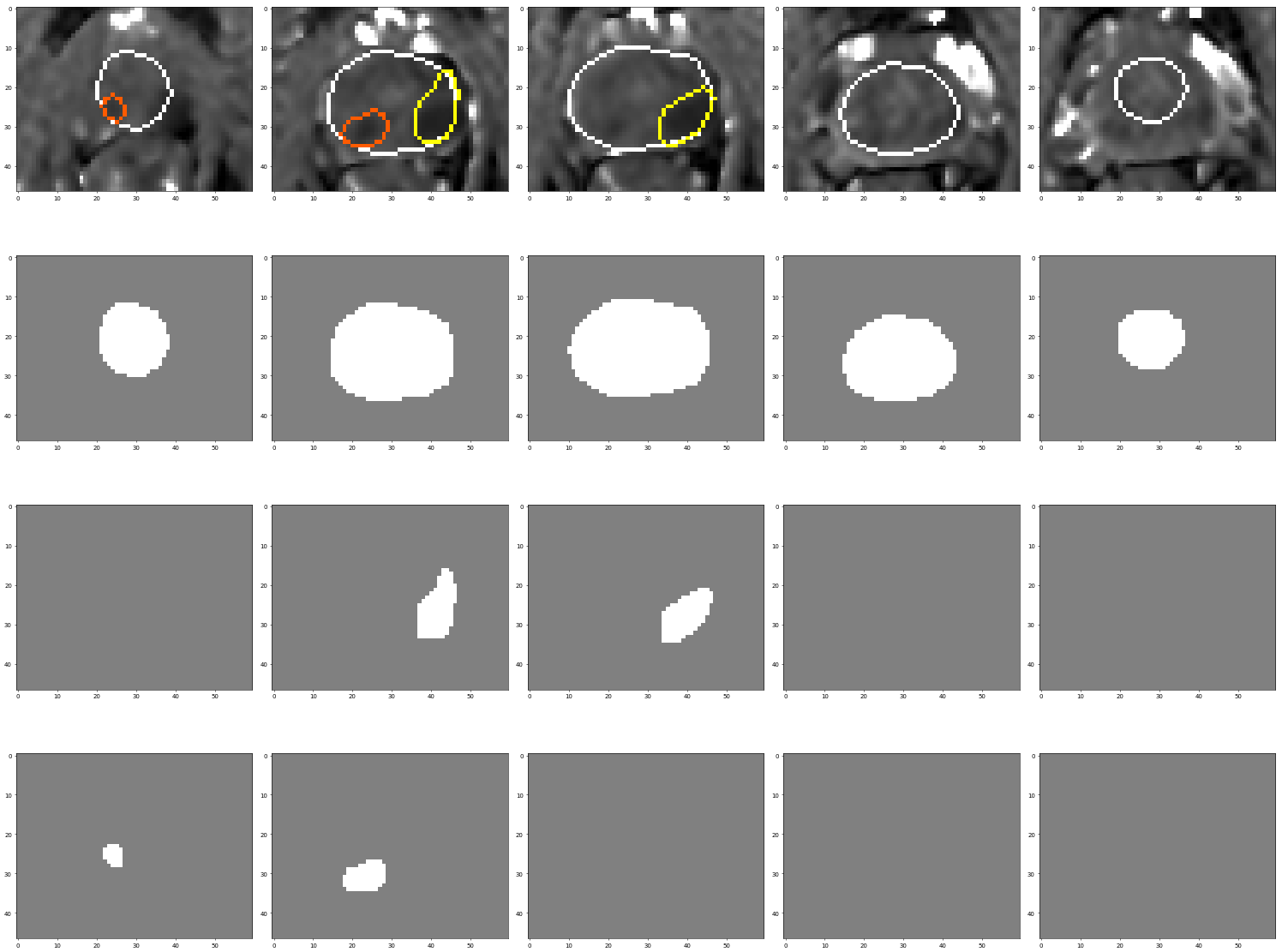

Supplement: S2 File — Files DWI-Mono-ADCm-xxx.png, T2-fitted-xxx.png, and T2w-std-xxx.png correspond to ADCm and T2 parametric maps, and T2-weighted images of each patient, respectively. On the first row of slices they show positions of regions of interest placed on the prostate cancer lesions (red, yellow) and around whole prostate (white). The prostate mask is on the second row, while the remaining rows are lesion masks. Files histology-xx.jpg contain the whole mount prostatectomy sections of each patient, with tumor outlines in green. Please note that identical MRI acquisition protocol has been used on all patients, including slice thickness. Here all prostate cancer masks are show with corresponding whole mount prostatectomy sections. (ZIP) [file pone.0217702.s002.zip › supporting_figures/DWI-Mono-ADCm-029.png]

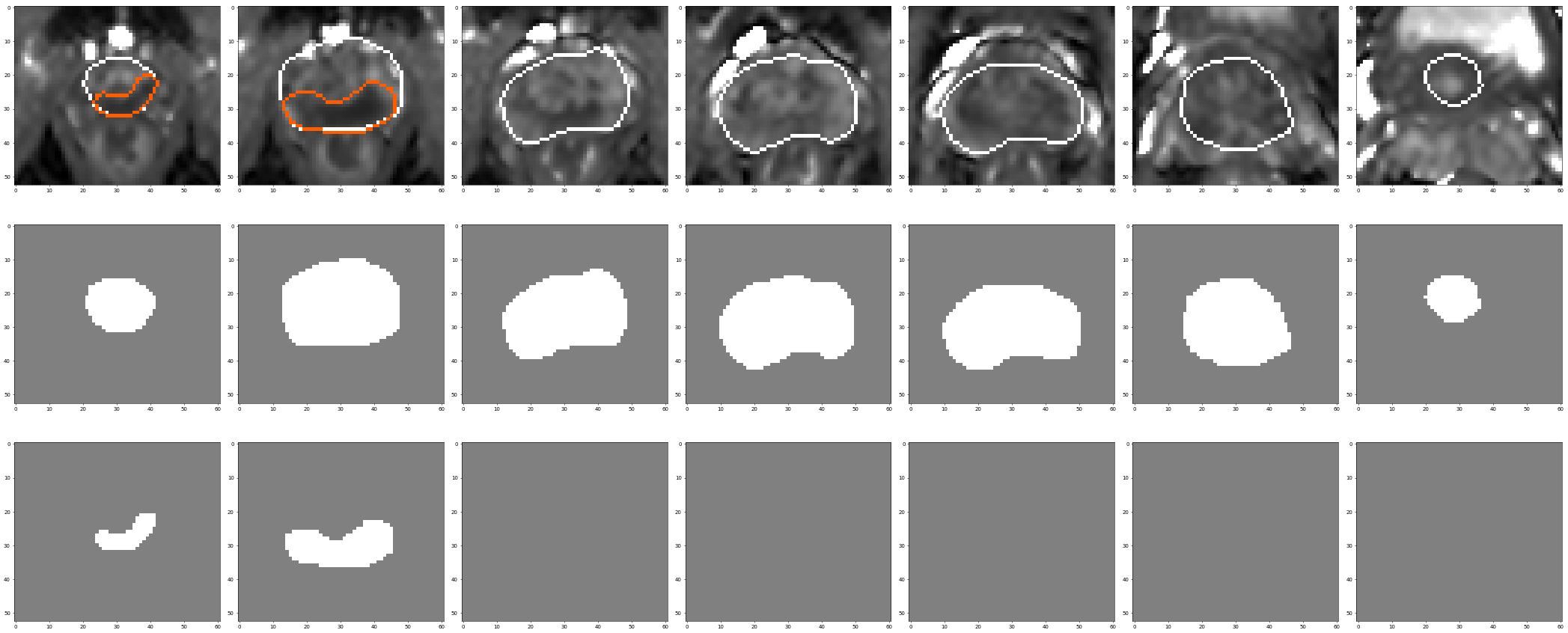

Supplement: S2 File — Files DWI-Mono-ADCm-xxx.png, T2-fitted-xxx.png, and T2w-std-xxx.png correspond to ADCm and T2 parametric maps, and T2-weighted images of each patient, respectively. On the first row of slices they show positions of regions of interest placed on the prostate cancer lesions (red, yellow) and around whole prostate (white). The prostate mask is on the second row, while the remaining rows are lesion masks. Files histology-xx.jpg contain the whole mount prostatectomy sections of each patient, with tumor outlines in green. Please note that identical MRI acquisition protocol has been used on all patients, including slice thickness. Here all prostate cancer masks are show with corresponding whole mount prostatectomy sections. (ZIP) [file pone.0217702.s002.zip › supporting_figures/DWI-Mono-ADCm-030.png]

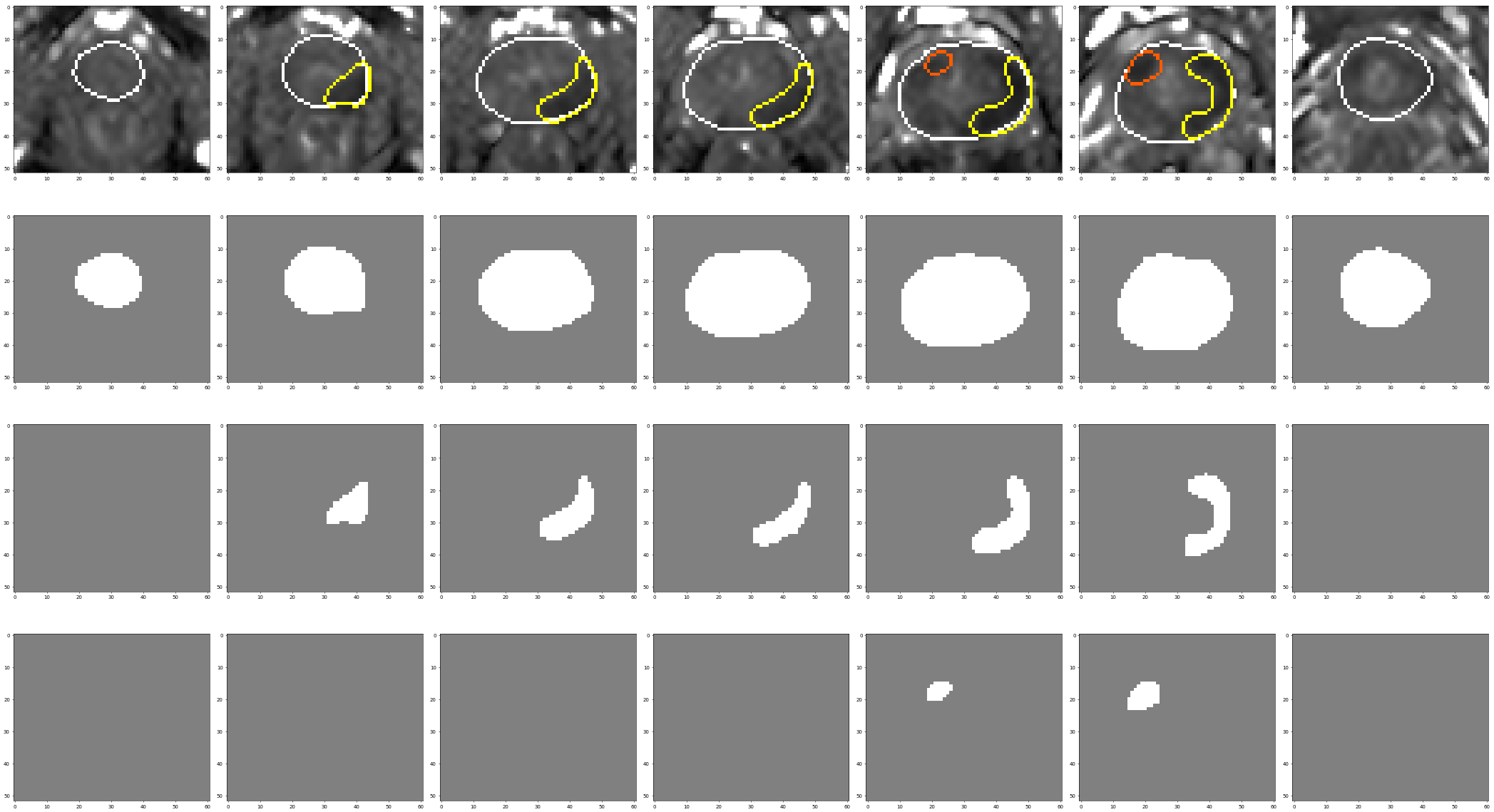

Supplement: S2 File — Files DWI-Mono-ADCm-xxx.png, T2-fitted-xxx.png, and T2w-std-xxx.png correspond to ADCm and T2 parametric maps, and T2-weighted images of each patient, respectively. On the first row of slices they show positions of regions of interest placed on the prostate cancer lesions (red, yellow) and around whole prostate (white). The prostate mask is on the second row, while the remaining rows are lesion masks. Files histology-xx.jpg contain the whole mount prostatectomy sections of each patient, with tumor outlines in green. Please note that identical MRI acquisition protocol has been used on all patients, including slice thickness. Here all prostate cancer masks are show with corresponding whole mount prostatectomy sections. (ZIP) [file pone.0217702.s002.zip › supporting_figures/DWI-Mono-ADCm-031.png]

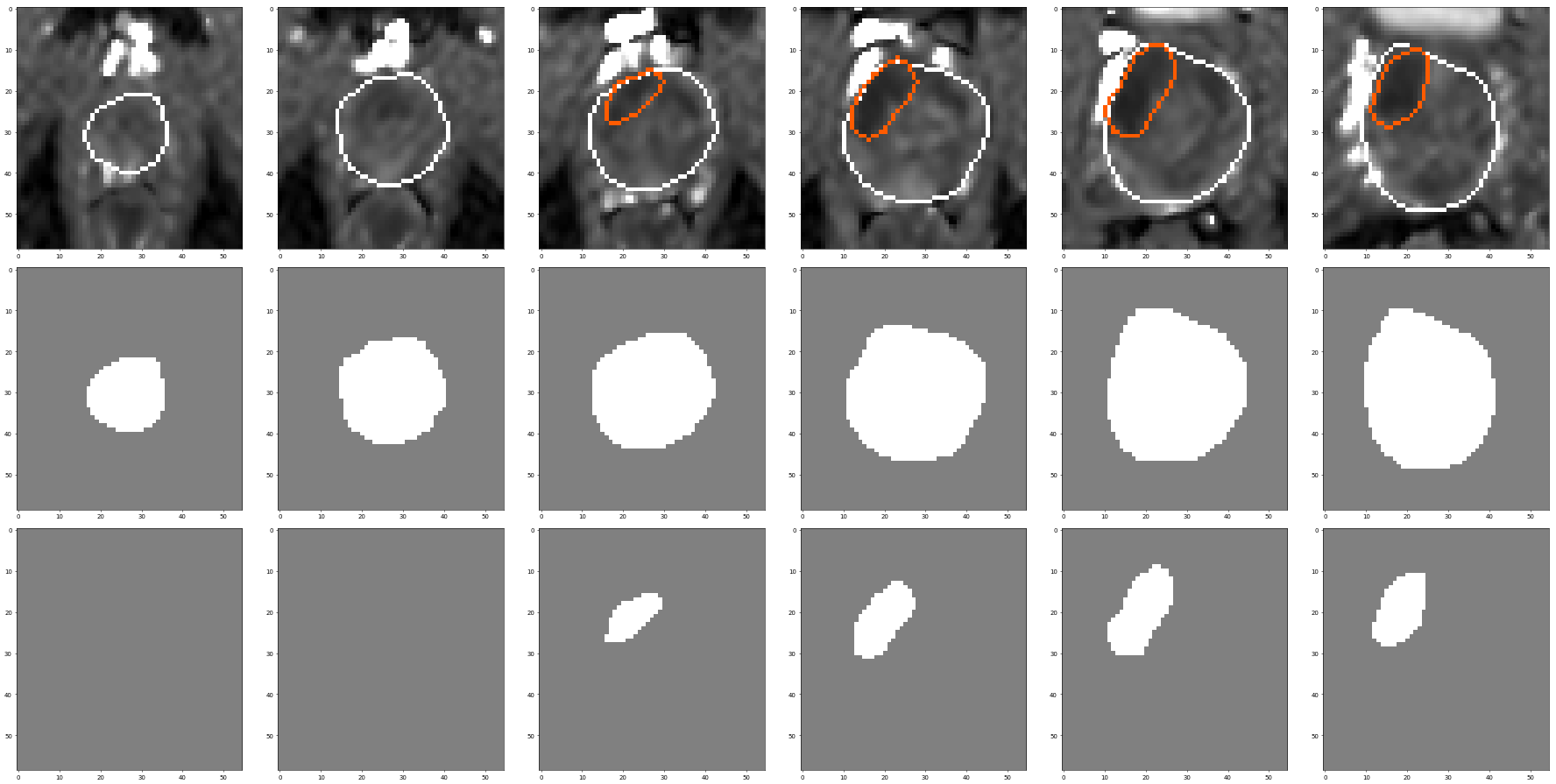

Supplement: S2 File — Files DWI-Mono-ADCm-xxx.png, T2-fitted-xxx.png, and T2w-std-xxx.png correspond to ADCm and T2 parametric maps, and T2-weighted images of each patient, respectively. On the first row of slices they show positions of regions of interest placed on the prostate cancer lesions (red, yellow) and around whole prostate (white). The prostate mask is on the second row, while the remaining rows are lesion masks. Files histology-xx.jpg contain the whole mount prostatectomy sections of each patient, with tumor outlines in green. Please note that identical MRI acquisition protocol has been used on all patients, including slice thickness. Here all prostate cancer masks are show with corresponding whole mount prostatectomy sections. (ZIP) [file pone.0217702.s002.zip › supporting_figures/DWI-Mono-ADCm-032.png]

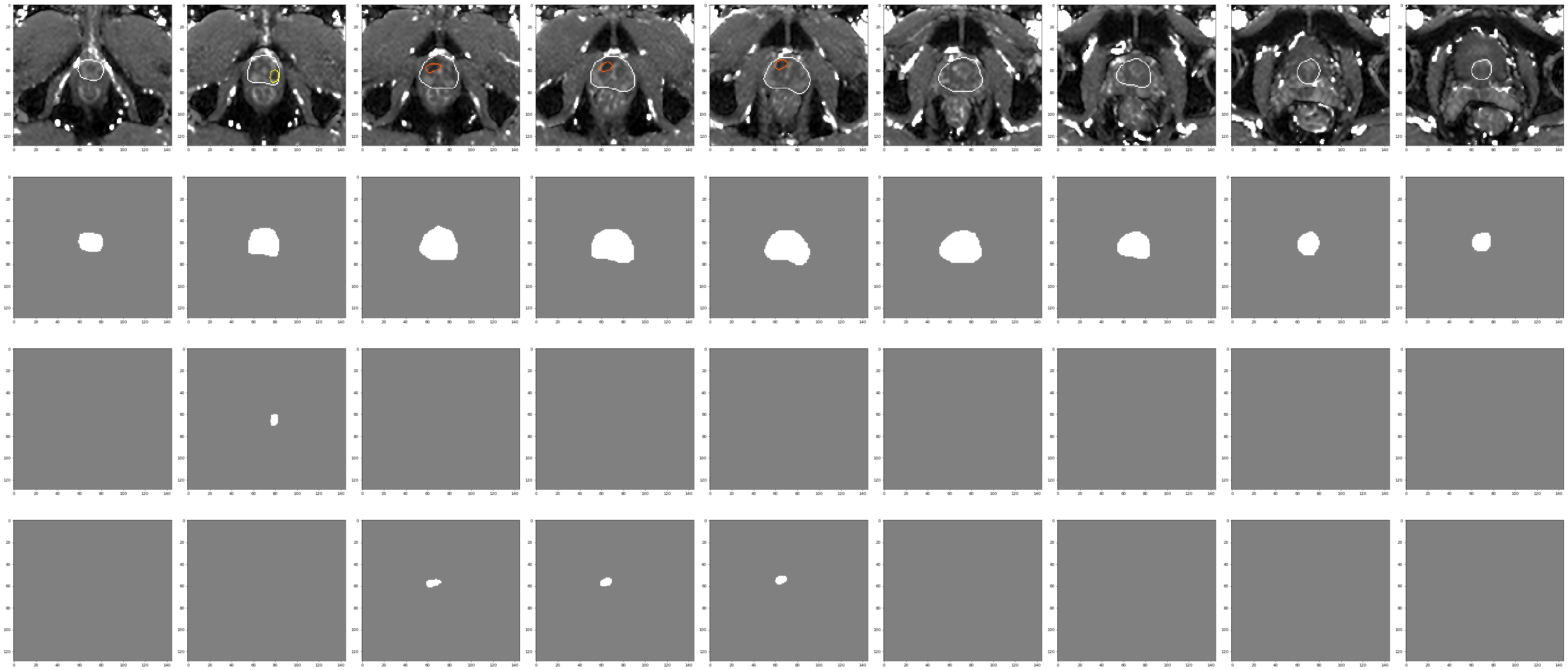

Supplement: S2 File — Files DWI-Mono-ADCm-xxx.png, T2-fitted-xxx.png, and T2w-std-xxx.png correspond to ADCm and T2 parametric maps, and T2-weighted images of each patient, respectively. On the first row of slices they show positions of regions of interest placed on the prostate cancer lesions (red, yellow) and around whole prostate (white). The prostate mask is on the second row, while the remaining rows are lesion masks. Files histology-xx.jpg contain the whole mount prostatectomy sections of each patient, with tumor outlines in green. Please note that identical MRI acquisition protocol has been used on all patients, including slice thickness. Here all prostate cancer masks are show with corresponding whole mount prostatectomy sections. (ZIP) [file pone.0217702.s002.zip › supporting_figures/DWI-Mono-ADCm-033.png]

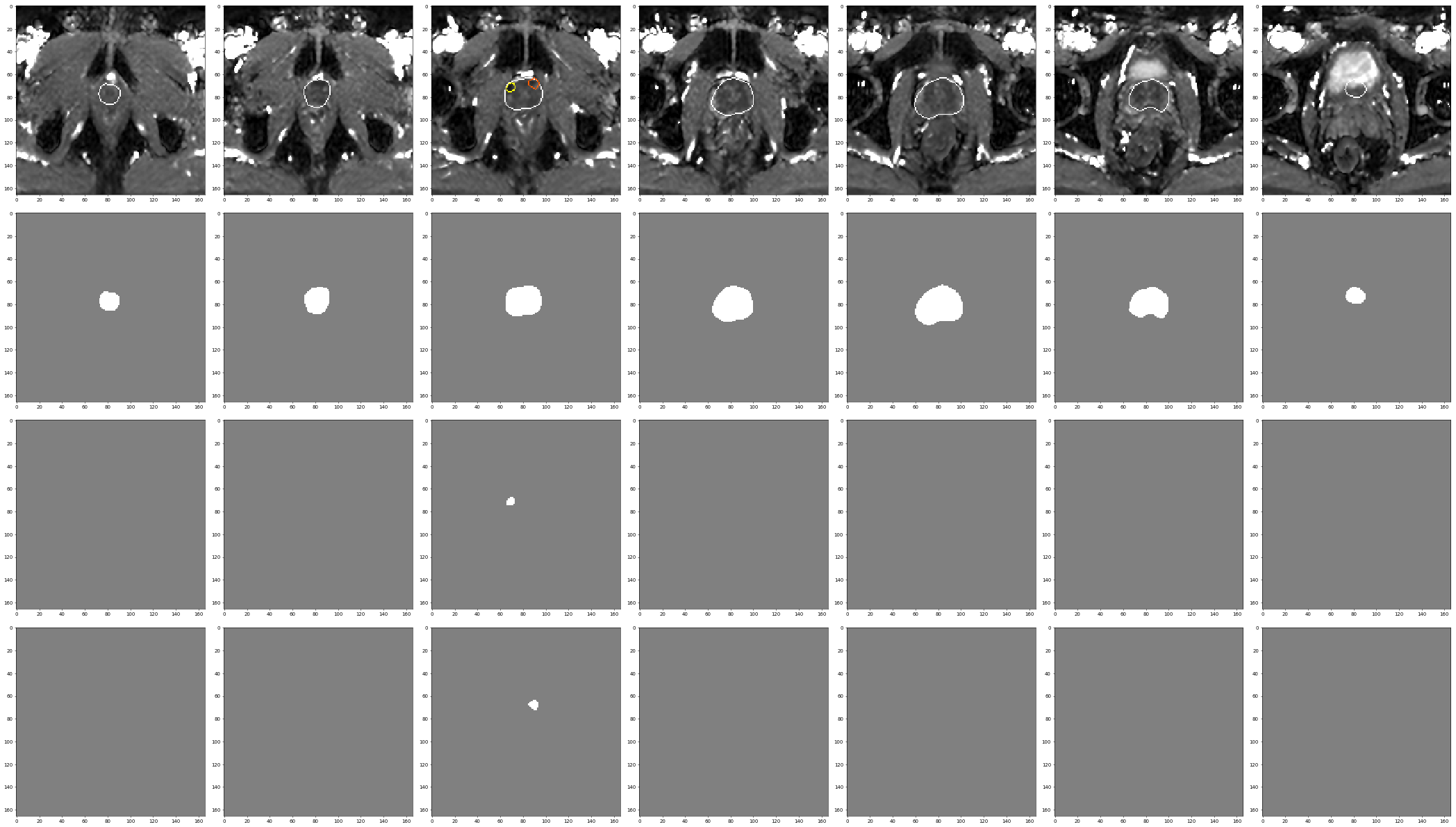

Supplement: S2 File — Files DWI-Mono-ADCm-xxx.png, T2-fitted-xxx.png, and T2w-std-xxx.png correspond to ADCm and T2 parametric maps, and T2-weighted images of each patient, respectively. On the first row of slices they show positions of regions of interest placed on the prostate cancer lesions (red, yellow) and around whole prostate (white). The prostate mask is on the second row, while the remaining rows are lesion masks. Files histology-xx.jpg contain the whole mount prostatectomy sections of each patient, with tumor outlines in green. Please note that identical MRI acquisition protocol has been used on all patients, including slice thickness. Here all prostate cancer masks are show with corresponding whole mount prostatectomy sections. (ZIP) [file pone.0217702.s002.zip › supporting_figures/DWI-Mono-ADCm-034.png]

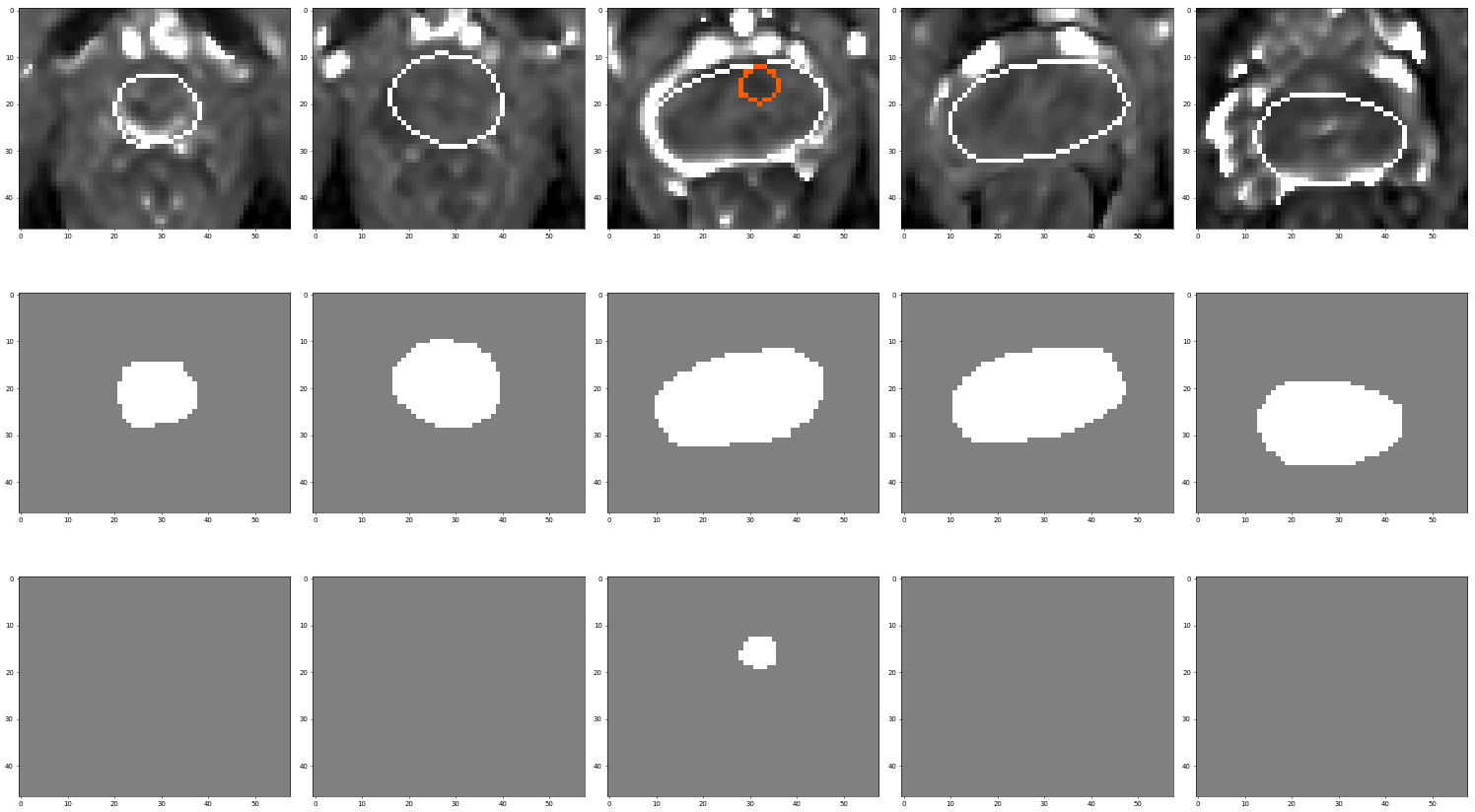

Supplement: S2 File — Files DWI-Mono-ADCm-xxx.png, T2-fitted-xxx.png, and T2w-std-xxx.png correspond to ADCm and T2 parametric maps, and T2-weighted images of each patient, respectively. On the first row of slices they show positions of regions of interest placed on the prostate cancer lesions (red, yellow) and around whole prostate (white). The prostate mask is on the second row, while the remaining rows are lesion masks. Files histology-xx.jpg contain the whole mount prostatectomy sections of each patient, with tumor outlines in green. Please note that identical MRI acquisition protocol has been used on all patients, including slice thickness. Here all prostate cancer masks are show with corresponding whole mount prostatectomy sections. (ZIP) [file pone.0217702.s002.zip › supporting_figures/DWI-Mono-ADCm-035.png]

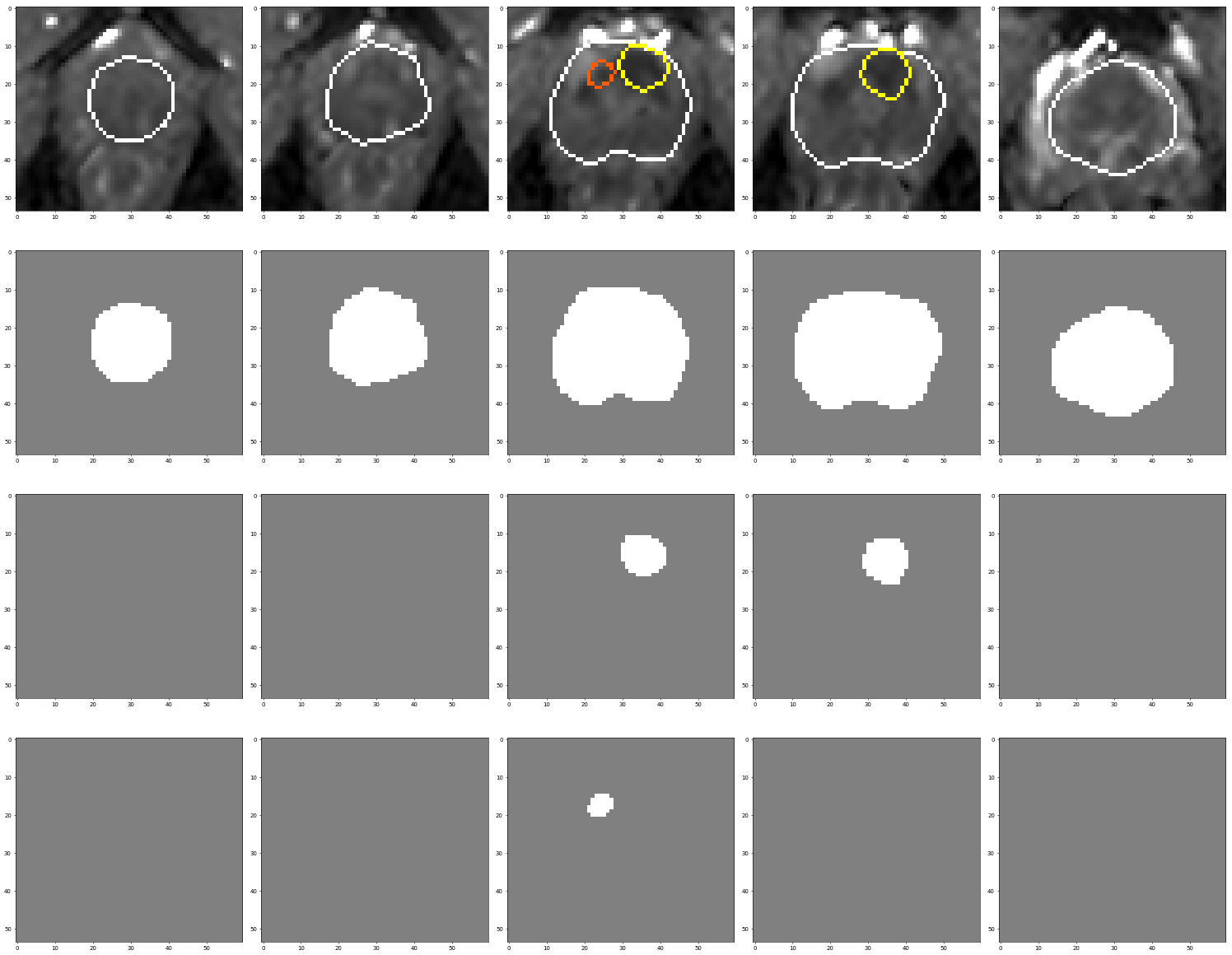

Supplement: S2 File — Files DWI-Mono-ADCm-xxx.png, T2-fitted-xxx.png, and T2w-std-xxx.png correspond to ADCm and T2 parametric maps, and T2-weighted images of each patient, respectively. On the first row of slices they show positions of regions of interest placed on the prostate cancer lesions (red, yellow) and around whole prostate (white). The prostate mask is on the second row, while the remaining rows are lesion masks. Files histology-xx.jpg contain the whole mount prostatectomy sections of each patient, with tumor outlines in green. Please note that identical MRI acquisition protocol has been used on all patients, including slice thickness. Here all prostate cancer masks are show with corresponding whole mount prostatectomy sections. (ZIP) [file pone.0217702.s002.zip › supporting_figures/DWI-Mono-ADCm-036.png]

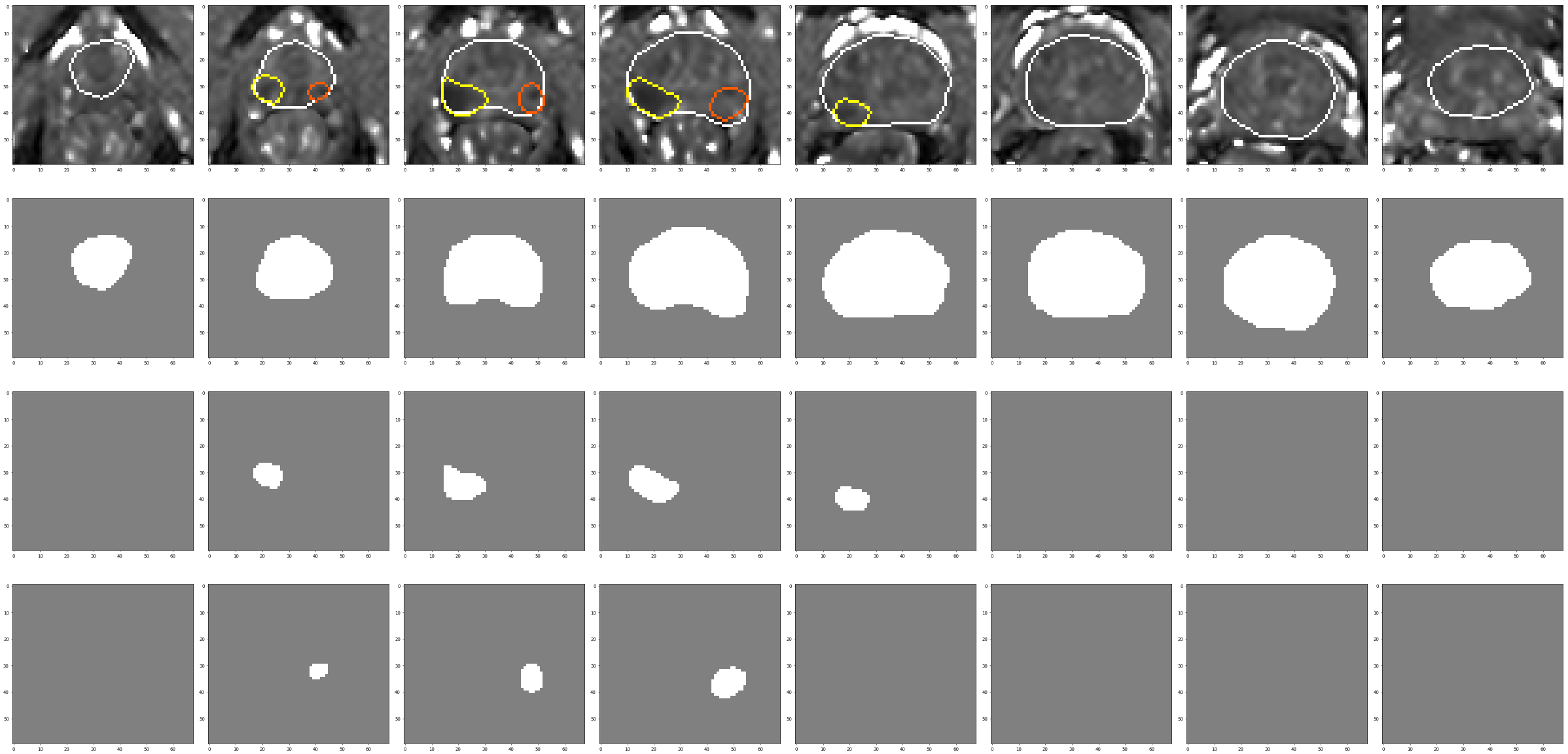

Supplement: S2 File — Files DWI-Mono-ADCm-xxx.png, T2-fitted-xxx.png, and T2w-std-xxx.png correspond to ADCm and T2 parametric maps, and T2-weighted images of each patient, respectively. On the first row of slices they show positions of regions of interest placed on the prostate cancer lesions (red, yellow) and around whole prostate (white). The prostate mask is on the second row, while the remaining rows are lesion masks. Files histology-xx.jpg contain the whole mount prostatectomy sections of each patient, with tumor outlines in green. Please note that identical MRI acquisition protocol has been used on all patients, including slice thickness. Here all prostate cancer masks are show with corresponding whole mount prostatectomy sections. (ZIP) [file pone.0217702.s002.zip › supporting_figures/DWI-Mono-ADCm-037.png]

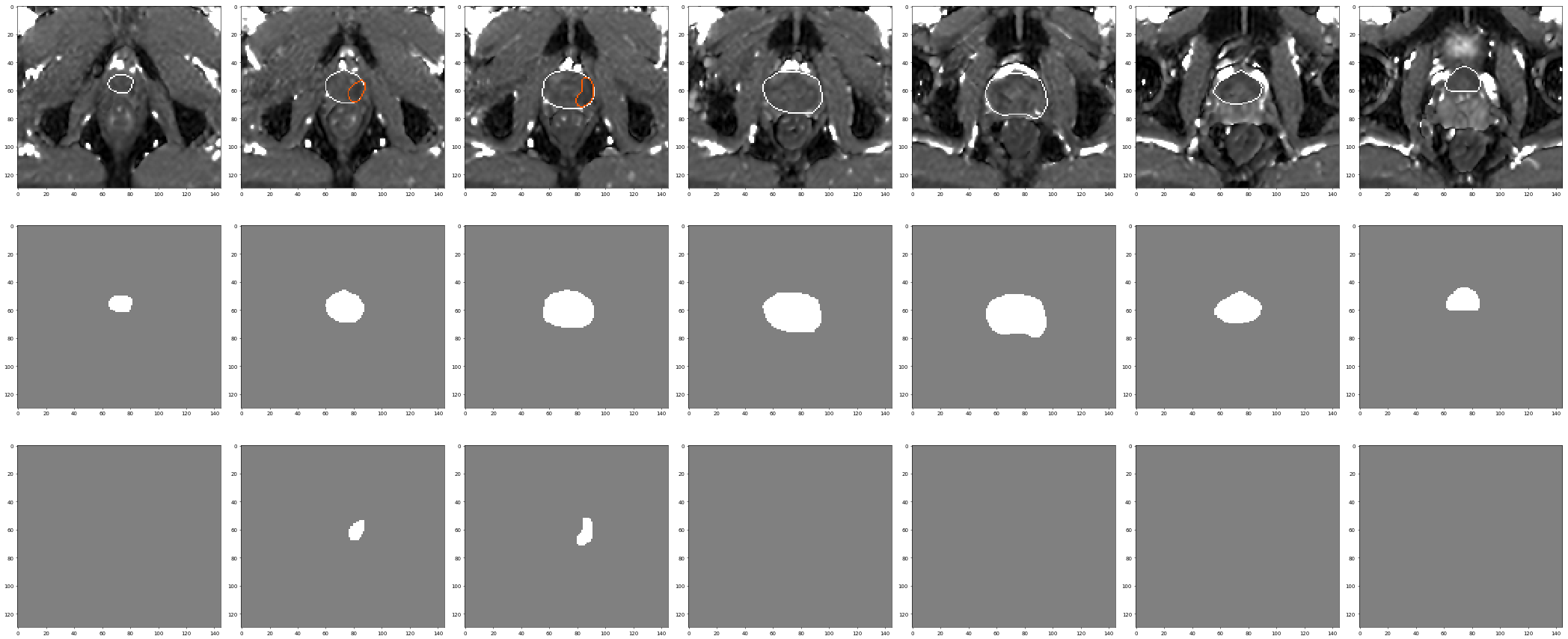

Supplement: S2 File — Files DWI-Mono-ADCm-xxx.png, T2-fitted-xxx.png, and T2w-std-xxx.png correspond to ADCm and T2 parametric maps, and T2-weighted images of each patient, respectively. On the first row of slices they show positions of regions of interest placed on the prostate cancer lesions (red, yellow) and around whole prostate (white). The prostate mask is on the second row, while the remaining rows are lesion masks. Files histology-xx.jpg contain the whole mount prostatectomy sections of each patient, with tumor outlines in green. Please note that identical MRI acquisition protocol has been used on all patients, including slice thickness. Here all prostate cancer masks are show with corresponding whole mount prostatectomy sections. (ZIP) [file pone.0217702.s002.zip › supporting_figures/DWI-Mono-ADCm-038.png]

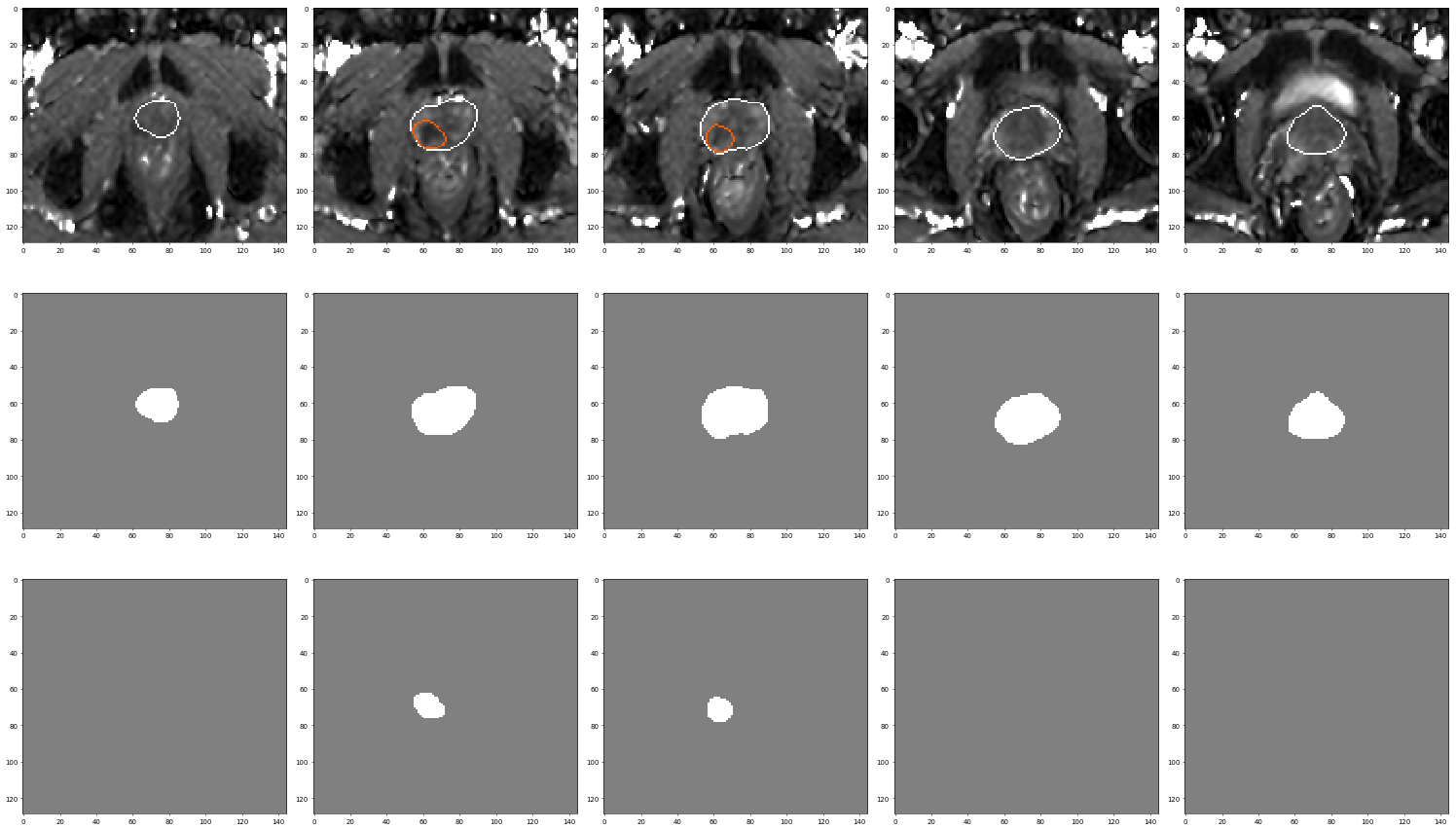

Supplement: S2 File — Files DWI-Mono-ADCm-xxx.png, T2-fitted-xxx.png, and T2w-std-xxx.png correspond to ADCm and T2 parametric maps, and T2-weighted images of each patient, respectively. On the first row of slices they show positions of regions of interest placed on the prostate cancer lesions (red, yellow) and around whole prostate (white). The prostate mask is on the second row, while the remaining rows are lesion masks. Files histology-xx.jpg contain the whole mount prostatectomy sections of each patient, with tumor outlines in green. Please note that identical MRI acquisition protocol has been used on all patients, including slice thickness. Here all prostate cancer masks are show with corresponding whole mount prostatectomy sections. (ZIP) [file pone.0217702.s002.zip › supporting_figures/DWI-Mono-ADCm-039.png]

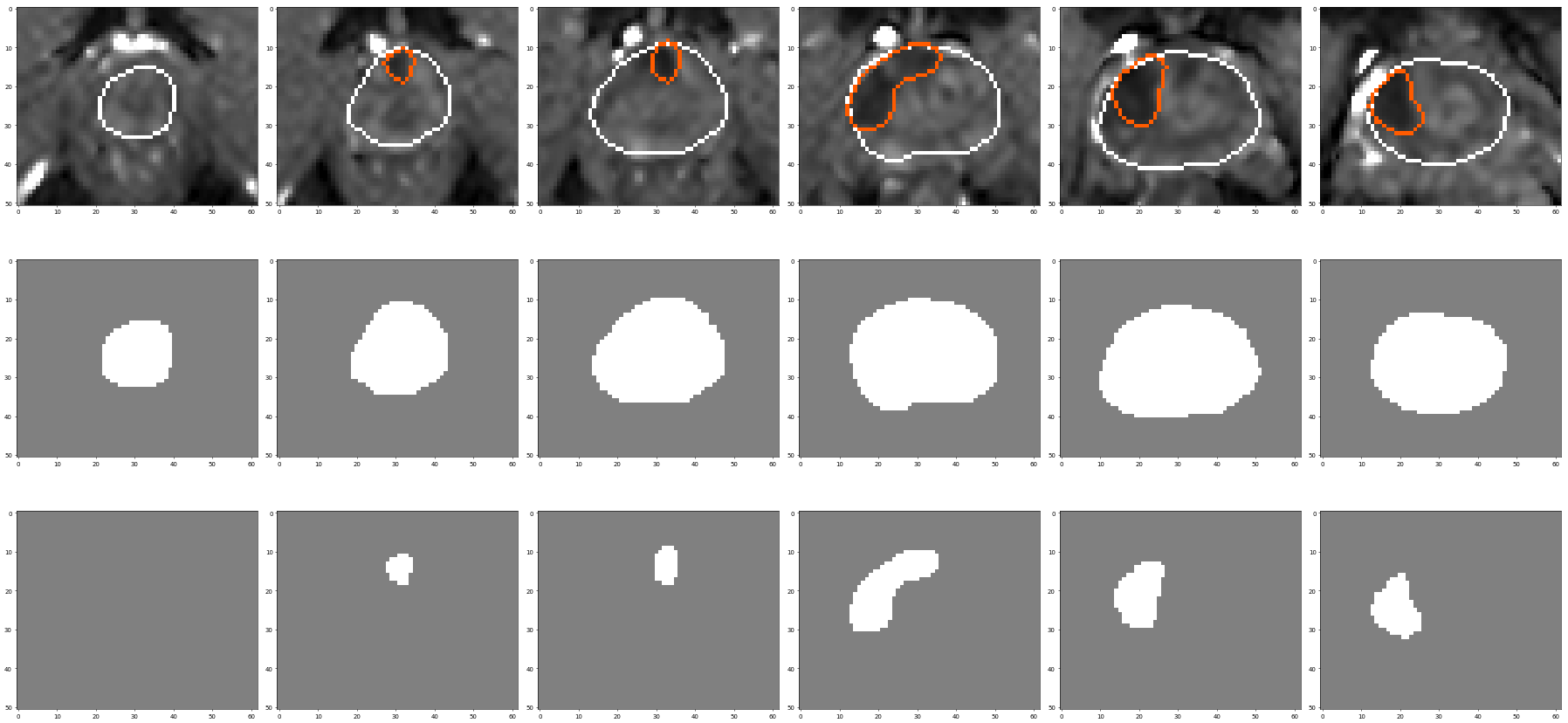

Supplement: S2 File — Files DWI-Mono-ADCm-xxx.png, T2-fitted-xxx.png, and T2w-std-xxx.png correspond to ADCm and T2 parametric maps, and T2-weighted images of each patient, respectively. On the first row of slices they show positions of regions of interest placed on the prostate cancer lesions (red, yellow) and around whole prostate (white). The prostate mask is on the second row, while the remaining rows are lesion masks. Files histology-xx.jpg contain the whole mount prostatectomy sections of each patient, with tumor outlines in green. Please note that identical MRI acquisition protocol has been used on all patients, including slice thickness. Here all prostate cancer masks are show with corresponding whole mount prostatectomy sections. (ZIP) [file pone.0217702.s002.zip › supporting_figures/DWI-Mono-ADCm-040.png]

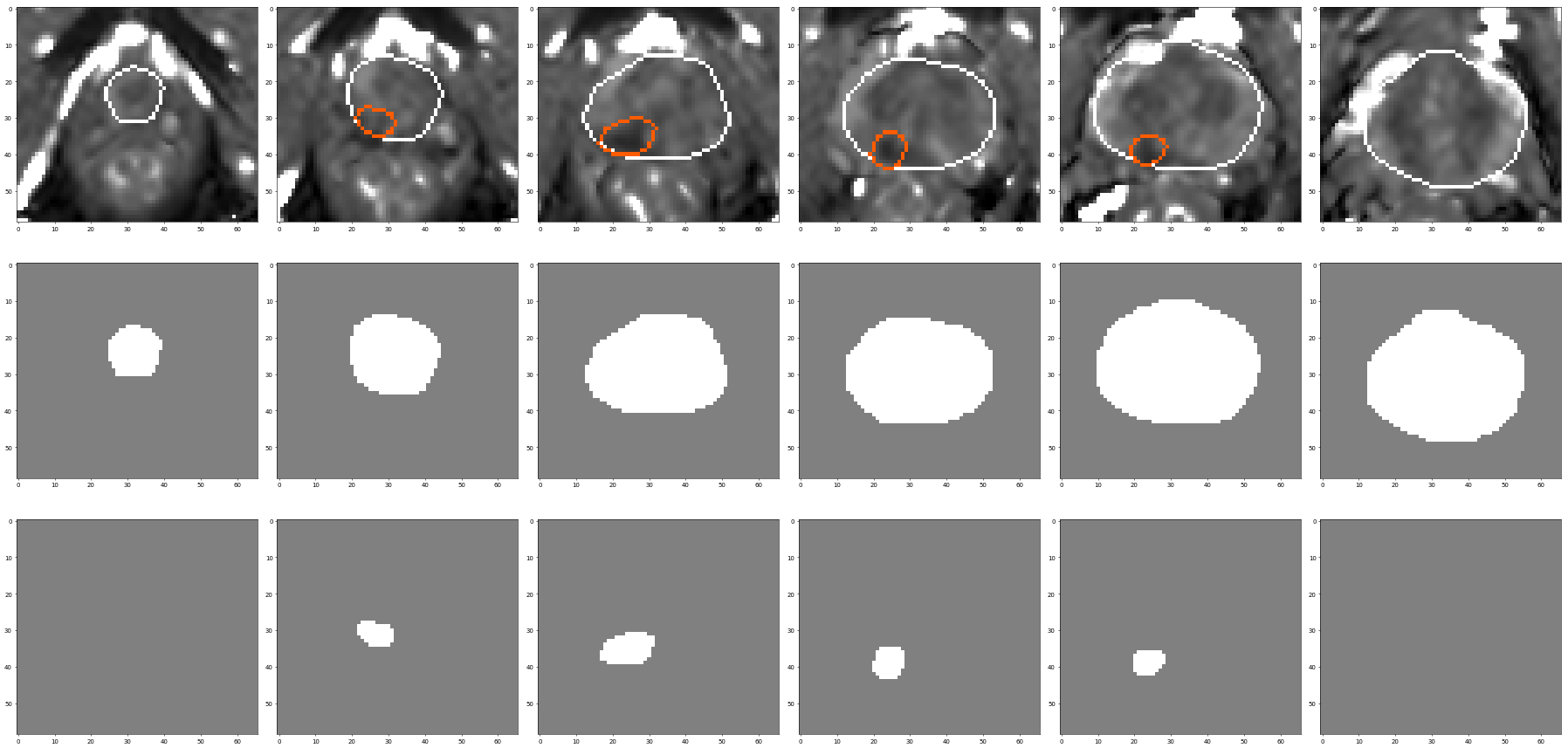

Supplement: S2 File — Files DWI-Mono-ADCm-xxx.png, T2-fitted-xxx.png, and T2w-std-xxx.png correspond to ADCm and T2 parametric maps, and T2-weighted images of each patient, respectively. On the first row of slices they show positions of regions of interest placed on the prostate cancer lesions (red, yellow) and around whole prostate (white). The prostate mask is on the second row, while the remaining rows are lesion masks. Files histology-xx.jpg contain the whole mount prostatectomy sections of each patient, with tumor outlines in green. Please note that identical MRI acquisition protocol has been used on all patients, including slice thickness. Here all prostate cancer masks are show with corresponding whole mount prostatectomy sections. (ZIP) [file pone.0217702.s002.zip › supporting_figures/DWI-Mono-ADCm-041.png]

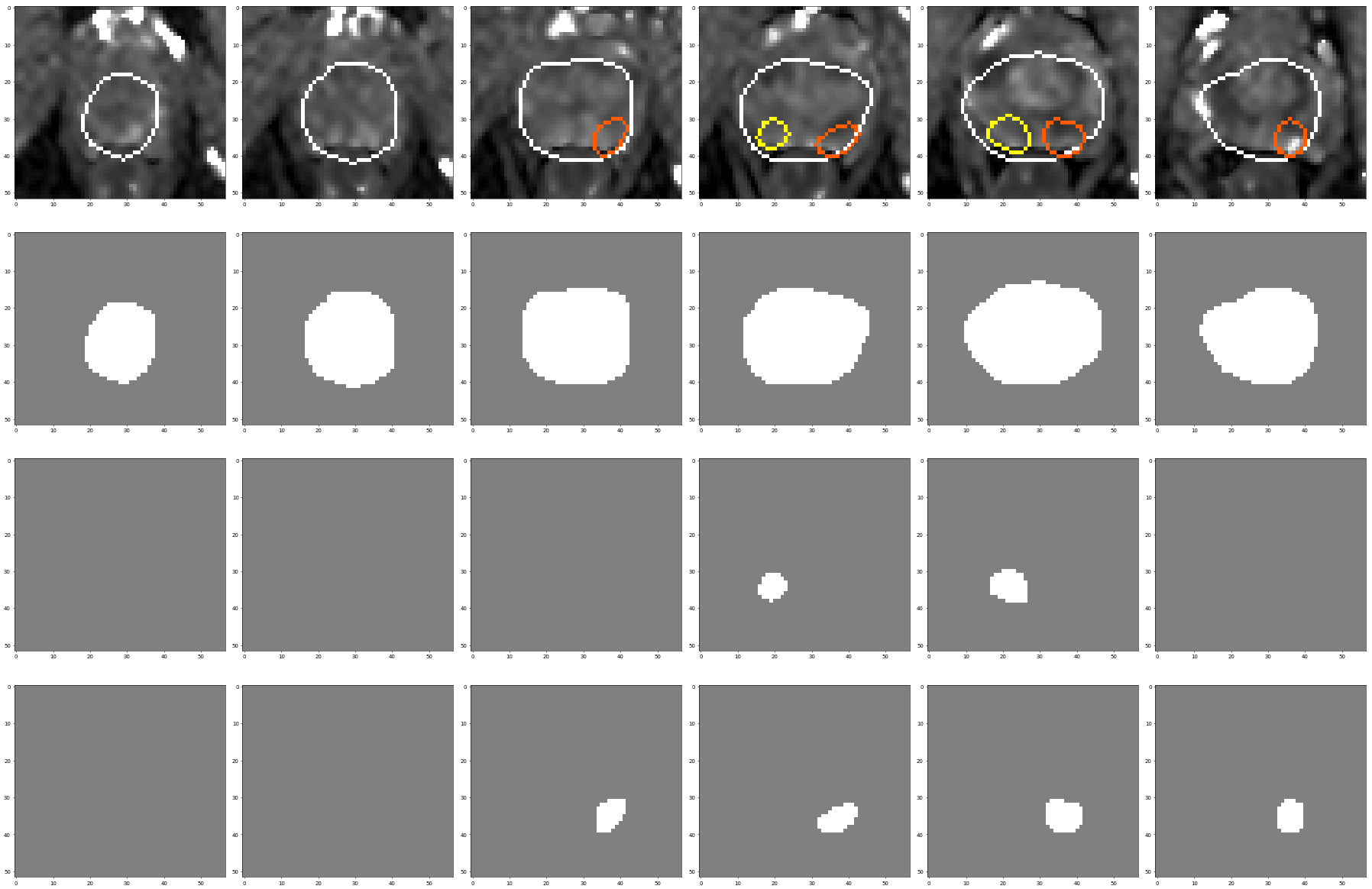

Supplement: S2 File — Files DWI-Mono-ADCm-xxx.png, T2-fitted-xxx.png, and T2w-std-xxx.png correspond to ADCm and T2 parametric maps, and T2-weighted images of each patient, respectively. On the first row of slices they show positions of regions of interest placed on the prostate cancer lesions (red, yellow) and around whole prostate (white). The prostate mask is on the second row, while the remaining rows are lesion masks. Files histology-xx.jpg contain the whole mount prostatectomy sections of each patient, with tumor outlines in green. Please note that identical MRI acquisition protocol has been used on all patients, including slice thickness. Here all prostate cancer masks are show with corresponding whole mount prostatectomy sections. (ZIP) [file pone.0217702.s002.zip › supporting_figures/DWI-Mono-ADCm-042.png]

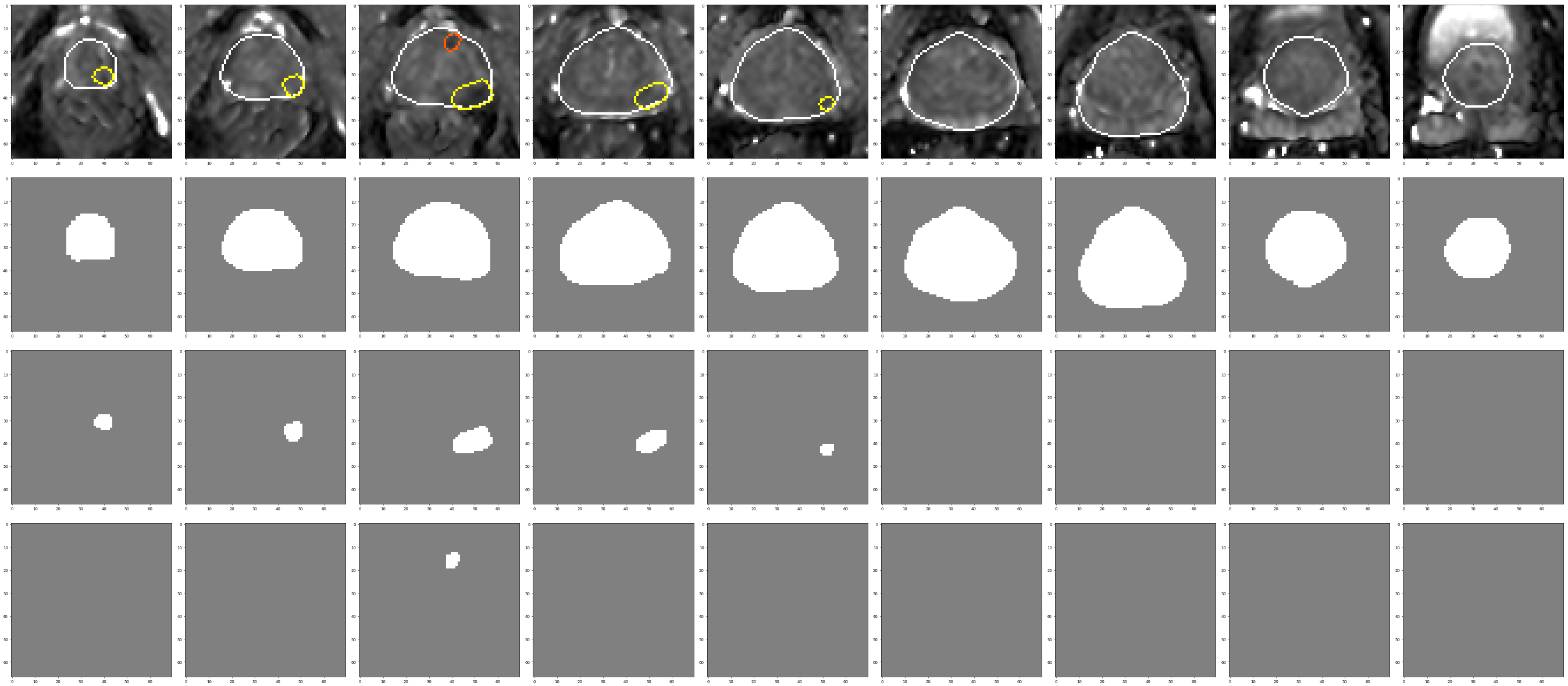

Supplement: S2 File — Files DWI-Mono-ADCm-xxx.png, T2-fitted-xxx.png, and T2w-std-xxx.png correspond to ADCm and T2 parametric maps, and T2-weighted images of each patient, respectively. On the first row of slices they show positions of regions of interest placed on the prostate cancer lesions (red, yellow) and around whole prostate (white). The prostate mask is on the second row, while the remaining rows are lesion masks. Files histology-xx.jpg contain the whole mount prostatectomy sections of each patient, with tumor outlines in green. Please note that identical MRI acquisition protocol has been used on all patients, including slice thickness. Here all prostate cancer masks are show with corresponding whole mount prostatectomy sections. (ZIP) [file pone.0217702.s002.zip › supporting_figures/DWI-Mono-ADCm-043.png]

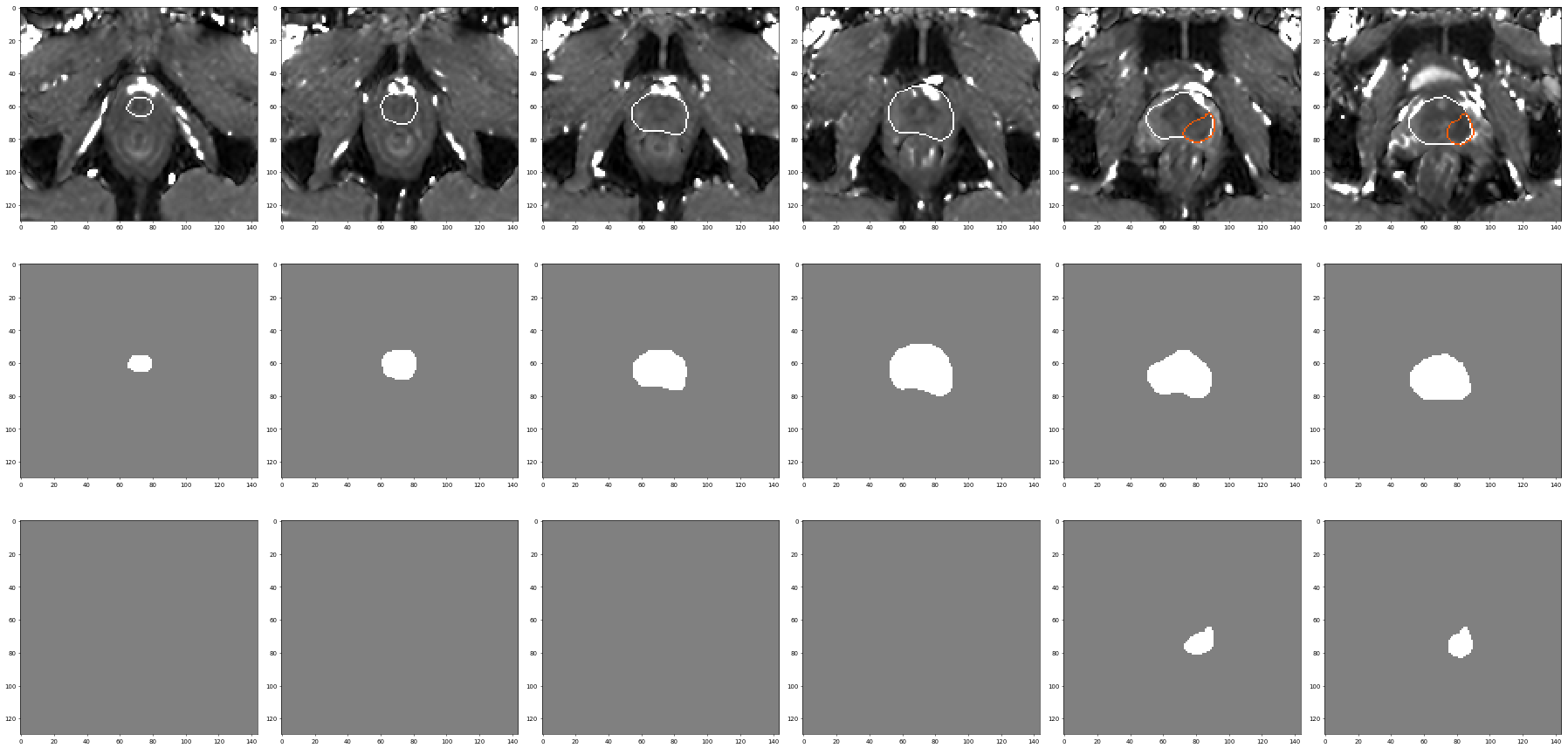

Supplement: S2 File — Files DWI-Mono-ADCm-xxx.png, T2-fitted-xxx.png, and T2w-std-xxx.png correspond to ADCm and T2 parametric maps, and T2-weighted images of each patient, respectively. On the first row of slices they show positions of regions of interest placed on the prostate cancer lesions (red, yellow) and around whole prostate (white). The prostate mask is on the second row, while the remaining rows are lesion masks. Files histology-xx.jpg contain the whole mount prostatectomy sections of each patient, with tumor outlines in green. Please note that identical MRI acquisition protocol has been used on all patients, including slice thickness. Here all prostate cancer masks are show with corresponding whole mount prostatectomy sections. (ZIP) [file pone.0217702.s002.zip › supporting_figures/DWI-Mono-ADCm-044.png]

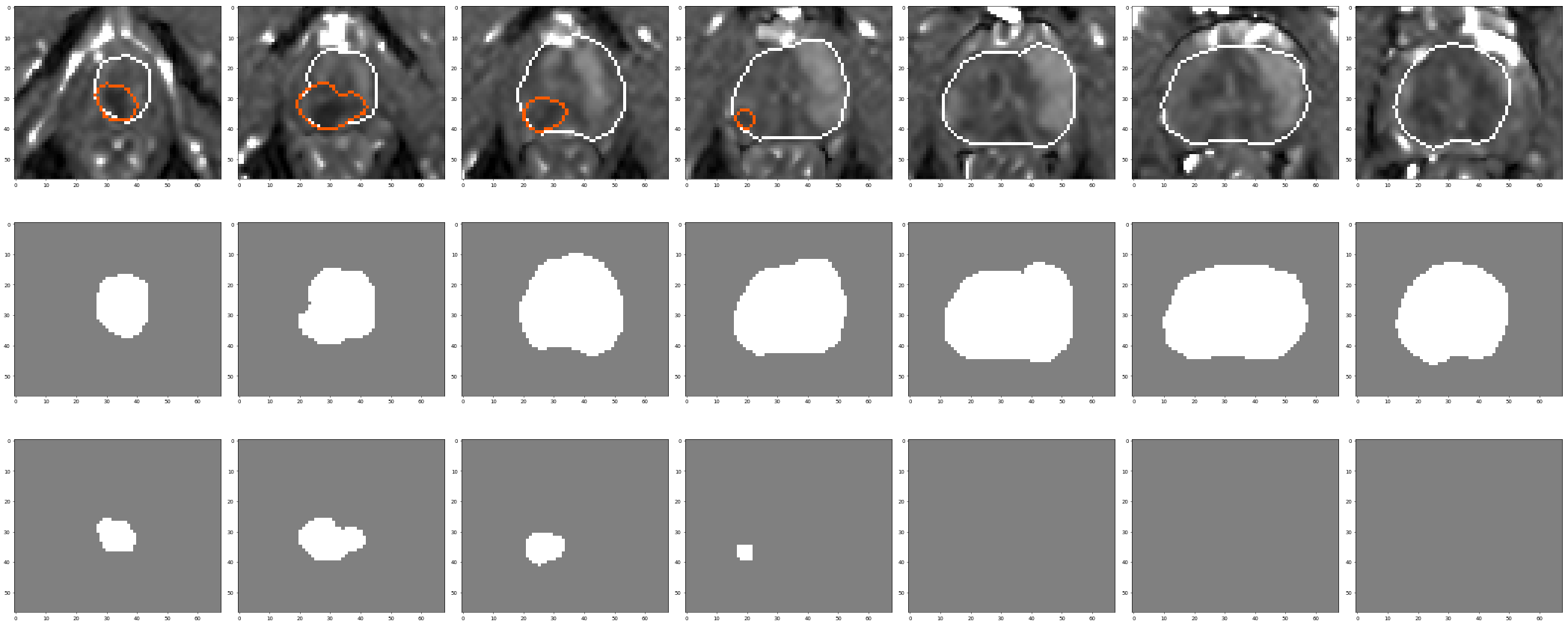

Supplement: S2 File — Files DWI-Mono-ADCm-xxx.png, T2-fitted-xxx.png, and T2w-std-xxx.png correspond to ADCm and T2 parametric maps, and T2-weighted images of each patient, respectively. On the first row of slices they show positions of regions of interest placed on the prostate cancer lesions (red, yellow) and around whole prostate (white). The prostate mask is on the second row, while the remaining rows are lesion masks. Files histology-xx.jpg contain the whole mount prostatectomy sections of each patient, with tumor outlines in green. Please note that identical MRI acquisition protocol has been used on all patients, including slice thickness. Here all prostate cancer masks are show with corresponding whole mount prostatectomy sections. (ZIP) [file pone.0217702.s002.zip › supporting_figures/DWI-Mono-ADCm-045.png]

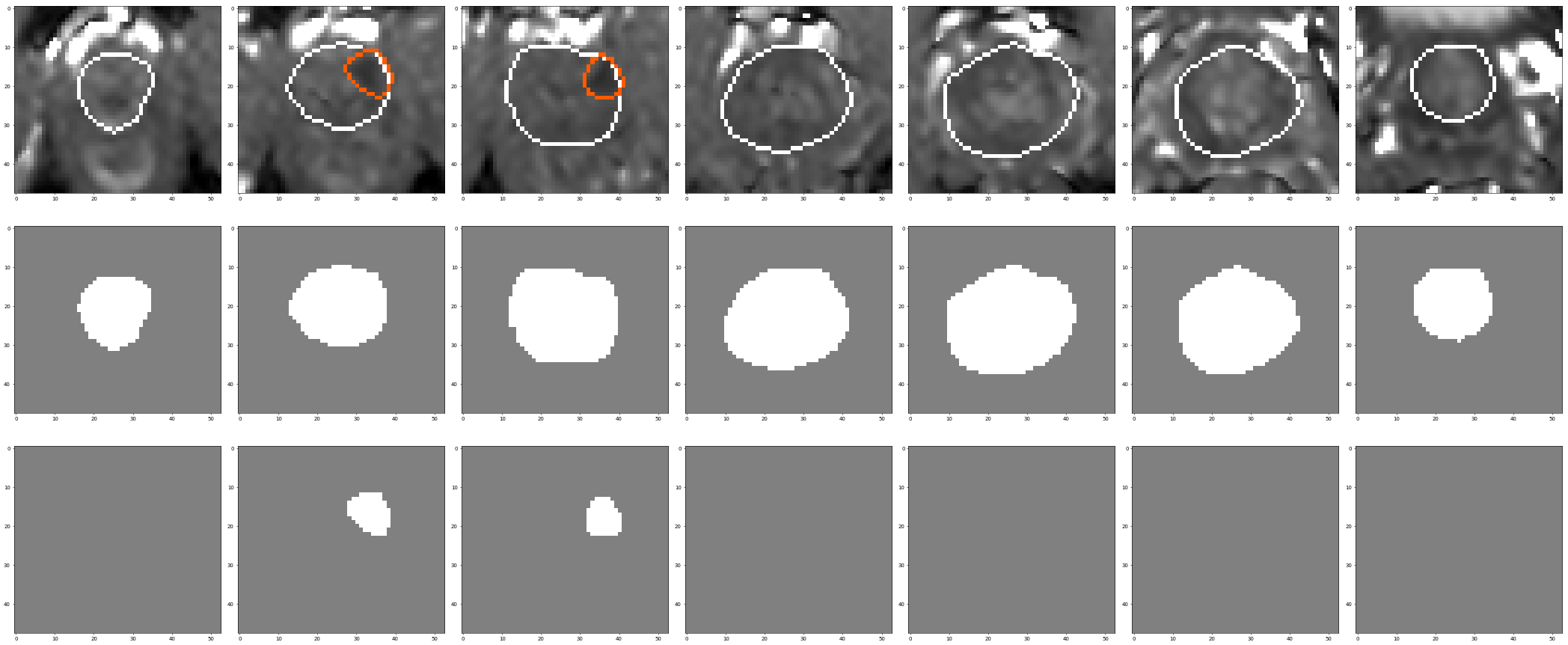

Supplement: S2 File — Files DWI-Mono-ADCm-xxx.png, T2-fitted-xxx.png, and T2w-std-xxx.png correspond to ADCm and T2 parametric maps, and T2-weighted images of each patient, respectively. On the first row of slices they show positions of regions of interest placed on the prostate cancer lesions (red, yellow) and around whole prostate (white). The prostate mask is on the second row, while the remaining rows are lesion masks. Files histology-xx.jpg contain the whole mount prostatectomy sections of each patient, with tumor outlines in green. Please note that identical MRI acquisition protocol has been used on all patients, including slice thickness. Here all prostate cancer masks are show with corresponding whole mount prostatectomy sections. (ZIP) [file pone.0217702.s002.zip › supporting_figures/DWI-Mono-ADCm-046.png]

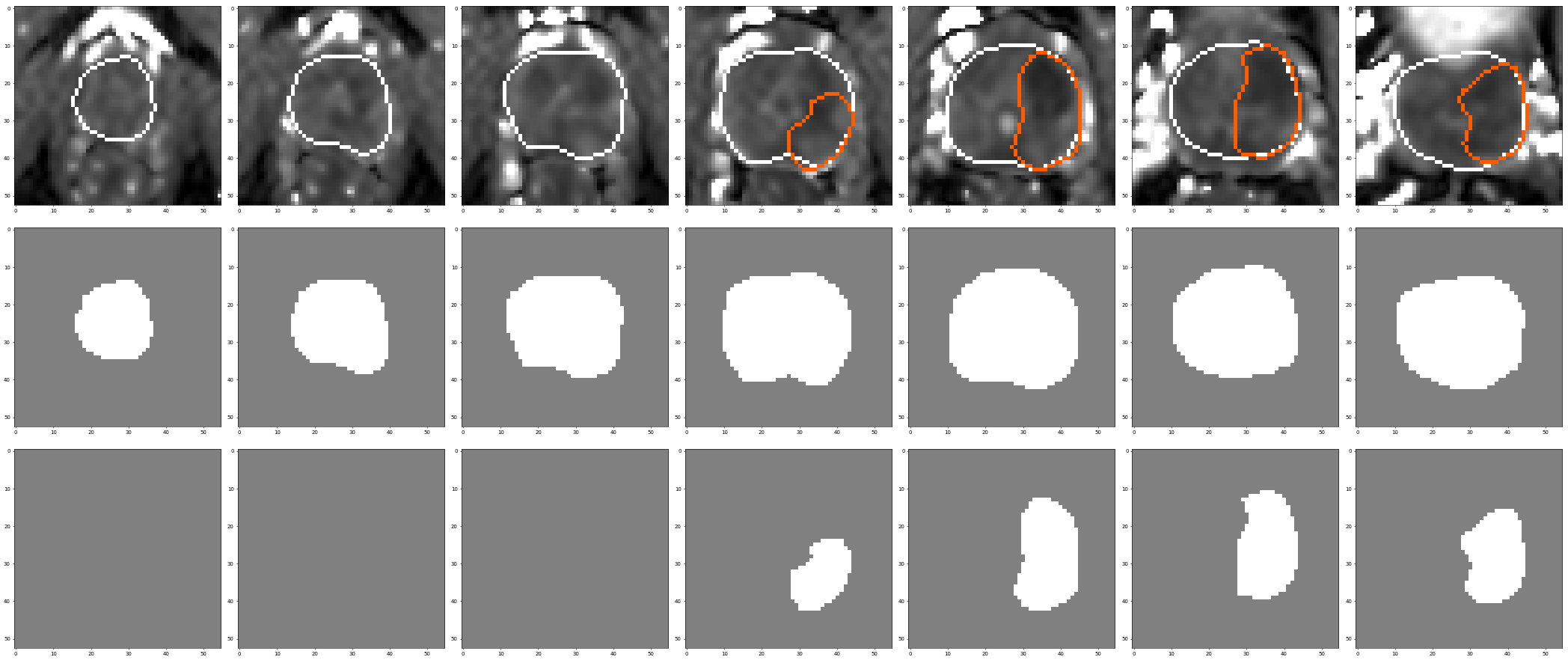

Supplement: S2 File — Files DWI-Mono-ADCm-xxx.png, T2-fitted-xxx.png, and T2w-std-xxx.png correspond to ADCm and T2 parametric maps, and T2-weighted images of each patient, respectively. On the first row of slices they show positions of regions of interest placed on the prostate cancer lesions (red, yellow) and around whole prostate (white). The prostate mask is on the second row, while the remaining rows are lesion masks. Files histology-xx.jpg contain the whole mount prostatectomy sections of each patient, with tumor outlines in green. Please note that identical MRI acquisition protocol has been used on all patients, including slice thickness. Here all prostate cancer masks are show with corresponding whole mount prostatectomy sections. (ZIP) [file pone.0217702.s002.zip › supporting_figures/DWI-Mono-ADCm-047.png]

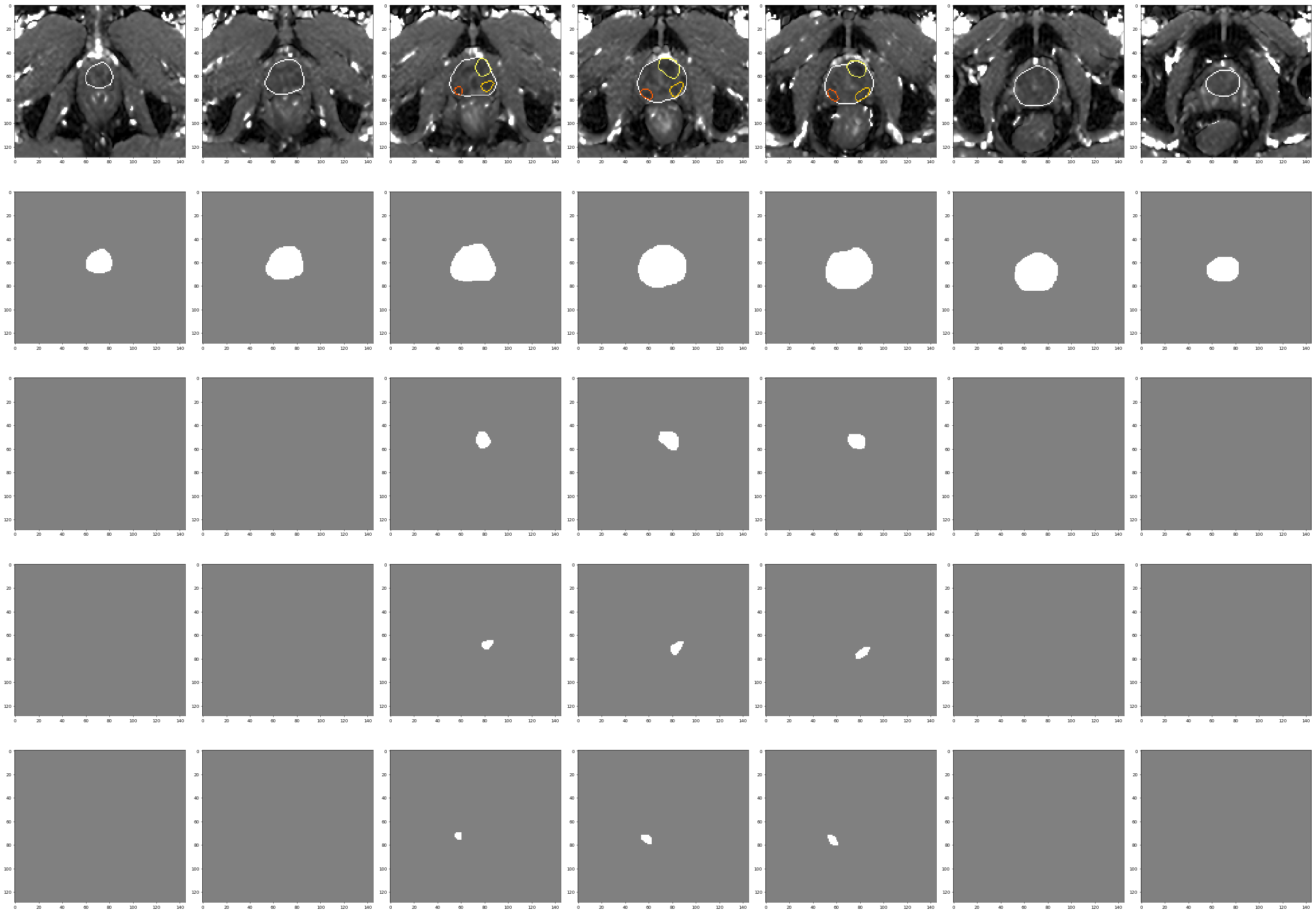

Supplement: S2 File — Files DWI-Mono-ADCm-xxx.png, T2-fitted-xxx.png, and T2w-std-xxx.png correspond to ADCm and T2 parametric maps, and T2-weighted images of each patient, respectively. On the first row of slices they show positions of regions of interest placed on the prostate cancer lesions (red, yellow) and around whole prostate (white). The prostate mask is on the second row, while the remaining rows are lesion masks. Files histology-xx.jpg contain the whole mount prostatectomy sections of each patient, with tumor outlines in green. Please note that identical MRI acquisition protocol has been used on all patients, including slice thickness. Here all prostate cancer masks are show with corresponding whole mount prostatectomy sections. (ZIP) [file pone.0217702.s002.zip › supporting_figures/DWI-Mono-ADCm-048.png]

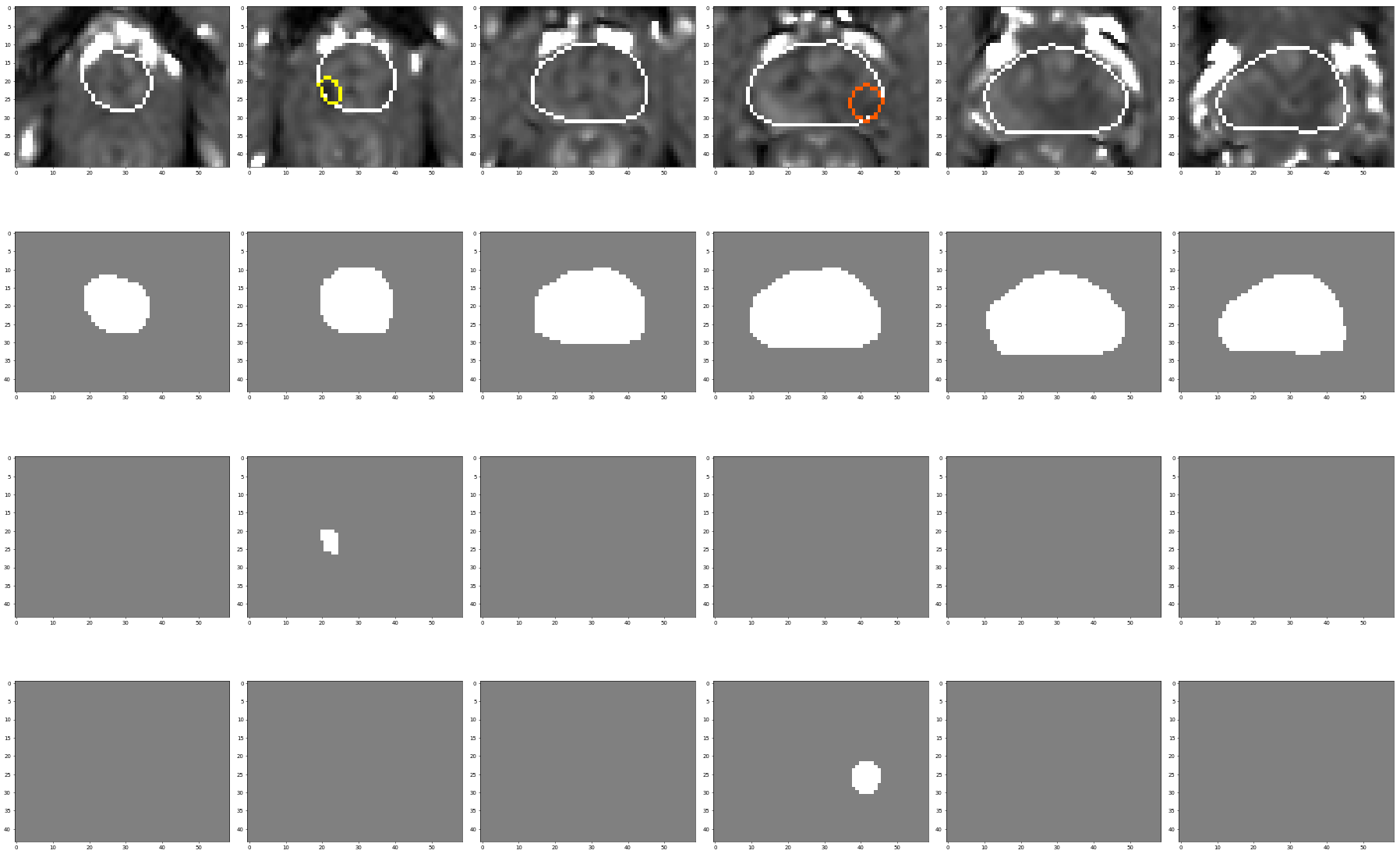

Supplement: S2 File — Files DWI-Mono-ADCm-xxx.png, T2-fitted-xxx.png, and T2w-std-xxx.png correspond to ADCm and T2 parametric maps, and T2-weighted images of each patient, respectively. On the first row of slices they show positions of regions of interest placed on the prostate cancer lesions (red, yellow) and around whole prostate (white). The prostate mask is on the second row, while the remaining rows are lesion masks. Files histology-xx.jpg contain the whole mount prostatectomy sections of each patient, with tumor outlines in green. Please note that identical MRI acquisition protocol has been used on all patients, including slice thickness. Here all prostate cancer masks are show with corresponding whole mount prostatectomy sections. (ZIP) [file pone.0217702.s002.zip › supporting_figures/DWI-Mono-ADCm-049.png]

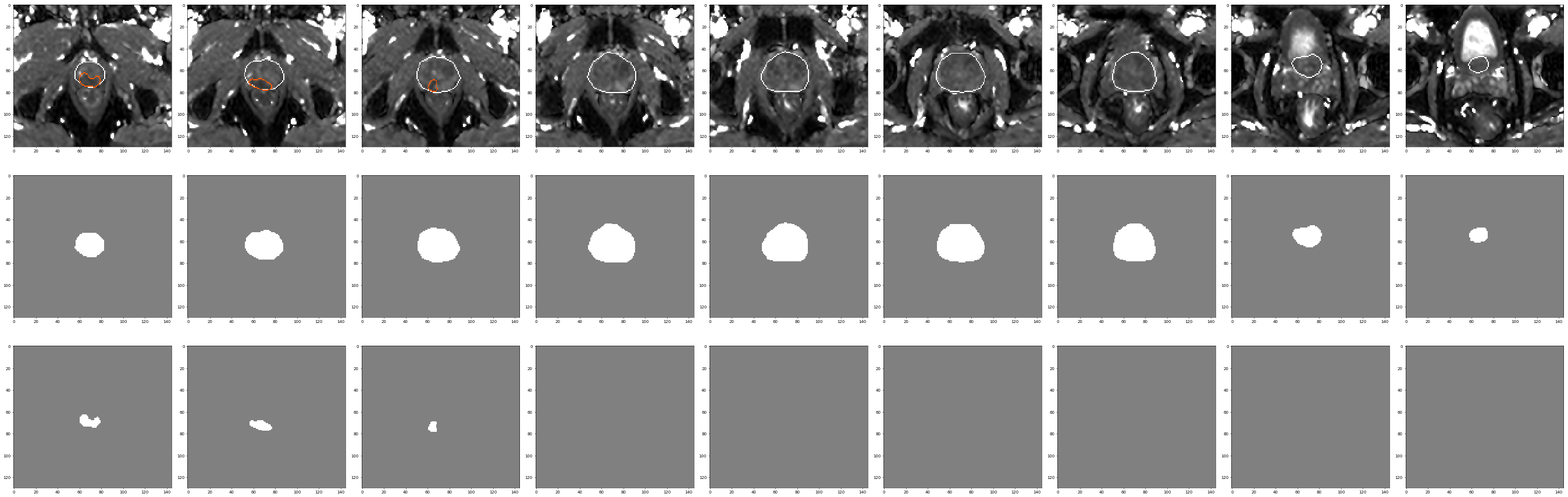

Supplement: S2 File — Files DWI-Mono-ADCm-xxx.png, T2-fitted-xxx.png, and T2w-std-xxx.png correspond to ADCm and T2 parametric maps, and T2-weighted images of each patient, respectively. On the first row of slices they show positions of regions of interest placed on the prostate cancer lesions (red, yellow) and around whole prostate (white). The prostate mask is on the second row, while the remaining rows are lesion masks. Files histology-xx.jpg contain the whole mount prostatectomy sections of each patient, with tumor outlines in green. Please note that identical MRI acquisition protocol has been used on all patients, including slice thickness. Here all prostate cancer masks are show with corresponding whole mount prostatectomy sections. (ZIP) [file pone.0217702.s002.zip › supporting_figures/DWI-Mono-ADCm-050.png]

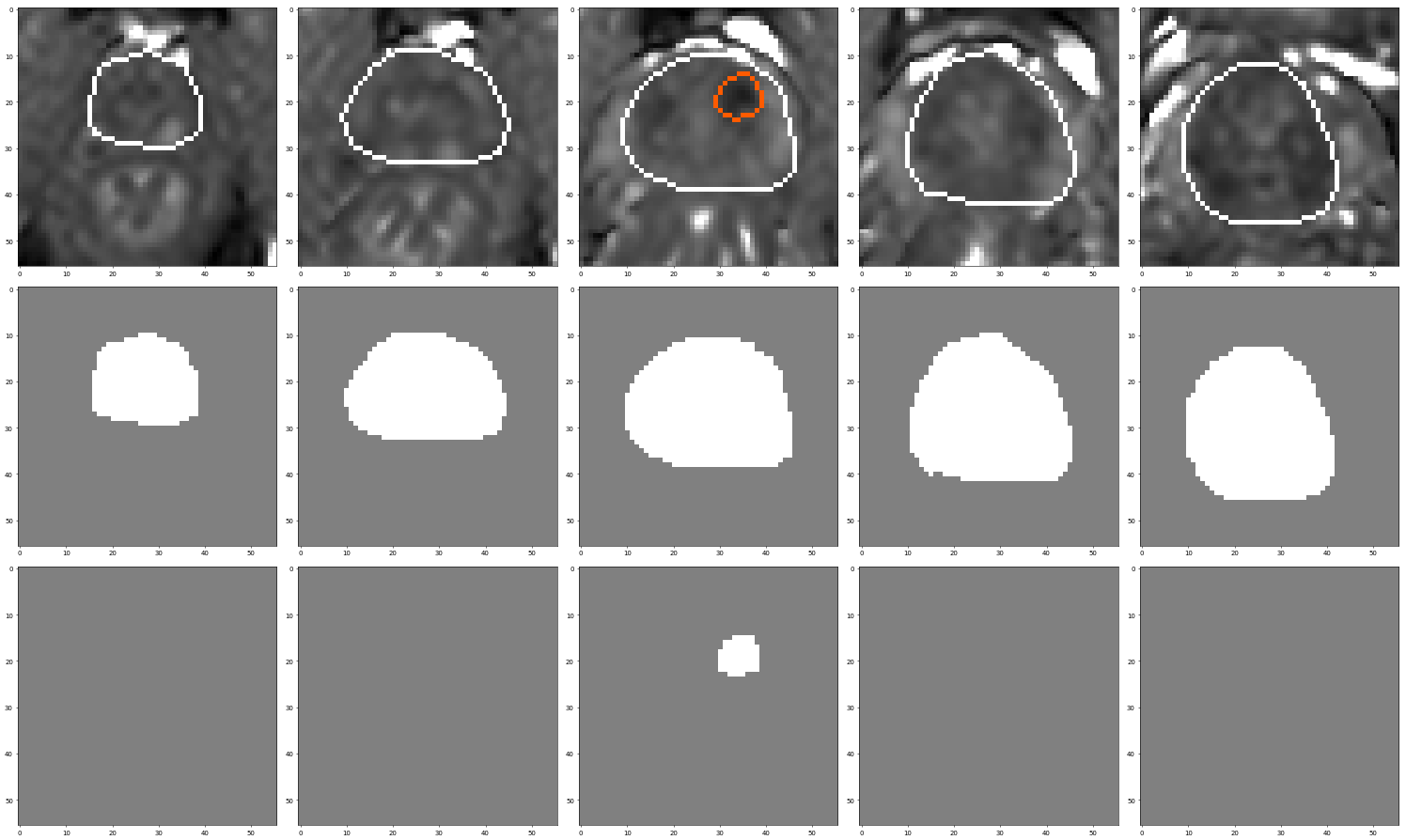

Supplement: S2 File — Files DWI-Mono-ADCm-xxx.png, T2-fitted-xxx.png, and T2w-std-xxx.png correspond to ADCm and T2 parametric maps, and T2-weighted images of each patient, respectively. On the first row of slices they show positions of regions of interest placed on the prostate cancer lesions (red, yellow) and around whole prostate (white). The prostate mask is on the second row, while the remaining rows are lesion masks. Files histology-xx.jpg contain the whole mount prostatectomy sections of each patient, with tumor outlines in green. Please note that identical MRI acquisition protocol has been used on all patients, including slice thickness. Here all prostate cancer masks are show with corresponding whole mount prostatectomy sections. (ZIP) [file pone.0217702.s002.zip › supporting_figures/DWI-Mono-ADCm-051.png]

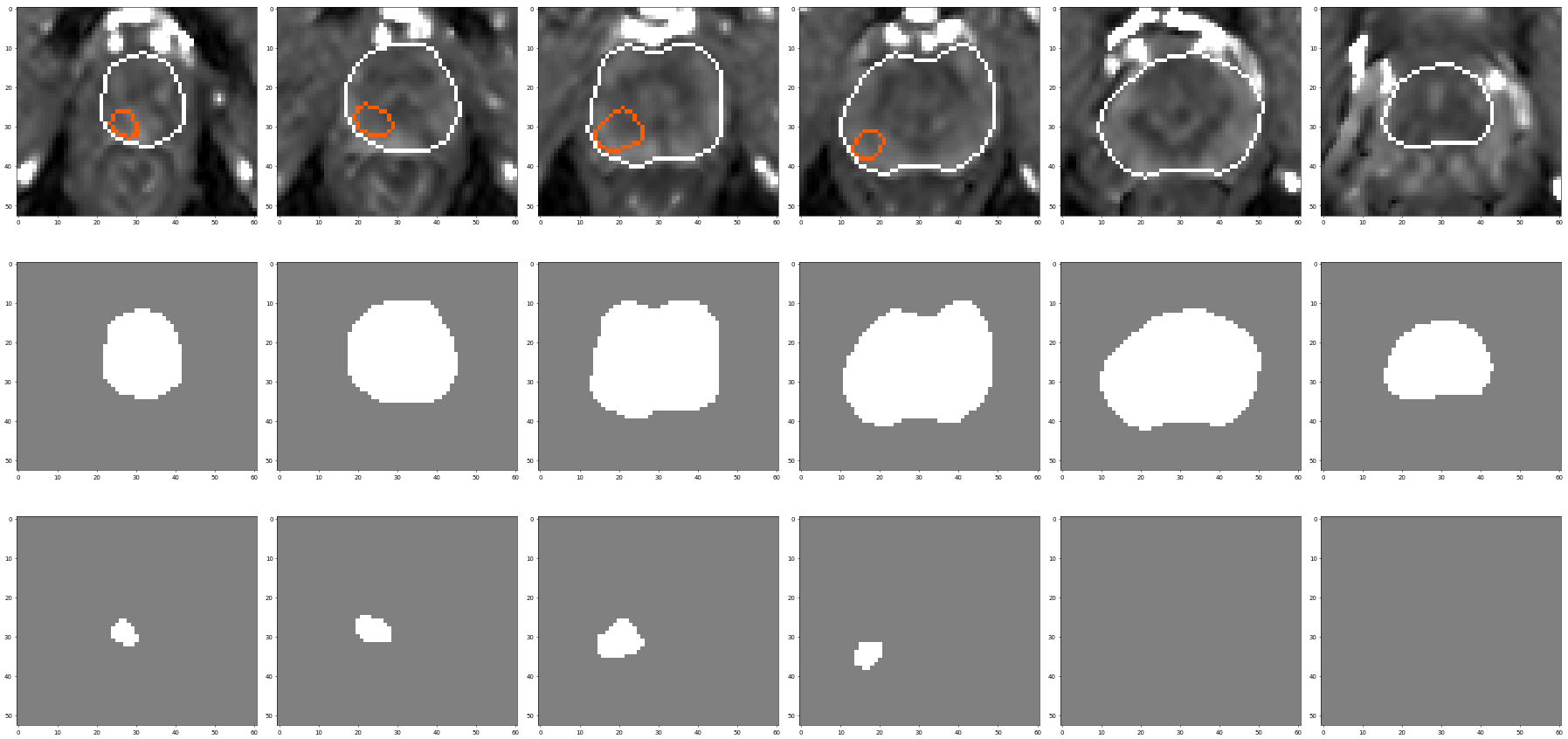

Supplement: S2 File — Files DWI-Mono-ADCm-xxx.png, T2-fitted-xxx.png, and T2w-std-xxx.png correspond to ADCm and T2 parametric maps, and T2-weighted images of each patient, respectively. On the first row of slices they show positions of regions of interest placed on the prostate cancer lesions (red, yellow) and around whole prostate (white). The prostate mask is on the second row, while the remaining rows are lesion masks. Files histology-xx.jpg contain the whole mount prostatectomy sections of each patient, with tumor outlines in green. Please note that identical MRI acquisition protocol has been used on all patients, including slice thickness. Here all prostate cancer masks are show with corresponding whole mount prostatectomy sections. (ZIP) [file pone.0217702.s002.zip › supporting_figures/DWI-Mono-ADCm-052.png]

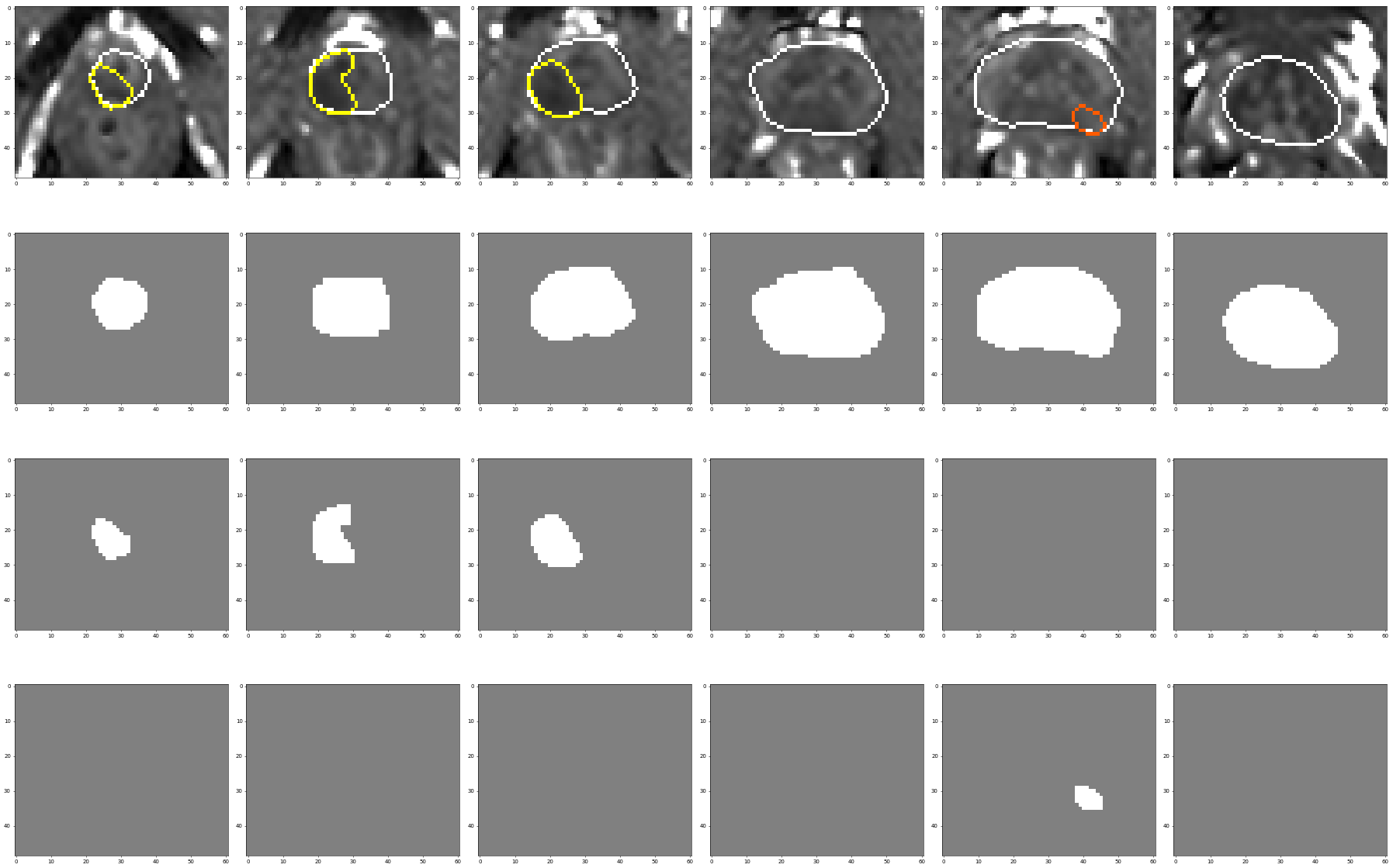

Supplement: S2 File — Files DWI-Mono-ADCm-xxx.png, T2-fitted-xxx.png, and T2w-std-xxx.png correspond to ADCm and T2 parametric maps, and T2-weighted images of each patient, respectively. On the first row of slices they show positions of regions of interest placed on the prostate cancer lesions (red, yellow) and around whole prostate (white). The prostate mask is on the second row, while the remaining rows are lesion masks. Files histology-xx.jpg contain the whole mount prostatectomy sections of each patient, with tumor outlines in green. Please note that identical MRI acquisition protocol has been used on all patients, including slice thickness. Here all prostate cancer masks are show with corresponding whole mount prostatectomy sections. (ZIP) [file pone.0217702.s002.zip › supporting_figures/DWI-Mono-ADCm-053.png]

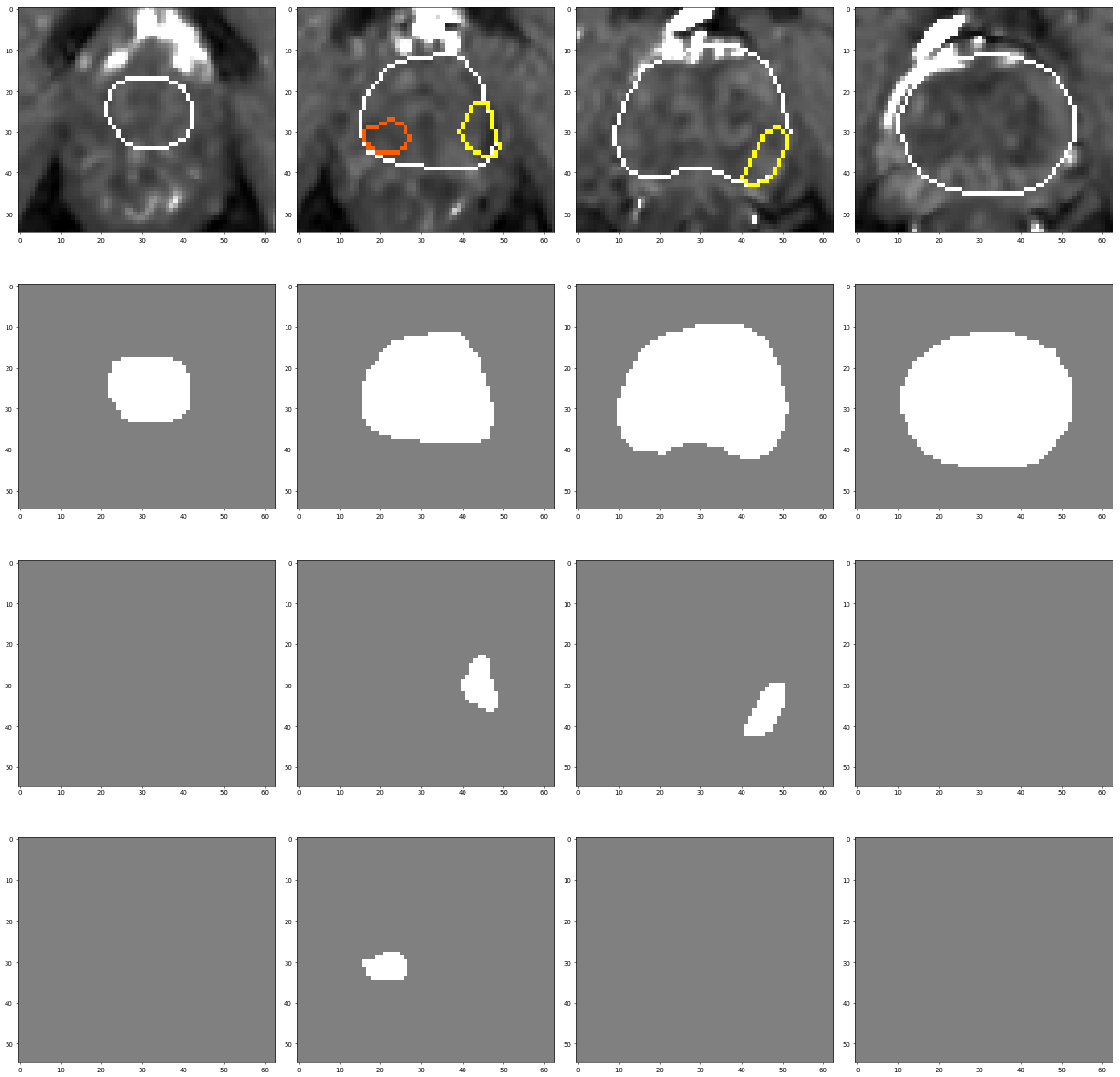

Supplement: S2 File — Files DWI-Mono-ADCm-xxx.png, T2-fitted-xxx.png, and T2w-std-xxx.png correspond to ADCm and T2 parametric maps, and T2-weighted images of each patient, respectively. On the first row of slices they show positions of regions of interest placed on the prostate cancer lesions (red, yellow) and around whole prostate (white). The prostate mask is on the second row, while the remaining rows are lesion masks. Files histology-xx.jpg contain the whole mount prostatectomy sections of each patient, with tumor outlines in green. Please note that identical MRI acquisition protocol has been used on all patients, including slice thickness. Here all prostate cancer masks are show with corresponding whole mount prostatectomy sections. (ZIP) [file pone.0217702.s002.zip › supporting_figures/DWI-Mono-ADCm-054.png]

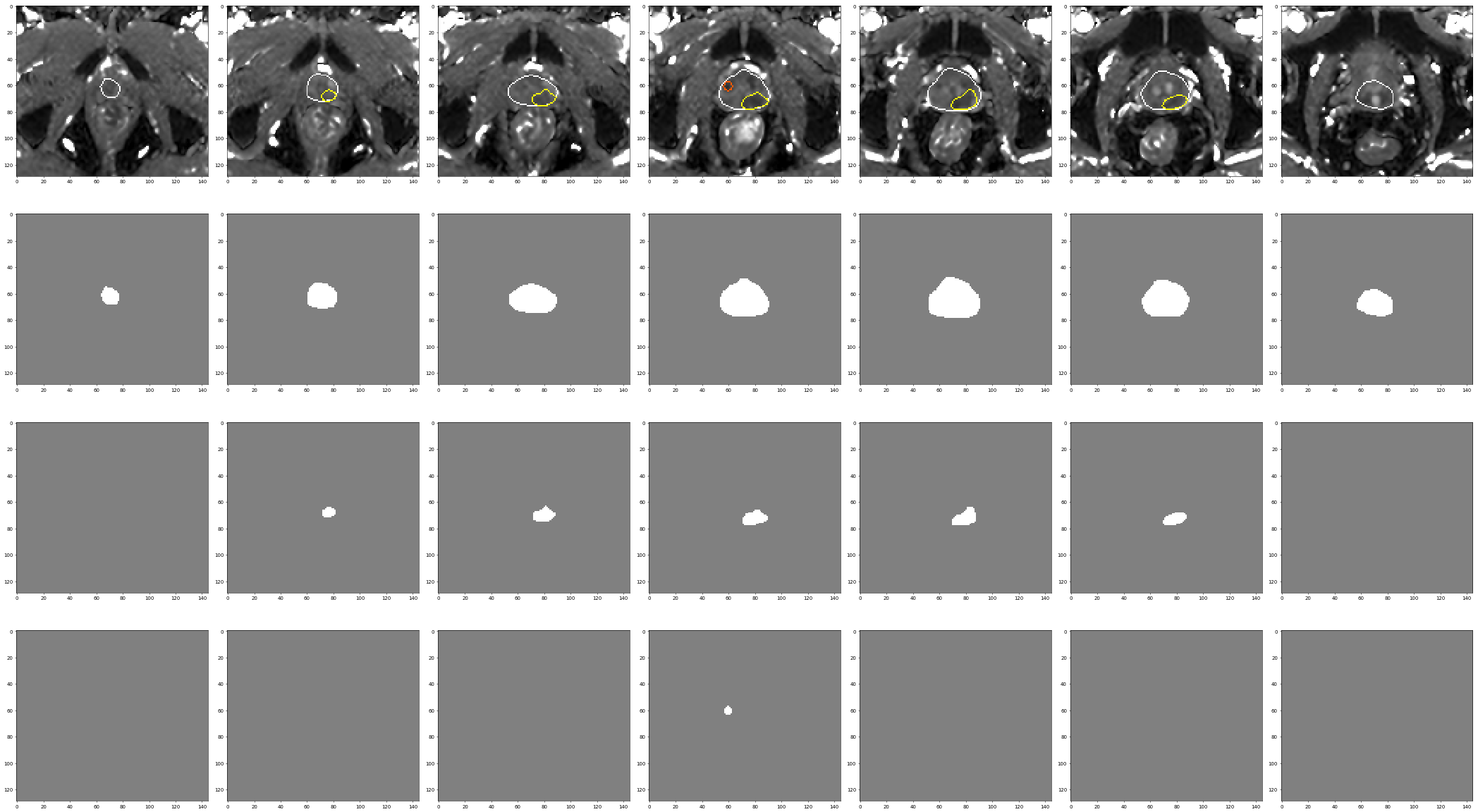

Supplement: S2 File — Files DWI-Mono-ADCm-xxx.png, T2-fitted-xxx.png, and T2w-std-xxx.png correspond to ADCm and T2 parametric maps, and T2-weighted images of each patient, respectively. On the first row of slices they show positions of regions of interest placed on the prostate cancer lesions (red, yellow) and around whole prostate (white). The prostate mask is on the second row, while the remaining rows are lesion masks. Files histology-xx.jpg contain the whole mount prostatectomy sections of each patient, with tumor outlines in green. Please note that identical MRI acquisition protocol has been used on all patients, including slice thickness. Here all prostate cancer masks are show with corresponding whole mount prostatectomy sections. (ZIP) [file pone.0217702.s002.zip › supporting_figures/DWI-Mono-ADCm-055.png]

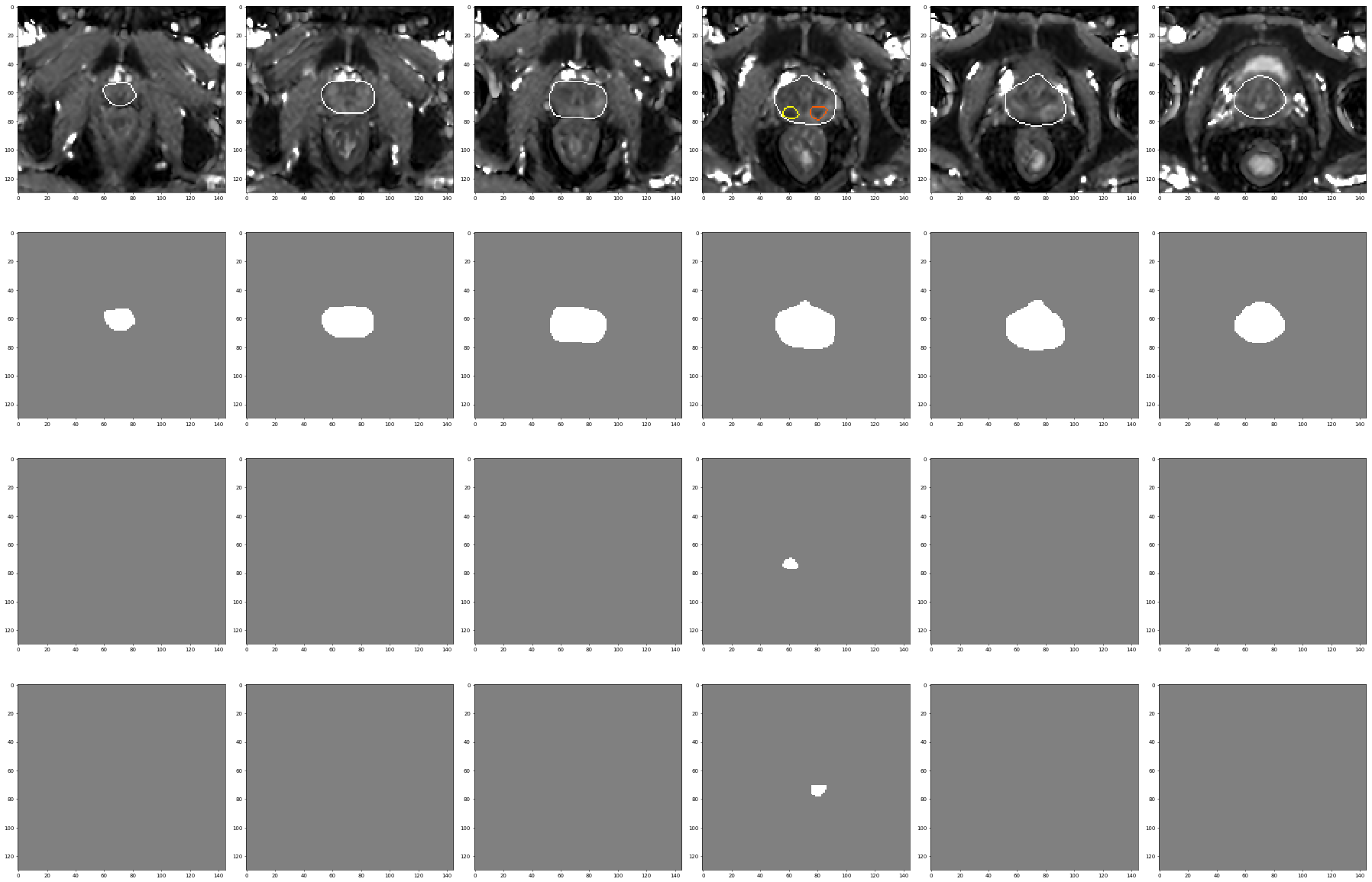

Supplement: S2 File — Files DWI-Mono-ADCm-xxx.png, T2-fitted-xxx.png, and T2w-std-xxx.png correspond to ADCm and T2 parametric maps, and T2-weighted images of each patient, respectively. On the first row of slices they show positions of regions of interest placed on the prostate cancer lesions (red, yellow) and around whole prostate (white). The prostate mask is on the second row, while the remaining rows are lesion masks. Files histology-xx.jpg contain the whole mount prostatectomy sections of each patient, with tumor outlines in green. Please note that identical MRI acquisition protocol has been used on all patients, including slice thickness. Here all prostate cancer masks are show with corresponding whole mount prostatectomy sections. (ZIP) [file pone.0217702.s002.zip › supporting_figures/DWI-Mono-ADCm-056.png]

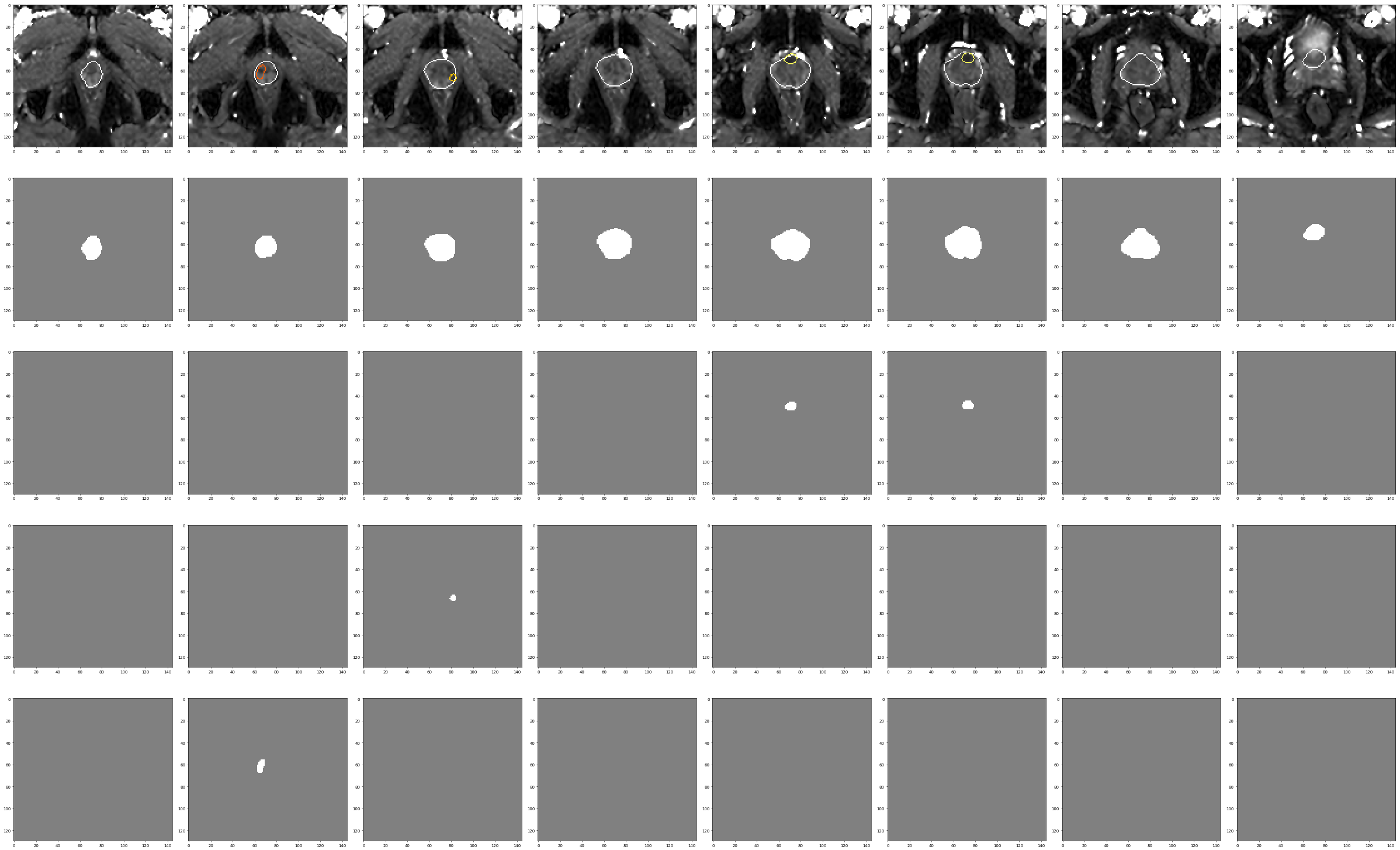

Supplement: S2 File — Files DWI-Mono-ADCm-xxx.png, T2-fitted-xxx.png, and T2w-std-xxx.png correspond to ADCm and T2 parametric maps, and T2-weighted images of each patient, respectively. On the first row of slices they show positions of regions of interest placed on the prostate cancer lesions (red, yellow) and around whole prostate (white). The prostate mask is on the second row, while the remaining rows are lesion masks. Files histology-xx.jpg contain the whole mount prostatectomy sections of each patient, with tumor outlines in green. Please note that identical MRI acquisition protocol has been used on all patients, including slice thickness. Here all prostate cancer masks are show with corresponding whole mount prostatectomy sections. (ZIP) [file pone.0217702.s002.zip › supporting_figures/DWI-Mono-ADCm-057.png]

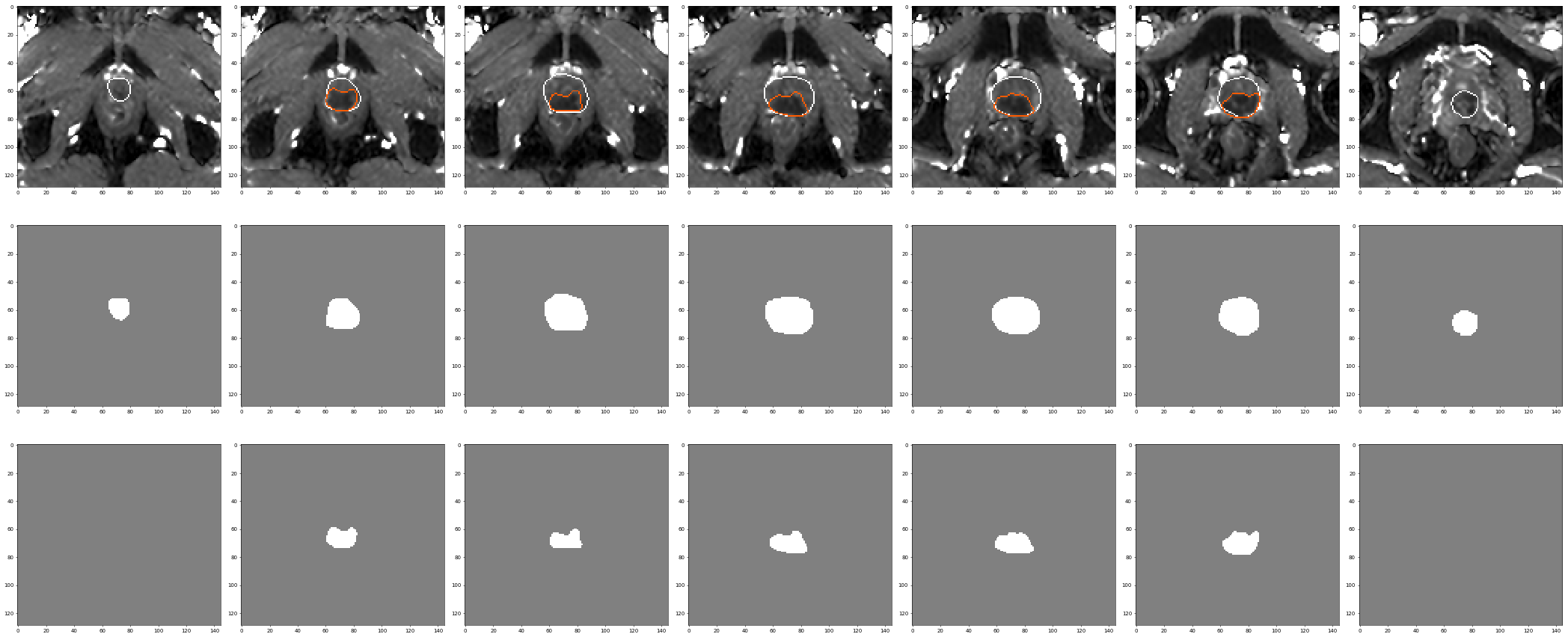

Supplement: S2 File — Files DWI-Mono-ADCm-xxx.png, T2-fitted-xxx.png, and T2w-std-xxx.png correspond to ADCm and T2 parametric maps, and T2-weighted images of each patient, respectively. On the first row of slices they show positions of regions of interest placed on the prostate cancer lesions (red, yellow) and around whole prostate (white). The prostate mask is on the second row, while the remaining rows are lesion masks. Files histology-xx.jpg contain the whole mount prostatectomy sections of each patient, with tumor outlines in green. Please note that identical MRI acquisition protocol has been used on all patients, including slice thickness. Here all prostate cancer masks are show with corresponding whole mount prostatectomy sections. (ZIP) [file pone.0217702.s002.zip › supporting_figures/DWI-Mono-ADCm-058.png]

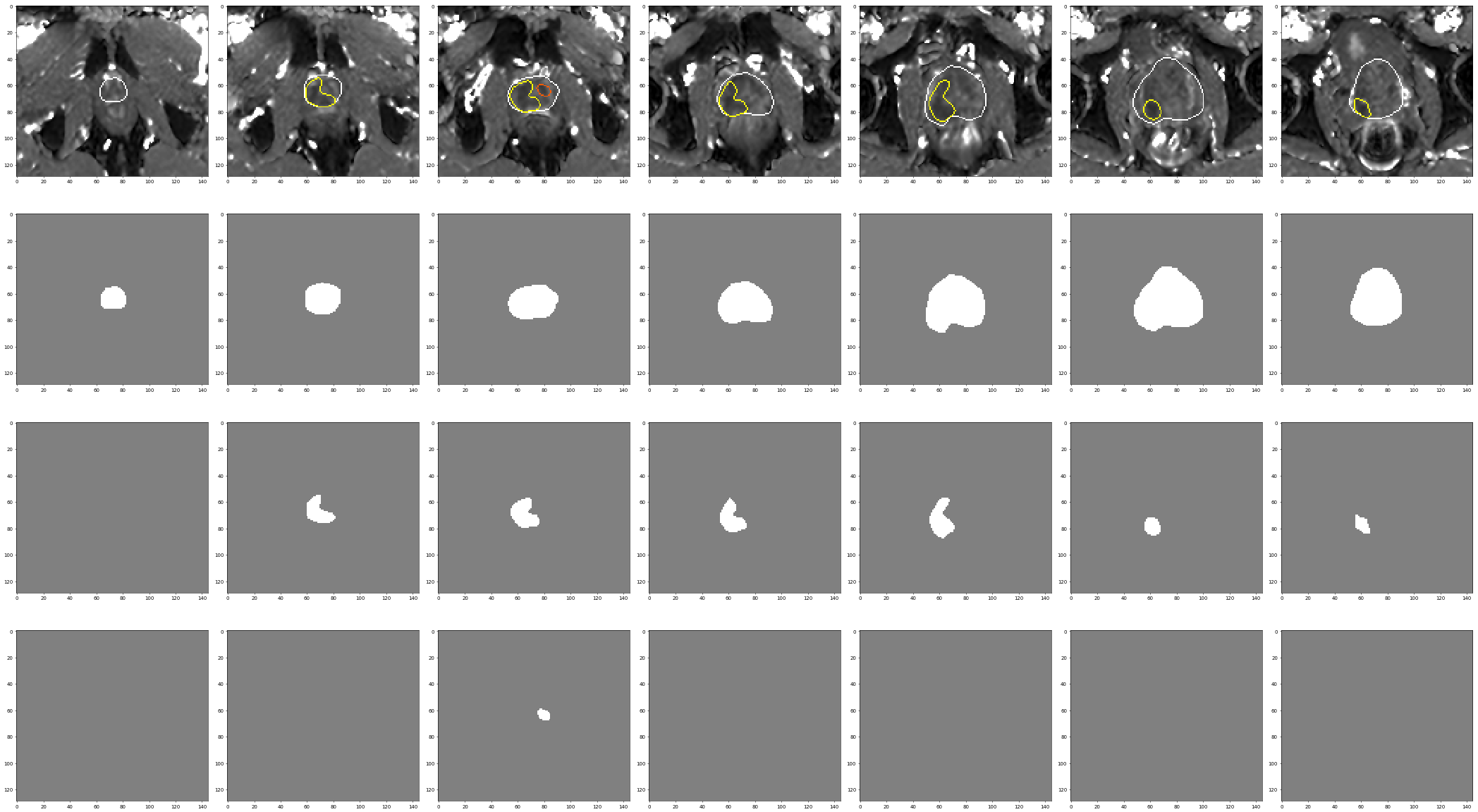

Supplement: S2 File — Files DWI-Mono-ADCm-xxx.png, T2-fitted-xxx.png, and T2w-std-xxx.png correspond to ADCm and T2 parametric maps, and T2-weighted images of each patient, respectively. On the first row of slices they show positions of regions of interest placed on the prostate cancer lesions (red, yellow) and around whole prostate (white). The prostate mask is on the second row, while the remaining rows are lesion masks. Files histology-xx.jpg contain the whole mount prostatectomy sections of each patient, with tumor outlines in green. Please note that identical MRI acquisition protocol has been used on all patients, including slice thickness. Here all prostate cancer masks are show with corresponding whole mount prostatectomy sections. (ZIP) [file pone.0217702.s002.zip › supporting_figures/DWI-Mono-ADCm-059.png]

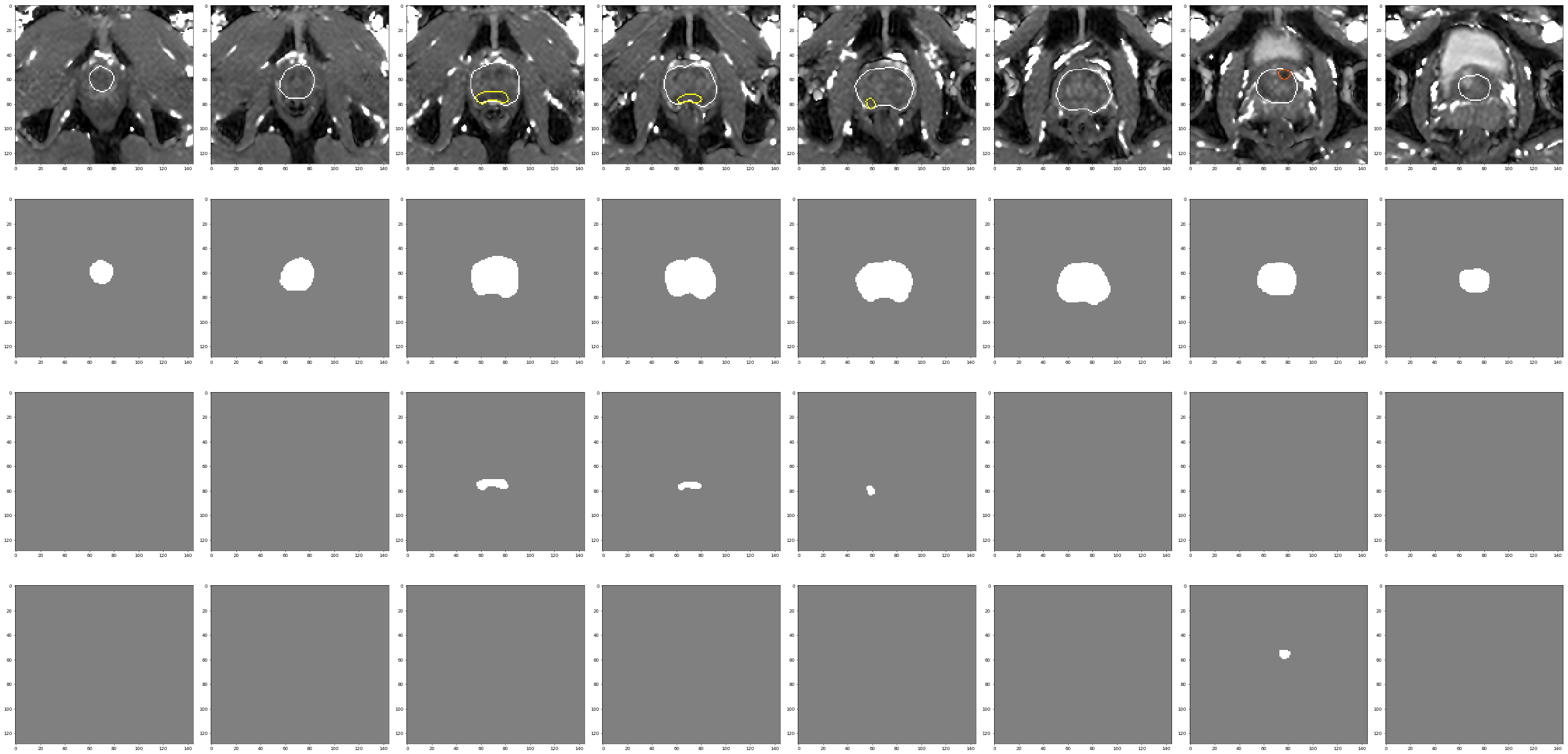

Supplement: S2 File — Files DWI-Mono-ADCm-xxx.png, T2-fitted-xxx.png, and T2w-std-xxx.png correspond to ADCm and T2 parametric maps, and T2-weighted images of each patient, respectively. On the first row of slices they show positions of regions of interest placed on the prostate cancer lesions (red, yellow) and around whole prostate (white). The prostate mask is on the second row, while the remaining rows are lesion masks. Files histology-xx.jpg contain the whole mount prostatectomy sections of each patient, with tumor outlines in green. Please note that identical MRI acquisition protocol has been used on all patients, including slice thickness. Here all prostate cancer masks are show with corresponding whole mount prostatectomy sections. (ZIP) [file pone.0217702.s002.zip › supporting_figures/DWI-Mono-ADCm-060.png]

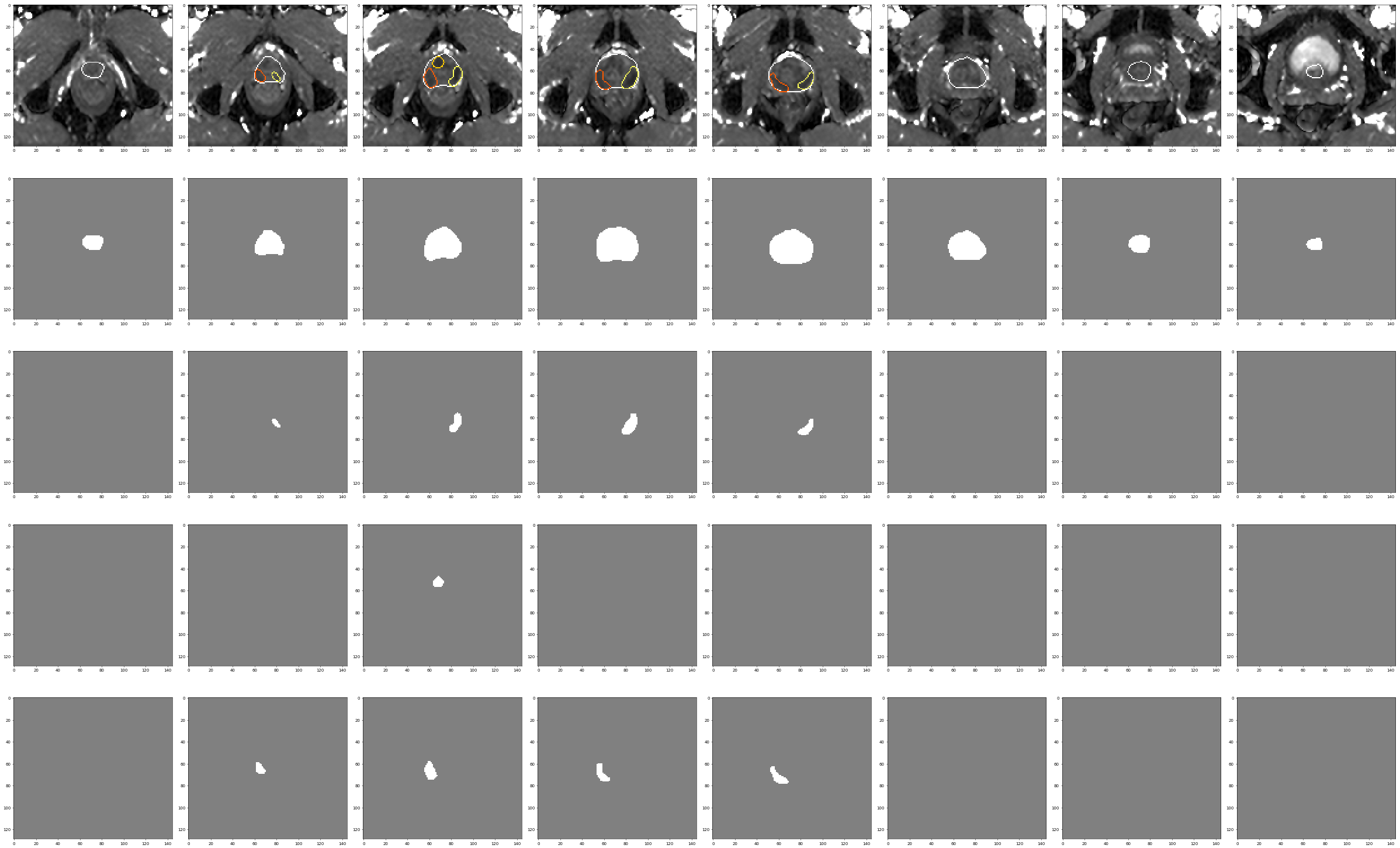

Supplement: S2 File — Files DWI-Mono-ADCm-xxx.png, T2-fitted-xxx.png, and T2w-std-xxx.png correspond to ADCm and T2 parametric maps, and T2-weighted images of each patient, respectively. On the first row of slices they show positions of regions of interest placed on the prostate cancer lesions (red, yellow) and around whole prostate (white). The prostate mask is on the second row, while the remaining rows are lesion masks. Files histology-xx.jpg contain the whole mount prostatectomy sections of each patient, with tumor outlines in green. Please note that identical MRI acquisition protocol has been used on all patients, including slice thickness. Here all prostate cancer masks are show with corresponding whole mount prostatectomy sections. (ZIP) [file pone.0217702.s002.zip › supporting_figures/DWI-Mono-ADCm-061.png]

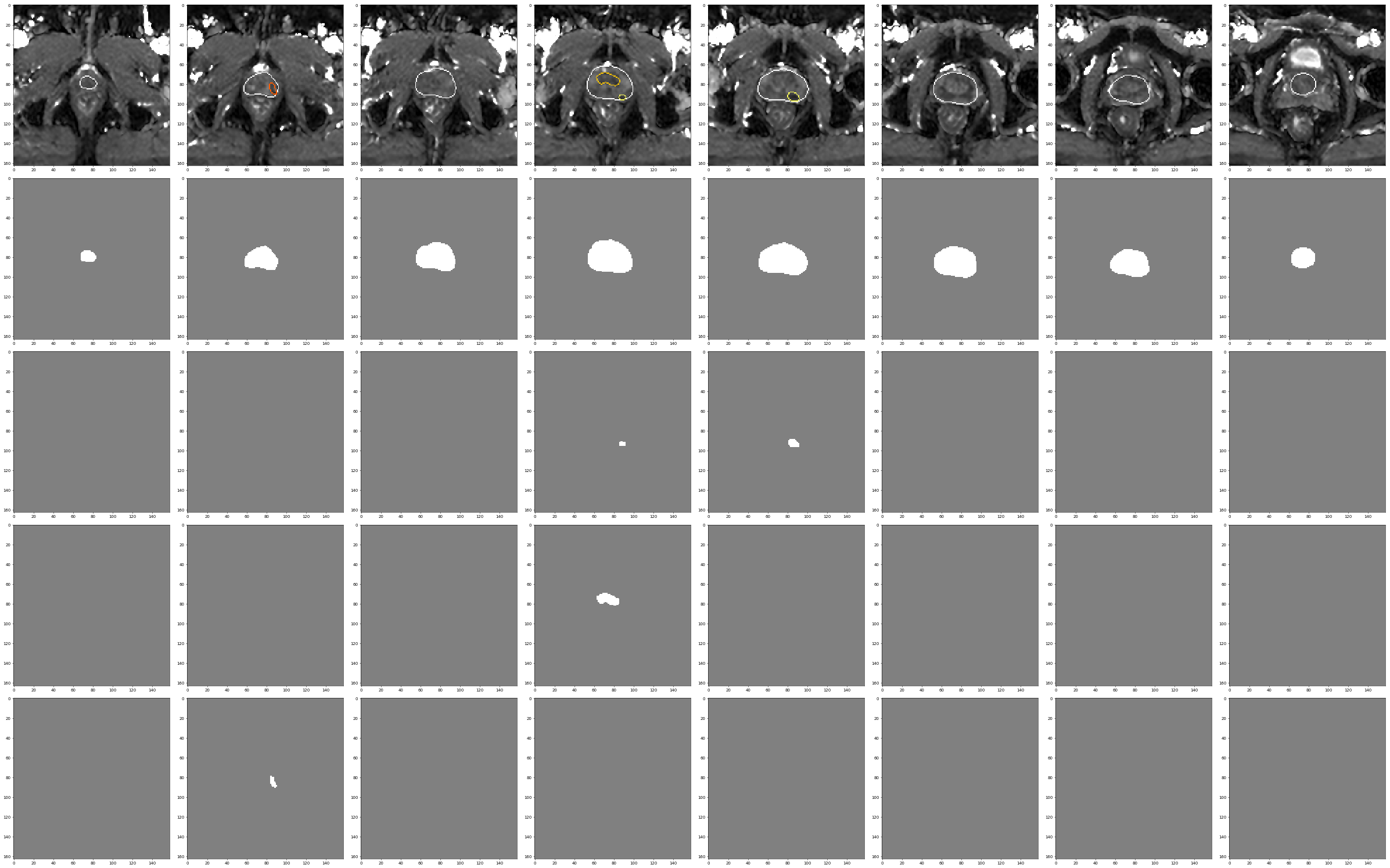

Supplement: S2 File — Files DWI-Mono-ADCm-xxx.png, T2-fitted-xxx.png, and T2w-std-xxx.png correspond to ADCm and T2 parametric maps, and T2-weighted images of each patient, respectively. On the first row of slices they show positions of regions of interest placed on the prostate cancer lesions (red, yellow) and around whole prostate (white). The prostate mask is on the second row, while the remaining rows are lesion masks. Files histology-xx.jpg contain the whole mount prostatectomy sections of each patient, with tumor outlines in green. Please note that identical MRI acquisition protocol has been used on all patients, including slice thickness. Here all prostate cancer masks are show with corresponding whole mount prostatectomy sections. (ZIP) [file pone.0217702.s002.zip › supporting_figures/DWI-Mono-ADCm-062.png]

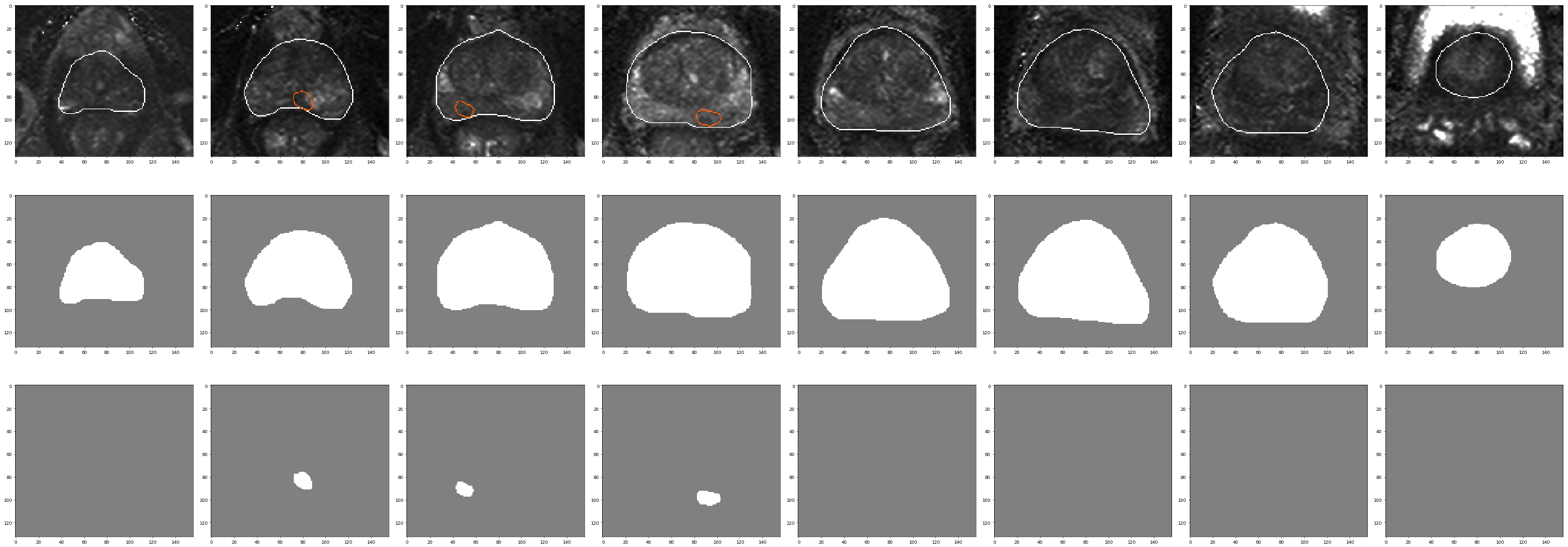

Supplement: S2 File — Files DWI-Mono-ADCm-xxx.png, T2-fitted-xxx.png, and T2w-std-xxx.png correspond to ADCm and T2 parametric maps, and T2-weighted images of each patient, respectively. On the first row of slices they show positions of regions of interest placed on the prostate cancer lesions (red, yellow) and around whole prostate (white). The prostate mask is on the second row, while the remaining rows are lesion masks. Files histology-xx.jpg contain the whole mount prostatectomy sections of each patient, with tumor outlines in green. Please note that identical MRI acquisition protocol has been used on all patients, including slice thickness. Here all prostate cancer masks are show with corresponding whole mount prostatectomy sections. (ZIP) [file pone.0217702.s002.zip › supporting_figures/T2-fitted-001.png]

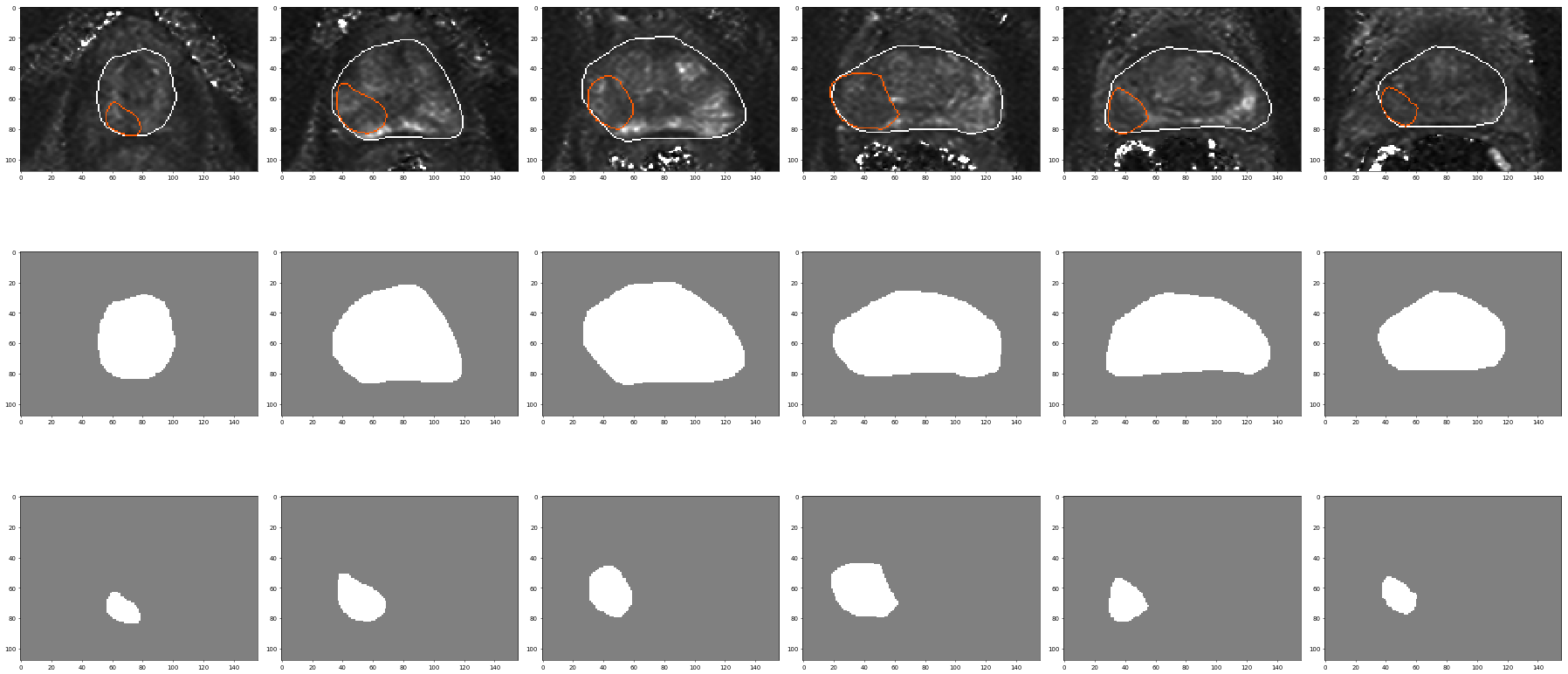

Supplement: S2 File — Files DWI-Mono-ADCm-xxx.png, T2-fitted-xxx.png, and T2w-std-xxx.png correspond to ADCm and T2 parametric maps, and T2-weighted images of each patient, respectively. On the first row of slices they show positions of regions of interest placed on the prostate cancer lesions (red, yellow) and around whole prostate (white). The prostate mask is on the second row, while the remaining rows are lesion masks. Files histology-xx.jpg contain the whole mount prostatectomy sections of each patient, with tumor outlines in green. Please note that identical MRI acquisition protocol has been used on all patients, including slice thickness. Here all prostate cancer masks are show with corresponding whole mount prostatectomy sections. (ZIP) [file pone.0217702.s002.zip › supporting_figures/T2-fitted-002.png]

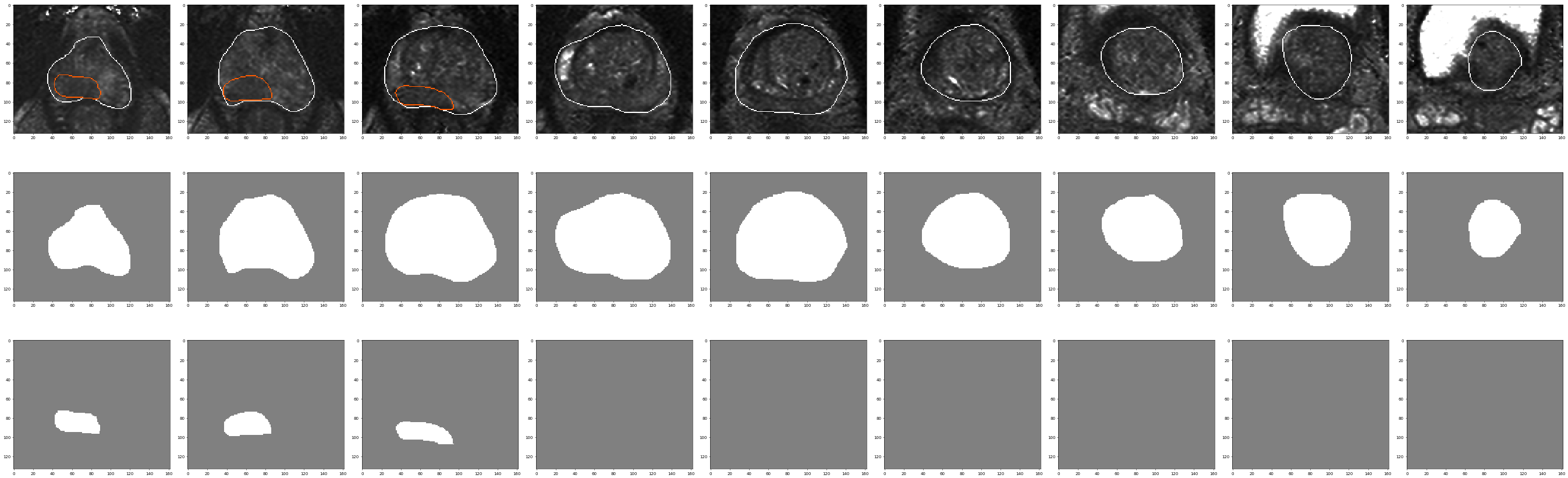

Supplement: S2 File — Files DWI-Mono-ADCm-xxx.png, T2-fitted-xxx.png, and T2w-std-xxx.png correspond to ADCm and T2 parametric maps, and T2-weighted images of each patient, respectively. On the first row of slices they show positions of regions of interest placed on the prostate cancer lesions (red, yellow) and around whole prostate (white). The prostate mask is on the second row, while the remaining rows are lesion masks. Files histology-xx.jpg contain the whole mount prostatectomy sections of each patient, with tumor outlines in green. Please note that identical MRI acquisition protocol has been used on all patients, including slice thickness. Here all prostate cancer masks are show with corresponding whole mount prostatectomy sections. (ZIP) [file pone.0217702.s002.zip › supporting_figures/T2-fitted-003.png]

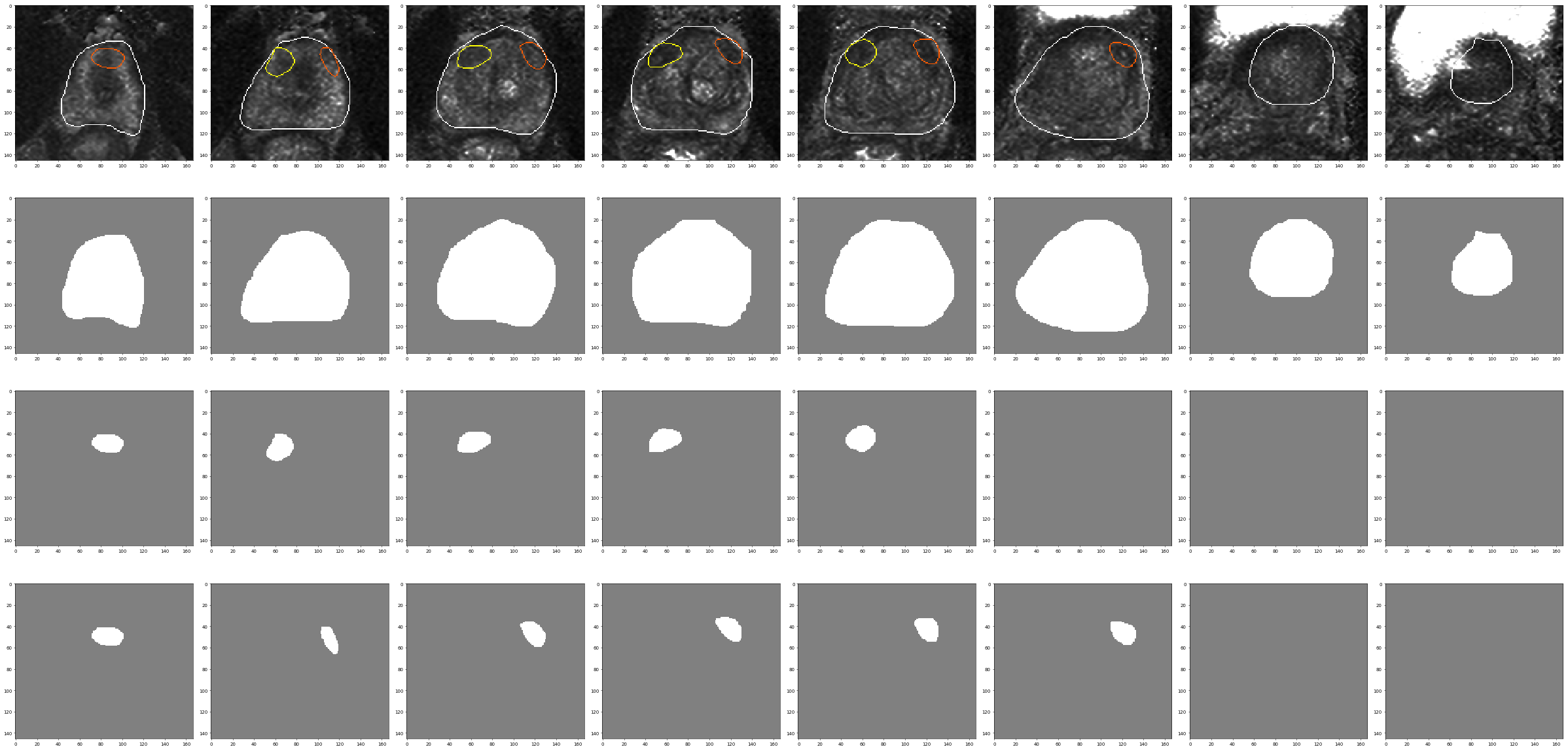

Supplement: S2 File — Files DWI-Mono-ADCm-xxx.png, T2-fitted-xxx.png, and T2w-std-xxx.png correspond to ADCm and T2 parametric maps, and T2-weighted images of each patient, respectively. On the first row of slices they show positions of regions of interest placed on the prostate cancer lesions (red, yellow) and around whole prostate (white). The prostate mask is on the second row, while the remaining rows are lesion masks. Files histology-xx.jpg contain the whole mount prostatectomy sections of each patient, with tumor outlines in green. Please note that identical MRI acquisition protocol has been used on all patients, including slice thickness. Here all prostate cancer masks are show with corresponding whole mount prostatectomy sections. (ZIP) [file pone.0217702.s002.zip › supporting_figures/T2-fitted-004.png]

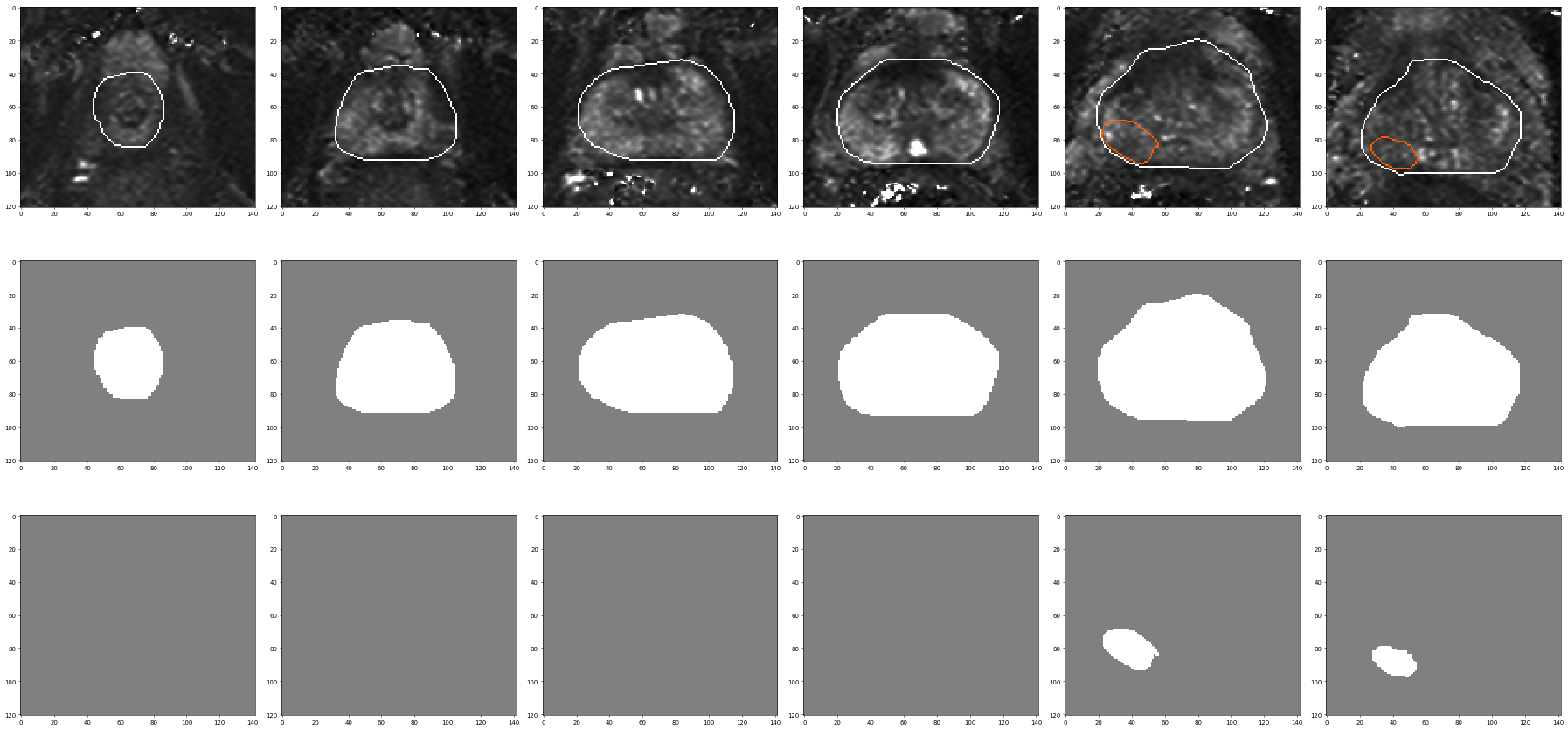

Supplement: S2 File — Files DWI-Mono-ADCm-xxx.png, T2-fitted-xxx.png, and T2w-std-xxx.png correspond to ADCm and T2 parametric maps, and T2-weighted images of each patient, respectively. On the first row of slices they show positions of regions of interest placed on the prostate cancer lesions (red, yellow) and around whole prostate (white). The prostate mask is on the second row, while the remaining rows are lesion masks. Files histology-xx.jpg contain the whole mount prostatectomy sections of each patient, with tumor outlines in green. Please note that identical MRI acquisition protocol has been used on all patients, including slice thickness. Here all prostate cancer masks are show with corresponding whole mount prostatectomy sections. (ZIP) [file pone.0217702.s002.zip › supporting_figures/T2-fitted-005.png]

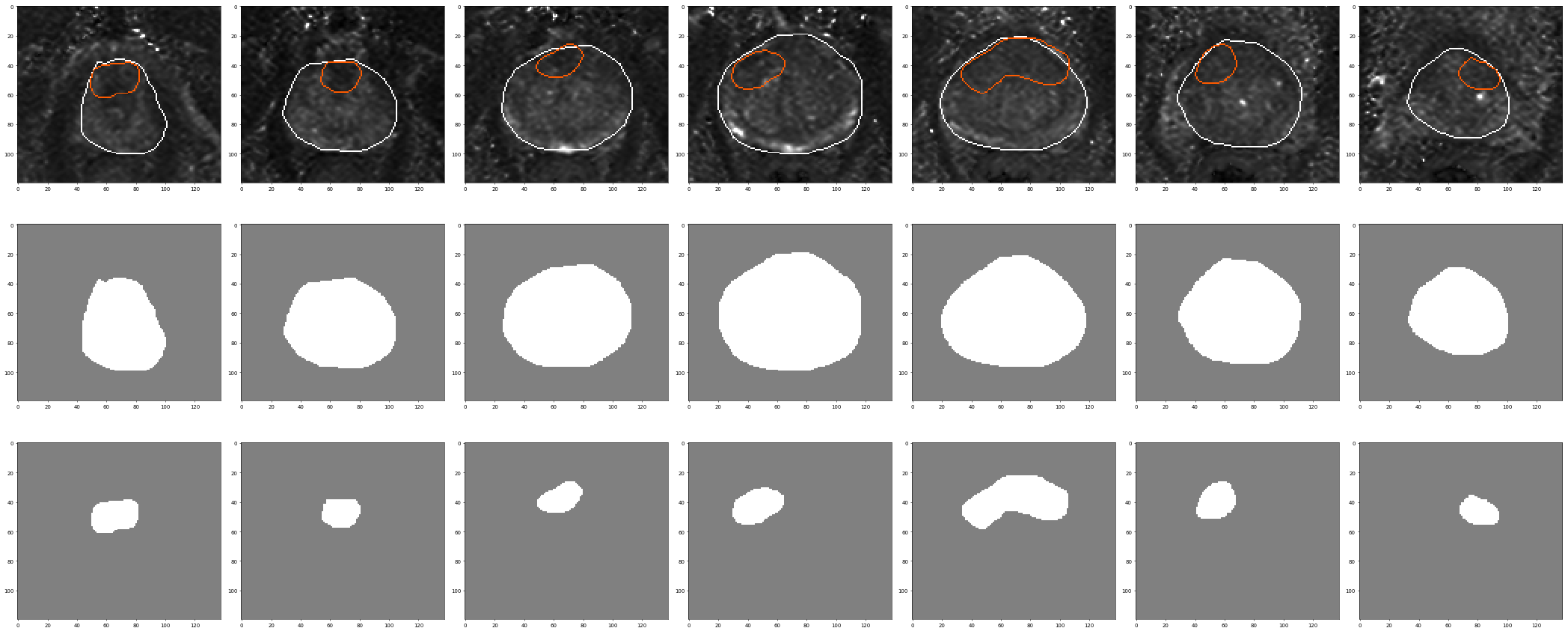

Supplement: S2 File — Files DWI-Mono-ADCm-xxx.png, T2-fitted-xxx.png, and T2w-std-xxx.png correspond to ADCm and T2 parametric maps, and T2-weighted images of each patient, respectively. On the first row of slices they show positions of regions of interest placed on the prostate cancer lesions (red, yellow) and around whole prostate (white). The prostate mask is on the second row, while the remaining rows are lesion masks. Files histology-xx.jpg contain the whole mount prostatectomy sections of each patient, with tumor outlines in green. Please note that identical MRI acquisition protocol has been used on all patients, including slice thickness. Here all prostate cancer masks are show with corresponding whole mount prostatectomy sections. (ZIP) [file pone.0217702.s002.zip › supporting_figures/T2-fitted-006.png]

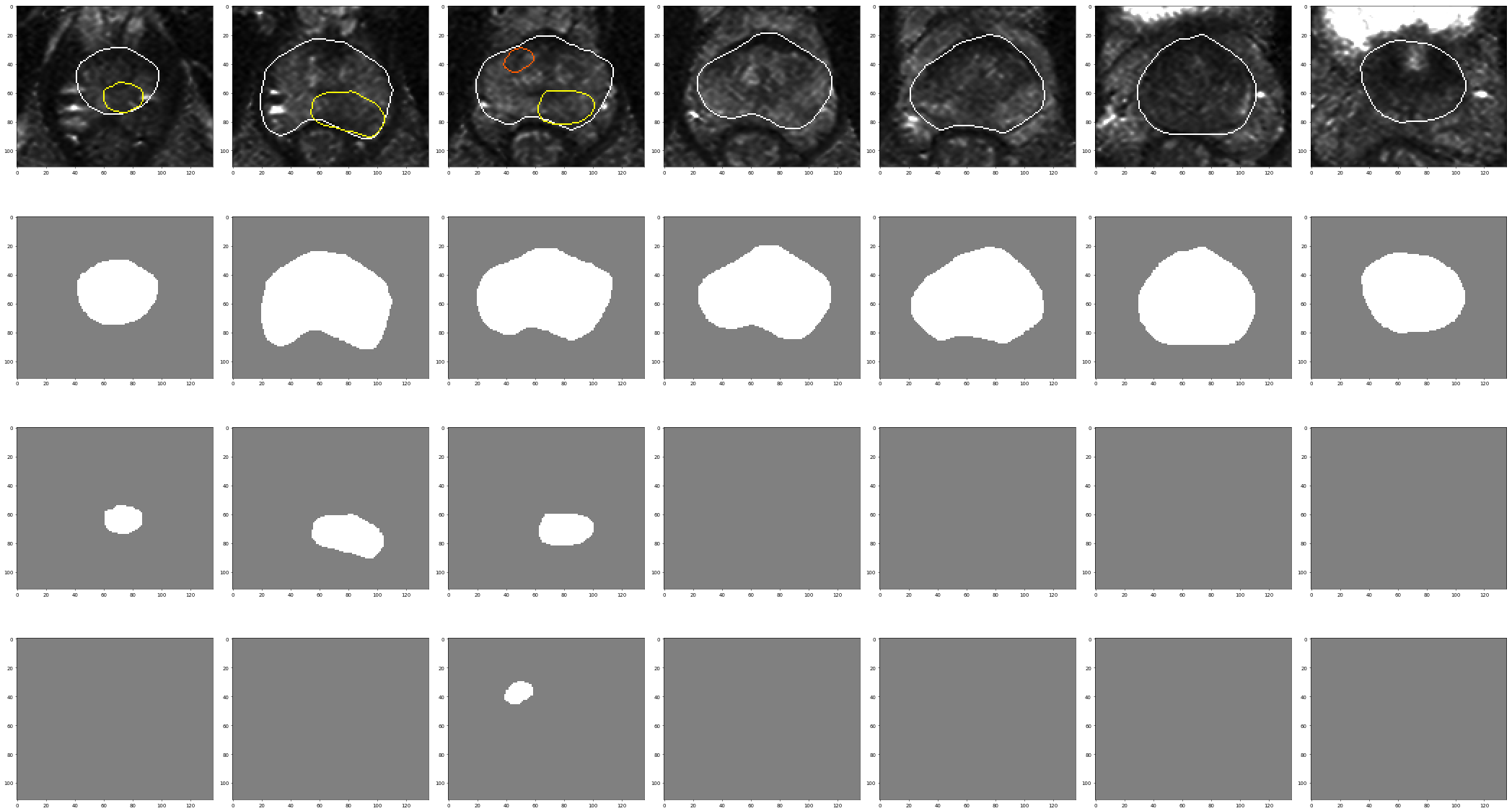

Supplement: S2 File — Files DWI-Mono-ADCm-xxx.png, T2-fitted-xxx.png, and T2w-std-xxx.png correspond to ADCm and T2 parametric maps, and T2-weighted images of each patient, respectively. On the first row of slices they show positions of regions of interest placed on the prostate cancer lesions (red, yellow) and around whole prostate (white). The prostate mask is on the second row, while the remaining rows are lesion masks. Files histology-xx.jpg contain the whole mount prostatectomy sections of each patient, with tumor outlines in green. Please note that identical MRI acquisition protocol has been used on all patients, including slice thickness. Here all prostate cancer masks are show with corresponding whole mount prostatectomy sections. (ZIP) [file pone.0217702.s002.zip › supporting_figures/T2-fitted-007.png]

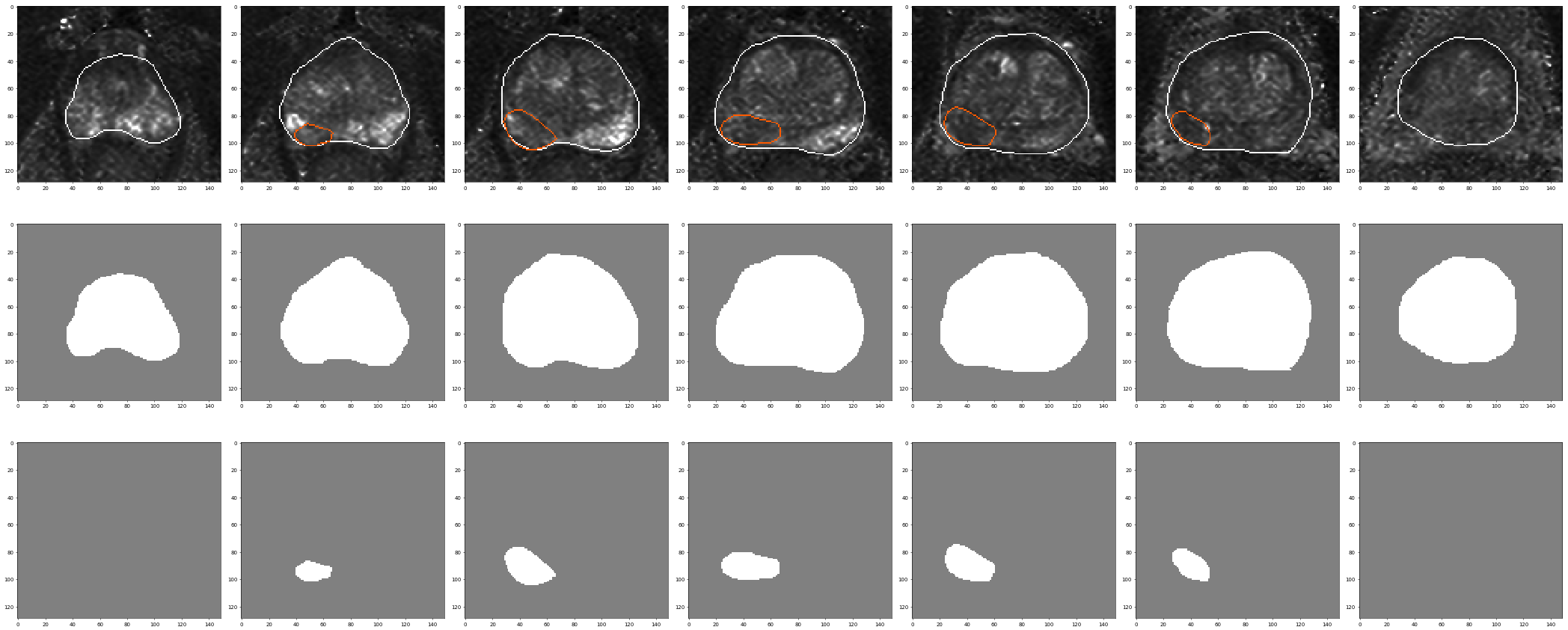

Supplement: S2 File — Files DWI-Mono-ADCm-xxx.png, T2-fitted-xxx.png, and T2w-std-xxx.png correspond to ADCm and T2 parametric maps, and T2-weighted images of each patient, respectively. On the first row of slices they show positions of regions of interest placed on the prostate cancer lesions (red, yellow) and around whole prostate (white). The prostate mask is on the second row, while the remaining rows are lesion masks. Files histology-xx.jpg contain the whole mount prostatectomy sections of each patient, with tumor outlines in green. Please note that identical MRI acquisition protocol has been used on all patients, including slice thickness. Here all prostate cancer masks are show with corresponding whole mount prostatectomy sections. (ZIP) [file pone.0217702.s002.zip › supporting_figures/T2-fitted-008.png]

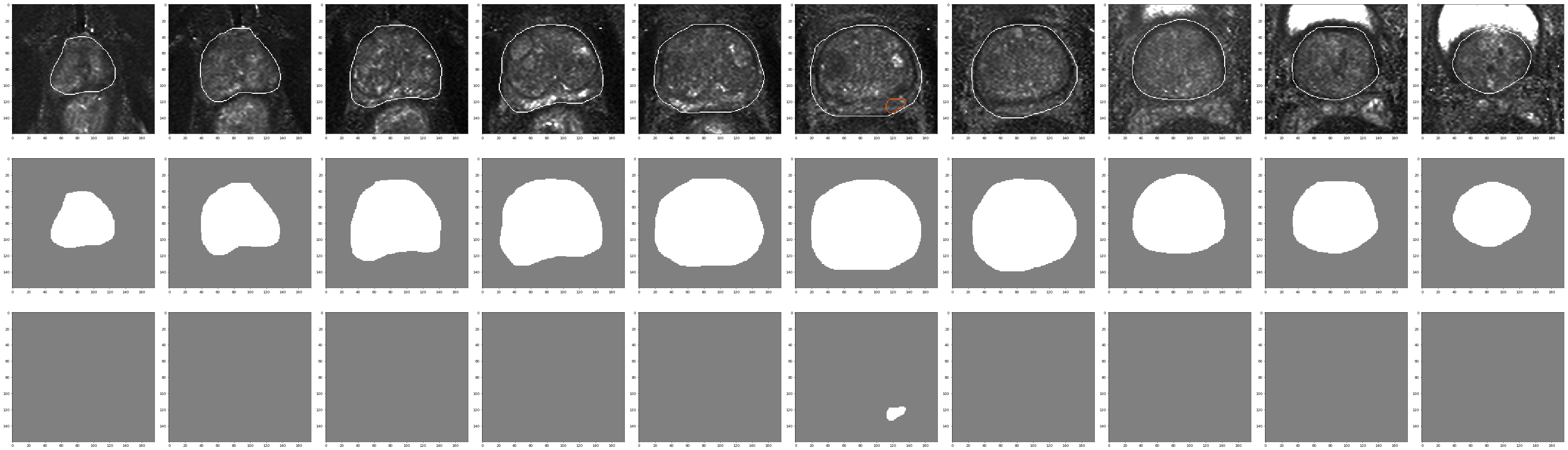

Supplement: S2 File — Files DWI-Mono-ADCm-xxx.png, T2-fitted-xxx.png, and T2w-std-xxx.png correspond to ADCm and T2 parametric maps, and T2-weighted images of each patient, respectively. On the first row of slices they show positions of regions of interest placed on the prostate cancer lesions (red, yellow) and around whole prostate (white). The prostate mask is on the second row, while the remaining rows are lesion masks. Files histology-xx.jpg contain the whole mount prostatectomy sections of each patient, with tumor outlines in green. Please note that identical MRI acquisition protocol has been used on all patients, including slice thickness. Here all prostate cancer masks are show with corresponding whole mount prostatectomy sections. (ZIP) [file pone.0217702.s002.zip › supporting_figures/T2-fitted-009.png]

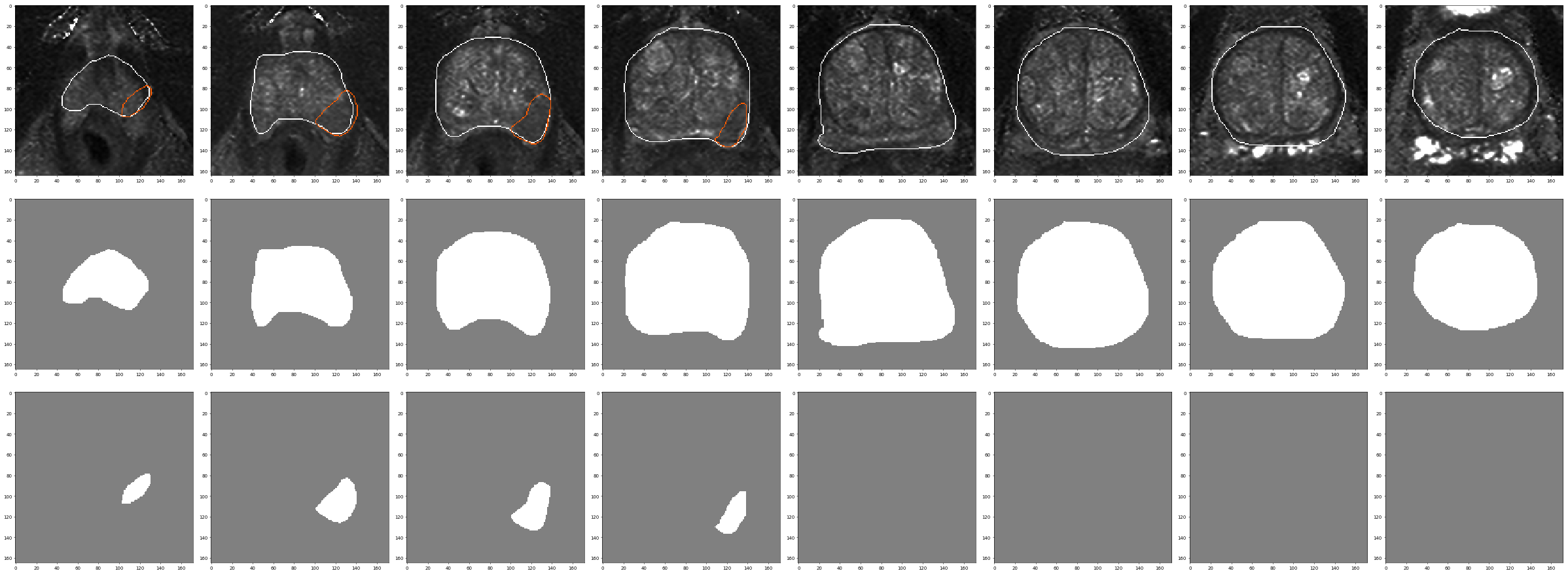

Supplement: S2 File — Files DWI-Mono-ADCm-xxx.png, T2-fitted-xxx.png, and T2w-std-xxx.png correspond to ADCm and T2 parametric maps, and T2-weighted images of each patient, respectively. On the first row of slices they show positions of regions of interest placed on the prostate cancer lesions (red, yellow) and around whole prostate (white). The prostate mask is on the second row, while the remaining rows are lesion masks. Files histology-xx.jpg contain the whole mount prostatectomy sections of each patient, with tumor outlines in green. Please note that identical MRI acquisition protocol has been used on all patients, including slice thickness. Here all prostate cancer masks are show with corresponding whole mount prostatectomy sections. (ZIP) [file pone.0217702.s002.zip › supporting_figures/T2-fitted-010.png]

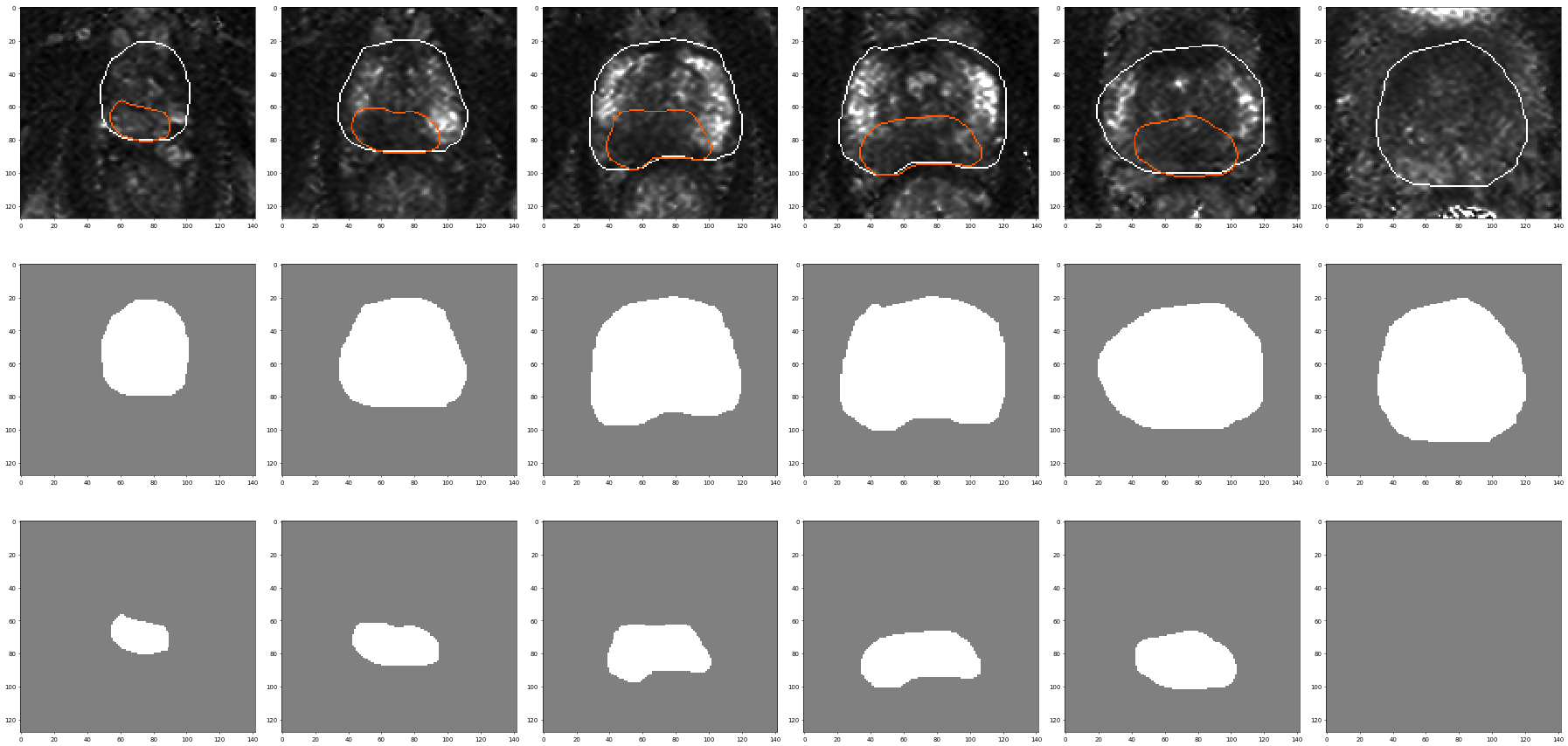

Supplement: S2 File — Files DWI-Mono-ADCm-xxx.png, T2-fitted-xxx.png, and T2w-std-xxx.png correspond to ADCm and T2 parametric maps, and T2-weighted images of each patient, respectively. On the first row of slices they show positions of regions of interest placed on the prostate cancer lesions (red, yellow) and around whole prostate (white). The prostate mask is on the second row, while the remaining rows are lesion masks. Files histology-xx.jpg contain the whole mount prostatectomy sections of each patient, with tumor outlines in green. Please note that identical MRI acquisition protocol has been used on all patients, including slice thickness. Here all prostate cancer masks are show with corresponding whole mount prostatectomy sections. (ZIP) [file pone.0217702.s002.zip › supporting_figures/T2-fitted-011.png]

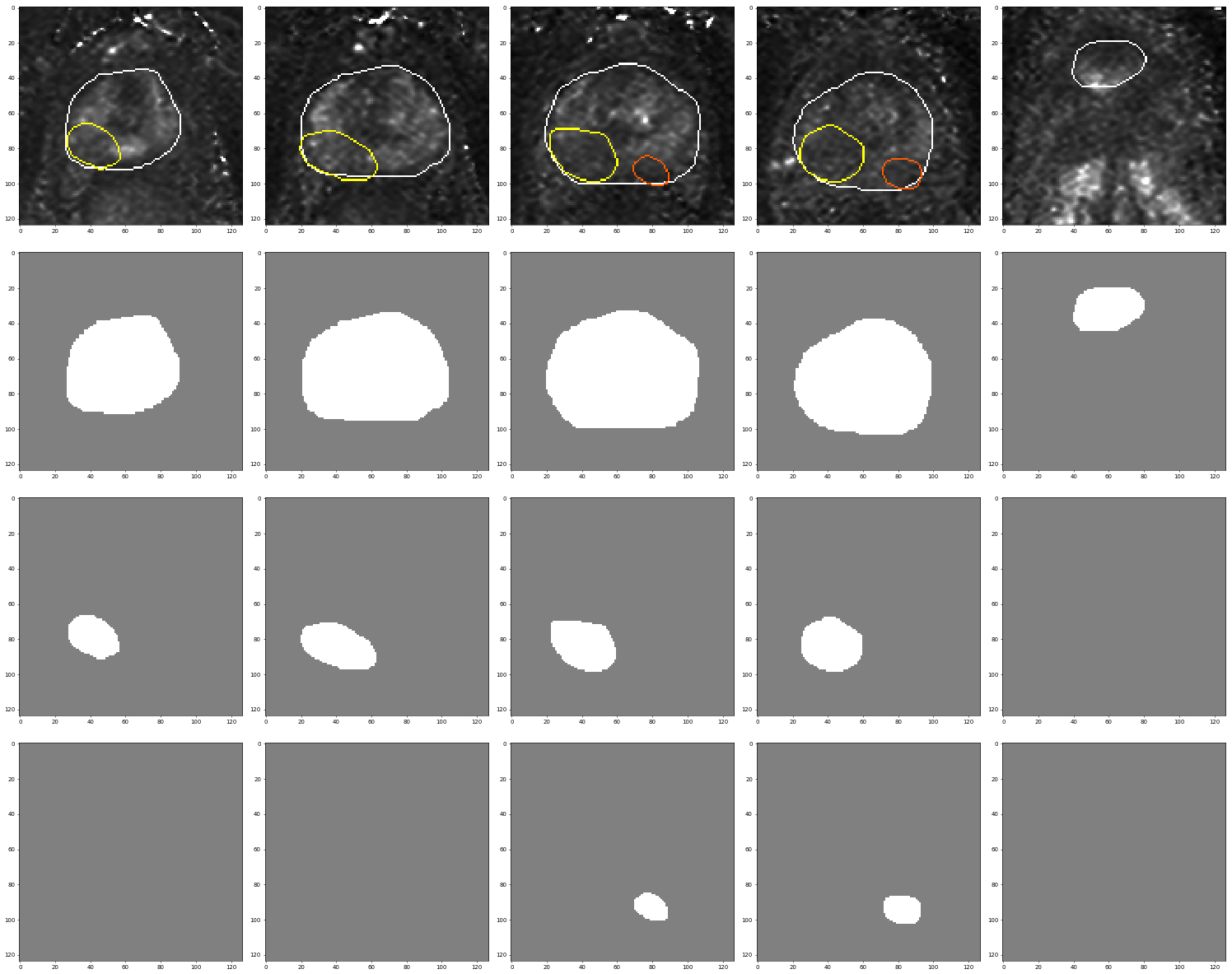

Supplement: S2 File — Files DWI-Mono-ADCm-xxx.png, T2-fitted-xxx.png, and T2w-std-xxx.png correspond to ADCm and T2 parametric maps, and T2-weighted images of each patient, respectively. On the first row of slices they show positions of regions of interest placed on the prostate cancer lesions (red, yellow) and around whole prostate (white). The prostate mask is on the second row, while the remaining rows are lesion masks. Files histology-xx.jpg contain the whole mount prostatectomy sections of each patient, with tumor outlines in green. Please note that identical MRI acquisition protocol has been used on all patients, including slice thickness. Here all prostate cancer masks are show with corresponding whole mount prostatectomy sections. (ZIP) [file pone.0217702.s002.zip › supporting_figures/T2-fitted-012.png]

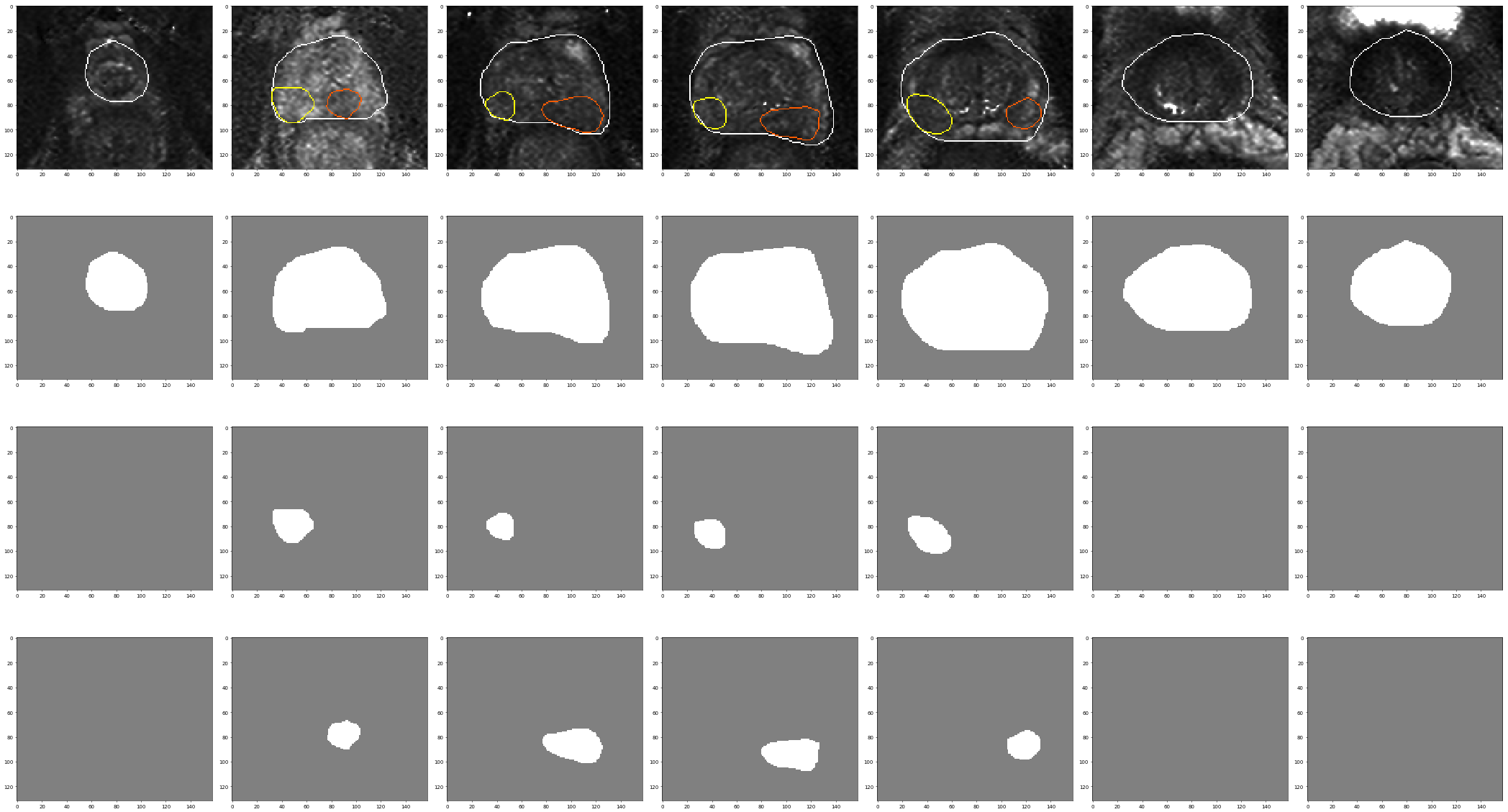

Supplement: S2 File — Files DWI-Mono-ADCm-xxx.png, T2-fitted-xxx.png, and T2w-std-xxx.png correspond to ADCm and T2 parametric maps, and T2-weighted images of each patient, respectively. On the first row of slices they show positions of regions of interest placed on the prostate cancer lesions (red, yellow) and around whole prostate (white). The prostate mask is on the second row, while the remaining rows are lesion masks. Files histology-xx.jpg contain the whole mount prostatectomy sections of each patient, with tumor outlines in green. Please note that identical MRI acquisition protocol has been used on all patients, including slice thickness. Here all prostate cancer masks are show with corresponding whole mount prostatectomy sections. (ZIP) [file pone.0217702.s002.zip › supporting_figures/T2-fitted-013.png]

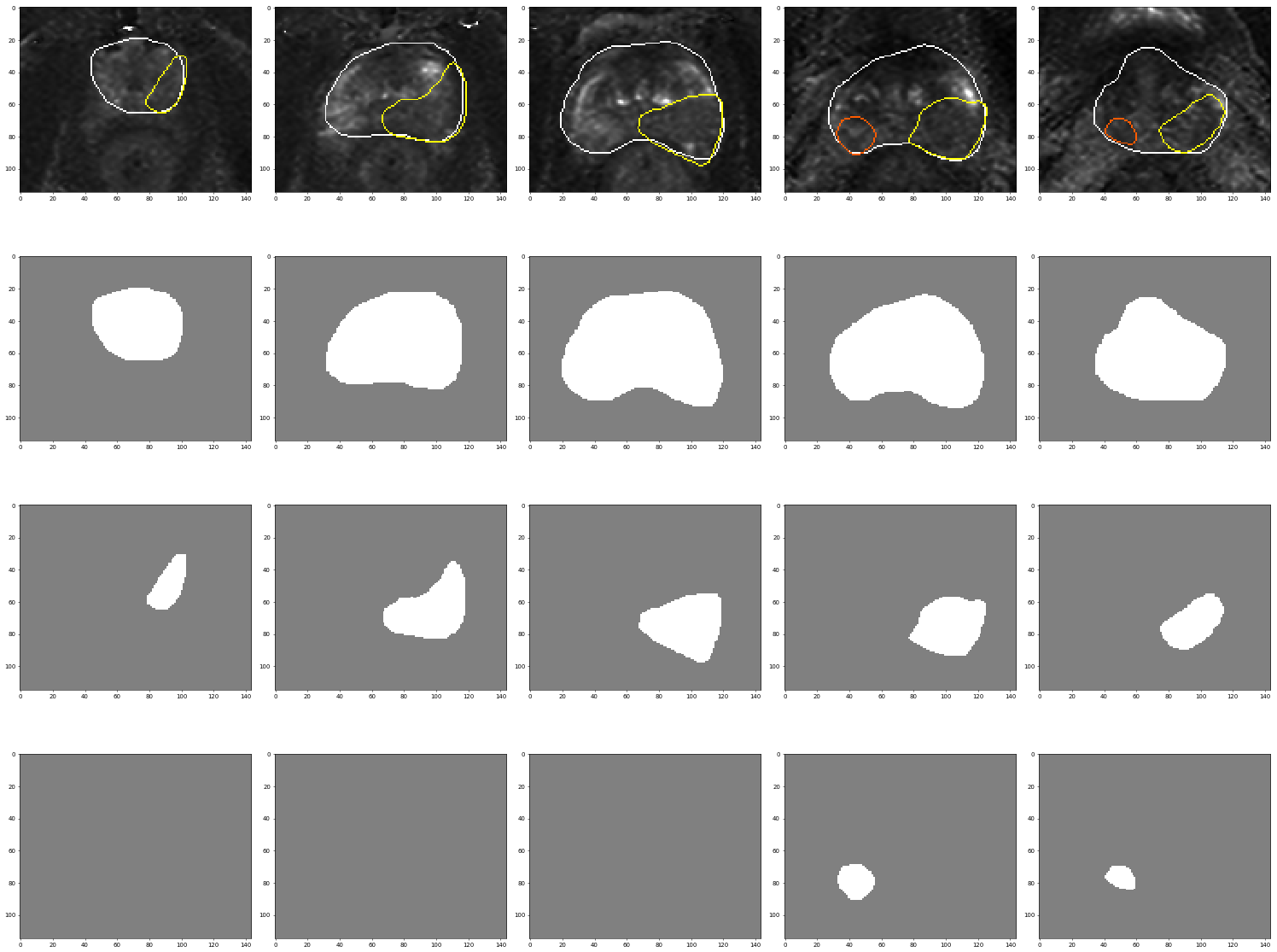

Supplement: S2 File — Files DWI-Mono-ADCm-xxx.png, T2-fitted-xxx.png, and T2w-std-xxx.png correspond to ADCm and T2 parametric maps, and T2-weighted images of each patient, respectively. On the first row of slices they show positions of regions of interest placed on the prostate cancer lesions (red, yellow) and around whole prostate (white). The prostate mask is on the second row, while the remaining rows are lesion masks. Files histology-xx.jpg contain the whole mount prostatectomy sections of each patient, with tumor outlines in green. Please note that identical MRI acquisition protocol has been used on all patients, including slice thickness. Here all prostate cancer masks are show with corresponding whole mount prostatectomy sections. (ZIP) [file pone.0217702.s002.zip › supporting_figures/T2-fitted-014.png]

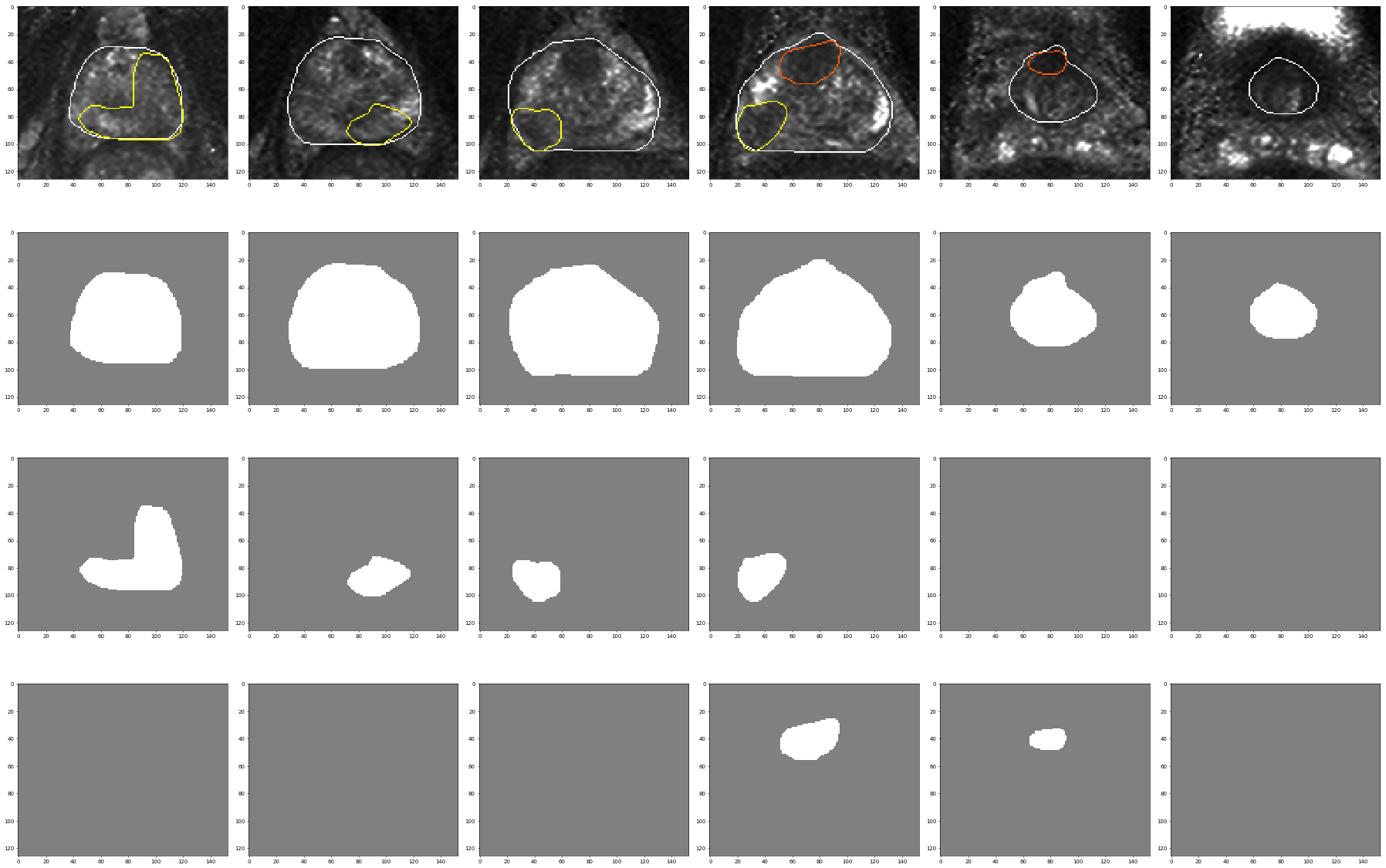

Supplement: S2 File — Files DWI-Mono-ADCm-xxx.png, T2-fitted-xxx.png, and T2w-std-xxx.png correspond to ADCm and T2 parametric maps, and T2-weighted images of each patient, respectively. On the first row of slices they show positions of regions of interest placed on the prostate cancer lesions (red, yellow) and around whole prostate (white). The prostate mask is on the second row, while the remaining rows are lesion masks. Files histology-xx.jpg contain the whole mount prostatectomy sections of each patient, with tumor outlines in green. Please note that identical MRI acquisition protocol has been used on all patients, including slice thickness. Here all prostate cancer masks are show with corresponding whole mount prostatectomy sections. (ZIP) [file pone.0217702.s002.zip › supporting_figures/T2-fitted-015.png]

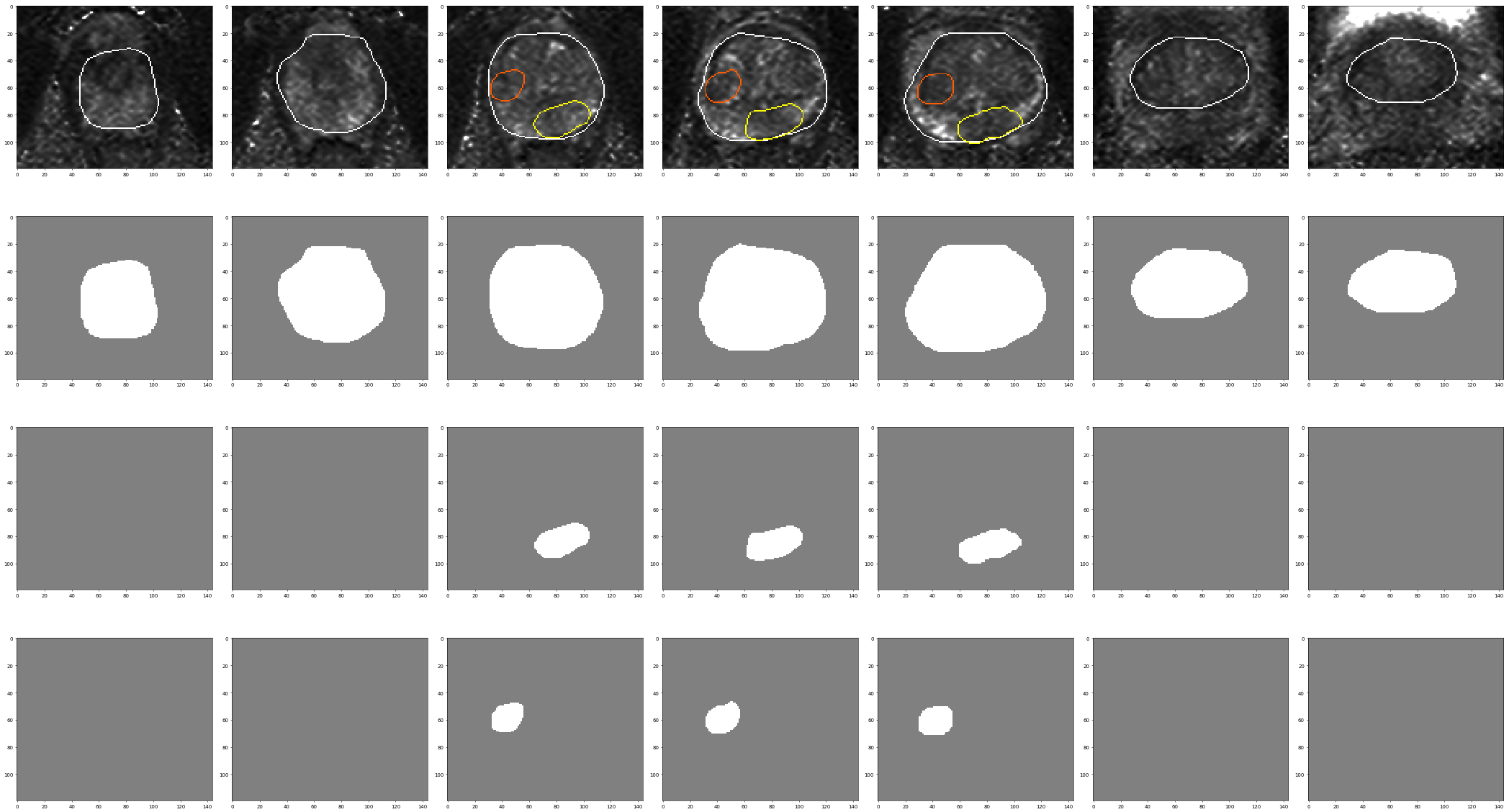

Supplement: S2 File — Files DWI-Mono-ADCm-xxx.png, T2-fitted-xxx.png, and T2w-std-xxx.png correspond to ADCm and T2 parametric maps, and T2-weighted images of each patient, respectively. On the first row of slices they show positions of regions of interest placed on the prostate cancer lesions (red, yellow) and around whole prostate (white). The prostate mask is on the second row, while the remaining rows are lesion masks. Files histology-xx.jpg contain the whole mount prostatectomy sections of each patient, with tumor outlines in green. Please note that identical MRI acquisition protocol has been used on all patients, including slice thickness. Here all prostate cancer masks are show with corresponding whole mount prostatectomy sections. (ZIP) [file pone.0217702.s002.zip › supporting_figures/T2-fitted-016.png]

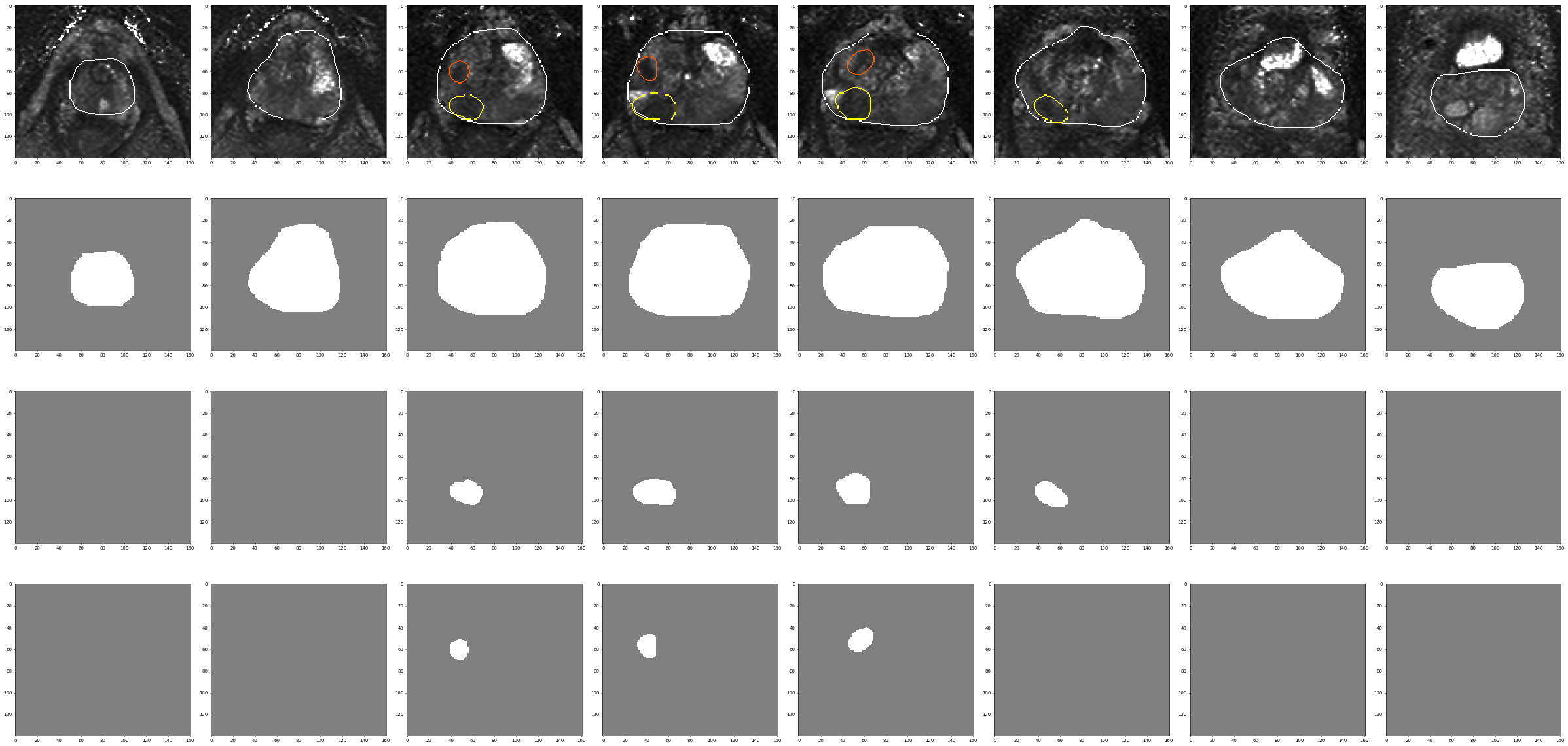

Supplement: S2 File — Files DWI-Mono-ADCm-xxx.png, T2-fitted-xxx.png, and T2w-std-xxx.png correspond to ADCm and T2 parametric maps, and T2-weighted images of each patient, respectively. On the first row of slices they show positions of regions of interest placed on the prostate cancer lesions (red, yellow) and around whole prostate (white). The prostate mask is on the second row, while the remaining rows are lesion masks. Files histology-xx.jpg contain the whole mount prostatectomy sections of each patient, with tumor outlines in green. Please note that identical MRI acquisition protocol has been used on all patients, including slice thickness. Here all prostate cancer masks are show with corresponding whole mount prostatectomy sections. (ZIP) [file pone.0217702.s002.zip › supporting_figures/T2-fitted-017.png]

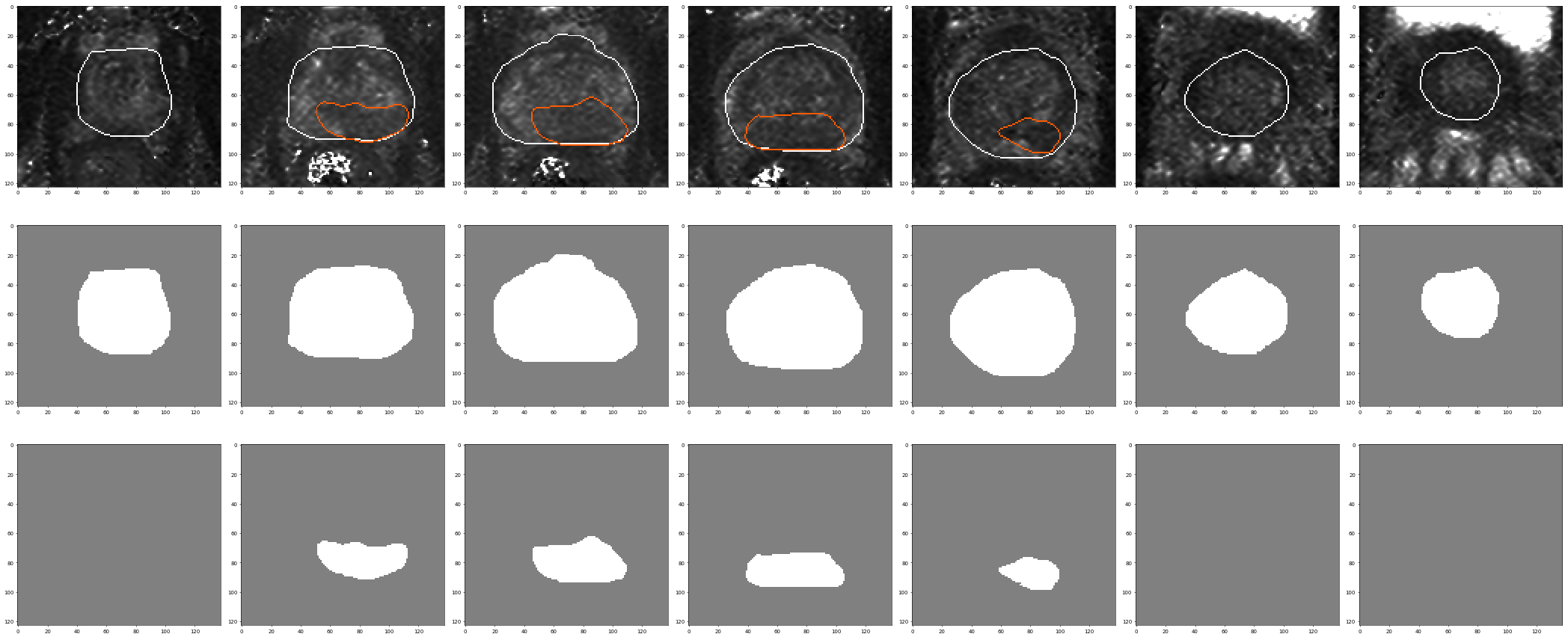

Supplement: S2 File — Files DWI-Mono-ADCm-xxx.png, T2-fitted-xxx.png, and T2w-std-xxx.png correspond to ADCm and T2 parametric maps, and T2-weighted images of each patient, respectively. On the first row of slices they show positions of regions of interest placed on the prostate cancer lesions (red, yellow) and around whole prostate (white). The prostate mask is on the second row, while the remaining rows are lesion masks. Files histology-xx.jpg contain the whole mount prostatectomy sections of each patient, with tumor outlines in green. Please note that identical MRI acquisition protocol has been used on all patients, including slice thickness. Here all prostate cancer masks are show with corresponding whole mount prostatectomy sections. (ZIP) [file pone.0217702.s002.zip › supporting_figures/T2-fitted-018.png]

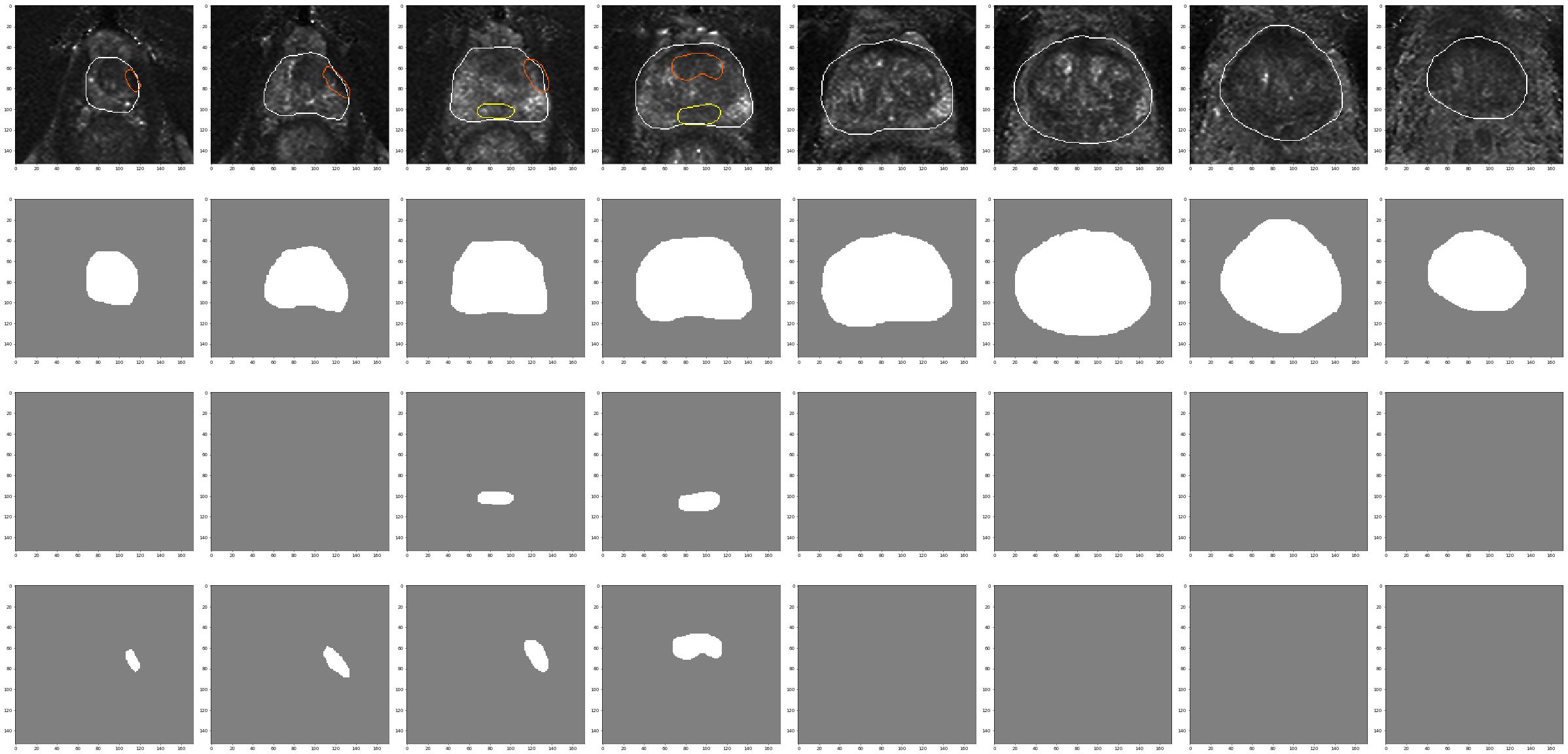

Supplement: S2 File — Files DWI-Mono-ADCm-xxx.png, T2-fitted-xxx.png, and T2w-std-xxx.png correspond to ADCm and T2 parametric maps, and T2-weighted images of each patient, respectively. On the first row of slices they show positions of regions of interest placed on the prostate cancer lesions (red, yellow) and around whole prostate (white). The prostate mask is on the second row, while the remaining rows are lesion masks. Files histology-xx.jpg contain the whole mount prostatectomy sections of each patient, with tumor outlines in green. Please note that identical MRI acquisition protocol has been used on all patients, including slice thickness. Here all prostate cancer masks are show with corresponding whole mount prostatectomy sections. (ZIP) [file pone.0217702.s002.zip › supporting_figures/T2-fitted-019.png]

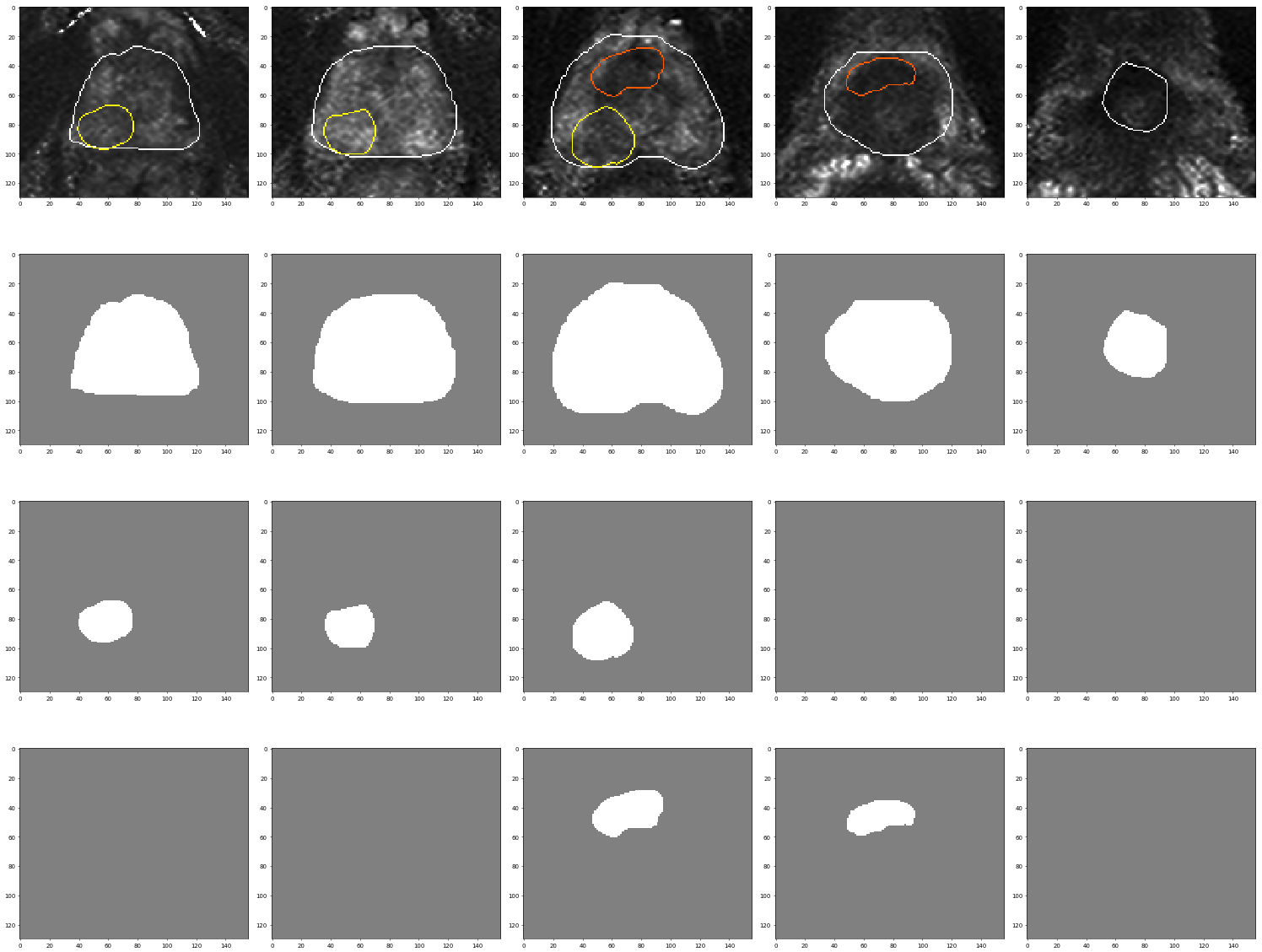

Supplement: S2 File — Files DWI-Mono-ADCm-xxx.png, T2-fitted-xxx.png, and T2w-std-xxx.png correspond to ADCm and T2 parametric maps, and T2-weighted images of each patient, respectively. On the first row of slices they show positions of regions of interest placed on the prostate cancer lesions (red, yellow) and around whole prostate (white). The prostate mask is on the second row, while the remaining rows are lesion masks. Files histology-xx.jpg contain the whole mount prostatectomy sections of each patient, with tumor outlines in green. Please note that identical MRI acquisition protocol has been used on all patients, including slice thickness. Here all prostate cancer masks are show with corresponding whole mount prostatectomy sections. (ZIP) [file pone.0217702.s002.zip › supporting_figures/T2-fitted-020.png]

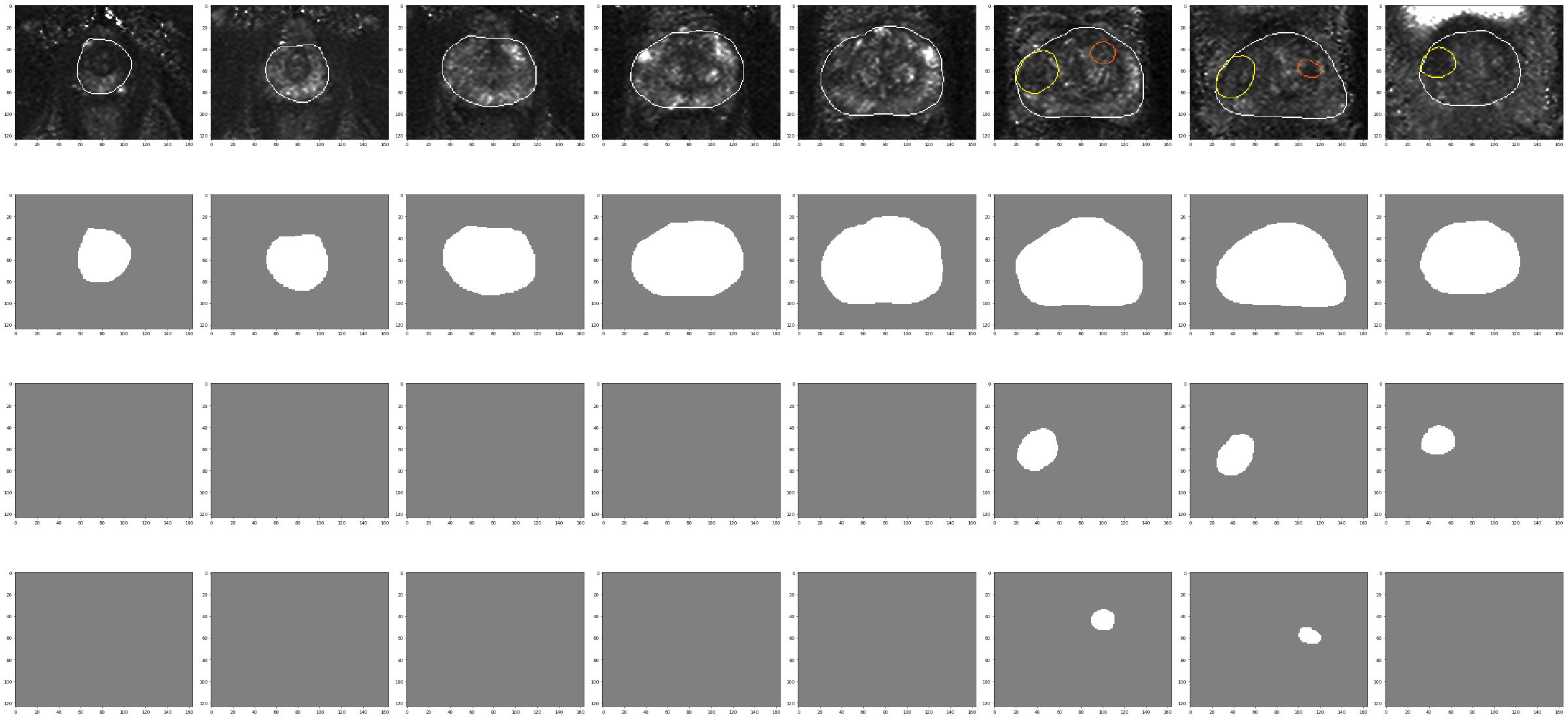

Supplement: S2 File — Files DWI-Mono-ADCm-xxx.png, T2-fitted-xxx.png, and T2w-std-xxx.png correspond to ADCm and T2 parametric maps, and T2-weighted images of each patient, respectively. On the first row of slices they show positions of regions of interest placed on the prostate cancer lesions (red, yellow) and around whole prostate (white). The prostate mask is on the second row, while the remaining rows are lesion masks. Files histology-xx.jpg contain the whole mount prostatectomy sections of each patient, with tumor outlines in green. Please note that identical MRI acquisition protocol has been used on all patients, including slice thickness. Here all prostate cancer masks are show with corresponding whole mount prostatectomy sections. (ZIP) [file pone.0217702.s002.zip › supporting_figures/T2-fitted-021.png]

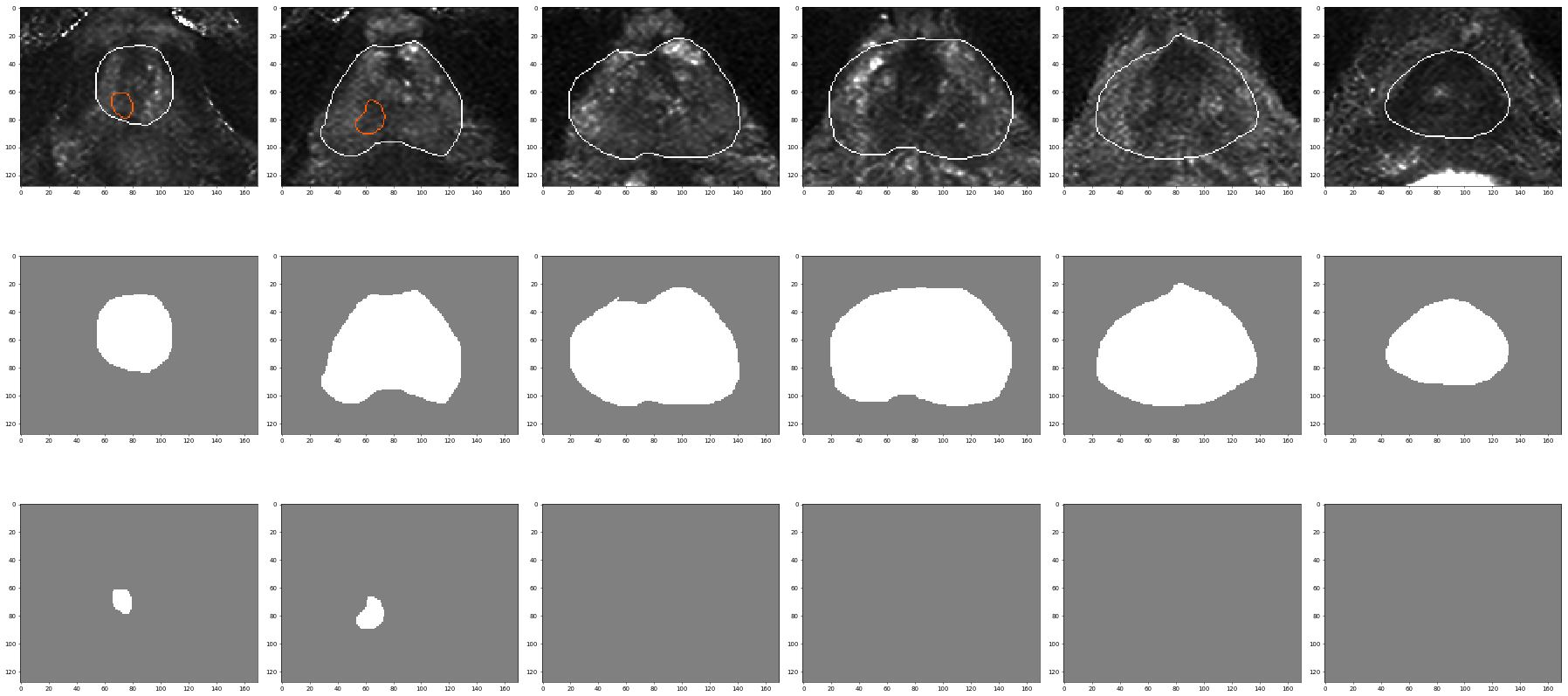

Supplement: S2 File — Files DWI-Mono-ADCm-xxx.png, T2-fitted-xxx.png, and T2w-std-xxx.png correspond to ADCm and T2 parametric maps, and T2-weighted images of each patient, respectively. On the first row of slices they show positions of regions of interest placed on the prostate cancer lesions (red, yellow) and around whole prostate (white). The prostate mask is on the second row, while the remaining rows are lesion masks. Files histology-xx.jpg contain the whole mount prostatectomy sections of each patient, with tumor outlines in green. Please note that identical MRI acquisition protocol has been used on all patients, including slice thickness. Here all prostate cancer masks are show with corresponding whole mount prostatectomy sections. (ZIP) [file pone.0217702.s002.zip › supporting_figures/T2-fitted-022.png]

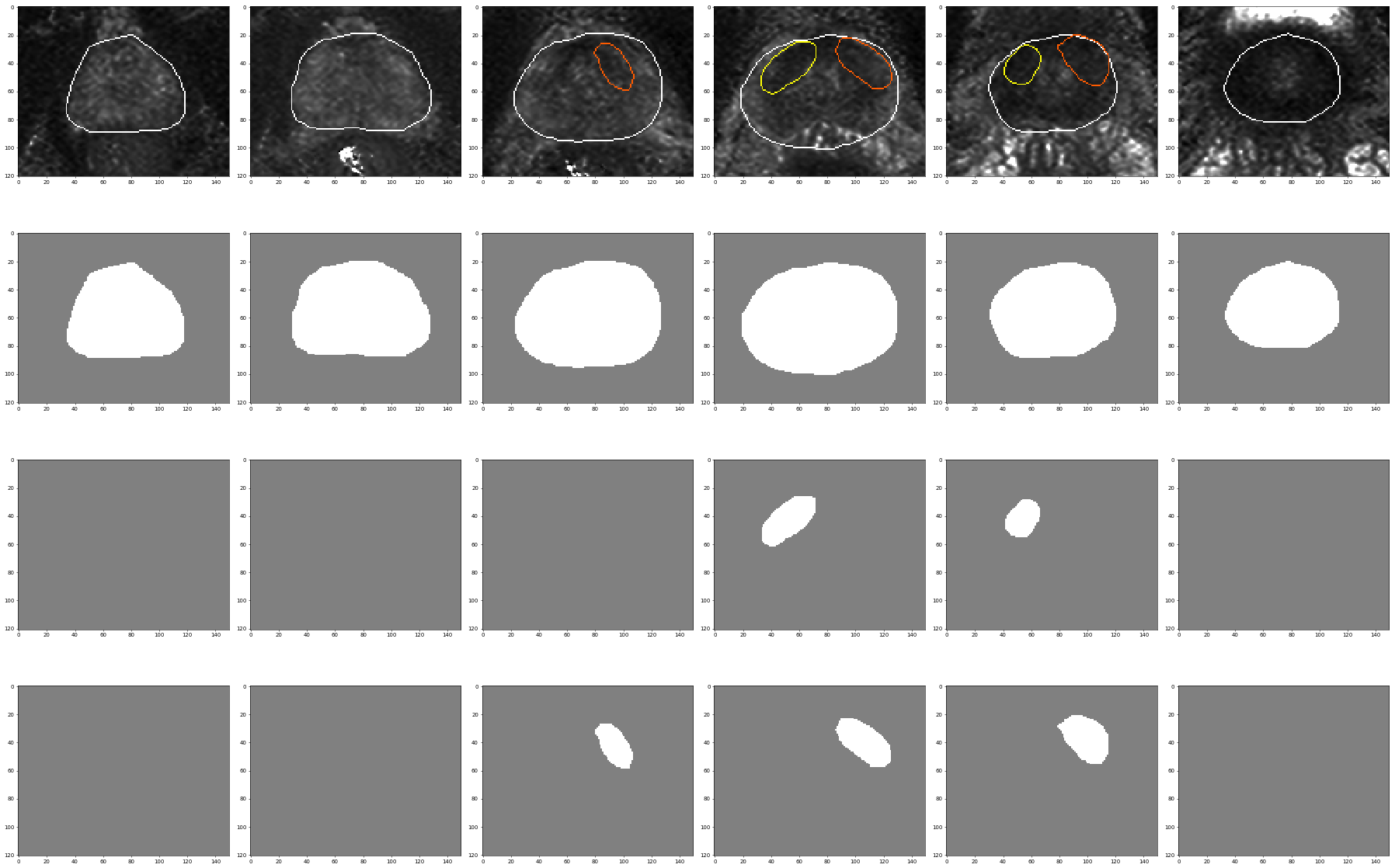

Supplement: S2 File — Files DWI-Mono-ADCm-xxx.png, T2-fitted-xxx.png, and T2w-std-xxx.png correspond to ADCm and T2 parametric maps, and T2-weighted images of each patient, respectively. On the first row of slices they show positions of regions of interest placed on the prostate cancer lesions (red, yellow) and around whole prostate (white). The prostate mask is on the second row, while the remaining rows are lesion masks. Files histology-xx.jpg contain the whole mount prostatectomy sections of each patient, with tumor outlines in green. Please note that identical MRI acquisition protocol has been used on all patients, including slice thickness. Here all prostate cancer masks are show with corresponding whole mount prostatectomy sections. (ZIP) [file pone.0217702.s002.zip › supporting_figures/T2-fitted-023.png]

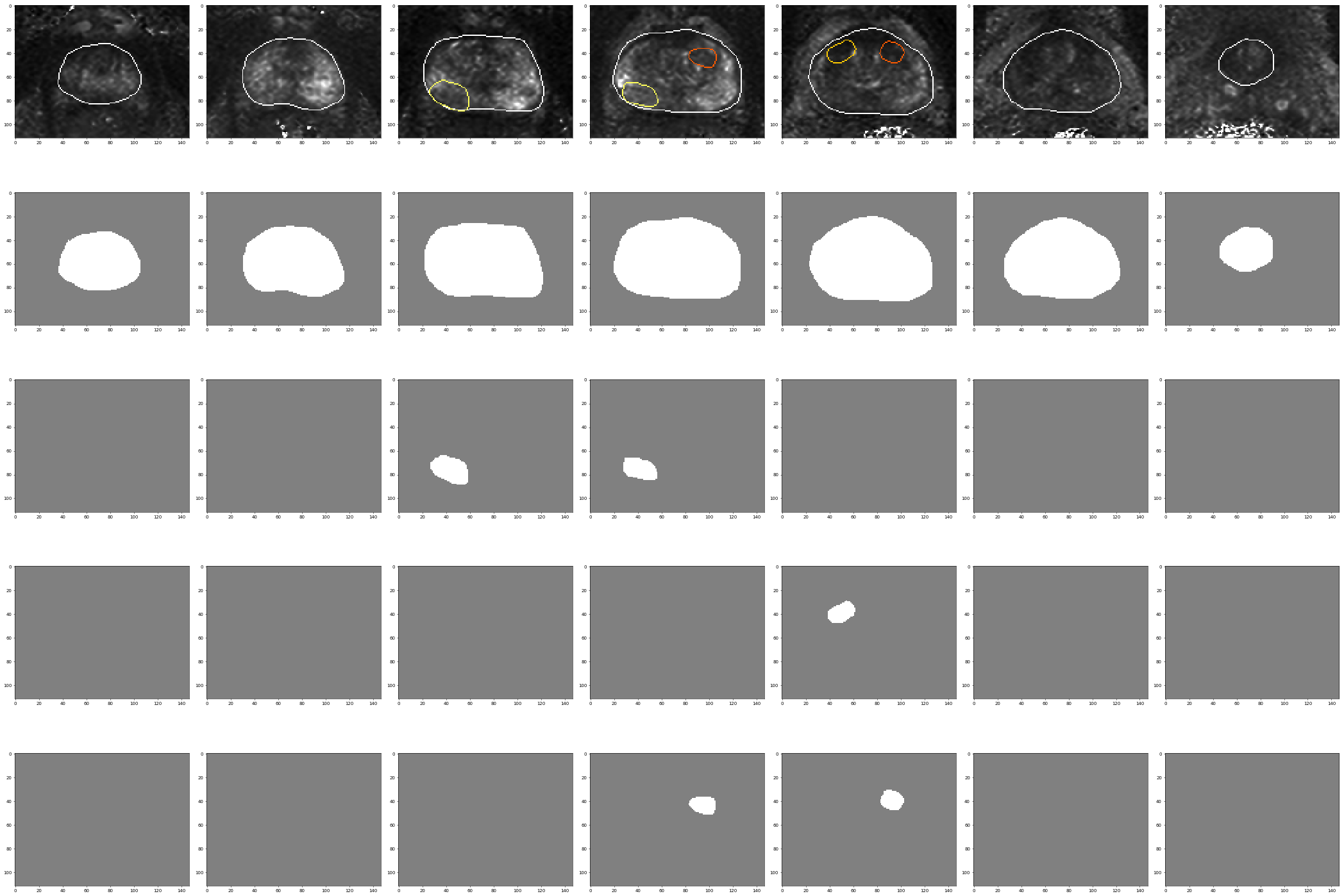

Supplement: S2 File — Files DWI-Mono-ADCm-xxx.png, T2-fitted-xxx.png, and T2w-std-xxx.png correspond to ADCm and T2 parametric maps, and T2-weighted images of each patient, respectively. On the first row of slices they show positions of regions of interest placed on the prostate cancer lesions (red, yellow) and around whole prostate (white). The prostate mask is on the second row, while the remaining rows are lesion masks. Files histology-xx.jpg contain the whole mount prostatectomy sections of each patient, with tumor outlines in green. Please note that identical MRI acquisition protocol has been used on all patients, including slice thickness. Here all prostate cancer masks are show with corresponding whole mount prostatectomy sections. (ZIP) [file pone.0217702.s002.zip › supporting_figures/T2-fitted-024.png]

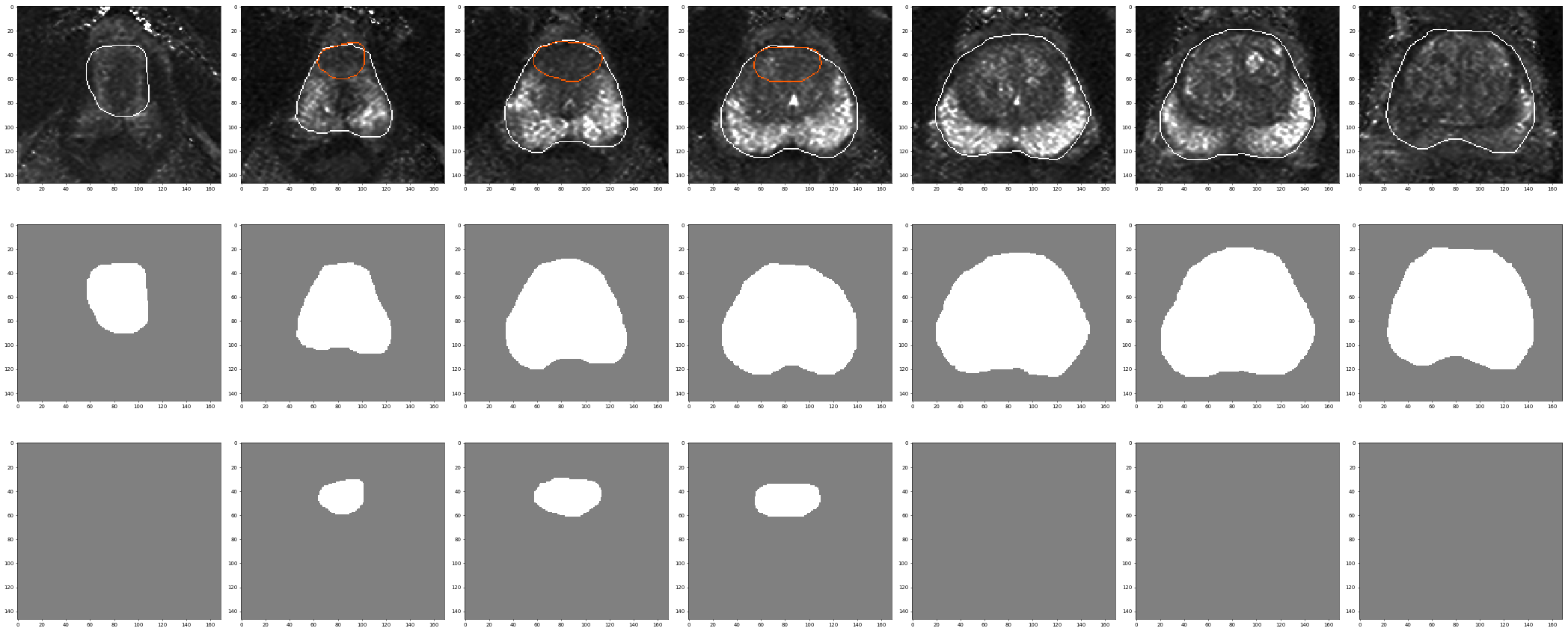

Supplement: S2 File — Files DWI-Mono-ADCm-xxx.png, T2-fitted-xxx.png, and T2w-std-xxx.png correspond to ADCm and T2 parametric maps, and T2-weighted images of each patient, respectively. On the first row of slices they show positions of regions of interest placed on the prostate cancer lesions (red, yellow) and around whole prostate (white). The prostate mask is on the second row, while the remaining rows are lesion masks. Files histology-xx.jpg contain the whole mount prostatectomy sections of each patient, with tumor outlines in green. Please note that identical MRI acquisition protocol has been used on all patients, including slice thickness. Here all prostate cancer masks are show with corresponding whole mount prostatectomy sections. (ZIP) [file pone.0217702.s002.zip › supporting_figures/T2-fitted-025.png]

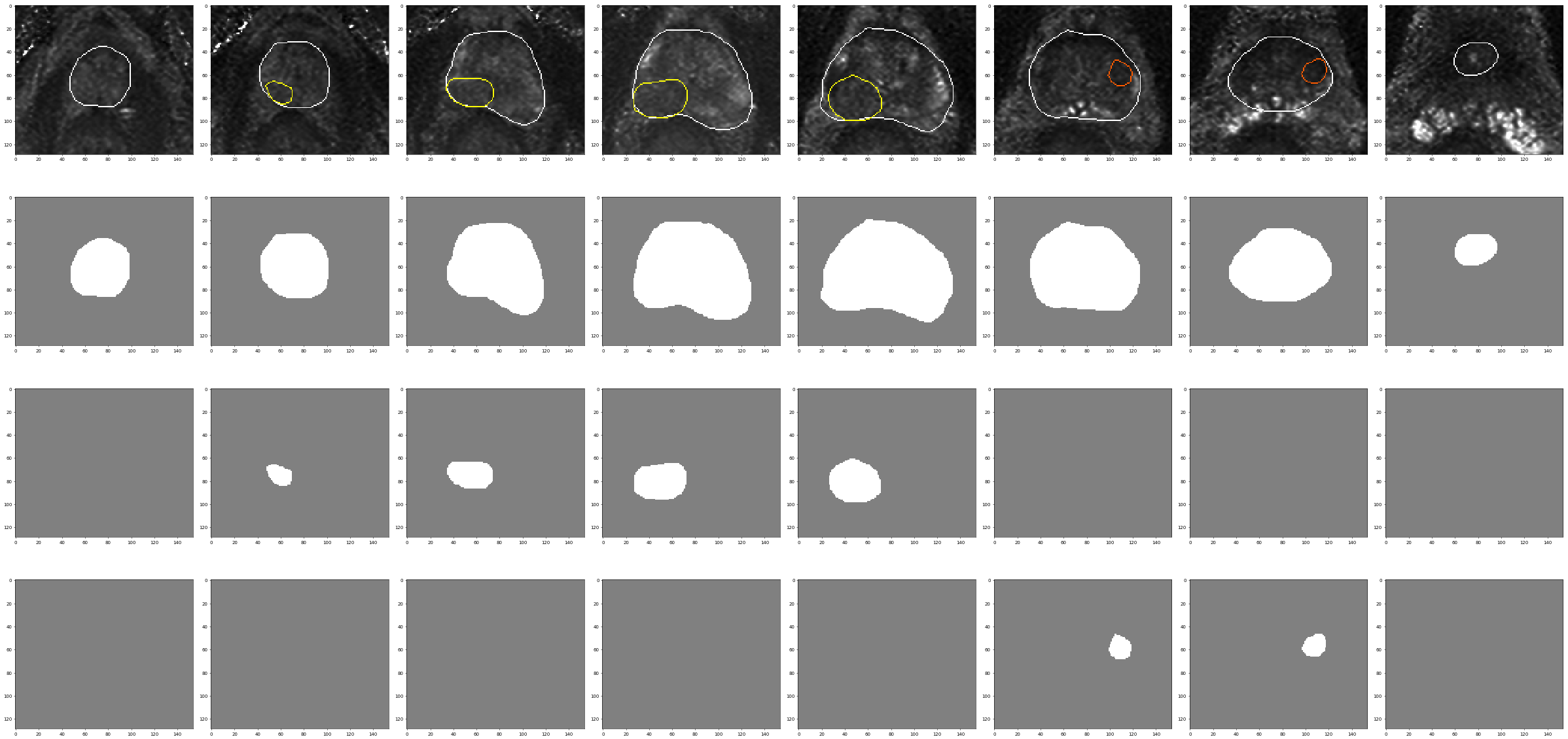

Supplement: S2 File — Files DWI-Mono-ADCm-xxx.png, T2-fitted-xxx.png, and T2w-std-xxx.png correspond to ADCm and T2 parametric maps, and T2-weighted images of each patient, respectively. On the first row of slices they show positions of regions of interest placed on the prostate cancer lesions (red, yellow) and around whole prostate (white). The prostate mask is on the second row, while the remaining rows are lesion masks. Files histology-xx.jpg contain the whole mount prostatectomy sections of each patient, with tumor outlines in green. Please note that identical MRI acquisition protocol has been used on all patients, including slice thickness. Here all prostate cancer masks are show with corresponding whole mount prostatectomy sections. (ZIP) [file pone.0217702.s002.zip › supporting_figures/T2-fitted-026.png]

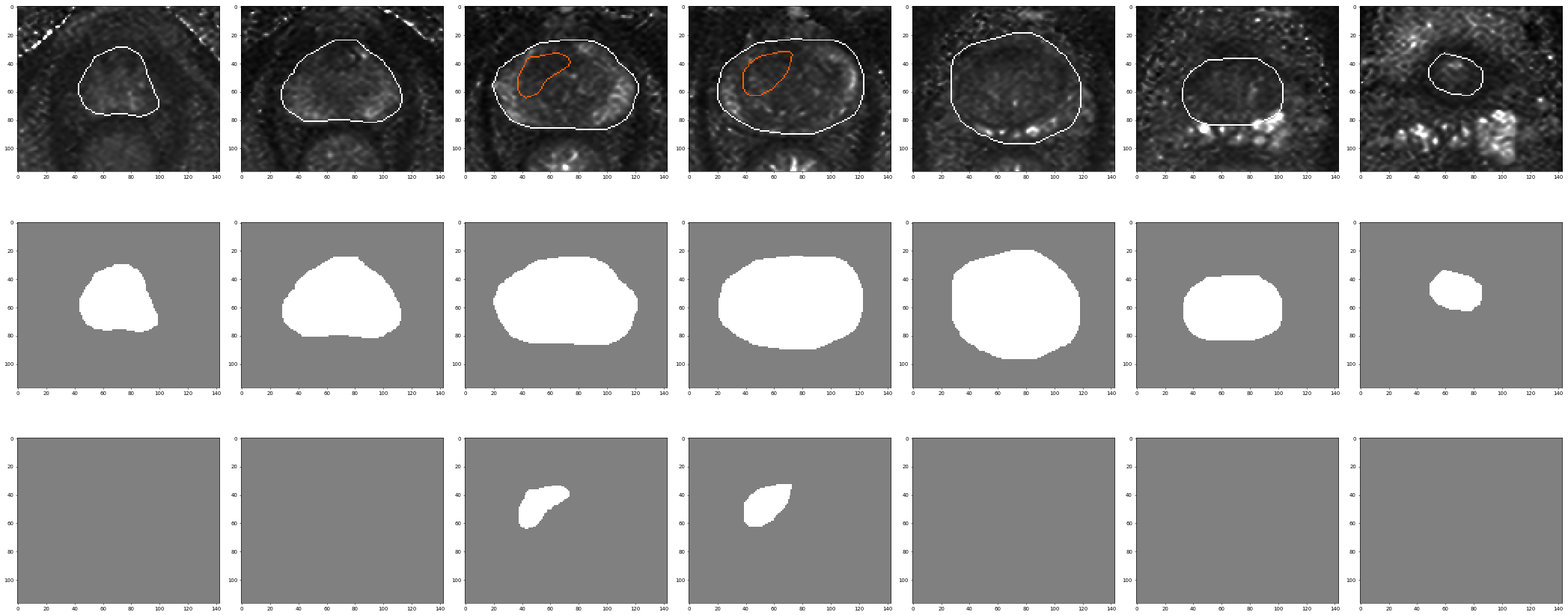

Supplement: S2 File — Files DWI-Mono-ADCm-xxx.png, T2-fitted-xxx.png, and T2w-std-xxx.png correspond to ADCm and T2 parametric maps, and T2-weighted images of each patient, respectively. On the first row of slices they show positions of regions of interest placed on the prostate cancer lesions (red, yellow) and around whole prostate (white). The prostate mask is on the second row, while the remaining rows are lesion masks. Files histology-xx.jpg contain the whole mount prostatectomy sections of each patient, with tumor outlines in green. Please note that identical MRI acquisition protocol has been used on all patients, including slice thickness. Here all prostate cancer masks are show with corresponding whole mount prostatectomy sections. (ZIP) [file pone.0217702.s002.zip › supporting_figures/T2-fitted-027.png]

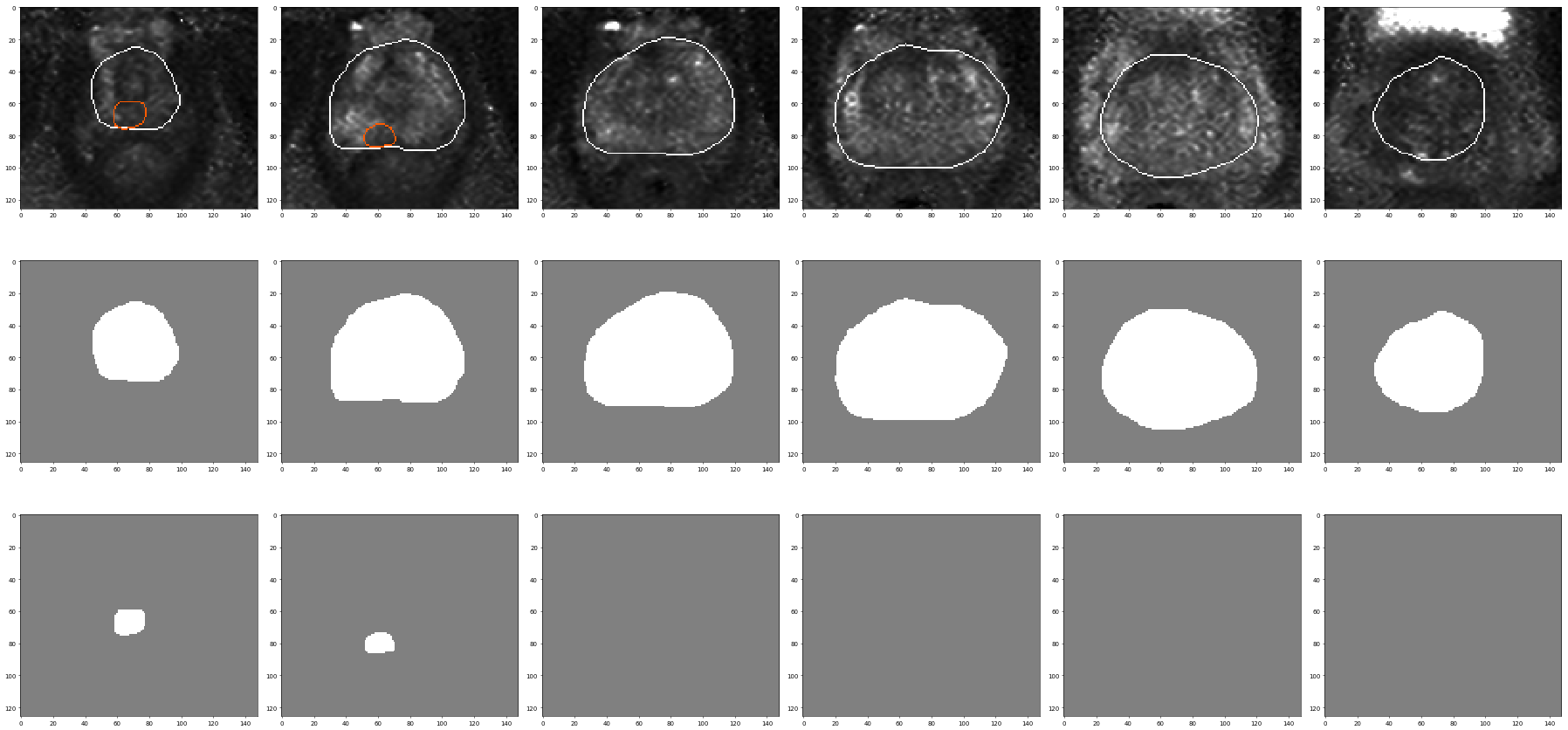

Supplement: S2 File — Files DWI-Mono-ADCm-xxx.png, T2-fitted-xxx.png, and T2w-std-xxx.png correspond to ADCm and T2 parametric maps, and T2-weighted images of each patient, respectively. On the first row of slices they show positions of regions of interest placed on the prostate cancer lesions (red, yellow) and around whole prostate (white). The prostate mask is on the second row, while the remaining rows are lesion masks. Files histology-xx.jpg contain the whole mount prostatectomy sections of each patient, with tumor outlines in green. Please note that identical MRI acquisition protocol has been used on all patients, including slice thickness. Here all prostate cancer masks are show with corresponding whole mount prostatectomy sections. (ZIP) [file pone.0217702.s002.zip › supporting_figures/T2-fitted-028.png]

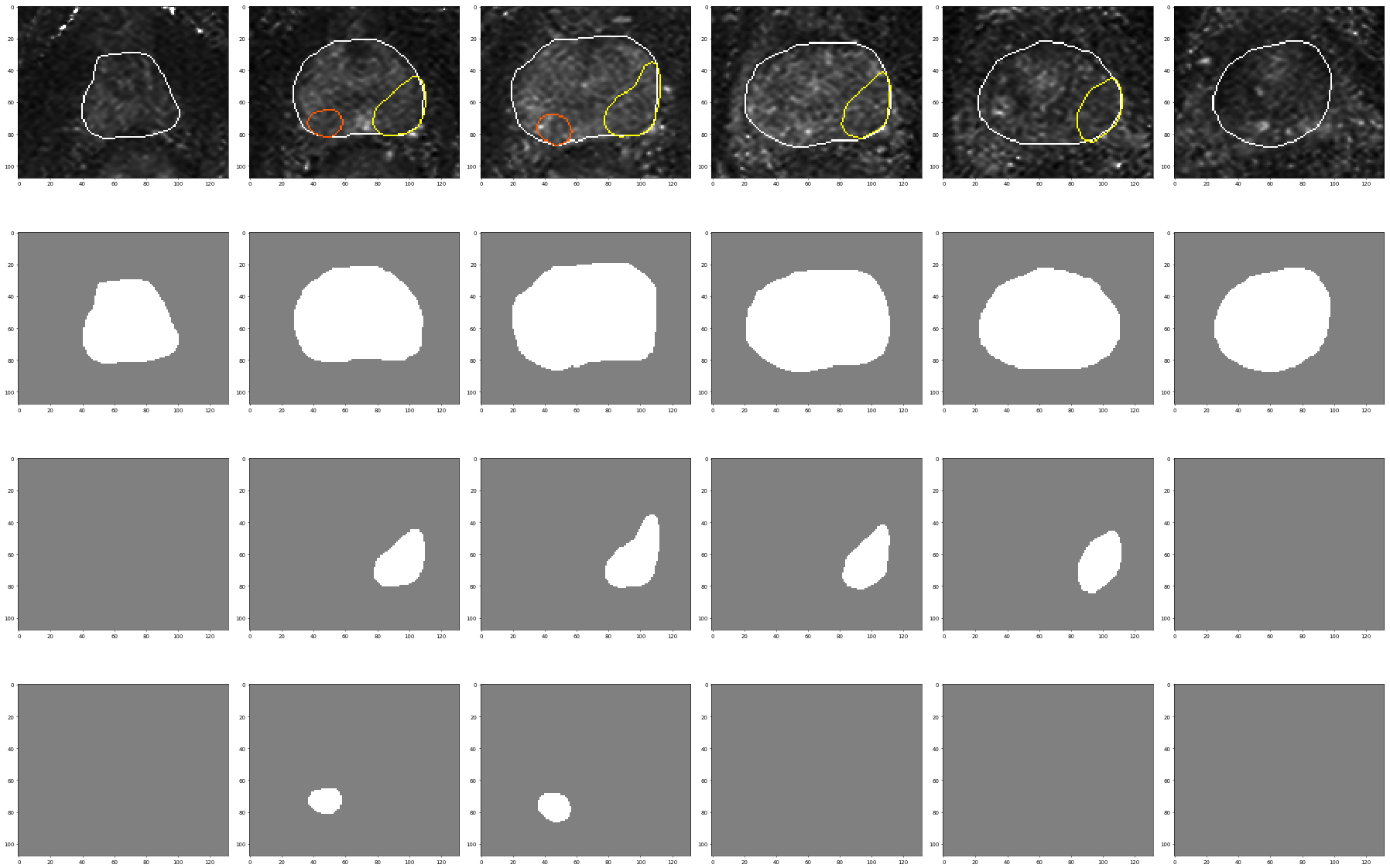

Supplement: S2 File — Files DWI-Mono-ADCm-xxx.png, T2-fitted-xxx.png, and T2w-std-xxx.png correspond to ADCm and T2 parametric maps, and T2-weighted images of each patient, respectively. On the first row of slices they show positions of regions of interest placed on the prostate cancer lesions (red, yellow) and around whole prostate (white). The prostate mask is on the second row, while the remaining rows are lesion masks. Files histology-xx.jpg contain the whole mount prostatectomy sections of each patient, with tumor outlines in green. Please note that identical MRI acquisition protocol has been used on all patients, including slice thickness. Here all prostate cancer masks are show with corresponding whole mount prostatectomy sections. (ZIP) [file pone.0217702.s002.zip › supporting_figures/T2-fitted-029.png]

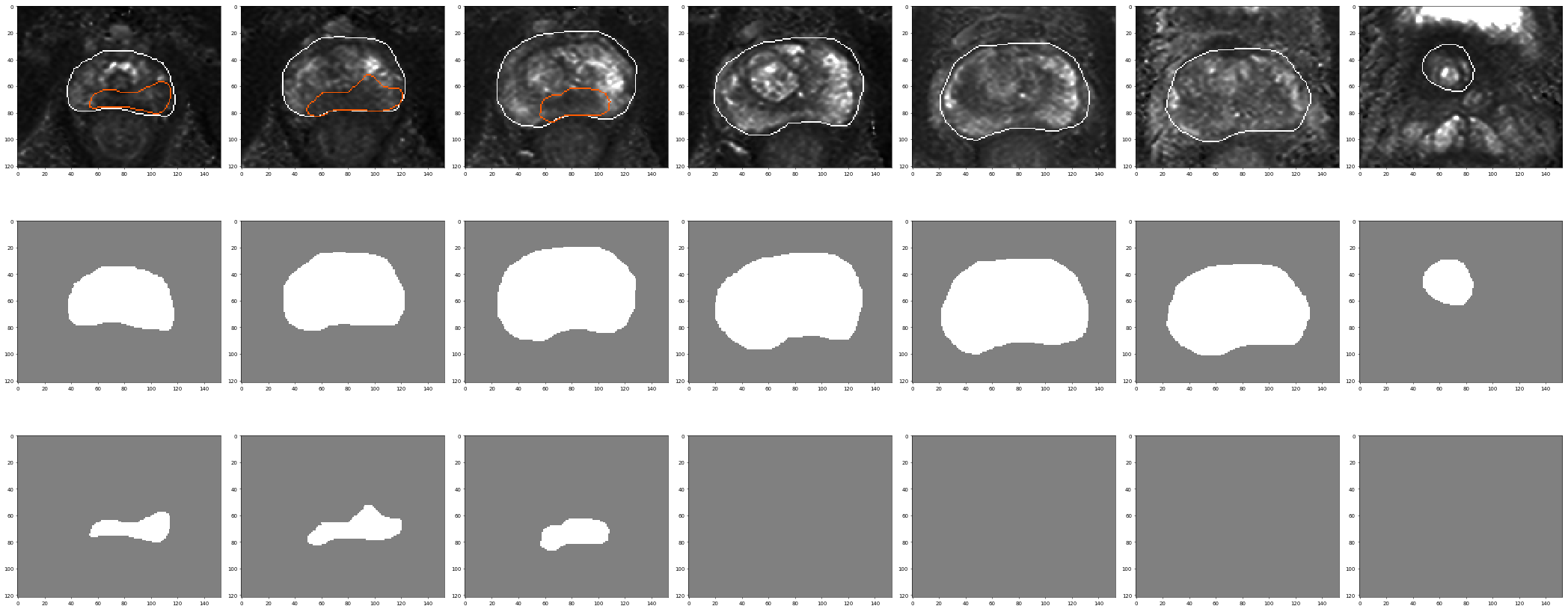

Supplement: S2 File — Files DWI-Mono-ADCm-xxx.png, T2-fitted-xxx.png, and T2w-std-xxx.png correspond to ADCm and T2 parametric maps, and T2-weighted images of each patient, respectively. On the first row of slices they show positions of regions of interest placed on the prostate cancer lesions (red, yellow) and around whole prostate (white). The prostate mask is on the second row, while the remaining rows are lesion masks. Files histology-xx.jpg contain the whole mount prostatectomy sections of each patient, with tumor outlines in green. Please note that identical MRI acquisition protocol has been used on all patients, including slice thickness. Here all prostate cancer masks are show with corresponding whole mount prostatectomy sections. (ZIP) [file pone.0217702.s002.zip › supporting_figures/T2-fitted-030.png]

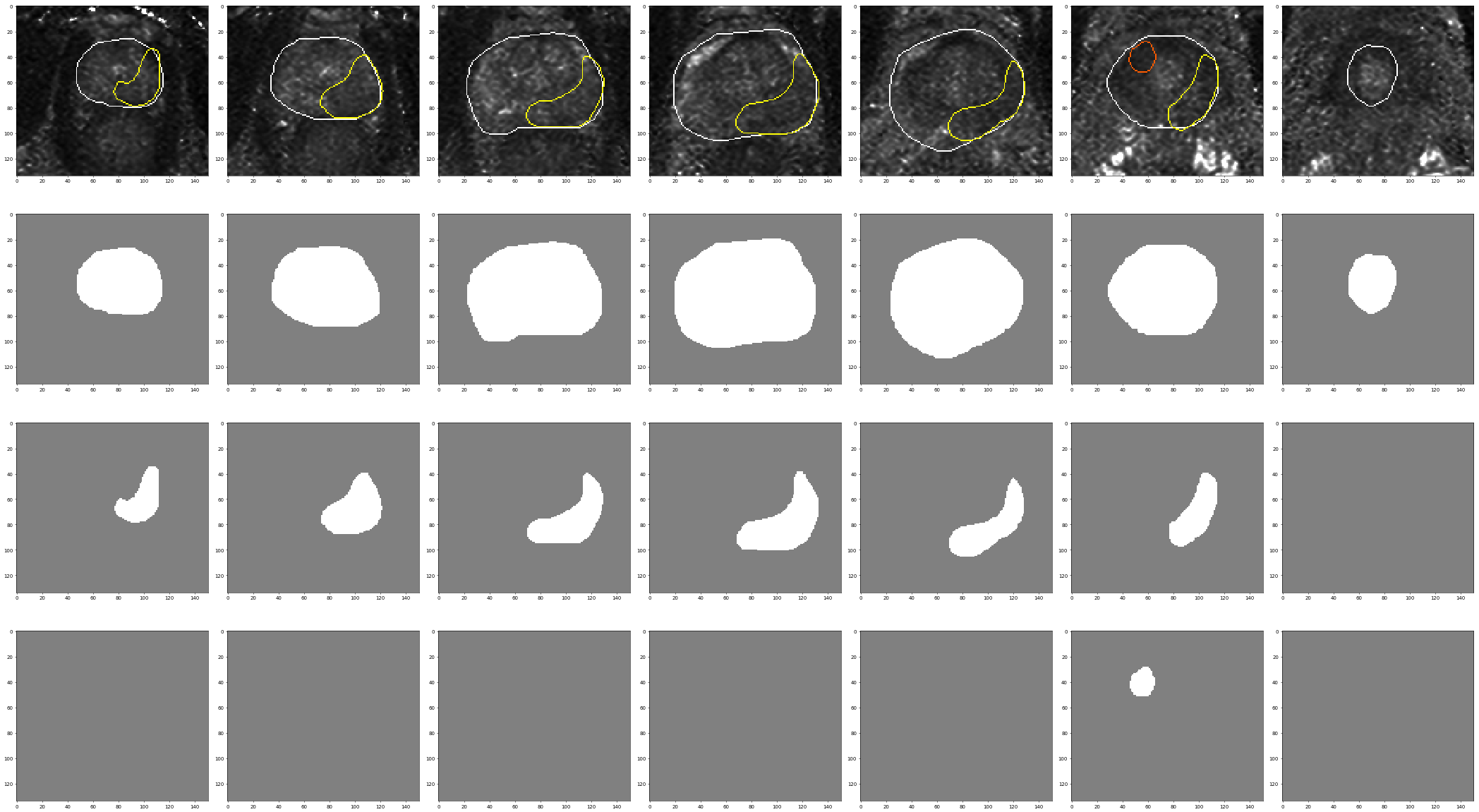

Supplement: S2 File — Files DWI-Mono-ADCm-xxx.png, T2-fitted-xxx.png, and T2w-std-xxx.png correspond to ADCm and T2 parametric maps, and T2-weighted images of each patient, respectively. On the first row of slices they show positions of regions of interest placed on the prostate cancer lesions (red, yellow) and around whole prostate (white). The prostate mask is on the second row, while the remaining rows are lesion masks. Files histology-xx.jpg contain the whole mount prostatectomy sections of each patient, with tumor outlines in green. Please note that identical MRI acquisition protocol has been used on all patients, including slice thickness. Here all prostate cancer masks are show with corresponding whole mount prostatectomy sections. (ZIP) [file pone.0217702.s002.zip › supporting_figures/T2-fitted-031.png]

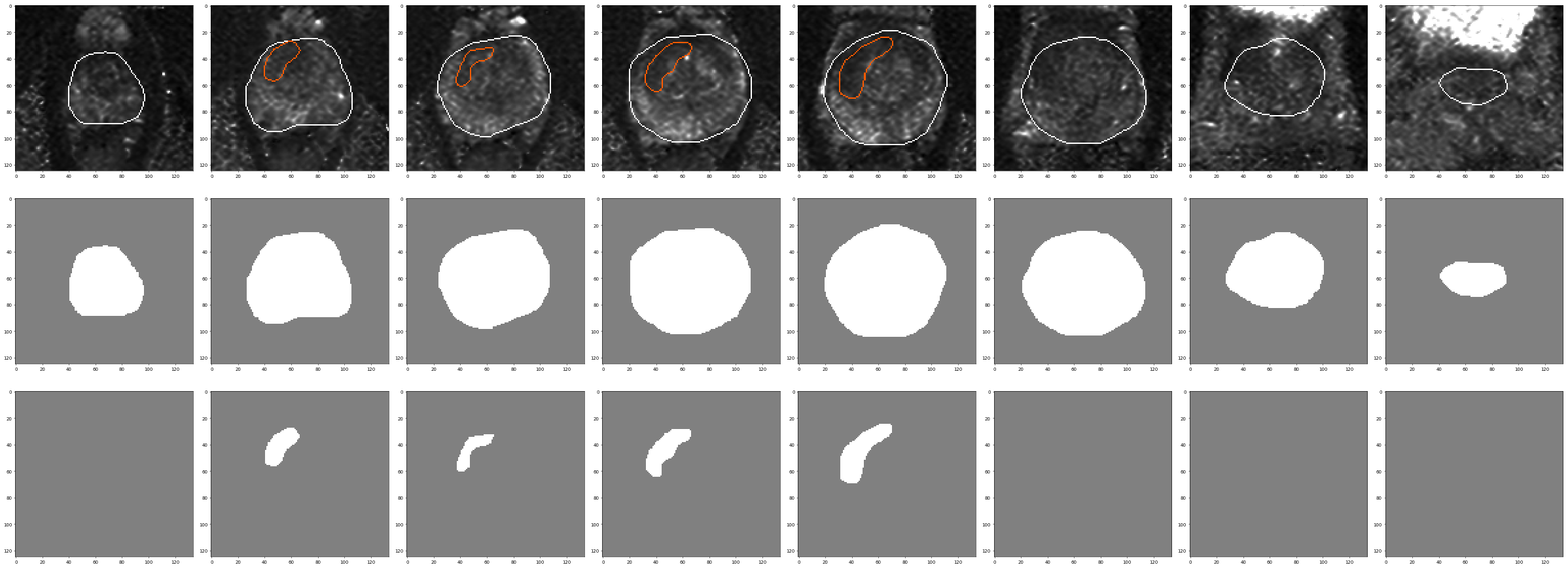

Supplement: S2 File — Files DWI-Mono-ADCm-xxx.png, T2-fitted-xxx.png, and T2w-std-xxx.png correspond to ADCm and T2 parametric maps, and T2-weighted images of each patient, respectively. On the first row of slices they show positions of regions of interest placed on the prostate cancer lesions (red, yellow) and around whole prostate (white). The prostate mask is on the second row, while the remaining rows are lesion masks. Files histology-xx.jpg contain the whole mount prostatectomy sections of each patient, with tumor outlines in green. Please note that identical MRI acquisition protocol has been used on all patients, including slice thickness. Here all prostate cancer masks are show with corresponding whole mount prostatectomy sections. (ZIP) [file pone.0217702.s002.zip › supporting_figures/T2-fitted-032.png]

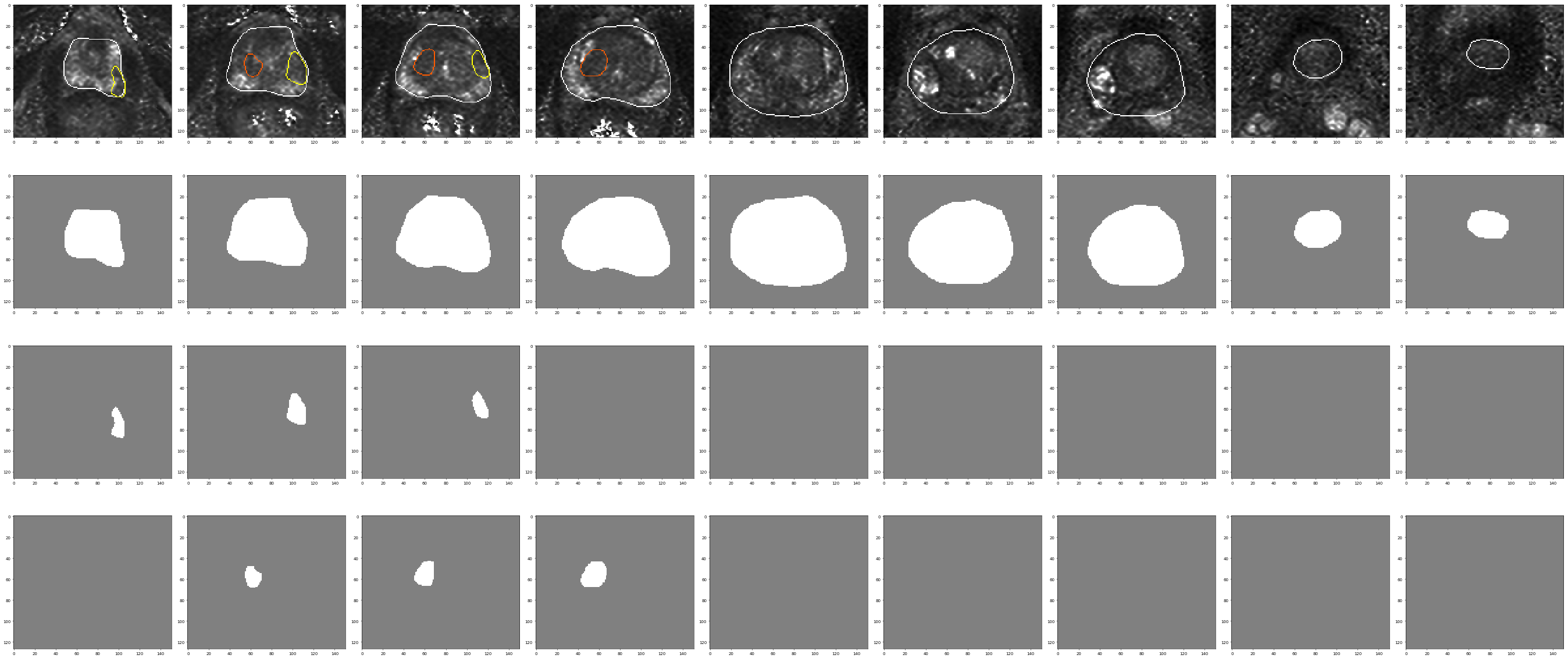

Supplement: S2 File — Files DWI-Mono-ADCm-xxx.png, T2-fitted-xxx.png, and T2w-std-xxx.png correspond to ADCm and T2 parametric maps, and T2-weighted images of each patient, respectively. On the first row of slices they show positions of regions of interest placed on the prostate cancer lesions (red, yellow) and around whole prostate (white). The prostate mask is on the second row, while the remaining rows are lesion masks. Files histology-xx.jpg contain the whole mount prostatectomy sections of each patient, with tumor outlines in green. Please note that identical MRI acquisition protocol has been used on all patients, including slice thickness. Here all prostate cancer masks are show with corresponding whole mount prostatectomy sections. (ZIP) [file pone.0217702.s002.zip › supporting_figures/T2-fitted-033.png]

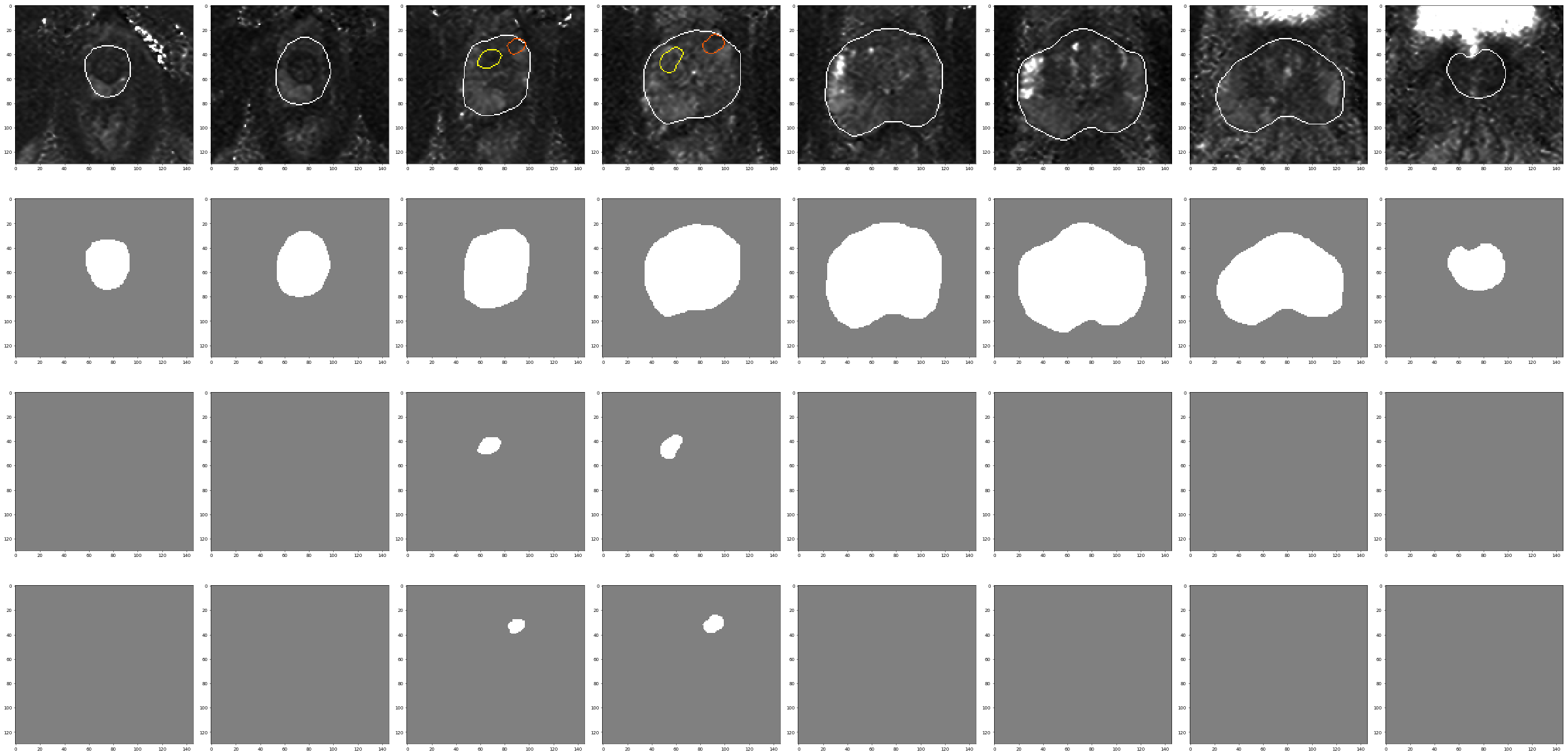

Supplement: S2 File — Files DWI-Mono-ADCm-xxx.png, T2-fitted-xxx.png, and T2w-std-xxx.png correspond to ADCm and T2 parametric maps, and T2-weighted images of each patient, respectively. On the first row of slices they show positions of regions of interest placed on the prostate cancer lesions (red, yellow) and around whole prostate (white). The prostate mask is on the second row, while the remaining rows are lesion masks. Files histology-xx.jpg contain the whole mount prostatectomy sections of each patient, with tumor outlines in green. Please note that identical MRI acquisition protocol has been used on all patients, including slice thickness. Here all prostate cancer masks are show with corresponding whole mount prostatectomy sections. (ZIP) [file pone.0217702.s002.zip › supporting_figures/T2-fitted-034.png]

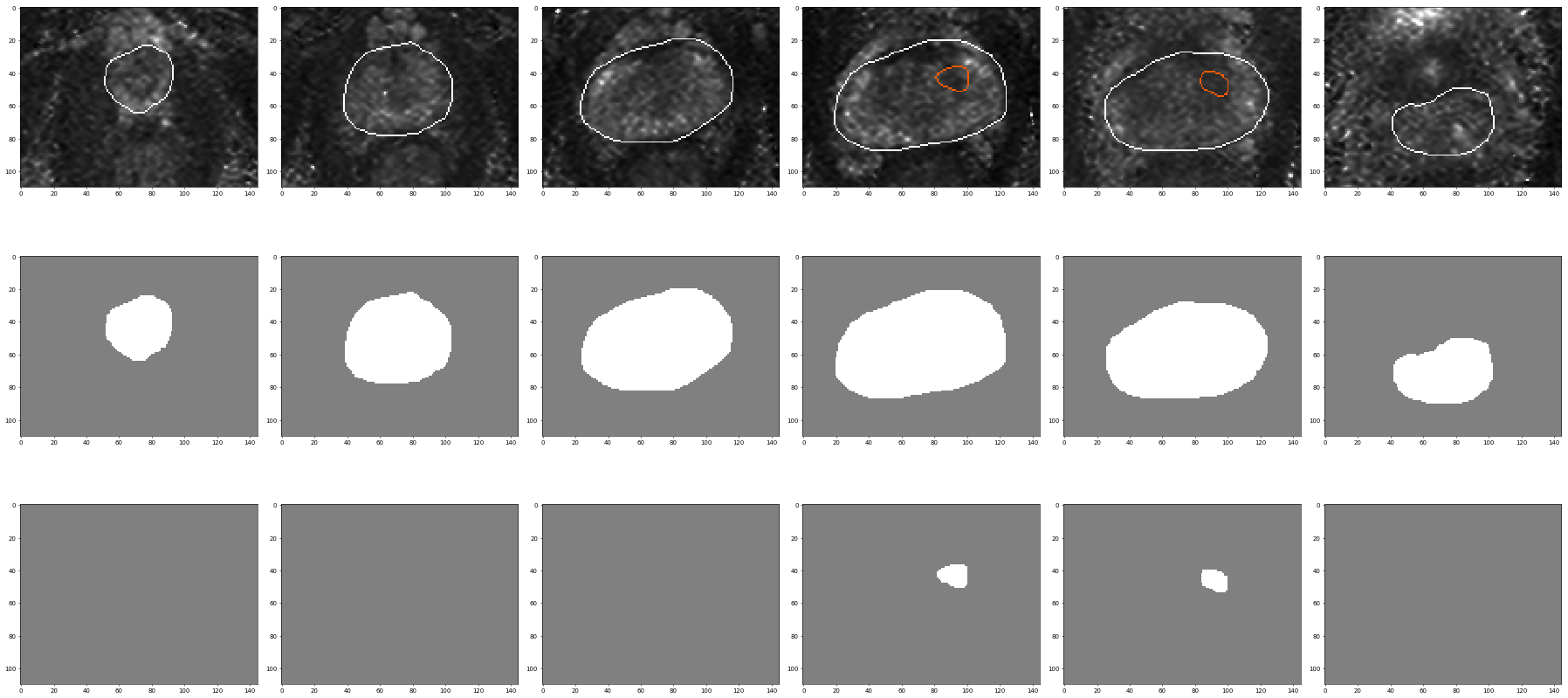

Supplement: S2 File — Files DWI-Mono-ADCm-xxx.png, T2-fitted-xxx.png, and T2w-std-xxx.png correspond to ADCm and T2 parametric maps, and T2-weighted images of each patient, respectively. On the first row of slices they show positions of regions of interest placed on the prostate cancer lesions (red, yellow) and around whole prostate (white). The prostate mask is on the second row, while the remaining rows are lesion masks. Files histology-xx.jpg contain the whole mount prostatectomy sections of each patient, with tumor outlines in green. Please note that identical MRI acquisition protocol has been used on all patients, including slice thickness. Here all prostate cancer masks are show with corresponding whole mount prostatectomy sections. (ZIP) [file pone.0217702.s002.zip › supporting_figures/T2-fitted-035.png]

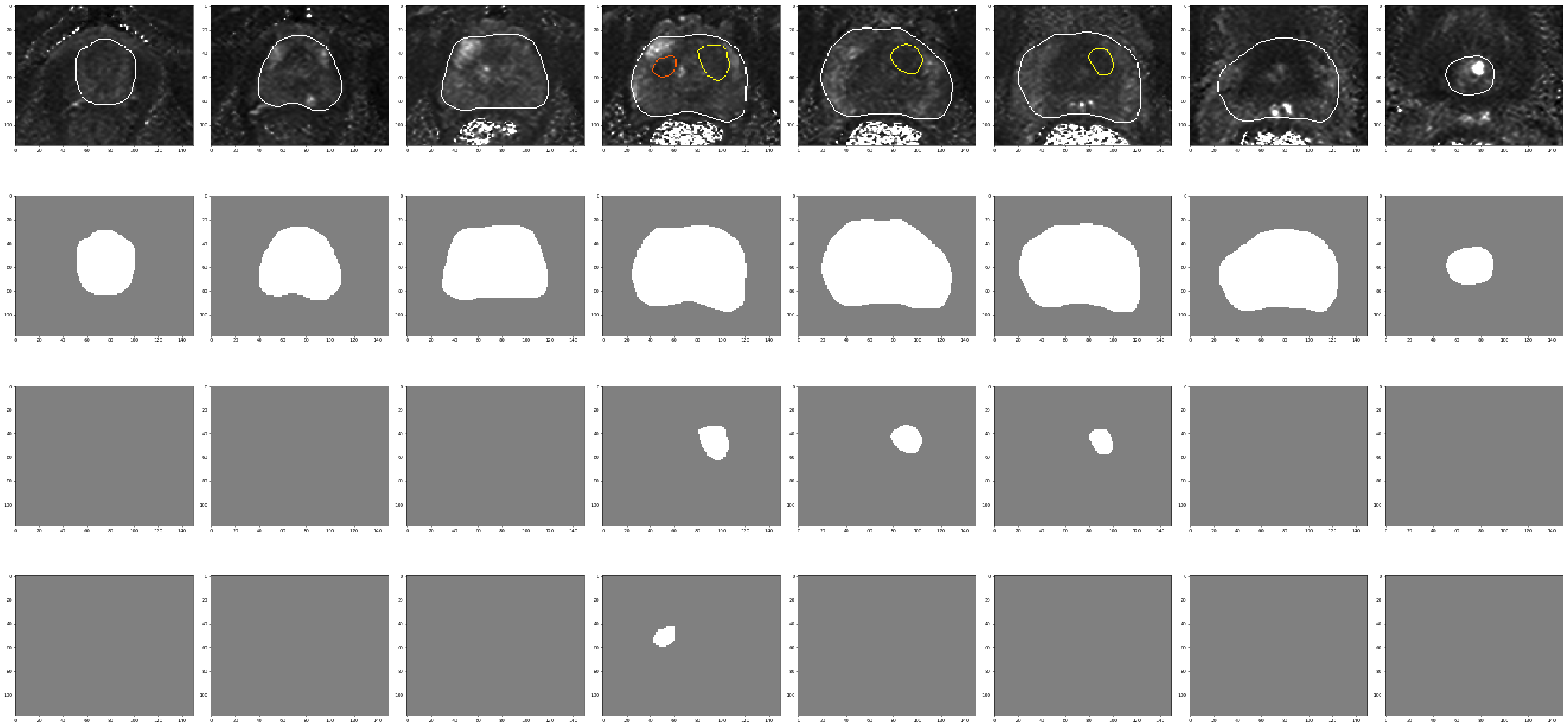

Supplement: S2 File — Files DWI-Mono-ADCm-xxx.png, T2-fitted-xxx.png, and T2w-std-xxx.png correspond to ADCm and T2 parametric maps, and T2-weighted images of each patient, respectively. On the first row of slices they show positions of regions of interest placed on the prostate cancer lesions (red, yellow) and around whole prostate (white). The prostate mask is on the second row, while the remaining rows are lesion masks. Files histology-xx.jpg contain the whole mount prostatectomy sections of each patient, with tumor outlines in green. Please note that identical MRI acquisition protocol has been used on all patients, including slice thickness. Here all prostate cancer masks are show with corresponding whole mount prostatectomy sections. (ZIP) [file pone.0217702.s002.zip › supporting_figures/T2-fitted-036.png]

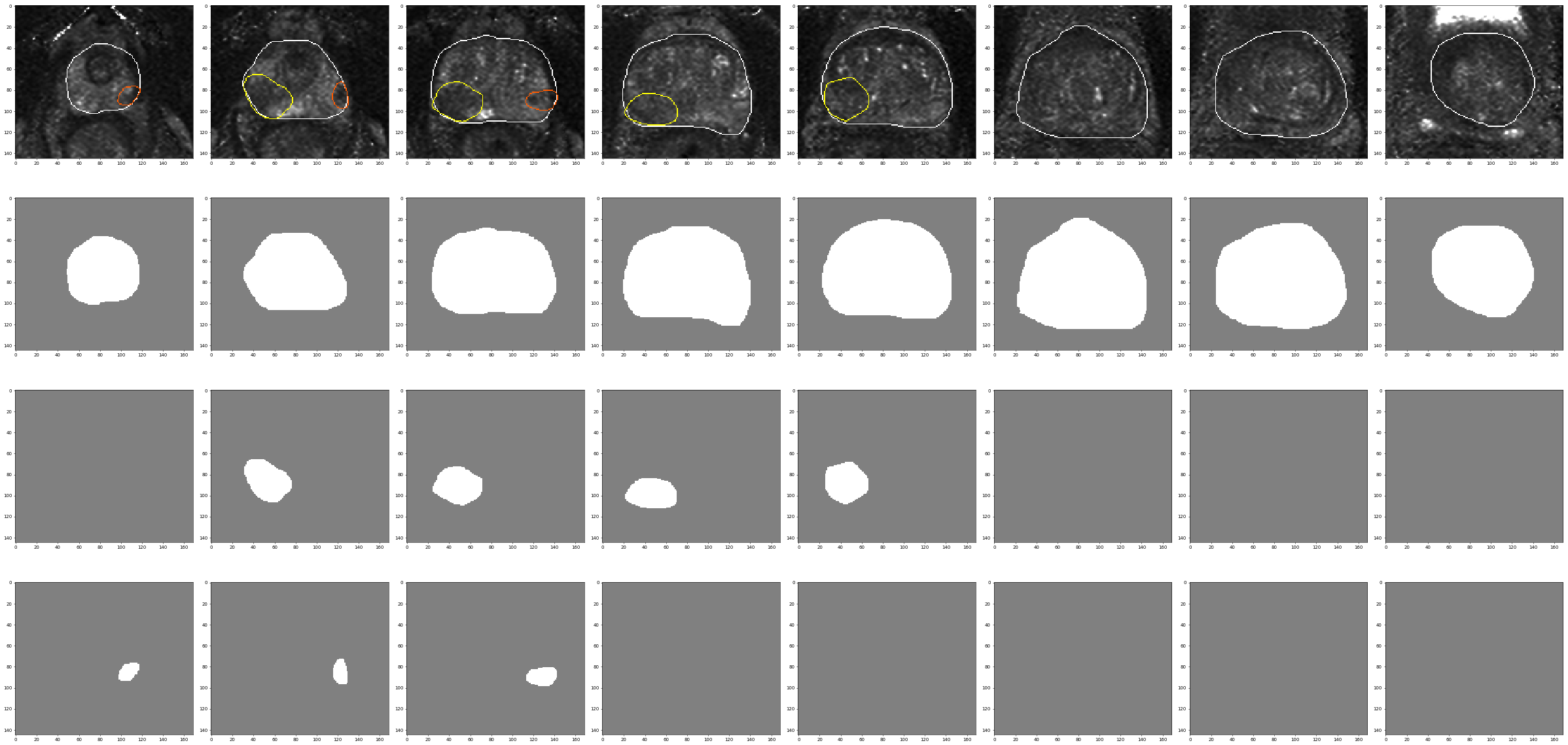

Supplement: S2 File — Files DWI-Mono-ADCm-xxx.png, T2-fitted-xxx.png, and T2w-std-xxx.png correspond to ADCm and T2 parametric maps, and T2-weighted images of each patient, respectively. On the first row of slices they show positions of regions of interest placed on the prostate cancer lesions (red, yellow) and around whole prostate (white). The prostate mask is on the second row, while the remaining rows are lesion masks. Files histology-xx.jpg contain the whole mount prostatectomy sections of each patient, with tumor outlines in green. Please note that identical MRI acquisition protocol has been used on all patients, including slice thickness. Here all prostate cancer masks are show with corresponding whole mount prostatectomy sections. (ZIP) [file pone.0217702.s002.zip › supporting_figures/T2-fitted-037.png]

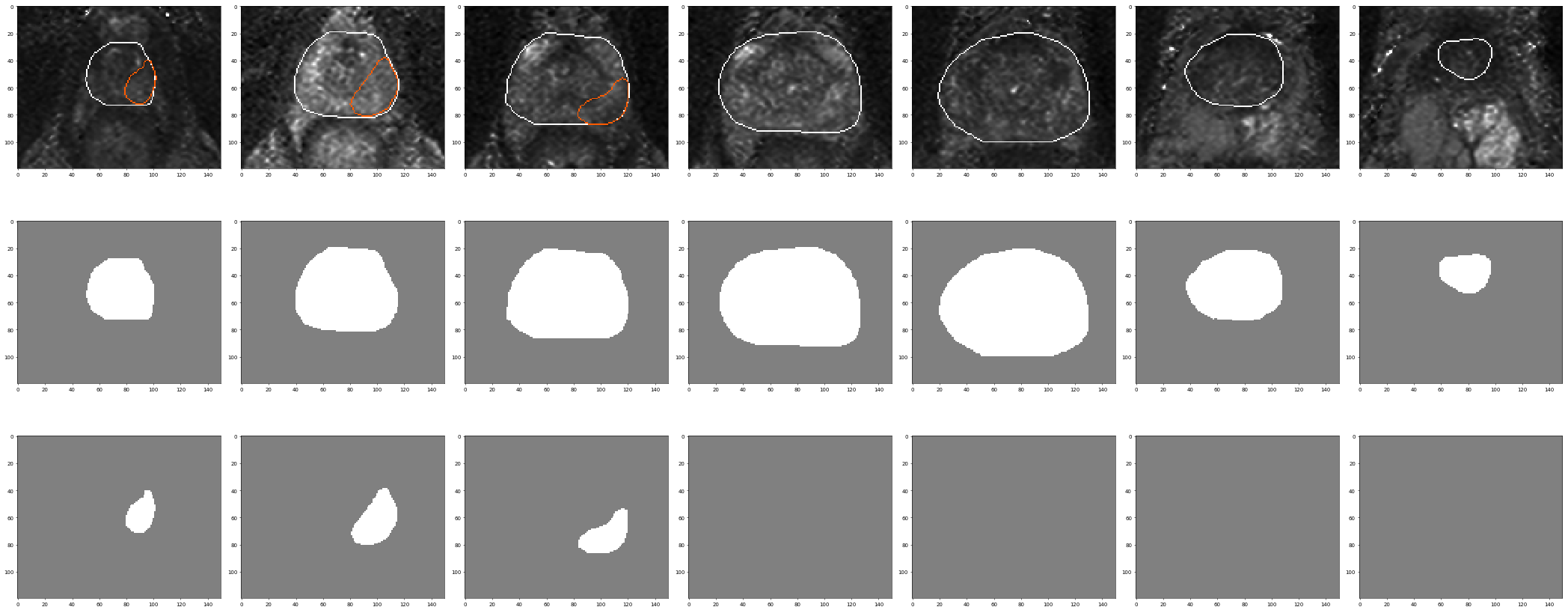

Supplement: S2 File — Files DWI-Mono-ADCm-xxx.png, T2-fitted-xxx.png, and T2w-std-xxx.png correspond to ADCm and T2 parametric maps, and T2-weighted images of each patient, respectively. On the first row of slices they show positions of regions of interest placed on the prostate cancer lesions (red, yellow) and around whole prostate (white). The prostate mask is on the second row, while the remaining rows are lesion masks. Files histology-xx.jpg contain the whole mount prostatectomy sections of each patient, with tumor outlines in green. Please note that identical MRI acquisition protocol has been used on all patients, including slice thickness. Here all prostate cancer masks are show with corresponding whole mount prostatectomy sections. (ZIP) [file pone.0217702.s002.zip › supporting_figures/T2-fitted-038.png]
